# Therapeutic strategies in vascular cognitive impairment: A systematic review and meta‐analysis

**DOI:** 10.1002/alz.70840

**Published:** 2025-11-06

**Authors:** Federico Masserini, Claudia Gendarini, Giacomo Baso, Emilia Salvadori, Leonardo Pantoni

**Affiliations:** ^1^ Neuroscience Research Center, Department of Biomedical and Clinical Sciences University of Milan Milan Italy; ^2^ Department of Radiology and Nuclear Medicine Amsterdam University Medical Centre Vrije Universiteit, Amsterdam Neuroscience Amsterdam the Netherlands; ^3^ Neurology Residency Program University of Milan Milan Italy; ^4^ Department of Neurorehabilitation Sciences Casa di Cura Igea Milan Italy

**Keywords:** cerebrovascular disease, clinical trials, cognitive impairment, meta‐analysis, therapy, vascular cognitive impairment

## Abstract

**INTRODUCTION:**

Vascular cognitive impairment (VCI) is a common, heterogeneous condition, currently lacking approved treatments.

**METHODS:**

We reviewed therapeutic strategies tested in VCI and meta‐analyzed efficacy data for eligible interventions to assess whether previously tested treatments warranted reconsideration.

**RESULTS:**

One‐hundred seventy‐three trials were extracted (22,347 participants, four VCI categories, 91 interventions, 145 outcomes). *Ginkgo biloba* extracts showed large to moderate improvements in cognition (Cohen's *d*: 0.83, 95% CI: 0.00 to 1.67) and small to moderate improvements in functional outcomes (Cohen's *d*: 0.50, 95% CI: 0.25 to 0.75). Small to moderate improvements in cognition were shown for acetylcholinesterase inhibitors, memantine, cerebrolysin, propentofylline, physical exercise, and cognitive rehabilitation.

**DISCUSSION:**

VCI clinical trials exhibited substantial heterogeneity. Few interventions were reproducibly tested in adequately sized and designed studies. Nonetheless, some interventions showed modest effects on global cognition and functional outcomes. To enhance the likelihood of success, future studies should focus on promising interventions with a solid rationale, target specific VCI subtypes, improve statistical power, and reduce heterogeneity.

**Highlights:**

- Considerable methodological heterogeneity across VCI trials undermined the strength of evidence for both positive and negative findings, ultimately decreasing confidence in the possibility of treating VCI.
- *Ginkgo biloba* extracts have shown the greatest cognitive and functional benefits, though their clinical significance remains uncertain, and certainty of evidence is overall low.
- Future trials should focus on a single VCI subtype, prioritize interventions with a strong mechanistic rationale, standardize diagnostic criteria, ensure adequate power for chosen outcomes, test against placebo, and implement FAIR data sharing to accelerate therapeutic discovery.

## 1 BACKGROUND

Vascular cognitive impairment (VCI) is a very common condition,^1^, ^2^ with recent studies suggesting that vascular contributions may be the most prevalent alterations in patients with cognitive impairment and dementia.^3^ VCI encompasses a wide spectrum of cognitive dysfunctions, associated with diverse pathological and clinical substrates (e.g., multi‐infarct dementia, post‐stroke dementia, small‐vessel disease). Effective therapeutic options for VCI are urgently needed, given its significant burden on patients and caregivers.^4^ Moreover, as cerebrovascular alterations are frequently present in patients with any type of cognitive decline, finding effective treatments for VCI may have potential benefits in mitigating the vascular contribution to cognitive impairment of other origins.

Numerous therapeutic approaches have been evaluated in patients with VCI, but no approved disease‐modifying or symptomatic treatment is currently registered. This may be partly due to the inherent heterogeneity of VCI, which hinders the identification of a single effective treatment. A potential underestimation of the efficacy of some treatments was recently suggested based on the heterogeneity of included populations, the use of outcome measures not primarily designed for VCI, and the short duration of many trials.^5^

This work aims to review the effects of all therapeutic strategies tested in patients with VCI and, when possible, perform meta‐analyses to explore whether some treatments have some potential unrecognized benefits. By recognizing potential pitfalls in previous studies, we also aimed to help pave the way for better designing future VCI treatment trials.

## 2 METHODS

We conducted a systematic literature search of MEDLINE, Embase, and the Cochrane Central Register of Controlled Trials (CENTRAL) from inception to June 23, 2025 (Prospero CRD: 4202127093). We used a comprehensive search string with relevant keywords (detailed in eMethods Section 1.2) to identify studies investigating any therapeutic intervention for VCI. We focused specifically on trials evaluating disease‐modifying or symptomatic effects in patients with established VCI; therefore, we excluded studies assessing secondary prevention of cognitive impairment (e.g., after stroke). We included randomized clinical trials (RCTs) and non‐RCTs, enrolling patients with any degree of cognitive impairment due to a vascular substrate, testing intervention either versus placebo or versus other treatments, and measuring any outcome. Additionally, we reviewed published Cochrane Reviews on VCI interventions (a complete list of the reviewed Cochrane Reviews is reported in Supplementary File 1) and the bibliographies of other identified systematic and narrative reviews to ensure comprehensiveness. Search results were uploaded to Covidence systematic review software, Veritas Health Innovation, Melbourne, Australia (available at www.covidence.org).

Duplicate entries were reviewed automatically and removed before screening. Prevention studies, non‐human studies, studies involving mixed dementia populations (e.g., AD plus vascular), studies not reporting separate results for VCI patients, and studies not published in English were excluded.

Three reviewers (GB, CG, and FM) independently screened non‐duplicate retrieved records in randomized pairs. Potentially relevant studies underwent full‐text review for eligibility and data extraction using an electronic database. Discrepancies between reviewers at any stage were resolved through consensus with a fourth investigator (LP). Detailed search logs for each screening step were recorded.

Extracted data included study design, participant characteristics, intervention class (pharmacological, rehabilitation, non‐pharmacological non‐rehabilitative), specific intervention with relevant details, comparators, and assessed outcomes with corresponding data at study time‐points (full list of extracted data in the eMethods Section 1.1.1). Enrollment diagnoses were categorized by VCI subtype (“multi‐infarct,” “post‐stroke,” “subcortical vascular”, “small‐vessel disease,” or “vascular”) and cognitive impairment degree (dementia, mild cognitive impairment, or unspecified). We extracted up to seven outcomes per study, prioritizing primary and cognitive outcomes. The quality of each included study was assessed using the National Institute of Health Quality Assessment Tool for controlled intervention studies (available at www.nhlbi.nih.gov/health‐topics/study‐quality‐assessment‐tools), and the efficacy of each study was awarded an overall rating based on reported results (eMethods Section 1.1.1).

### 2.1 Meta‐analysis

Interventions tested in at least three studies as monotherapy against inactive treatment were considered for meta‐analysis (further methodological details in eMethod Section 1.1.2). Where data availability permitted, meta‐analyses were done for overall cognitive function, functional parameters (i.e., outcomes assessing real‐world abilities and determining how well an individual can perform everyday activities, such as activities of daily living scales or functional independence measures like the modified Barthel Index), patient‐reported outcomes (quality of life, self‐reported measures), safety measures, and instrumental (surrogate) outcomes, using fixed‐ or random‐effects models according to statistical heterogeneity.

Interventions unsuitable for meta‐analysis were synthesized narratively and presented with summary tables, organized by the outcome classes used in the meta‐analyses. Furthermore, for monotherapy studies with an inactive treatment comparator, not included in the meta‐analyses, effect sizes (ES) were calculated for overall cognitive performance outcomes.

Continuous meta‐analyzed outcomes were presented as Cohen's *d* effect sizes (and unstandardized mean differences when studies employed the same outcome measure), dichotomous outcomes as odds ratios, and safety outcomes as rate ratios. Cohen's *d* effect size magnitude was interpreted according to a standard framework.^6^ The GRADE approach was used to assess the certainty of evidence for meta‐analyzed outcome classes,^7^ as outlined within the Supplementary Materials. Descriptive analyses for reported variables were conducted with IBM SPSS Statistics (version 29.0). Meta‐analyses were performed with Prometa (version 3, Internovi, 2015).

**RESEARCH IN CONTEXT**

1. **Systematic review**: We systematically reviewed interventional trials in VCI and meta‐analyzed treatments tested in ≥3 studies. The 173 included trials (22,347 participants) showed wide heterogeneity in populations, interventions, and outcomes. Notably, many lacked placebo‐control despite no approved VCI treatments. Meta‐analyses revealed global cognition improvements with *Ginkgo biloba*, AChE inhibitors, memantine, cerebrolysin, propentofylline, physical exercise, and cognitive rehabilitation. Small to moderate functional improvements were demonstrated with *Ginkgo biloba*.
2. **Interpretation**: The considerable heterogeneity across prior VCI studies has likely weakened the strength of evidence regarding the efficacy of evaluated treatments. Nevertheless, certain interventions have demonstrated promising cognitive benefits in VCI patients.
3. **Future directions**: Given the lack of approved therapies for VCI and its significant prevalence, identifying effective treatments remains crucial. Future research should focus on specific and homogeneous VCI subtypes, investigate drugs with strong biological rationale, and employ condition‐specific outcome measures. Overall, a completely pessimistic view regarding VCI treatment may be unjustified.

## 3 RESULTS

**FIGURE 1:**
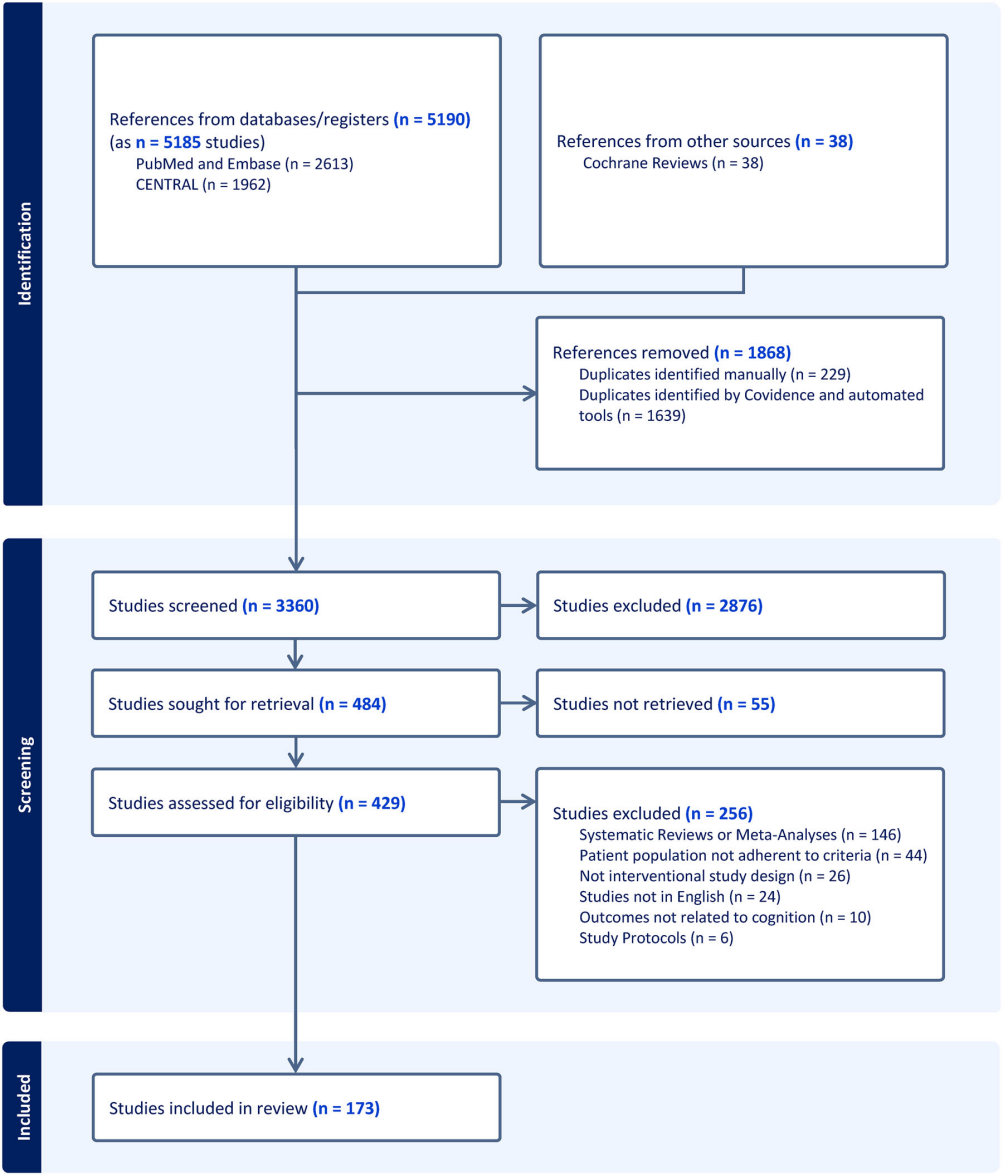
Flowchart of our systematic review research and screening process according to 2020 PRISMA guidelines for systematic reviews.

The titles and abstracts of 5190 unique entries were screened for eligibility (PRISMA flowchart is reported in Figure 1). Of the 429 eligible and retrieved full texts, 146 were excluded because they were systematic reviews or meta‐analyses, 44 because the included population did not fit the inclusion criteria (mostly because including participants with mixed dementia or because they were secondary prevention studies), 26 because they were not interventional studies by design (e.g., retrospective studies, case‐control studies, studies with no “control” arm), and 10 because they did not include any cognitive‐related variable. We identified 173 eligible articles, of which most were RCTs (*n = *160). Overall, 22,347 participants were enrolled across the four different diagnostic VCI labels. Sixty‐two studies were selected for meta‐analysis. Comprehensive lists of included studies, studies excluded after full‐text screening, and the screened Cochrane Reviews are provided in Supplementary Files 1–3 of the Supplementary Materials.

### 3.1 Characteristics of included studies

**FIGURE 2:**
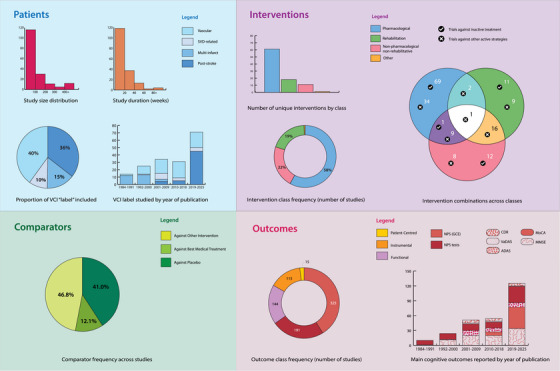
Overview of patient characteristics, interventions, comparators, and outcomes in included studies, organized in visual summary panels. Panels include simplified versions of fully detailed figures included within supplementary materials with specific reference to full figure for further consultation. Patients (top left) features a histogram of study size distribution and of study duration (Ref: eFigures 2 and 8), a pie chart depicting proportion of VCI label included, as well as a breakdown on VCI label use over time (Ref: eFigure 4). Interventions (top right) includes a bar chart of unique interventions reviewed, grouped by intervention class (pharmacological, rehabilitative, non‐pharmacological non‐rehabilitative), together with overall class frequency across studies (Ref: eFigure 6); a Venn diagram showing how these interventions were assessed, including whether they were used in monotherapy or combination therapy, and the types of comparators used (placebo or active intervention) is also provided (Ref: eFigure 5). Comparators (bottom left) features pie chart depicting proportion of comparator use (placebo, best medical treatment, or other intervention, i.e., head‐to‐head studies) across studies. Finally, Outcomes provides a donut chart of outcome class frequency (Ref: eFigure 7) as well as the frequency of use over time of the main global cognitive efficiency outcomes (Ref: eFigure 9). ADAS, Alzheimer's Disease Assessment Scale; CDR, Clinical Dementia Rating scale; GCE, global cognitive efficiency; incl., including; MMSE, Mini‐Mental State Examination; MoCA, Montreal Cognitive Assessment; NPS, neuropsychological; SVD, small‐vessel disease; VaDAS, Vascular Dementia Assessment Scale; VCI, vascular cognitive impairment.

A graphical summary of the main findings in terms of patients included, interventions investigated, comparators employed, and featured outcomes is depicted in Figure 2.

Included participants differed both in terms of nosological entity (i.e., the vascular “substrate” underlying cognitive decline) and degree of cognitive impairment (participants section of Figure 2 and in Table 1). Subspecification of VCI label occurred in 98 studies (56.6%). Within the studies enrolling participants according to a specific VCI label, the criteria for defining the vascular component and the degree of cognitive impairment varied, encompassing different combinations of established criteria (e.g., National Institute of Neurological Disorders and Stroke ‐ Association Internationale pour la Recherche et l'Enseignement en Neurosciences, NINDS‐AIREN, and Diagnostic Statistics Manual of Mental Disorder, DSM criteria), clinical or radiological parameters, and cognitive pre‐requisites (e.g., Montreal Cognitive Assessment, Mini‐Mental State Examination, Alzheimer's Disease Assessment Scale Cognition scores). Further details are available in the Supplementary Materials (eResults Section 2A.1‐2, eTable 1, eFigures 2–4).

Overall, 91 different types of interventions were evaluated: 61 pharmacological interventions, 18 rehabilitative interventions, and 11 non‐pharmacological non‐rehabilitative, and one other type of intervention. Fifty‐six studies investigated ≥2 interventions. Inactive treatment (placebo or best medical care) was employed as comparator in 92 trials (53.2%). The frequency of intervention classes investigated (stratified by comparator type), along with the most frequently studied single interventions, is presented in eFigures 5 and 6.

Efficacy was assessed across 145 different outcomes (eFigure 7), categorized as cognitive‐behavioral (*n = *49), instrumental (*n = *45), functional (*n = *40), and patient‐centered (*n = *11). These were primarily evaluated at the end of planned interventions, with 33 (19.1%) trials including also a follow‐up period. Overall, the mean duration of intervention was 18.01 weeks (SD 21.01, distribution of treatment duration is represented in Figure 2, *Participants* section, and eFigure 8; further details on the interventions included and the outcomes selected are provided in Supplementary Materials Sections 2A.3–5 and eFigures 9 and 10).

Overall, study findings were reported in favor of the investigated interventions in 86% of cases (Supplementary Results Section 2A.7, eFigure 11).

The quality of the studies included, as rated according to the National Institutes of Health (NIH) Quality Assessment tool for controlled intervention studies, is reported in Supplementary File 4. Overall, 75 studies (43%) were of good, 68 (39%) of fair, and 30 (17%) of poor quality. The five categories most frequently rated as having a high or unclear risk of bias were sample size estimation techniques (70% of studies), participant/operator blinding (52% of studies), treatment adherence (51% of studies), blinding of outcome assessors (48% of studies), and employed randomization methods (47% of studies). Overall quality ratings (and ratings broken down by NIH quality assessment tools items) along study quality time trend is reported in eFigure 12 and 13.

### 3.2 Qualitative synthesis of efficacy data

Data from all non‐meta‐analyzed studies (*n* = 111) were synthesized qualitatively. Fifty‐two unique pharmacological interventions (*n* = 64 studies) – encompassing diverse drug categories, essential elements, and various complementary and alternative medicines – six rehabilitative strategies (*n* = 10 studies), nine non‐pharmacological non‐rehabilitative strategies (*n* = 36 studies), and one other intervention were excluded from meta‐analyses. Detailed reviews and data syntheses for each intervention strategy are presented in eTable 2 (pharmacological interventions), eTable 3 (rehabilitative strategies), eTable 4 (non‐pharmacological non‐rehabilitative interventions), and eTable 5 (other strategies) of the Supplementary Materials. Studies specifically evaluating post‐stroke and subcortical vascular VCI subtypes have also been summarized in thematic eTables 6 and 7.

**FIGURE 3:**
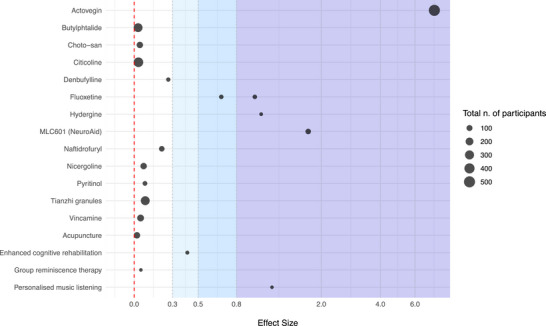
Scatter plots representing point estimates of effect size magnitude for global cognitive efficiency outcomes for studies not included in meta‐analyses. Effects sizes are expressed as Cohen's *d*, and dot size is proportional to study sample size. Areas of graph were colored in shades of greater intensity (from white to dark blue) to represent effect size magnitude: 0 to 0.3, small to negligible effect; 0.3 to 0.5, small effect; 0.5 to 0.8, moderate effect; higher than 0.8, large effect.

For 39 studies comparing monotherapy interventions with inactive treatment, a point estimate of effect size magnitude for global efficiency outcomes is provided, if possible and for illustrative purposes only, in Figure 3. The remaining 70 studies evaluated interventions either in combination or against other active treatment strategies (a complete list is provided in Supplementary File 5). For example, of the 22 trials evaluating acupuncture (including 16 studies on traditional acupuncture, four studies on electroacupuncture, and two studies on “yi qi tiao xue, fu ben pei yuan”), only four investigated it in monotherapy against placebo (two electroacupuncture and two traditional acupuncture); all the other studies investigated acupuncture against other interventions, either as monotherapy (*n* = 4 studies) or in combination with other interventions (*n* = 14 studies).

### 3.3 Meta‐analysis of efficacy data

A total of 14 interventions were included in meta‐analyses (as investigated as monotherapy against inactive treatment in three or more studies): *Ginkgo biloba* extracts (EGb 761), acetylcholinesterase inhibitors (rivastigmine, galantamine, and donepezil), memantine, vasodilatory drugs (pentoxifylline, propentofylline, nimodipine), nootropics (cerebrolysin), remote ischemic conditioning, cerebral stimulation techniques (repetitive transcranial magnetic stimulation, and transcranial direct current stimulation), cognitive rehabilitation, and physical exercise. Efficacy results for each of these candidate interventions were quantitatively pooled in individual meta‐analyses according to prespecified outcome classes (global cognitive efficiency, functional outcomes, patient‐centered outcomes, safety outcomes). A complete list of interventions excluded from meta‐analyses is provided in the Supplementary Materials, Section 2C.1.

Some interventions were associated with large to moderate efficacy on global cognitive efficiency at the end of the intervention including (in order of effect size) *Ginkgo biloba* extracts (Cohen's *d* ES, 0.83, 95% CI: 0.00 to 1.67, Unstandardized Mean Difference +2.56, 95% CI: −0.17 to 5.29, outcome: Short Cognitive Performance Test [SKT]), cognitive training (ES 0.71, 95% CI: 0.10 to 1.32), and physical exercise (Cohen's *d* ES: 0.60, 95% CI: 0.23 to 0.97). Other interventions were associated with moderate to low efficacy on global cognitive efficiency, including propentofylline (ES 0.44, 95% CI: 0.06 to 0.82), memantine (ES 0.33, 95% CI: 0.11 to 0.55), cerebrolysin (ES 0.28, 95% CI: 0.03 to 0.52), and AChE inhibitors (galantamine and donepezil, ES 0.28, 95% CI: 0.15 to 0.41 and 0.29, 95% CI: 0.17 to 0.41). Data on functional endpoints were reported for a limited number of interventions (and indeed pentoxifylline, propentofylline, galantamine, remote ischemic conditioning, and cerebrolysin lacked sufficient data for meta‐analysis). Of those reporting them, efficacy in improving functional endpoints was demonstrated for (in order of effect size) *Ginkgo biloba* extracts (ES 0.5, 95% CI: 0.25 to 0.75) and donepezil (ES 0.11, 95% CI: 0.04 to 0.19). All meta‐analyses refer to effects measured at the end of treatment, as follow‐up data were insufficient for pooling in all cases, except for physical exercise. In this instance, the statistically significant effect demonstrated after the end of treatment did not persist after the end of follow‐up.

None of the meta‐analyzed treatments showed an increased rate of adverse events or severe adverse events within the active intervention group.

No study reported sufficient data on patient‐centered metrics. Only studies on remote ischemic conditioning provided enough data for meta‐analysis of instrumental outcomes (details in Supplementary Materials Section, 2C.11).

Statistical heterogeneity for global cognitive efficiency outcomes was moderate to substantial for seven out of 14 interventions (50%). It was low to negligible for functional outcomes, with none of the interventions showing substantial heterogeneity for these outcomes.

**FIGURE 4:**
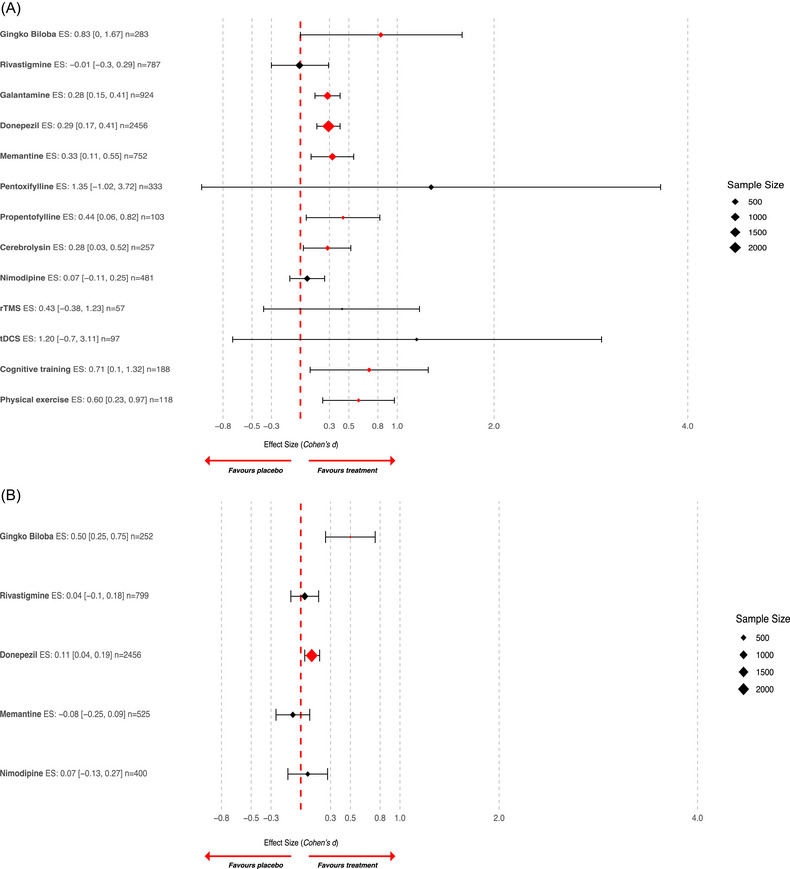
Forest plot of meta‐analysis of global cognitive efficiency outcomes (A) and of functional outcomes (B) for all meta‐analyzed interventions providing enough data for meta‐analysis. Effect sizes (expressed as Cohen's *d*) along with their 95% confidence interval are reported. Statistically significant differences are highlighted in red; diamond size is proportional to total number of participants included in meta‐analysis.

Meta‐analyses of single treatment efficacy, with study characteristics, forest plots, and adverse events, are detailed in the Supplementary Materials (Sections 2C.2–15 and eFigures 14–69). All‐treatment meta‐analysis results, aggregated by outcome, are presented in Table 1A, 1B and depicted in Figure 4 and eFigure 70.

**TABLE 1A:** Summary tables of meta‐analysis of treatment efficacy in vascular cognitive impairment. A. Summary of treatment efficacy on *global cognitive efficiency metrics*.

Intervention	GCE outcomes treatment duration	*No*. participants (*n* of studies)	VCI population	Efficacy metric	Quality of evidence (GRADE)	Statistical heterogeneity	Studies
*Ginkgo biloba*	*SKT* *Duration: 22 to 24 weeks (no follow‐up)*	283 (three RCTs)	*Vascular dementia* *MID*	**Cohen's *d***: 0.83, 95% CI: 0.00 to 1.67 **Mean difference**: + 2.56, 95% CI: −0.17 to 5.29	⊕○○○ Very low^a^, ^b^, ^c^	*I* ^2^ = 87.71	[23] [36] [43]
*Rivastigmine*	*ADAS‐CoG, MMSE Duration: 24–26 weeks (no follow‐up)*	*787 (three RCTs)*	*Subcortical vascular dementia Vascular dementia post‐stroke MCI*	**Cohen's *d***: ‐0.01, 95% CI: −0.30 to 0.29	⊕○○○ Very low ^a^, ^c^, ^d^, ^e^, ^f^	*I* ^2^ = 39.10	[97] [93] [82]
*Galantamine*	*ADAS‐CoG Duration: 6 months (no follow‐up)*	*924 (two RCTs)*	*Vascular dementia*	**Cohen's *d***: 0.28, 95% CI: 0.15 to 0.41	⊕⊕⊕○ Moderate^e^	*I* ^2^ = 0	[65] [83]
*Donepezil (all doses)*	*ADAS‐CoG, VaDAS‐Cog Duration: 24–26 weeks (no follow‐up)*	*2456 (four RCTs)*	*Vascular dementia*	**Cohen's *d***: 0.29, 95% CI: 0.17 to 0.41	⊕⊕⊕○ Moderate^d^	*I* ^2^ = 54.40	[31] [33] [78] [86]
*Memantine*	*ADAS Duration: 28 weeks (no follow‐up)*	*525 (two RCTs)*	*Vascular dementia*	**Cohen's *d***: 0.33, 95% CI: 0.11 to 0.55	⊕⊕⊕○ Moderate^e^	*I* ^2^ = 52.54	[21] [32]
*Pentoxifylline*	*ADAS, GBS Duration: 36–38.5 weeks (no follow‐up)*	*333 (two RCTs)*	*Vascular dementia – MID*	**Cohen's *d***: 1.35, 95% CI: −1.02 to 3.72	⊕○○○ Very low^a^, ^d^, ^g^	*I* ^2^ = 98.74	[79] [81]
*Propentofylline*	*SKT, MMSE Duration: 12 – 52 weeks (no follow‐up)*	*116 (two RCTs)*	*Vascular dementia*	**Cohen's *d***: 0.44, 95% CI: 0.06 to 0.82	⊕⊕○○ Low^d^, ^f^	*I* ^2^ = 0	[26] [54]
*Cerebrolysin*	*MMSE, ADAS‐CoG Duration: 4 weeks. Follow‐up after treatment: reported by only 1 RCT (not analysed)*	173 (*two* RCTs)	*Vascular dementia*	**Cohen's *d***: 0.35, 95% CI: 0.03 to 0.52	⊕⊕○○ Low^d^, ^e^	*I* ^2^ = 33.43	[29] [44]
*Nimodipine*	*SCAG, GBS‐intellectual. Duration: 26‐52 weeks (no follow‐up)*	481 (*two* RCTs)	*Subcortical vascular dementia MID*	**Cohen's *d***: 0.09, 95% CI: −0.00 to 0.27	⊕⊕○○ Low^a^, ^d^	*I* ^2^ = 0	[17] [18]^a^
*rTMS*	*MoCA Duration: 4 weeks. Follow‐up after treatment: none*	*57 (two RCTs)*	*Post‐stroke cognitive impairment*	**Cohen's *d***: 0.43, 95% CI: −0.38 to 1.23 **Mean difference**: + 1.23, 95% CI: −1.16 to 3.62	⊕○○○ Very low^c^, ^e^, ^h^	*I* ^2^ = 55.40	[122] [148]
*tDCS*	*ADAS‐Cog, MoCA* *Duration: 2 weeks. Follow‐up after treatment: none*	*97 (two RCTs)*	*Vascular dementia, POST‐stroke dementia*	**Cohen's *d***: 1.20, 95% CI: −0.70 to 3.11	⊕○○○ Very low^c^, ^d^, ^e^, ^g^	*I* ^2^ = 92.43%	[159] [124]
*Cognitive training*	*MoCA and MMSE* *Duration: 4 to 20 weeks. Follow‐up after treatment: 6 months (1)* ^[136]^	188 (four RCTs)	*Subcortical vascular MCI. Subcortical vascular cognitive impairment no dementia. Post‐stroke cognitive impairment*	**Cohen's *d***: 0.71, 95% CI: 0.10 to 1.32	⊕○○○ Very low^a^, ^c^, ^d^, ^e^, ^f^	*I* ^2^ = 75.34	[134] [135] [139] [168]
*Physical exercise*	*ADAS‐Cog, MoCA* *Duration: 24 weeks to 6 months. Follow‐up after treatment: 1 to 6 months*	*118 (two RCTs)*	*Subcortical vascular MCI Post‐stroke cognitive impairment*	***At end of treatment***: Cohen's *d*: 0.60, 95% CI: 0.23 to 0.97 ***At end of follow‐up***: Cohen's *d*: 0.50, 95% CI −0.17 to 1.16	⊕○○○ Very low^a^, ^d^, ^e^, ^h^ ⊕○○○ Very low^d^, ^e^, ^f^, ^i^	*I* ^2^ = 0 *I* ^2^ = 67.59	[137] [133]

*Note*: The specific outcomes meta‐analyzed as well as VCI population, sample size, effect size metrics, quality of evidence according to GRADE framework, statistical heterogeneity metric, and single study codes are included.

Abbreviations: CI, confidence interval; ADAS, Alzheimer's Disease Assessment Scale; ADAS‐CoG, Alzheimer's Disease Assessment Scale Cognition; GBS, Göttfries‐Brane‐Steen Scale; GCE, Global Cognitive Efficiency; MMSE, Mini‐Mental State Examination; MoCA, Montreal Cognitive Assessment; rTMS, repetitive transcranial magnetic stimulation; SKT, Short Cognitive Performance Test; tCDS, transcranial direct current stimulation; VaDAS, Vascular Dementia Assessment Scale; VaDAS‐CoG, Vascular Dementia Assessment Scale cognitive subscale; VCI, vascular cognitive impairment.

**GRADE Working Group grades of evidence**:

**High certainty**: We are very confident that the true effect lies close to that of the estimate of the effect.

**Moderate certainty**: We are moderately confident in the effect estimate: The true effect is likely to be close to the estimate of the effect, but there is a possibility that it is substantially different.

**Low certainty**: Our confidence in the effect estimate is limited: The true effect may be substantially different from the estimate of the effect.

**Very low certainty**: We have very little confidence in the effect estimate: The true effect is likely to be substantially different from the estimate of effect.

^a^ Low generalizability due to inclusion of different VCI populations (downgraded once for indirectness).

^b^ Downgraded twice due to imprecision: The 95% CI includes a result that would not be considered clinically important and a result that would be considered important.

^c^ Two or more items at uncertain (or greater) risk of bias (downgraded once).

^d^ Different outcome measures employed for the same outcome (downgraded once).

^e^ Inconsistency in point estimates (downgraded once).

^f^ Some imprecision (wide 95% CI, downgraded once).

^g^ Imprecision (wide 95% CI, downgraded twice).

^h^ Downgraded once due to imprecision: The 95% CI includes a result that would not be considered clinically important and a result that would be considered important.

^i^ Low generalizability due to inclusion of different VCI populations and due to different follow‐up timespans (despite comparable treatment durations). Downgraded twice for indirectness.

**TABLE 1B:** Summary of treatment efficacy on *functional outcomes*.

Intervention	Functional outcomes Treatment duration	*No*. participants (*n* of studies)	VCI population	Efficacy metric	Quality of evidence (GRADE)	Statistical heterogeneity	Studies
*Ginkgo biloba*	*GBS‐ADL subscale and ADL‐IS (2 RCT)* *Duration: 22 to 24 weeks (no follow‐up)*	*252 (2 RCTs)*	*Vascular dementia (2) ^[23] [43]^*	**Cohen's *d***: 0.50, 95% CI: 0.25 to 0.75	⊕⊕○○Low^c^, ^d^	*I* ^2^ = 0	[23] [43]
*Rivastigmine*	*ADCS‐ADL, IADL Duration: 24 to 26 weeks (no follow‐up)*	*799 (3 RCTs)*	*Subcortical vascular dementia, Vascular dementia, post‐stroke MCI*	**Cohen's *d***: 0.04, 95% CI: −0.10 to 0.18	⊕○○○ Very Low ^a^, ^b^, ^c^	*I* ^2^ = 0	[97] [93] [82]
*Donepezil* *(all doses)*	*ADFACS, DAD, ADL* *Duration: 24 to 26 weeks* *(no follow‐up)*	*2456 (4 RCTs)*	*Vascular dementia*	**Cohen's *d***: 0.11, 95% CI: 0.04 to 0.19	⊕⊕⊕○ Moderate^c^	*I* ^2^ = 0	[31] [33] [78] [86]
*Memantine*	*NOSGER Duration: 28 weeks (no follow‐up)*	*525 (2 RCTs)*	*Vascular Dementia (2 RCT)*	**Cohen's *d***: −0.08, 95% CI: −0.25 to 0.09	⊕⊕⊕⊕ High	*I* ^2^ = 0	[21] [32]
*Nimodipine*	*IADL, NOSGERDuration: 26 to 52 weeks (no follow‐up)*	481 (2 RCTs)	*Subcortical vascular dementia (2) – MID (1)*	**Cohen's *d***: 0.07, 95% CI: −0.13 to 0.27	⊕⊕○○Low^1^, ^3^	*I* ^2^ = 0	[17] [18]

Abbreviations: CI, confidence interval; ADCS‐ADL, Alzheimer's Disease Cooperative Study–Activities of Daily Living; ADFACS, Alzheimer's Disease Functional Assessment and Change Scale; ADL‐IS, Activities of Daily Living International Scale; DAD, Disability Assessment for Dementia; GBS, Göttfries‐Brane‐Steen Scale; IADL, Instrumental Activities of Daily Living; NOSGER, Nurses Observation Scale for Geriatric Patients.

**GRADE Working Group grades of evidence**:

**High certainty**: We are very confident that the true effect lies close to that of the estimate of the effect.

**Moderate certainty**: We are moderately confident in the effect estimate: The true effect is likely to be close to the estimate of the effect, but there is a possibility that it is substantially different.

**Low certainty**: Our confidence in the effect estimate is limited: The true effect may be substantially different from the estimate of the effect.

**Very low certainty**: We have very little confidence in the effect estimate: The true effect is likely to be substantially different from the estimate of effect.

^a^ Low generalizability due to inclusion of different VCI populations (downgraded once for indirectness).

^b^ Two or more items at uncertain (or greater) risk of bias (downgraded once).

^c^ Different measures employed for the same outcome (downgraded once).

^d^ Some inconsistency in point estimates (downgraded once).

## 4 DISCUSSION

This systematic review and meta‐analysis sought to establish whether any previously studied treatments for VCI possessed unrecognized benefits and to elucidate why no treatment is currently licensed for VCI. We addressed these aims by dividing them into four key areas: variability of interventions and interventional studies in VCI, potentially effective strategies that may have been overlooked, possible reasons for the current absence of licensed treatments, and methods to enhance the probability of identifying effective treatments.

### 4.1 Previously investigated strategies and study heterogeneity

Our systematic review revealed substantial heterogeneity in both interventions and study designs. The primary source of this heterogeneity was the definition of VCI itself, as shown in the observed variability of inclusion criteria and nosological labels. This inherent heterogeneity has likely impeded the development of standardized diagnostic criteria, resulting in a multitude of different criteria currently in use.^8^, ^9^ Moreover, this broad definition may have diverted the focus from precise subtype definition, which is crucial when evaluating potential treatments for a disease‐specific biological effect.

Another important source of heterogeneity was the diverse range of investigated treatments, closely linked to the diverse rationales behind their selection. From our review, four distinct approaches emerged: strategies selected due to efficacy in other dementias (primarily AD), those selected due to the postulated effect on potential disease‐related mechanisms (e.g., cerebral perfusion enhancement, neuroprotection), those aimed at compensatory or symptomatic effects, and strategies with unclear rationale. In our analysis, only studies employing the first three approaches met the criteria for quantitative synthesis and demonstrated the most promising efficacy.

Finally, heterogeneity was contributed by a wide array of outcomes and comparators. Outcomes were predominantly cognitive, occasionally functional or instrumental, and rarely patient‐centered. Comparators were frequently active treatment strategies, despite the absence of any currently approved for VCI.

### 4.2 Reappraisal of tested interventions and potentially promising avenues

Our meta‐analysis focused on all treatments sufficiently investigated as monotherapy and against placebo, assessed within specific outcome classes of interest, when data were available. Among cognitive outcomes, we prioritized global cognitive efficiency, as neuropsychological tests were often unavailable or highly heterogeneous in both the domains assessed and the specific test used.

Several interventions demonstrated positive effects on cognitive function, as well as on functional outcomes, albeit to a significantly lesser extent (with low or neutral effect sizes, except for *Ginkgo biloba* extracts).

Some of these interventions, such as cerebrolysin,^10^ acetylcholinesterase inhibitors,^11^ memantine,^12^ and nimodipine,^13^ have been the subject of previous meta‐analyses. As no additional studies have been published for these interventions, our results are entirely consistent with previous meta‐analyses, and all these interventions were associated with small statistical effects and negligible clinically meaningful effects, we will not discuss them further.

We highlight instead the efficacy of treatments whose effectiveness may have been only partially recognized. Among these, *Ginkgo biloba* extracts demonstrated improvement of global cognitive performance and functional status in VCI patients. We acknowledge that the three studies included in this meta‐analysis exhibited heterogeneity, that the estimate for cognitive efficiency was moderately imprecise, and that their clinical significance may be questionable. Nevertheless, we consider these findings promising and conclude that *Ginkgo biloba* extracts merit further investigation, particularly given their oral formulation, relative affordability, and favorable safety profile.

Physical exercise showed a moderate positive effect on global cognition, though the effect was not maintained at follow‐up, and data on functional outcomes were not sufficient for meta‐analysis.

Cognitive training also showed a moderate positive effect on global cognition but showed no effect on functional outcomes. Notably, a considerable proportion of the reviewed studies did not consider functional outcomes altogether. Lack of functional outcome assessment (or efficacy) represents a critical limitation, as functional outcomes should be considered the primary measure of effectiveness in rehabilitative interventions. Indeed, improvements in cognitive outcomes might be anticipated regardless of disease‐specific effects, due to the well‐established temporary enhancement of cognitive performance observed with closely spaced neuropsychological assessments. To address this, future trials in cognitive rehabilitation should consistently include functional outcome measures.

Acupuncture warrants special consideration as, in other dedicated reviews, it was reported to have consistently good results.^14^, ^15^ However, very few studies utilized sham acupuncture as a comparator, and acupuncture was often investigated in combination with other treatments. Therefore, this technique merits further evaluation in rigorous clinical trials.[Fig alz70840-fig-0005]

Studies only rarely reported data on patient‐centered outcomes. We must acknowledge also the sparse reporting of instrumental outcomes. This widespread lack may reflect the absence of shared biomarkers or – given that at least imaging biomarkers have been codified^16^ – a certain reluctance to employ them as secondary outcomes. We recognize that many trials were conducted before the formulation of the standards for reporting vascular changes on neuroimaging (STRIVE) criteria,^16^, ^17^ and the widespread use of magnetic resonance, but surrogate biomarkers are a crucial component in evaluating candidate treatment efficacy and should be implemented consistently in future studies.

### 4.3 Critical appraisal for the absence of approved VCI treatment

Although the qualitative review of evidence highlighted numerous “positive” trials and some meta‐analyses yielded promising findings, no treatment for VCI has yet received regulatory approval. We believe this is attributable to several factors. First, the previously discussed population heterogeneity reduced statistical power and increased indirectness, thereby limiting the generalizability of the findings. Second, the evaluation of combined treatment strategies, comparison with multiple active treatments, and use of many different outcomes significantly reduced interpretability and comparability across studies. Third, studies exhibited varying methodological quality and very different statistical power, resulting in highly imprecise effect estimates. Ultimately, methodological limitations were so substantial that reasonable confidence in the evidence of treatment efficacy (or lack thereof) could only be achieved for a limited number of interventions. Moreover, in most cases where statistical efficacy was demonstrated, the clinical significance remained doubtful.

It is important to note that most interventions included in this review – and in previous meta‐analyses, such as those on acetylcholinesterase inhibitors – have been tested in specific VCI subtypes only in a minority of studies. Data were insufficient to allow analyses by diagnostic label, except for nimodipine. This limitation, critical for assessing clinical effectiveness, is even more pronounced for non‐meta‐analyzed interventions, where data were scarcer and participants were often recruited under the broad label of “VCI.” For these reasons, all the treatments reviewed could still be considered for adequately powered and rigorously designed future trials, provided there is a clear rationale for their use in the selected VCI subtype.

### 4.4 Future directions

**FIGURE 5 alz70840-fig-0005:**
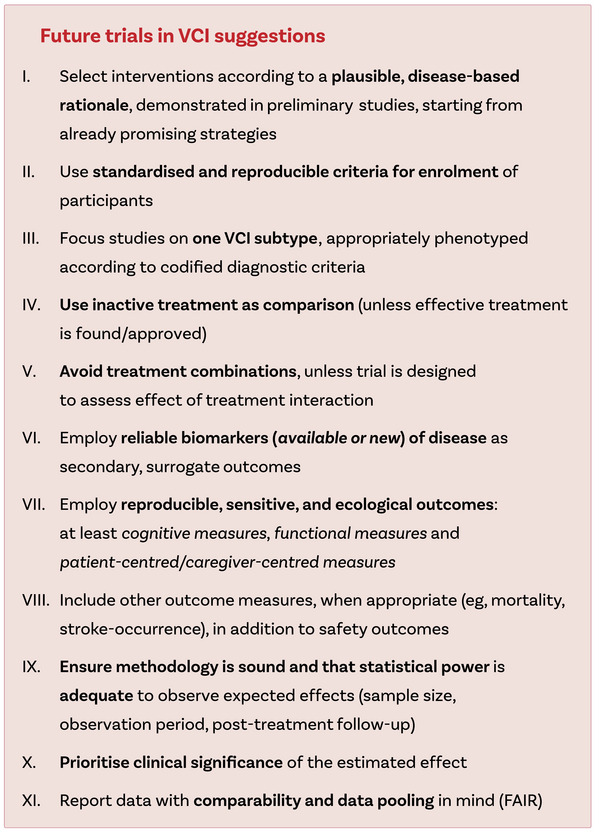
Suggestions for future trials in vascular cognitive impairment.

We suggest some key features that future studies should implement to maximize the likelihood of identifying effective treatments (Figure 5), some of which are already outlined in the framework for clinical trials in cerebral small vessel disease (FINESSE) and a previous review.^18^, ^19^ Priority should be given to therapeutic strategies based on pathophysiology and adequately tested in preliminary studies for safety and plausible effects. These interventions should be assessed as monotherapy against inactive treatment, and studies should focus on a single, standardized VCI label to reduce indirectness and improve study power.

Reproducible and sensitive cognitive outcomes are essential. While global cognitive efficiency metrics may be efficient, they should always include comprehensive neuropsychological testing, particularly batteries sensitive to the most frequent cognitive alterations in VCI.^20^

Ecologically relevant outcomes (i.e., functional and patient‐centered), surrogate outcomes for specific diseases, and etiology‐relevant hard measures (e.g., mortality, stroke) should be included whenever possible. Studies should be designed to achieve adequate statistical power and to prioritize clinical significance. Finally, data reporting should adhere to Findability, Accessibility, Interoperability, and Reuse (FAIR) principles,^21^ ensuring that trial data are ultimately quantitatively summarizable in future meta‐analyses.

Our review is in line with a previous one on the same topic.^19^ We report similar heterogeneity in outcomes and interventions and concur on the actions needed to address this issue in future trials. Notably, our review builds upon this previous one and completes our earlier work^5^ by summarizing efficacy results and by pooling data quantitatively. This approach provides objective evidence of efficacy, together with an estimation of evidence strength, which may inform the re‐evaluation of some interventions in future studies.

### 4.5 Limitations

Our study has several limitations. The literature search was restricted to three medical databases, and thus some studies may not have been retrieved by our original search; to mitigate this, we reviewed studies included within other reviews. We only included studies published in English, which may have limited the inclusion of pertinent research in other languages, particularly Chinese. When quantitatively pooling the results, specific methodological choices were necessary to account for heterogeneity. These choices were, however, aimed at minimizing the risk of false positive findings. We clearly outlined these decisions in the text, contextualized them within best meta‐analysis practices, and tested their robustness through sensitivity analyses.

Finally, we excluded studies involving participants with mixed dementia. While this may have reduced the volume of included data, we consider it essential to minimize confounding from neurodegenerative disease and potential treatment effects on these components (especially considering that many interventions were specifically borrowed from the Alzheimer's disease field). Nonetheless, we believe that any intervention shown to be effective in “pure” VCI would also benefit the vascular component of mixed cases.

## 5 CONCLUSIONS

Our review demonstrates that previous studies frequently suffered from methodological flaws and significant heterogeneity, hindering the identification of effective treatments and contributing to the growing skepticism regarding the possibility of finding successful VCI therapies. Despite some promising interventions that may warrant further investigation, it remains unclear whether effective treatments for VCI are within reach or far away, as the current approach to research has not provided sufficient evidence to either support or refute the efficacy of most tested treatments. Future research should prioritize rigorous, standardized studies focusing on specific VCI subtypes, employing appropriate outcomes, and adhering to FAIR data‐sharing principles to accelerate the development of effective therapies.

## CONFLICT OF INTEREST STATEMENT

Federico Masserini: none. Claudia Gendarini: none. Giacomo Baso: none. Emilia Salvadori: none. Leonardo Pantoni: none. Leonardo Pantoni has received personal consulting fees from Medtronic, PIAM, and Amicus. Federico Masserini was partially supported by Associazione Italiana Ricerca Alzheimer (AIRALZH), after being awarded a competitive grant on an unrelated project (AGYR2024). Author disclosures are available in the supporting information.

## CONSENT STATEMENT

This study was a meta‐analysis based on previously published research. As no individual participant data were available to the authors, no human participants were directly involved in this study.

## Supporting information



Supporting Information

Supporting Information

Supporting Information

Supporting Information

Supporting Information

Supporting Information

Supporting Information
