# Supplementary material for: Therapeutic strategies in vascular cognitive impairment: A systematic review and meta‐analysis
Source: Alzheimers Dement. 2025 Nov 6;21(11):e70840. doi: 10.1002/alz.70840 (PMC12591988; doi:10.1002/alz.70840)
Supplement: Supplementary file 3 — Supporting Information [file ALZ-21-e70840-s003.docx]

***Supplementary File 3***: **Summary of included study characteristics, including publication year, study aim, design, VCI subtype and cognitive degree severity, inclusion criteria in summary, interventions employed, and outcomes used**.

| **Study ID** | **Covidence ID** | **Title** | **Corresponding Author** | **Country** | **Year** | **Study aim** | **Design** | **VCI label** | **VCI severity** | **Inclusion criteria** | **Other dementia** | **Study size  (n of patients)** | **Intervention 1** | **Intervention 2** | **Intervention 3** | **Comparator** | **Outcomes collected*** |
| --- | --- | --- | --- | --- | --- | --- | --- | --- | --- | --- | --- | --- | --- | --- | --- | --- | --- |
| 1 | 58 | *Shenmayizhi Formula Combined with Ginkgo Extract Tablets for the Treatment of Vascular Dementia: A Randomized, Double-Blind, Controlled Trial* | H. Zhang | China | 2020 | To evaluate whether a combination of SMYZF with Ginkgo extract tablets improves mild-to-moderate VaD. | RCT | Vascular | Dementia | DSM + NINDS-AIREN, CDR 1-2, 10 ≤ MMSE ≤ 26, HIS ≥ 7, NIHSS 5-15 | No | 196 | Ginkgo Biloba and Shenmayizhi formula (SMYZF) |  |  | Placebo and Ginkgo Biloba | MMSE Endothelin-1 levels Plasmatic nitric oxide  Plasmatic von Willebrand factor  Plasmatic neuron-specific enolase  BDNF CM-SS |
| 2 | 59 | *A clinical trial investigating the effect of trimetazidine combined with oxiracetam in patients with vascular dementia* | C. Zhang | China | 2016 | To evaluate the efficacy of trimetazidine combined with oxiracetam in patient with VaD. | RCT | Vascular | Dementia | DSM-IV | No | 82 | Trimtazidine and cerebrolysin |  |  | Oxiracetam | MMSE CDR Barthel Index |
| 3 | 260 | *Efficacy and Safety of the Association of Nimodipine and Choline Alphoscerate in the Treatment of Cognitive Impairment in Patients with Cerebral Small Vessel Disease. The CONIVaD Trial* | E. Salvadori | Italy | 2021 | To investigate the feasibility, efficacy, and safety of a combined treatment with choline alphoscerate and nimodipine in patients with small vessel disease and mild-to-moderate cognitive impairment. | RCT | Subcortical vascular; small vessel disease | MCI; dementia | Clinical and/or radiological data (0.5 ≤ CDR ≤ 2, Fazekas 2-3 on MRI or van Swieten > 2 on CT) | No | 62 | Nimodipine and and alfa-Glycerylphosphorylcholine/Choline alfoscerate |  |  | Placebo and Nimodipine | MoCA IADL ADL DAD Stroke Adapted-Sickness Impact Profile 30 Neuropsycological tests (2): attentive and executive functions |
| 4 | 270 | *Nicergoline in senile dementia of Alzheimer type and multi-infarct dementia: A double blind, placebo controlled, clinical and EEG/ERP mapping study* | B. Saletu | Austria | 1995 | To investigate in a double-blind, placebo-controlled study, the therapeutic efficacy of nicergoline, in patients with SDAT and MID. | RCT | Multi-infarct | Dementia | DSM-III and DSM-IIIR; MMSE [13-25]; HIS (> 7) | Yes | 56 | Nicergoline |  |  | Placebo | ADCG-CGIC MMSE SCAP HAM-D EEG parameters P300 |
| 5 | 275 | *EEG mapping and psychopharmacological studies with denbufylline in SDAT and MID* | B. Saletu | Multi-national (Europe) | 1992 | To assess therapeutic efficacy of denbufylline on cognitive impairment and EEG in patients diagnosed with SDAT or MID. | RCT | Multi-infarct | Dementia | HIS (> 7); MMSE (10-25) | Yes | 51 | Denbufylline |  |  | Placebo | ADCG-CGIC MMSE SCAG Neuropsychological tests (2): executive functions, memory EEG parameters |
| 6 | 350 | *Effects of Butyphthalide Combined with Idebenone on Inflammatory Cytokines and Vascular Endothelial Functions of Patients with Vascular Dementia* | F. Qi | China | 2020 | To determinate the clinical effect of butyphthalide combined with idebenone in the treatment of vascular dementia (VD) and the influence on inflammatory cytokines and vascular endothelial function. | RCT | Vascular | Dementia | DSM-IV | No | 88 | Idebenone and piracetam and butylphtalide |  |  | Piracetam Idebenone | MMSE CDR ADL Serum IL-7 TNF-alfa SOD activity |
| 7 | 420 | *Ateroid in the clinical treatment of multi-infarct dementia* | M. Passeri | Italy | 1989 | To evaluate the efficacy of sulfomucopolysaccharides in patients with a diagnosis of MID. | RCT | Multi-infarct | Dementia | HIS | No | 30 | Sulfomucopolysaccharides |  |  | Placebo | Stuard Hospital geriatric scale GBS NPI Neuropsychological tests: memory |
| 8 | 424 | *Vascular dementia italian sulodexide study (VA.D.I.S.S.) clinical and biological results* | L. Parnetti | Italy | 1997 | To evaluate the biological effects on some hemostasis factors of antithrombotic-hemorheological treatments on patients with vascular dementia of sulodexide and pentoxifylline. | RCT | Vascular | Dementia | NINDS-AIREN; MMSE [11-22] | No | 93 | Sulodexide |  |  | Pentoxifylline | Plasmatic fibrinogen GBS MMSE Factor VII-Ag |
| 9 | 427 | *Posatirelin in the treatment of vascular dementia: A double-blind multicentre study vs placebo* | L. Parnetti | Italy | 1996 | To evaluate the usefulness of posatirelin (L-pyro-2-aminoadipyl-L-leucyl-L-prolinamide) in vascular dementia. | RCT | Vascular | Dementia | NINDS-AIREN; HIS (>6) | No | 136 | Posatirelin |  |  | Placebo | Neuropsychological tests (2): memory, executive functions GBS |
| 10 | 486 | *Treatment with Huperzine A Improves Cognition in Vascular Dementia Patients* | Z. Xu | China | 2012 | To test the efficacy and safety of Huperzine A in treatment of mild to moderate vascular dementia. | RCT | Vascular | Dementia | NINDS-AIREN, 12≤ MMSE ≤ 24, radiological evidence of CVD, HIS ≥ 7 | No | 78 | Huperzine A |  |  | Vitamin C | MMSE CDR ADL |
| 11 | 493 | *An open-label pilot study of acetylcholinesterase inhibitors to promote functional recovery in elderly cognitively impaired stroke patients* | E. M. Whyte | United States | 2008 | To investigate feasibility of using acetylcholinesterase inhibitors in older patients with acute post-stroke cognitive impairment and examine their effects on functional recovery. | Non-randomised experimental study | Post-stroke; acute/subacute stroke | MCI; dementia | Clinical and/or radiological data | No | 40 | Galantamine |  |  | Donepezil | FIM Apathy Evaluation Scale Neuropsychological tests (2): executive functions, memory |
| 12 | 511 | *Effects of Chinese medicine for promoting blood circulation and removing blood stasis in treating patients with mild to moderate vascular dementia: A randomized, double-blind and parallel-controlled trial* | M. Wei | China | 2012 | To evaluate the effects of Chinese medicine for promoting blood circulation and removing blood stasis in patients with to moderate VaD. | RCT | Vascular | Dementia | DSM-IV + NINDS-AIREN; HIS > 4, MMSE 11-26 | No | 48 | Danshen (Radix Salviae Miltiorrhiae) and Sanqi (Radix Notoginseng) |  |  | Placebo | ADAS-Cog MMSE ADL |
| 13 | 532 | *Efficacy of butylphthalide soft capsules in patients with vascular dementia and the relevant antioxidative mechanism* | S. Wang | China | 2019 | To observe the efficacy of butylphthalide soft capsules in patients with vascular dementia. | RCT | Vascular | Dementia | Dementia (Chinese medical association criteria) within 3 months after the occurrence of cerebrovascular disease | No | 124 | Butylphtalide |  |  | Donepezil | MMSE CDR ADL Chinese version SF-36 SOD activity Malonaldehyde levels Transcranial US |
| 14 | 543 | *Comparison of integrated traditional Chinese and western medicine therapy on vascular cognitive impairment with no dementia* | L.P. Wang | China | 2015 | To investigate the clinical effect of western medicine therapy assisted by Ginkgo biloba tablets in patients with vascular cognitive impairment with no dementia. | RCT | Vascular | MCI | Clinical and/or radiological data; HIS ≥ 7 | No | 80 | Ginkgo Biloba and ASA |  |  | ASA | MoCA Transcranial US |
| 15 | 646 | *Jian Nao Ning for treatment of memory impairment in patients with mild or moderate multi-infarct dementia* | J. Tian | China | 2002 | To assess effectiveness of Jian Nao Ning compared to duxil in patients with multi-infarct dementia. | RCT | Multi-infarct | Dementia | DSM-IV; NINDS-AIREN (probable VaD); radiological evidence; CDR [1-2], MMSE [11-24] | No | 40 | Jin Nao Ning |  |  | Duxil | Neuropsychological tests: memory |
| 16 | 682 | *Efficacy and safety of nimodipine in subcortical vascular dementia: A subgroup analysis of the Scandinavian Multi-Infarct Dementia Trial* | L. Pantoni | Multi-national (Europe) | 2000 | Post-hoc subgroup analysis of the Scandinavian Multi-Infarct Dementia Trial that evaluated the efficacy and safety of oral nimodipine administered for 6 months in 259 patients. | RCT | Subcortical vascular | Dementia | DSM-III-R; CT assessment of subcortical ischemic vascular disease of the brain | No | 92 | Nimodipine |  |  | Placebo | GBS MMSE Cerebrovascular events Neuropsychological tests (3): attentive functions, memory, language IADL |
| 17 | 685 | *Efficacy and safety of nimodipine in subcortical vascular dementia: A randomized placebo-controlled trial* | L. Pantoni | Italy | 2005 | To study the efficacy and safety of the calcium antagonist nimodipine in subcortical VaD. | RCT | Subcortical vascular | Dementia | ICD-10; MMSE [12-24]; CT (van Swieten scale) | No | 242 | Nimodipine |  |  | Placebo | SCAG Neuropsychological tests (2): language, executive functions MMSE NOSGER Neuropsychological tests: praxis/dexterity HAM-D |
| 18 | 687 | *The Scandinavian Multi-Infarct Dementia Trial: A double-blind, placebo-controlled trial on nimodipine in multi-infarct dementia* | L. Pantoni | Multi-national (Europe) | 2000 | To evaluate the safety and efficacy of nimodipine administered for as long as 26 weeks in improving cognition or slowing cognitive deterioration in patients defined as having multi-infarct dementia (DSM-III-R criteria). | RCT | Multi-infarct | Dementia | DSM-III and DSM-IIIR; MMSE (> 12) | No | 259 | Nimodipine |  |  | Placebo | GBS MMSE Cerebrovascular events Neuropsychological tests (2): attentive functions, memory, language IADL |
| 19 | 692 | *Effect of Yin-Xing-Tong-Zhi Tablets on Improving Vascular Cognitive Impairment No Dementia* | W. Pan | China | 2018 | To observe the effect of the Chinese herbal extracts, Yin-Xing-Tong-Zhi, in tablet form, on improving vascular cognitive impairment no dementia. | RCT | Vascular | MCI | Clinical and/or radiological data; MMSE ≥ 20 | No | 68 | Yin-Xing-Tong-Zhi |  |  | Placebo | ADAS-Cog CIBIC-plus MMSE Serum IL-7 IL-9 TNF-alfa |
| 20 | 695 | *MLC601 in vascular dementia: An efficacy and safety pilot study* | H. Pakdaman | Iran | 2017 | To investigate the efficacy and safety of MLC601 in the treatment of VaD. | RCT | Vascular | Dementia | DSM-V; MMSE ≤ 10, ADAS-CoG > 12, radiological evidence of CVD on CT/MRI | No | 81 | MLC601 (NeuroAid) |  |  | Placebo | MMSE ADAS-Cog |
| 21 | 700 | *Efficacy and safety of memantine in patients with mild to moderate vascular dementia: A randomized, placebo-controlled trial (MMM 300)* | JM. Orgogozo | Europe | 2002 | To examine the efficacy and tolerability of memantine, an uncompetitive N-methyl-D-aspartate antagonist, in the treatment of mild to moderate vascular dementia. | RCT | Vascular | Dementia | NINDS-AIREN (probable VaD) | No | 321 | Memantine |  |  | Placebo | ADAS-Cog CIBIC-plus MMSE GBS ADCG-CGIC NOSGER |
| 22 | 717 | *Correction of Subdementia Cognitive Impairments in Patients with Cerebral Ischemia* | M. M. Odinak | Russia | 2014 | To assess the potential of using Vitrum Memory (Gingko Biloba extract) in the treatment of patients with grade I and II cerebral ischemia. | RCT | Vascular | MCI | Clinical and/or radiological data, MMSE ≤ 23; HIS ≥ 4 | No | 45 | Ginkgo Biloba |  |  | Best medical treatment | MMSE MoCA Neuropsychological tests (4): language, memory, executive functions, CDT |
| 23 | 739 | *Efficacy and tolerability of Ginkgo biloba extract EGb 761 by type of dementia: Analyses of a randomised controlled trial* | O. Napryeyenko | Multi-national (Europe) | 2009 | To confirm the efficacy of EGb761 in patients suffering from dementia with neuropsychiatric symptoms and to investigate the efficacy of EGb 761 in the two diagnostic subgroups. | RCT | Vascular | Dementia | NINDS-AIREN; 9 ≤ SKT ≤ 23; 14 ≤ MMSE ≤ 25 | Yes | 181 | Ginkgo Biloba |  |  | Placebo | SKT NPI GBS Neuropsychological tests (2): language, CDT |
| 24 | 749 | *A crossover trial of bromocriptine in the treatment of vascular dementia* | S. E. Nadeau | United States | 1988 | To evaluate the efficacy of bromocripitine in patients with VaD. | RCT | Vascular | Dementia | Other: not specified | No | 7 | Bromocriptine |  |  | Placebo | Neuropsychological tests (2): attentive and executive functions WAIS-R IQ |
| 25 | 762 | *Randomized, double-blind, placebo controlled, multicentre study of idebenone in patients suffering from multi-infarct dementia* | V. Marigliano | Italy | 1992 | To evaluate the effect of idebenone on MID patients. | RCT | Multi-infarct | Dementia | DSM-III-R; NINDS-AIREN; HIS | No | 108 | Idebenone |  |  | Placebo | GBS Neuropsychological tests (3): attentive functions, language, memory |
| 26 | 770 | *A 12-month, randomized, placebo-controlled trial of propentofylline (HWA 285) in patients with dementia according to DSM III-R* | J. Marcusson | Multi-national (Europe) | 1997 | To assess efficacy of propentofylline in Alzheimer's disease and Vascular dementia. | RCT | Vascular | Dementia | DSM-III-R; HIS; MMSE (15-25) | Yes | 90 | Propentofylline |  |  | Placebo | GBS ADCG-CGIC SKT |
| 27 | 813 | *Network topology and machine learning analyses reveal microstructural white matter changes underlying Chinese medicine Dengzhan Shengmai treatment on patients with vascular cognitive impairment* | H. Lu | China | 2020 | To evaluate the 6-month treatment effects of Dengzhan Shengmai capsules and to explore the underlying neural mechanisms with graph theory-based analysis and machine learning method based on diffusion tensor imaging data. | RCT | Vascular | MCI | Clinical and/or radiological data | No | 100 | Dengzhan Shengmai |  |  | Placebo | MMSE ADAS-Cog Neuropsychological tests (5): memory, attentive and executive functions, language, CDT |
| 28 | 858 | *Effects of fluoxetine on brain-derived neurotrophic factor serum concentration and cognition in patients with vascular dementia* | X. Liu | China | 2014 | To investigate the effects of fluoxetine, a selective serotonin reuptake inhibitor, on cognition and serum BDNF levels in patients with vascular dementia. | RCT | Vascular | Dementia | DSM-IV; HIS > 7 | No | 50 | Fluoxetine |  |  | Placebo | MMSE Neuropsychological tests (2): CDT, language BDNF |
| 29 | 916 | *The efficacy of cerebrolysin in patients with vascular dementia: Results of a Chinese multicentre, randomised, double-blind, placebo-controlled trial* | S. Xiao | China | 1999 | To evaluate the efficacy and safety of cerebrolysin in patients with mild to moderately severe vascular dementia. | RCT | Vascular | Dementia | DSM-IV; MMSE (15-25); HIS (> 7), GDS (3-5) | No | 148 | Cerebrolysin |  |  | Placebo | MMSE ADCG-CGIC Neuropsychological tests: executive functions SCAG ADL HAM-D NAI |
| 30 | 931 | *Memantine in severe dementia: results of the 9M-Best Study (Benefit and efficacy in severely demented patients during treatment with memantine)* | B. Winblad | Multi-national (Europe) | 1999 | To assess clinical efficacy and safety of memantine in moderately severe to severe primary dementia. | RCT | Vascular | Dementia | DSM-III and DSM-IIIR; MMSE (< 10); HIS > 5 | Yes | 87 | Memantine |  |  | Placebo | ADCG-CGIC BGP nursing rating |
| 31 | 946 | *Donepezil in vascular dementia: A randomized, placebo-controlled study* | D. Wilkinson | International | 2003 | To evaluate the efficacy and tolerability of donepezil in patients with vascular dementia. | RCT | Vascular | Dementia | NINDS-AIREN (possible and probable VaD); MMSE [10-26] | No | 616 | Donepezil | Donepezil |  | Placebo | ADAS-Cog CIBIC-plus MMSE CDR-sb ADFACS |
| 32 | 950 | *A double-blind, placebo-controlled multicentre study of memantine in mild to moderate vascular dementia (MMM500)* | G. Wilcock | United Kingdom | 2002 | To investigate the safety and efficacy of memantine in mild to moderate vascular dementia. | RCT | Vascular | Dementia | DSM-III and DSM-IIIR; NINDS-AIREN; HIS (≥ 4); MMSE (10-22) | No | 548 | Memantine |  |  | Placebo | ADAS-Cog ADCG-CGIC NOSGER GBS MMSE |
| 33 | 966 | *Randomized, placebo-controlled, clinical trial of donepezil in vascular dementia: Differential effects by hippocampal size* | G. C. Roman | United States | 2010 | To assess the efficacy and safety of donepezil in patients with vascular dementia. | RCT | Vascular | Dementia | NINDS-AIREN | No | 974 | Donepezil |  |  | Placebo | VaDAS-Cog MMSE CIBIC-plus Neuropsychological tests: executive functions CDR-sb DAD |
| 34 | 982 | *Effect of Pushen capsule for treating vascular mild cognitive impairment: a pilot observational study* | S. Li | China | 2019 | To evaluate the efficacy of Pushen capsule, a compound containing several TCM components, for treating vascular mild cognitive impairment. | RCT | Vascular | MCI | DSM-V; MMSE > 24, MoCA > 22 | No | 62 | Pushen capsule |  |  | Ginkgo Biloba | MoCA MMSE Blood lipids Hematocrit and/or hemorrheological measures |
| 35 | 1004 | *Methylphenidate and galantamine in patients with vascular cognitive impairment-the proof-of-principle study STREAM-VCI* | J. F. Leijenaar | Multi-national (Europe) | 2020 | To evaluate whether a single dose of a monoaminergic drug (methylphenidate) improves executive functioning and whether a single dose of a cholinergic drug (galantamine) improves memory in VCI patients. | RCT | Vascular | MCI; dementia | Clinical and/or radiological data (MMSE ≥ 16, CDR 0.5-1; CVD evidence on MRI) | No | 30 | Methylphenydate | Galantamine |  | Placebo | Neuropsychological tests (3): executive functions, memory, other EEG |
| 36 | 1094 | *Proof of efficacy of the ginkgo biloba special extract EGb 761 in outpatients suffering from mild to moderate primary degenerative dementia of the Alzheimer type or multi-infarct dementia* | S. Kanowski | Germany | 1996 | To investigate efficacy of the ginkgo biloba special extract EGb761 in outpatients with presenile and senile primary degenerative dementia of the Alzheimer type and multi-infarct dementia according to DSM-Ill-R. | RCT | Multi-infarct | Dementia | Other: DSM-III-R + IS (Rosen) | Yes | 31 | Ginkgo Biloba |  |  | Placebo | ADCG-CGIC SKT |
| 37 | 1096 | *Efficacy of xantinolnicotinate in patients with dementia* | S. Kanowski | Multi-national (Europe) | 1990 | To evaluate the efficacy and safety of xantinolnicotinate in patients with MID and SDAT. | RCT | Multi-infarct | Dementia | DSM-III and DSM-IIIR; HIS ≥ 7 | Yes | 143 | Xantinolnicotinate |  |  | Placebo | ADCG-CGIC or CGI(C) SCAG BGP nursing rating Neuropsychological tests: executive functions |
| 38 | 1119 | *Intervention effect of folic acid and vitamin B12 on vascular cognitive impairment complicated with hyperhomocysteinemia* | B. Jiang | China | 2014 | To explore the effects of folic acid and VitB12 on the homocysteine level and cognitive function in patients with vascular cognitive impairment‚ dementia complicated with hyperhomocysteinemia. | RCT | Vascular | MCI | HIS | No | 120 | Folic acid Vitamin B12 |  |  | Best medical treatment | MoCA Evoked potentials Plasmatic homocysteine Serum folic acid Serum vitamin B12 levels |
| 39 | 1121 | *The effects of DL-3-n-butylphthalide in patients with vascular cognitive impairment without dementia caused by subcortical ischemic small vessel disease: A multicentre, randomized, double-blind, placebo-controlled trial* | J. Jia | China | 2016 | To evaluate the therapeutic efficacy of NBP for patients with subcortical vascular cognitive impairment without dementia. | RCT | Subcortical vascular | MCI | Clinical and/or radiological data; DSM-IV | No | 281 | Butylphtalide |  |  | Placebo | ADAS-Cog CIBIC-plus MMSE CDR CDR -sb ADL Chinese version NPI |
| 40 | 1123 | *Efficacy and safety of the compound Chinese medicine SaiLuoTong in vascular dementia: A randomized clinical trial* | J. Jia | China | 2018 | To evaluate efficacy and safety of the compound Chinese medicine SaiLuoTong in vascular dementia. | RCT | Vascular | Dementia | Other: DSM-IV, NINDS-AIREN, HIS | No | 340 | SaiLuo Tong | SaiLuo Tong |  | Placebo Galantamine | VaDAS-Cog ADCG-CGIC MMSE ADL CDR Neuropsychological tests: visuospatial domain NPI |
| 41 | 1136 | *Efficacy of Choto-san on vascular dementia and the protective effect of the hooks and stems of Uncaria sinensis on glutamate-induced neuronal death* | T. Itoh | Japan | 1999 | To evaluate the efficacy of Choto-san on patients with vascular dementia. | RCT | Vascular | Dementia | DSM-III and DSM-IIIR; time criterion (more than 1 months since latest stroke) | No | 139 | Choto-san |  |  | Placebo | CGI HDS |
| 42 | 1140 | *The effects of donepezil, galantamine, rivastigmine and memantine on mini-mental state examination and mean flow velocity in patients with vascular dementia: A double- blinded randomized clinical trial* | F. Iranmanesh | Iran | 2020 | To evaluate the effects of donepezil, memantine, rivastigmine and galantamine on mean flow velocity and MMSE of patients with vascular dementia in a three-month follow-up period. | Non-randomised experimental study | Vascular | Dementia | DSM-V | No | 44 | Memantine | Rivastigmine | Galantamine | Donepezil | MMSE Transcranial US |
| 43 | 1160 | *Efficacy and tolerability of a once daily formulation of Ginkgo biloba extract EGb 761 in Alzheimer's Disease and vascular dementia: Results from a randomised controlled trial* | R. Ihl | Multi-national (Europe) | 2012 | To evaluate efficacy and tolerability of a once daily formulation of Ginkgo biloba extract EGb 761 in AD and VaD. | RCT | Vascular | Dementia | NINDS-AIREN | Yes | 71 | Ginkgo Biloba |  |  | Placebo | SKT NPI ADCG-CGIC ADL DEMQOL-Proxy Neuropsychological tests: language |
| 44 | 1199 | *Persistence of the effects of Cerebrolysin on cognition and qEEG slowing in vascular dementia patients: Results of a 3-month extension study* | D. F. Muresanu | Multi-national (Europe) | 2008 | To assess the maintenance of the effects of Cerebrolysin, a peptidergic compound with neurotrophic activity, on cognitive performance and qEEG activity (12-week, open-label extension of a 4-week, randomised, placebo-controlled pilot study). | Non-randomised experimental study | Vascular | Dementia | NINDS-AIREN; 9 ≤ MMSE ≤ 26 | No | 41 | Cerebrolysin | Cerebrolysin |  | Placebo | ADAS-Cog EEG |
| 45 | 1203 | *A neurotropic approach to the treatment of multi-infarct dementia using L-glycerylphosphorylchlorine* | A. Muratorio | Italy | 1992 | To evaluate the effects of 3 months of treatment with L-a-GPC, compared with those of cytidine diphosphocholine (CDP-choline), on the cognitive and behavioral deficits of patients with mild to moderate MID and to observe how long these effects could be maintained during a 3-month period without therapy. | RCT | Multi-infarct | Dementia | HIS; MMSE (12-23); HAM-D (<22) | No | 112 | Alfa-Glycerylphosphorylcholine/Choline alfoscerate |  |  | Citicoline (CDP-Choline) | SCAG DS Neuropsychological tests (3): memory, language, visuospatial domain RDRS |
| 46 | 1225 | *Different responses to rivastigmine in subcortical vascular dementia and multi-infarct dementia* | R. Moretti | Italy | 2008 | To determine the effects of rivastigmine on cognitive function, global daily living performance, and behavioral disorders in VaD patients versus an active control (nimodipine), stratifying patients according to the type of VaD, subcortical vascular dementia (sVAD), and multi-infarct dementia (MID). | RCT | Subcortical vascular | Dementia | NINDS-AIREN; DSM-IV; MMSE (≥12) | No | 100 | Rivastigmine and  ASA |  |  | Nimodipine and ASA | BEHAVE-AD CDR Neuropsychological tests (2): CDT, language MMSE Tinetti scale CIRS |
| 46bis | 2241 | *Different responses to rivastigmine in subcortical vascular dementia and multi-infarct dementia (Duplicate for Data Extraction)* | R. Moretti | Italy | 2008 | To determine the effects of rivastigmine on cognitive function, global daily living performance, and behavioral disorders in VaD patients versus an active control (nimodipine), stratifying patients according to the type of VaD, subcortical vascular dementia and multi-infarct dementia. | RCT | Multi-infarct | Dementia | NINDS-AIREN | No | 100 | Rivastigmine and ASA |  |  |  | BEHAVE-AD CDR Neuropsychological tests: CDT MMSE Neuropsychological tests: language Tinetti scale CIRS |
| 47 | 1227 | *Rivastigmine superior to aspirin plus nimodipine in subcortical vascular dementia: an open, 16-month, comparative study* | R. Moretti | Italy | 2004 | To investigate the efficacy of rivastigmine compared to cardioaspirin in treating cognitive impairment in subcortical vascular dementia. | Non-randomised experimental study | Subcortical vascular | Dementia | NINDS-AIREN; MMSE (≥ 12), DSM-IV | No | 64 | Rivastigmine |  |  | ASA Nimodipine | MMSE Neuropsychological tests (3): attentive functions, CDT, language BEHAVE-AD Barthel Index IADL |
| 48 | 1231 | *Rivastigmine in subcortical vascular dementia: A randomized, controlled, open 12-month study in 208 patients* | R. Moretti | Italy | 2003 | To determine whether rivastigmine has any effects on the typical symptoms of subcortical VaD. | RCT | Subcortical vascular | Dementia | DSM-IV; NINDS-AIREN; MMSE (≥ 14) | No | 208 | Rivastigmine |  |  | ASA | MMSE Neuropsychological tests (2): CDT, language BEHAVE-AD GDS CIRS |
| 49 | 1233 | *An open-label pilot study comparing rivastigmine and low-dose aspirin for the treatment of symptoms specific to patients with subcortical vascular dementia* | R. Moretti | Italy | 2002 | To investigate the efficacy of rivastigmine in improving the symptoms of VaD. | Non-randomised experimental study | Subcortical vascular | Dementia | Other: NINDS-AIREN + DSM-IV + CT | No | 16 | Rivastigmine |  |  | Best medical treatment | MMSE Neuropsychological tests (2): CDT, language IADL NPI CDR RSS |
| 50 | 1236 | *Olanzapine as a possible treatment for anxiety due to vascular dementia: An open study* | R. Moretti | Italy | 2004 | To assess the efficacy of olanzapine compared to bromazepam on cognition and in treating behavioral symptoms of dementia in vascular dementia patients. | RCT | Vascular | Dementia | DSM-IV; NINDS-AIREN; NPI (≥ 3), MMSE (≥ 14) | No | 94 | Olanzapine |  |  | Bromazepam | MMSE BEHAVE-AD NPI CIRS RSS |
| 51 | 1237 | *Olanzapine as a possible treatment of behavioral symptoms in vascular dementia: Risks of cerebrovascular events - A controlled, open-label study* | R. Moretti | Italy | 2005 | To assess efficacy and safety of olanzapine in controlling BPSD due to vascular dementia in an open-label study. | Non-randomised experimental study | Vascular | Dementia | NINDS-AIREN; DSM-IV; MMSE (≥ 14); BPSD for more than 4 weeks; NPI (> 30) | No | 356 | Olanzapine |  |  | Promazine, haloperidol | CDR NPI Barthel Index Tinetti scale HIS Caregiver burden inventory |
| 52 | 1238 | *Cholinesterase inhibition as a possible therapy for delirium in vascular dementia: A controlled, open 24-month study of 246 patients* | R. Moretti | Italy | 2004 | To determine whether rivastigmine has any effect on delirium in vascular dementia (VaD). | Non-randomised experimental study | Subcortical vascular; multi-infarct | Dementia | NINDS-AIREN; DSM-IV; MMSE (> 14) | No | 246 | Rivastigmine |  |  | Best medical treatment | BEHAVE-AD CDR CIRS Agitation/delirium/psychosis (aggregated) |
| 53 | 1255 | *Naftidrofuryl in the treatment of vascular dementia* | H. J. Moller | Multi-national (Europe) | 2001 | To examine the efficacy and safety of oral naftidrofuryl in the treatment of vascular and mixed type dementia. | RCT | Vascular | Dementia | NINDS-AIREN; ADAS-COG (≥ 18); SCAG (≥ 35); HIS (≥ 4); MRI featuring cerebrovascular disease. | Yes | 403 | Naftidrofuryl | Naftidrofuryl |  | Placebo | ADAS-Cog SCAG NOSGER ADCG-CGIC |
| 54 | 1284 | *Propentofylline improves regional cerebral glucose metabolism and neuropsychologic performance in vascular dementia* | R. Mielke | Germany | 1996 | To evaluate the effects of the adenosine uptake blocker propentofylline (HWA 285) on regional cerebral glucose metabolism (rCMRG1). | RCT | Vascular | Dementia | DSM-III and DSM-IIIR | No | 30 | Propentofylline |  |  | Placebo | FDG-PET SUVr MMSE Neuropsychological tests (3): memory, attentive functions, visuospatial domain |
| 55 | 1290 | *Randomized clinical trial of daily aspirin therapy in multi-infarct dementia. A pilot study* | J. Stirling Meyer | United States | 1989 | To investigate effect of aspirin on cognitive performance in patients with multi-infarct dementia. | RCT | Multi-infarct | Dementia | HIS; CT/MRI evidence of cerebrovascular disease | No | 70 | ASA |  |  | Best medical treatment | CCSE rCBF |
| 56 | 1364 | *A multicenter randomized double-blind study on the efficacy and safety of nicergoline in patients with multi-infarct dementia* | W. M. Herrmann | Multi-national (Europe) | 1997 | To evaluate the efficacy and safety of nicergoline in patients with MID. | RCT | Multi-infarct | Dementia | DSM-III and DSM-IIIR | 1 | 136 | Nicergoline |  |  | Placebo | SCAG MMSE Neuropsychological tests (2): memory, executive functions BL-A behavior scale |
| 57 | 1366 | *Moving from the question of efficacy to the question of therapeutic relevance: an exploratory reanalysis of a controlled clinical study of 130 inpatients with dementia syndrome taking piracetam* | W. M. Herrmann | Multi-national (Europe) | 1992 | To survey the extent of drug-related improvement and response rates when assessed at different levels and to investigate the comparability of efficacy in subgroups suffering from either senile dementia of the Alzheimer type or multi-infarct dementia. | RCT | Multi-infarct | Dementia | HIS | Yes | 65 | Piracetam |  |  | Placebo | SCAG BGP nursing rating SKT |
| 58 | 1379 | *Comparative randomized study of cerebral blood flow after long-term administration of pentoxifylline and co-dergocrine mesylate in patients with chronic cerebrovascular disease* | A. Hartmann | Multi-national (Europe) | 1985 | To evaluate the effects of long term administration of pentoxifylline and co-dergocrine mesylate. | RCT | Vascular | Dementia | Clinical and/or radiological data | No | 90 | Pentoxifylline | Co-dergocrine mesylate |  |  | rCBF |
| 59 | 1422 | *Oxpentifylline in dementia: a controlled study* | K. Ghose | UK | 1987 | To investigate the effect of oxpentifylline in primary degenerative dementia and multi-infarct dementia patients. | RCT | Multi-infarct | Dementia | HIS; MMSE (10-24) | Yes | 11 | Pentoxifylline |  |  | Placebo | MMSE SCAG |
| 60 | 1449 | *Study of the P300 and cerebral maps in subjects with multi-infarct dementia treated with cytidine* | V. Gallai | Italy | 1991 | To evaluate the efficacy of cytidine in subjects with reduced mental capacity by means of the P300, an event-related potential, and topographical brain maps. | Non-randomised controlled trial | Multi-infarct | Dementia | HIS; MMSE (15-25) | No | 20 | Citidina |  |  | Placebo | Evoked potentials EEG parameters |
| 61 | 1468 | *Multicenter clinical comparison of the effects of choline alfoscerate and cytidine diphosphocholine in the treatment of multi-infarct dementia* | L. Frattola | Italy | 1991 | To compare the effectiveness and tolerability of alfa-GPC with those of CFP-Choline on cognitive and bahavior deficits of mild to moderate degree in patients with MID. | RCT | Multi-infarct | Dementia | HIS | No | 126 | Alfa-Glycerylphosphorylcholine/Choline alfoscerate |  |  | Citicoline (CDP-Choline) | SCAG Parkside behaviour rating scale Neuropsychological tests (2): memory, language |
| 62 | 1503 | *Therapeutic efficacy of pyritinol in patients with senile dementia of the Alzheimer type (SDAT) and multi-infarct dementia (MID)* | P. K. Fischhof | Multi-national (Europe) | 1992 | To investigate the efficacy of pyritinol in the treatment of senile dementia. | RCT | Multi-infarct | Dementia | HIS; DSM-III R; MMSE (10-23) | Yes | 57 | B6 (piritinol) |  |  | Placebo | ADCG-CGIC or CGI(C) SKT SCAG |
| 63 | 1504 | *Therapeutic efficacy of vincamine in dementia* | P. K. Fischhof | Multi-national (Europe) | 1996 | To investigate whether vincamine is effective in the treatment of elderly patients suffering from mild to moderate dementia of the Alzheimer type or multi-infarct dementia. | RCT | Multi-infarct | Dementia | HIS | Yes | 77 | Vincamine |  |  | Placebo | SCAG SKT |
| 64 | 1538 | *Galantamine in the treatment of cognitive decline in patients with vascular dementia or Alzheimer's disease with cerebrovascular disease* | T. Erkinjuntti | International | 2003 | To evaluate the efficacy and safety profiles of galantamine in patients with VaD or AD with cerebrovascular disease over the longer term (> 6 months). | Non-randomised experimental study | Vascular | Dementia | NINDS-AIREN | Yes | 195 | Galantamine |  |  | Placebo and Galantamine | ADAS-Cog |
| 65 | 1540 | *Efficacy of galantamine in probable vascular dementia and Alzheimer's disease combined with cerebrovascular disease: A randomised trial* | T. Erkinjunnti | International | 2002 | To evaluate the effects of galantamine in patients with a diagnosis of probable vascular dementia. | RCT | Vascular | Dementia | NINDS-AIREN | Yes | 188 | Galantamine |  |  | Placebo | ADAS-Cog CIBIC-plus |
| 66 | 1604 | *Clinical trials in dementia with propentofylline* | B. Kittner | International | 1997 | To investigate whether propentofylline is efficacious in Alzheimer's disease and vascular dementia, and whether the suggested neuroprotective mechanisms translate into disease-modifying effects in the studied patient population. | RCT | Vascular | Dementia | DSM-III and DSM-IIIR; HIS; MMSE [15-25] | Yes | 359 | Propentofylline |  |  | Placebo | GBS CCSE SKT MMSE NAI |
| 67 | 1656 | *Donepezil in patients with subcortical vascular cognitive impairment: a randomised double-blind trial in CADASIL* | M. Dichigans | International | 2008 | To determine whether the cholinesterase inhibitor donepezil improves cognition in patients with CADASIL. | RCT | Subcortical vascular (CADASIL) | MCI; dementia | Mutation in NOTCH3 or electron-dense granular osmiophilic material in blood vessels; MMSE (10-27) | No | 168 | Donepezil |  |  | Placebo | VaDAS-Cog ADAS-Cog MMSE Neuropsychological tests (2): executive functions, CDT DAD CDR-sb |
| 68 | 1661 | *A multicentre trial to evaluate the efficacy and tolerability of alpha-glycerylphosphorylcholine versus cytosine diphosphocholine in patients with vascular dementia* | R. Di Perri | Italy | 1991 | To compare the efficacy and the tolerability of 1 g/day a-glycerylphosphorylcholine with 1g/day cytosinediphosphocholine, both given intramuscularly for 90 days in 120 patients with mild to moderate vascular dementia. | RCT | Multi-infarct | Dementia | DSM-III and DSM-IIIR; HIS | No | 120 | Alfa-Glycerylphosphorylcholine/Choline alfoscerate |  |  | Citicoline (CDP-Choline) | Parkside behaviour rating scale SCAG Neuropsychological tests (2): language, memory HAM-D |
| 69 | 1685 | *Comparison of sulfomucopolysaccharides and cytidine diphosphocholine in the treatment of multi-infarct dementia. A randomized double-blind test* | D. Cucinotta | Italy | 1988 | To compare sulfomucopolysaccharides and cytidine diphosphocholine in the treatment of multi-infarct dementia. | RCT | Multi-infarct | Dementia | HIS | No | 30 | Sulfomucopolysaccharides |  |  | Citicoline | SCAG NPI Neuropsychological tests (2): attentive functions, memory |
| 70 | 1687 | *Multicentre clinical placebo-controlled study with buflomedil in the treatment of mild dementia of vascular origin* | D. Cucinotta | Italy | 1989 | To evaluate Buflomedil in the treatment of mild dementia of vascular origin. | RCT | Vascular | Dementia | HIS | No | 73 | Buflomedil then buflomedil then buflomedil | Buflomedil then buflomedil then no treatment | No treatment then buflomedil then buflomedil | No treatment then Buflomedil then no treatment | Stuard Hospital geriatric  Symptoms rating scale CGI NPI Neuropsychological tests: attentive functions Global clinical assessment |
| 71 | 1696 | *Clinical and biochemical responses to therapy in Alzheimer's disease and multi-infarct dementia* | G. L. Corona | Italy | 1989 | Memory performance, central monoaminergic function and sympathetic nerve activity were studied in patients with dementia of the Alzheimer type or with multi-infarct dementia before and after 4 weeks with single or combined drug therapy (choline-piracetam). | RCT | Multi-infarct | Dementia | HIS | No | 16 | Citicoline (CDP-Choline) or Piracetam |  |  | Citicoline (CDP-Choline) Piracetam | Neuropsychological tests: memory Central monoamine levels Overall monoamine levels Sympathetic activity |
| 72 | 1708 | *Long-term citicoline (cytidine diphosphate choline) use in patients with vascular dementia: Neuroimaging and neuropsychological outcomes* | R. A. Cohen | United States | 2003 | To determine whether daily citicoline treatment improves neurocognitive and neuroimaging outcome over 12 months among patients diagnosed with vascular dementia. | RCT | Vascular | Dementia | NINDS-AIREN | No | 39 | Citicoline (CDP-Choline) |  |  | Placebo | White matter radiological burden MMSE Neuropsychological tests (5): attentive and executive functions, memory, visuospatial domain, language, praxis/dexterity |
| 73 | 1759 | *Effects of butylphthalide combined with naofukang on cognitive function and the expression of serum neurotrophic factor in patients with vascular dementia* | Y. Chen | China | 2020 | To determine the effect of butylphthalide combined with Naofukang on cognitive function and serum neurotrophic factor level in patients with vascular dementia. | RCT | Vascular | Dementia | DSM-V; HIS, 12 ≤ MMSE ≤ 23 | No | 172 | Butylphtalide Piracetam |  |  | Piracetam | MMSE CDR ADL Barthel Index BDNF |
| 74 | 1832 | *The effects of bromvincamine and vincamine on regional cerebral blood flow and mental functions in patients with multi-infarct dementia* | S. Hagstadius | Multi-national (Europe) | 1984 | To evaluate the effects of vincamine arid bromvincamine (BV 26-723) on mental functions. | RCT | Multi-infarct | Dementia | HIS | No | 10 | Brovincamine | Vincamine |  | Placebo | rCBF Psychiatric symptoms quantitative rating scale Neuropsychological tests (3): memory, language, executive functions |
| 75 | 1847 | *Cerebrolysin in vascular dementia: Improvement of clinical outcome in a randomized, double-blind, placebo-controlled multicenter trial* | A. B. Guekht | International | 2011 | To evaluate improvement of Clinical Outcome in patients with Vascular Dementia after use of cerebrolysin. | RCT | Vascular | Dementia | NINDS-AIREN; MMSE 10-24, HIS > 4 | No | 242 | Cerebrolysin |  |  | Placebo | ADAS-Cog+ CIBIC-plus MMSE ADL Neuropsychological tests (2): CDT, attentive functions |
| 76 | 1852 | *ARTEMIDA Trial (A Randomized Trial of Efficacy, 12 Months International Double-Blind Actovegin): A Randomized Controlled Trial to Assess the Efficacy of Actovegin in Poststroke Cognitive Impairment* | A. Guekht | Russia, Belarus, and Kazakhstan | 2017 | To assess whether Actovegin confers cognitive benefit in patients who have had an ischemic stroke. | RCT | Post-stroke | MCI | Clinical and/or radiological data; Acute supratentorial ischemic stroke (NIHSS 3-18); MoCA <= 25 | No | 503 | Actovegin |  |  | Placebo | ADAS-Cog+ MoCA NIHSS BDI Barthel Index EuroQoL EQ-5D questionnaire Dementia diagnosis (ICD) |
| 77 | 1856 | *Naftidrofuryl in the treatment of mild senile dementia. A double-blind study* | W. M. Grossmann | Multi-national (Europe) | 1990 | To examine the effectiveness and tolerance of naftidrofuryl in patients with senile dementia, with reference to the aetiology of the disease. | RCT | Multi-infarct | Dementia | HIS | Yes | 27 | Naftidrofuryl |  |  | Placebo | SKT Neuropsychological tests (3): visuospatial domain, attentive functions, memory Depressiveness Scale AGP-Score EEG parameters |
| 78 | 1942 | *Efficacy and tolerability of donepezil in vascular dementia: Positive results of a 24-week, multicenter, international, randomized, placebo-controlled clinical trial* | S. Black | International | 2003 | To evaluate the efficacy and safety of donepezil for relieving symptoms of dementia in VaD. | RCT | Vascular | Dementia | NINDS-AIREN | No | 603 | Donepezil | Donepezil |  | Placebo | CIBIC-plus ADAS-Cog CDR-sb MMSE IADL ADL |
| 79 | 1945 | *Pentoxifylline in cerebrovascular dementia* | R.S. Black | United States | 1992 | To test the effect of pentoxifylline on the course of vascular dementia. | RCT | Vascular | Dementia | Hachinski Ischemic Score | No | 64 | Pentoxifylline |  |  | Placebo | ADAS ADAS-Cog ADAS-CoG-NoMemory ADAS-NoCog |
| 80 | 1968 | *Idebenone in the treatment of multi-infarct dementia: A randomised, double-blind, placebo controlled multicentre trial* | B. Bergamasco | Italy | 1992 | A controlled double blind randomised open multicentre study was carried out on 104 patients with multi-infarct dementia from a mild to a moderate degree to assess efficacy of idebenone in improving cognitive function. | RCT | Multi-infarct | Dementia | HIS (6-10); MMSE (> 10); SCAG (40-90) | No | 104 | Idebenone |  |  | Placebo | GBS SCAG DSA Neuropsychological tests: memory, language |
| 81 | 1993 | *European Pentoxifylline Multi-Infarct Dementia Study* | A.J. Bayer | Multi-national (Europe) | 1996 | A double blind, placebo-controlled, parallel-group, multicentre study to evaluate the efficacy of pentoxifylline in multi-infarct dementia. | RCT | Multi-infarct | Dementia | DSM-III and DSM-IIIR | No | 289 | Pentoxifylline |  |  | Placebo | GBS MMSE SCAG Cerebrovascular events Neuropsychological tests (2): memory, language |
| 82 | 2021 | *Efficacy, safety and tolerability of rivastigmine capsules in patients with probable vascular dementia: the VantagE study* | C. Ballard | International | 2008 | To evaluate the efficacy, safety and tolerability of rivastigmine capsules in patients diagnosed with probable vascular dementia. | RCT | Vascular | Dementia | NINDS-AIREN | No | 710 | Rivastigmine |  |  | Placebo | VaDAS ADCG-CGIC ADAS-Cog MMSE Neuropsychiatric inventory (NPI) ADL Geriatric Depression Scale |
| 83 | 2050 | *Galantamine treatment of vascular dementia: A randomized trial* | A.P. Auchus | International | 2007 | To evaluate efficacy and safety of galantamine for patients with vascular dementia. | RCT | Vascular | Dementia | NINDS-AIREN; MMSE (10-26); ADAS-CoG (≥ 12) | No | 788 | Galantamine |  |  | Placebo | ADAS-Cog ADL CIBIC-plus NPI Neuropsychological tests: executive functions |
| 84 | 2059 | *Effects of intravenous high dose c-dergocrine mesylate ('Hydergine'¬Æ) in elderly patients with severe multi-infarct dementia: A double-blind, placebo-controlled trial* | A. Arrigo | Italy | 1989 | To evaluate if a daily intravenous infusion of 3 mg co-dergocrine mesylate over 14 days would improve severely deteriorated elderly patients and shorten the latent period (3 months) which is observed when the drug is given orally. | RCT | Multi-infarct | Dementia | HIS | No | 40 | Co-dergocrine mesylate |  |  | Placebo | SCAG NPI |
| 85 | 2090 | *Efficacy and safety of Ginkgo biloba standardized extract in the treatment of vascular cognitive impairment: A randomized, double-blind, placebo-controlled clinical trial* | V. Demarin | Europe | 2017 | The aim of this randomized, double-blind, placebo-controlled trial was to determine the efficacy and safety of Ginkgo biloba extract in patients diagnosed with vascular cognitive impairment (VCI). | RCT | Vascular | MCI; dementia | Clinical and/or radiological data; MMSE ≤ 20 | No | 90 | Ginkgo Biloba | Ginkgo Biloba |  | Placebo | MDRS ADCG-CGIC Sandoz Clinical Assessment Geriatric scale (SCAG) MMSE Transcranial US |
| 86 | 2238 | *Tianzhi granule improves cognition and BPSD of vascular dementia: a randomized controlled trial* | J. Shi | China | 2020 | To assess the effect of Tianzhi granules by a randomised clinical trials. | RCT | Vascular | Dementia | NINDS-AIREN;14 ≤ MMSE ≤ 26, HIS > 7 | No | 543 | MLC601 (NeuroAid) | Donepezil |  | Placebo | VaDAS CIBIC-plus MMSE NPI Neuropsychological tests: executive functions ADL |
| 87 | 2252 | *Effects of candesartan on cerebral microvascular function in mild cognitive impairment: Results of two clinical trials* | I. Hajjar | United States | 2023 | Investigate the effects of renin–angiotensin system modulation on cerebrovascular reactivity in individuals with mild cognitive impairment (MCI) due to underlying vascular or AD etiologies. | RCT | Vascular | MCI | MCI with executive dysfunction (defined with NPS testing) , hypertension, MoCA ≤ 26 | No | 55 | Candesartan |  |  | Lisinopril | Neuropsychological  tests (4): memory,  attention, executive  functions, language  fMRI |
| 88 | 2291 | *Naoxin’an capsules protect brain function and structure in patients with vascular cognitive impairment* | J. Zhang | China | 2023 | To investigate the protectiveness of Naoxin'an capsule (NXA) on human brain structure and function in patients with VCI. | RCT | Vascular | MCI | DSM-V or NINDS-AIREN | No | 45 | Naoxin’an |  |  | *Ginkgo Biloba extracts* | MMSE  ADAS-CoG  Neuropsychological tests (4): memory, visuospatial functions, executive functions, language  fMRI |
| 89 | 2381 | *The Effect of Guilingji Capsules on Vascular Mild Cognitive Impairment: A Randomized, Double-Blind, Controlled Trial* | L. Ma | China | 2022 | To evaluate the clinical efficacy and safety of Guilingji capsules (GLJC) for the treatment of vascular mild cognitive impairment (VaMCI). | RCT | Vascular | MCI | DSM-V, MoCA > 22 or MMSE > 24, CDR ≤ 0.5, HIS ≥ 7, temporal relationship with vascular event and cognitive impairment onset | No | 37 | Guilingji |  |  | *Ginkgo Biloba extracts* | MoCA  MMSE  ADAS-CoG  FMA  Acetylcholine  Acetylcholine esterase high-sensitivity C-reactive protein |
| 90 | 2407 | *Augmentation therapy with tandospirone citrate in vascular depression patients with mild cognitive impairment: A prospective randomized clinical trial* | N. Liu, Y. Xiao, H. Chen | China | 2022 | To assess the efficacy of tandospirone citrate in VaDep cases with mild cognitive impairment (VaDep-MCI)  as well as the role of plasma monoamine neurotransmitters during the treatment. | RCT | Vascular | MCI | Clinical and/or radiological data; MoCA < 26, CDR 0.5 | No | 55 | Tandospirone  Escitalopram |  |  | Escitalopram | Neuropsychological tests (4): attention, executive functions, language, memory  CDT  Hamilton Depression Rating Scale  Hamilton Anxiety Rating Scale |
| 91 | 2534 | *Modified Suanzaoren decoction in treating post-stroke cognitive impairment with comorbid insomnia symptoms: A clinical trial* | M. Zhu | China | 2023 | To investigate the effectiveness of Modified Suanzaoren decoction (M-SZRD) in treating post-stroke cognitive impairment with comorbid insomnia symptoms. | RCT | Post-stroke | MCI  Dementia | DSM-V | No | 38 | Modified Suanzaoren decoction (M-SZRD) | Promazine |  | Zolpidem | MoCA  PSQI  NIHSS  Modified Barthel Index  Hamilton Depression Rating Scale  Hamilton Anxiety Rating Scale  Plasma ACTH |
| 92 | 1794 | *Neural correlates of donepezil-induced cognitive improvement in patients with right hemisphere stroke: a pilot study* | W. H. Chang | Korea | 2011 | To valuate the effect of donepezil on reorganization of the cognitive neural network in patients with post-stroke cognitive impairment using functional MRI. | RCT | Post-stroke | MCI | Other: First-ever stroke + right lesion + MMSE 10-26 | No | 14 | Donepezil |  |  | Placebo | MMSE Neuropsychological tests: memory functional MRI |
| 93 | 2552 | *A randomized controlled trial of rivastigmine in patients with cognitive impairment no dementia because of cerebrovascular disease* | E. K. Tan | Singapore | 2010 | We investigated the safety and efficacy of rivastigmine in cognition, particularly executive function in patients with CIND because of cerebrovascular disease. | RCT | Acute/subacute stroke  Post-stroke | MCI | MRI assessment for vascular burden; Cognitive impairment no dementia (DSM-IV) | No | 25 | Rivastigmine |  |  | Placebo | CDT  Neuropsychological tests: executive functions  ADAS-CoG  ADL  GDS  NPI |
| 94 | 2562 | *NEURoaid II (MLC901) in cognitively Impaired not demenTEd patientS (NEURITES): a pilot double blind, placebo-controlled randomized trial* | C. L. H. Chen | Multinationa (Singapore, Philippines, Vietnam) | 2020 | Evaluate the comparative change in executive function from baseline (BL) to 24 weeks in VCIND patients after treatment with either MLC901 or placebo. | RCT | Post-stroke | MCI | Cognitive impairment no dementia (DSM IV) due to cerebrovascular disease (onset within 12 month from index stroke) | No | 57 | MLC901(NEURoaid II) |  |  | Placebo | ADAS-CoG  MoCA  CDT  Neuropsychological tests (3): attention, executive functions, language  ADL |
| 95 | 2565 | *Effectiveness and safety of citicoline in mild vascular cognitive impairment: the IDEALE study* | A. M. Cotroneo | Italy | 2013 | To assess the effectiveness and safety of oral citicoline in elderly people with mild vascular cognitive impairment. | Non-randomised experimental study | Vascular | MCI | Clinical and/or radiological data; MMSE ≥ 21 | No | 265 | Citicoline |  |  | Best medical treatment | MMSE  ADL  IADL  Depression (aggregated) |
| 96 | 2584 | *Effects of Dengzhan Shengmai Capsule combined with butylphthalide soft capsule on oxidative stress indexes and serum Hcy and CRP levels in patients with vascular dementia* | W. Sun | China | 2020 | To investigate the effect of Dengzhan Shengmai capsule combined with butylphthalide soft capsule on oxidative stress indexes and serum homocysteine (Hcy) and C-reactive protein (CRP) levels in patients with vascular dementia (VD). | RCT | Vascular | Dementia | Clinical and/or radiological data: vascular involvement at imaging; dementia (12 ≤ MMSE ≤ 24, loss of autonomy) | No | 41 | Butylphtalide | Dengzhan Shengmai | Butylphtalide  Dengzhan Shengmai |  | MMSE  MoCA  SDSVD  Hematocrit  serum CRP  pSOD  MDA |
| 97 | 2587 | *Rivastigmine in Chinese patients with subcortical vascular dementia* | V. Mok | China | 2007 | We explored the efficacy and tolerability of rivastigmine among Chinese patients with subcortical vascular dementia. | RCT | Subcortical vascular | Dementia | NINDS-AIREN | No | 20 | Rivastigmine |  |  | Placebo | MMSE  Neuropsychological tests: executive functions  NPI  IADL  CDR-sb |
| 98 | 2611 | *Fluoxetine May Enhance VEGF, BDNF and Cognition in Patients with Vascular Cognitive Impairment No Dementia: an Open-Label Randomized Clinical Study* | X. Liu | China | 2021 | To evaluate fluoxetine in cognition and serum BDNF and VEGF in patients with vascular cognitive impairment no dementia. | RCT | Vascular | MCI | HIS > 7, cognitive impairment no dementia (DSM-IV) | No | 25 | Fluoxetine |  |  | Best medical treatment | ADAS-CoG  CDT  MMSE  Neuropsychological tests (2): language, attention  BDNF  VEGF |
| 99 | 2612 | *Clinical study on effect of Xianjong Capsule in treating senile vascular dementia* | Y. Zhao | China | 2002 | To assess the efficacy of Xianlong Capsule (XLC) in treating senile vascular dementia. | RCT | Post-stroke | Dementia | Clinical and/or radiological data | No | 24 | Xialong capsule |  |  | Hydergine | MMSE  HDS  GUIDANCE  ADL |
| 100 | 34 | *Electroacupuncture on the head points for improving Gnosia in patients with vascular dementia* | Z. Ling | China | 2009 | To investigate the clinical effects of electroacupuncture (EA) on the head points for improving gnosia in patients with vascular dementia (VD). | RCT | Vascular | Dementia | NINDS-AIREN; HIS ≥ 7; CDR 1 or 2 | No | 90 | Nimodipine and ‘yi qi tiao xue, fu ben pei yuan’ (acupuncture) | Electro-acupuncture |  | Nimodipine | MMSE P300 |
| 101 | 530 | *Efficacy and safety assessment of acupuncture and nimodipine to treat mild cognitive impairment after cerebral infarction: A randomized controlled trial* | S. Wang | China | 2016 | To investigate the efficacy and safety of acupuncture and nimodipine to treat post-cerebral infarction MCI. | RCT | Post-stroke; acute/subacute stroke | MCI | Clinical and/or radiological data, MoCA < 26; recent episode of ischemic stroke | No | 126 | Nimodipine and acupuncture | Acupuncture |  | Nimodipine | MoCA |
| 102 | 555 | *A promising approach to the treatment of multi-infarct dementia* | M. Walzl | Austria | 2000 | To determine whether reduction of fibrinogen and lipid fractions produces an improvement of the rheological pattern as well as improved clinical symptoms in MID. | RCT | Multi-infarct | Dementia | DMS-III; NINCDS-ADRDA; HIS; MRI assessment; fibrinogen ≥ 500 mg/dL | No | 216 | Pentoxifylline  & Heparin-induced extracorporeal LDL/fibrinogen precipitation (HELP) |  |  | Pentoxifylline | MS MMSE ADL Hematocrit and/or hemorrheological measures Blood lipids |
| 103 | 867 | *Neuroprotection against vascular dementia after acupuncture combined with donepezil hydrochloride: P300 event related potential* | Q. Liu | China | 2016 | To investigate the effect of acupuncture combined with donepezil hydrochloride in treating vascular dementia after stroke on P300 event related potential and MMSE. | RCT | Vascular | Dementia | NINDS-AIREN, MMSE ≤ 23; HIS ≥ 7 | No | 272 | Donepezil and acupuncture |  |  | Donepezil | MMSE Evoked potentials |
| 104 | 2328 | *Effect of acupuncture on cerebral blood flow, serum S100β and ALP in the patients with post-stroke mild cognitive impairment in the convalescence stage* | W. Shi | China | 2022 | To explore the clinical therapeutic effect and the impacts of acupuncture on cerebral blood flow and serum S100β and alkaline phosphatase (ALP) in the patients with post-stroke mild cognitive impairment in convalescence stage | RCT | Post-stroke | MCI | Clinical and/or radiological data | No | 32 | Acupunture  Oxiracetam |  |  | Oxiracetam | MoCA  MMSE  modified Barthel Index  Ecocolordoppler ICA  S100β  Alkaline phophsatase |
| 105 | 1775 | *Clinical research on comprehensive treatment of senile vascular dementia* | L. Chen | China | 2011 | To observe the curative effect of combined Chinese medicine, acupuncture and rehabilitation therapy on vascular dementia, and to compare the Western medicine piracetam. | RCT | Vascular | Dementia | DSM-IV | No | 134 | Chinese medicine combinations and cognitive rehabilitation | Chinese medicine combinations and acupuncture | Chinese medicine combinations and a cupuncture amd cognitive rehabilitation | Piracetam | MMSE |
| 106 | 167 | *Effects of acupuncture on Chinese medicine syndromes of vascular dementia* | G. Shi | China | 2014 | To study the effects of acupuncture on Chinese medicine syndromes of vascular dementia. | RCT | Vascular | Dementia | NINDS-AIREN, 11≤ MMSE ≤ 26, at least grade 9 education | No | 68 | Acupuncture |  |  | Cognitive rehabilitation | SDSVD |
| 107 | 169 | *Acupuncture for Vascular Dementia: A Pragmatic Randomized Clinical Trial* | G. Shi | China | 2015 | Investigate the effectiveness of acupuncture in addition to routine care among patients with VaD. | RCT | Vascular | Dementia | NINDS-AIREN, MMSE 0-23 | No | 68 | Acupuncture |  |  | Best medical treatment | MMSE ADL DEMQOL-proxy |
| 108 | 449 | *Effect of acupuncture treatment on vascular dementia* | J. Yu | China | 2006 | To observe the clinical therapeutic effects for VaD of "yi qi tiao xue, fu ben pei yuan" acupuncture method. | RCT | Vascular | Dementia | NINDS-AIREN; HIS; MMSE (≤ 23) | No | 60 | ‘yi qi tiao xue, fu ben pei yuan’ (acupuncture) |  |  | Regular acupuncture | MMSE HDS ADL |
| 109 | 457 | *Effects of rTMS Treatment on Cognitive Impairment and Resting-State Brain Activity in Stroke Patients: A Randomized Clinical Trial* | M. Yin | China | 2020 | To identify the effects of rTMS intervention on PSCI patients and its potential neural correlates to behavioral improvements. | RCT | Post-stroke; acute/subacute stroke | MCI | Clinical and/or radiological data (first-ever stroke occurred between 1 and 6 months from enrollment, MoCA < 26) | No | 34 | Transcranial Magnetic Stimulation (TMS) and cognitive rehabilitation |  |  | Cognitive rehabilitation | MoCA Neuropsychological tests (2): executive functions, memory ADL functional MRI |
| 110 | 469 | *Effectiveness of acupuncture for vascular cognitive impairment no dementia: a randomized controlled trial* | JW Yang | China | 2019 | To evaluate the effectiveness of acupuncture in patients with vascular cognitive impairment no dementia in comparison with citicoline. | RCT | Vascular | MCI | HIS; MMSE or MoCA scores according to education | No | 216 | Acupuncture |  |  | Citicoline (CDP-Choline) | ADAS-Cog Neuropsychological tests: CDT ADL Chinese version |
| 111 | 488 | *Protective Effect of Hyperbaric Oxygen Therapy on Cognitive Function in Patients with Vascular Dementia* | Y. Xu | China | 2019 | To assess the effect of hyperbaric oxygen on vascular dementia. | RCT | Vascular | Dementia | NINDS-AIREN | No | 158 | Hyperbaric oxygen Donepezil |  |  | Donepezil | MMSE Serum humanin |
| 112 | 527 | *Remote ischemic conditioning may improve outcomes of patients with cerebral small-vessel disease* | Y. Wang | China | 2017 | To evaluate the efficacy of remote ischemic conditioning in patients with cerebral small-vessel disease. | RCT | Subcortical vascular; small vessel disease | MCI | Clinical and/or radiological data (evicence on WMH on MR/CT); MMSE ≥ 20; MoCA ≤ 26 | No | 30 | Remote ischemic conditioning |  |  | Placebo | White matter radiological burden MMSE MoCA Hematocrit and/or hemorrheological measures Blood lipids Other laboratory parameters |
| 113 | 889 | *Remote ischemic conditioning improves cognition in patients with subcortical ischemic vascular dementia* | Z. Liao | China | 2019 | To evaluate therapeutic efficacy of remote ischemic conditioning on subcortical ischemic vascular dementia. | RCT | Subcortical vascular | Dementia | DSM-IV; 10 ≤ MMSE ≤ 26; MoCA < 20; CDR 1 or 2; radiological evidence of CVD (MRI) | No | 42 | Remote ischemic conditioning |  |  | Placebo | Neuropsychological tests (4): memory, executive functions, language, visuospatial domain Other laboratory values White matter radiological burden DTI variables (fractional anisotropy and/or mean diffusivity) |
| 114 | 896 | *Cerebral Functional Manipulation of Repetitive Transcranial Magnetic Stimulation in Cognitive Impairment Patients After Stroke: An fMRI Study* | Y. Li | China | 2020 | To figure out cerebral functional manipulation of repetitive TMS in patients with post-stroke cognitive impairment through using the resting-state functional magnetic resonance imaging. | RCT | Post-stroke; acute/subacute stroke | MCI; dementia | Clinical and/or radiological data; MMSE < 24; First-ever and hemorrhagic stroke </= 3 months | No | 30 | Transcranial Magnetic Stimulation (TMS) and cognitive rehabilitation |  |  | Cognitive rehabilitation | MMSE MoCA functional MRI |
| 115 | 1033 | *A comparative study on the acupoints of specialty of Baihui, Shuigou and Shenmen in treating vascular dementia* | X. Lai | China | 2005 | To study the relative specialty of the acupoints of Baihui (DU20), Shuigou (DU26) and Shenmen (HTT) in treating vascular dementia. | RCT | Post-stroke | Dementia | DSM-IV; MMSE ≤ 24; HIS (≥ 7) | No | 50 | Acupuncture |  |  |  | HDS ADL Functional activity questionnaire |
| 116 | 1183 | *Comparative study of the specificities of needling acupoints DU20, DU26 and HT7 in intervening vascular dementia in different areas in the brain on the basis of scale assessment and cerebral functional imaging* | H. Yong | China | 2007 | To compare the relative specificity of needling acupoints Baihui (DU20), Shuigou (DU26) and Shenmen (HT7) in intervening vascular dementia in different areas in the brain through clinical scale assessment and cerebral functional imaging. | RCT | Vascular | Dementia | HIS (≥ 7); HDS-R (< 24) | No | 50 | Acupuncture |  |  | Acupuncture | MMSE ADL FDG-PET SUVr |
| 117 | 1270 | *Randomized, dim light controlled, crossover test of morning bright light therapy for rest-activity rhythm disorders in patients with vascular dementia and dementia of alzheimer's type* | K. Mishima | Japan | 1998 | To compare the therapeutic effect of morning bright and dim light exposure on rest-activity (R-A) rhythm disorders in patients with vascular dementia and patients with dementia of Alzheimer type. | RCT | Vascular | Dementia | DSM-IV; HIS | Yes | 12 | Light Therapy |  |  | Light Therapy | Night-time Rest activity (Absolute) Night-time Rest activity (Percentage to total activity) |
| 118 | 1870 | *The effects of light therapy on mini-mental state examination scores in demented patients* | A. Graf | International | 2001 | To assess whether bright light therapy can improve cognitive functions in patients with Alzheimer-type dementia or vascular dementia. | RCT | Vascular | Dementia | Clinical and/or radiological data; MMSE (≤ 23) | Yes | 12 | Light Therapy |  |  | Light Therapy | MMSE |
| 119 | 2212 | *Effects of Acupuncture on Vascular Cognitive Impairment with No Dementia: A Randomized Controlled Trial* | L. Huang | China | 2021 | To assess whether acupuncture could improve the cognitive function of patients with vascular cognitive impairment with no dementia. | RCT | Vascular | MCI | Hachinski Ischemic Score; MoCA < 26, CDR = 0.5 | No | 120 | Electro-acupuncture |  |  | Placebo | MoCA MMSE Barthel Index Parkside behaviour rating scale |
| 120 | 2329 | *Clinical observation of taking acupuncture at the acupoints based on "four seas theory" for post-stroke cognitive impairment 基于“四海理论”组穴针刺治疗脑卒中后认知障碍的临床观察* | J. Yang | China | 2022 | To observe the clinical effect on post-stroke cognitive impairment treated by acupuncture at the acupoints composed in accordance with the "four seas theory". | RCT | Post-stroke | MCI  Dementia | Other: Cognitive Disorders Professional Committee of Neurology Branch of Chinese Medical Doctor Association; HIS ≥ 7 | No | 35 | Acupuncture  Cognitive rehabilitation |  |  | Cognitive rehabilitation | MMSE  MoCA  SDSVD  Evoked potentials (P300 wave) |
| 121 | 2337 | *Efficacy of Intermittent Theta-Burst Stimulation and Transcranial Direct Current Stimulation in Treatment of Post-Stroke Cognitive Impairment* | J. Chen | China | 2022 | To observe the effects of intermittent theta-burst stimulation and tDCS combined with cognitive train PSCI. | RCT | Post-stroke | MCI  Dementia | Cerebral hemorrhage or cerebral ischemia (according to Chinese guidelines) accompanied by cognitive impairment development (MMSE < 26) within 6 months | No | 21 | Intermittent Theta Burst Stimulation  Computerised cognitive rehabilitation | tDCS  Computerised cognitive rehabilitation |  | Computerised cognitive rehabilitation | LOTCA  modified Barthel Index  functional near-infrared spectroscopy |
| 122 | 2429 | *Effect of repetitive transcranial magnetic stimulation combined with transcranial direct current stimulation on post-stroke dysmnesia: A preliminary study* | L. Wu | China | 2023 | To ascertain the effect of repetitive transcranial magnetic stimulation combined with transcranial direct current stimulation as a bimodal neuromodulatory approach for post-stroke dysmnesia. | RCT | Post-stroke | MCI  Dementia | Clinical and/or radiological data, MoCA < 26 | No | 12 | Computerised Cognitive Rehabilitation | Computerised Cognitive Rehabilitation  tDCS |  | Placebo | MoCA  Neuropsychological tests: memory  EEG  Evoked potentials (P300 wave) |
| 123 | 2549 | *Impact of transcranial direct current stimulation combined with motor-cognitive intervention on post-stroke cognitive impairment* | L. Zhang | China | 2023 | To explore whether the efficacy of transcranial direct current stimulation (tDCS) combined with motor-cognitive intervention was greater than that on applying each method alone for patients with for post-stroke cognitive impairment. | RCT | Post-stroke | MCI  Dementia | Clinical and/or radiological data; MoCA < 26 | No | 30 | tDCS | Cognitive Rehabilitation  Physiotherapy | tDCS  Cognitive Rehabilitation  Physiotherapy |  | MoCA  LOTCA |
| 124 | 2555 | *At-home tDCS of the left dorsolateral prefrontal cortex improves visual short-term memory in mild vascular dementia* | V. Mylius | Europe | 2016 | To evaluate the effect of transcranial direct current stimulation of the left dorsolateral prefrontal cortex (DLPFC) on cognitive functions in patient affect by mild vascular dementia. | RCT | Vascular | Dementia | Clinical and/or radiological data | No | 13 | tDCS |  |  | Placebo | ADAS-CoG  Depression scales (aggregated)  Neuropsychological tests (2): memory,  executive functions  CGI |
| 125 | 2574 | *Non-invasive cortical stimulation improves post-stroke attention decline* | N. Paik | South Korea | 2009 | To assess if a single session of non-invasive cortical stimulation, in the form of excitatory anodal tDCS applied to the left DLPFC, improves attention in stroke patients. | RCT | Post-stroke | MCI  Dementia | Clinical and/or radiological data; MMSE ≤ 25 | No | 10 | tDCS |  |  | Placebo | Neuropsychological tests: attention |
| 126 | 2580 | *Remote Ischemic Post-Conditioning may Improve Post-Stroke Cognitive Impairment: a Pilot Single Center Randomized Controlled Trial* | J. Zhao | China | 2020 | To evaluate the tolerability and feasibility and the effect of remote ischemic post-conditioning on cognitive functioning in patients with post-stroke cognitive impairment. | RCT | Acute/subacute stroke | MCI | Clinical and/or radiological data; imaging-confirmed stroke (TC/MR), MoCA < 25, mRS ≤ 2, no dementia | No | 24 | Remote Ischemic Conditioning |  |  | Placebo | NIHSS  MoCA  ADAS-CoG |
| 127 | 2608 | *Cerebral activity manipulation of low-frequency repetitive transcranial magnetic stimulation in post-stroke patients with cognitive impairment* | W. Shiyan | China | 2022 | To evaluate the therapeutic effect of low-frequency repetitive transcranial magnetic stimulation (rTMS) on post-stroke cognitive impairment. | RCT | Post-stroke | MCI  Dementia | Clinical and/or radiological data: previous history or stroke; MMSE < 20 | No | 18 | rTMS |  |  | Placebo | LOTCA  P300 |
| 128 | 909 | *The effect of combined scalp acupuncture and cognitive training in patients with stroke on cognitive and motor functions* | J. Xiong | China | 2020 | To investigate the effect of combined scalp acupuncture and cognitive training on cognitive and motor functioning in patients with stroke during the recovery stage. | RCT | Post-stroke; acute/subacute stroke | MCI | Clinical and/or radiological data | No | 70 | Acupuncture and cognitive rehabilitation |  |  | Placebo and cognitive rehabilitation | MMSE LOTCA FMA modified ADL BDNF NGF |
| 129 | 2344 | *The effects and mechanisms of transcranial ultrasound stimulation combined with cognitive rehabilitation on post-stroke cognitive impairment* | S. Chen | China | 2022 | To investigate whether transcranial ultrasound stimulation could improve cognition in patients with post-stroke cognitive impairment treated with cognitive rehabilitation. | RCT | Post-stroke | MCI | Clinical and/or radiological data; 17 ≤ MoCA ≤ 25 | No | 30 | Transcranial ultrasonic stimulation (TUS)  Cognitive rehabilitation |  |  | Cognitive rehabilitation  Sham TUS | MMSE  modified Barthel Index  ADAS-CoG  Evoked potentials (P300 wave)  BDNF |
| 130 | 2369 | *Effect of Interactive Dynamic Scalp Acupuncture on Post-Stroke Cognitive Function, Depression, and Anxiety: A Multicenter, Randomized, Controlled Trial* | Y. Wang | China | 2022 | To compare the clinical effects of interactive dynamic scalp acupuncture, simple combination therapy, and traditional scalp acupuncture on cognitive function, depression simple combination therapy, and traditional scalp acupuncture on cognitive function, depression  and anxiety in patients with post-stroke cognitive impairment. | RCT | Post-stroke | MCI  Dementia | Clinical and/or radiological data: diagnosis of stroke, MoCA < 26, manifestation of “clear mind” | No | 200 | Dynamic Scalp Acupuncture  Computerised Cognitive Rehabilitation | Acupuncture (morning) + Computerised Cognitive Rehabilitation (afternoon) | Acupuncture |  | MoCA  MMSE  Hamilton Depression Rating Scale  Ryden Aggression Scale  modified Barthel Index |
| 131 | 2539 | *Effects of combined use of intermittent theta burst stimulation and cognitive training on post-stroke cognitive impairment : a single-blind randomized controlled trial* | J. Chen | China | 2022 | To assess the therapeutic efficacy of intermittent theta burst stimulation combined with cognitive training for PSCI. | RCT | Post-stroke | MCI  Dementia | Other: Stroke (Chinese guidelines for the diagnosis and treatment of cerebral hemorrhage 2019 or acute ischemic stroke 2018); MMSE < 26 | No | 19 | Intermittent Theta-burst stimulation |  |  | Computerised Cognitive Rehabilitation | LOTCA  modified Barthel Index  Transcranial US  fNIRS |
| 132 | 2576 | *Home-Based Transcranial Direct Current Stimulation to Enhance Cognition in Stroke: randomized Controlled Trial* | Y. Kim | South Korea | 2022 | To evaluate the cognitive improvement effect and feasibility of remotely supervised tDCS (RS-tDCS) in patients with chronic stroke. | RCT | Post-stroke | MCI  Dementia | Clinical and/or radiological data; MoCA < 26 | No | 12 | tDCS  Computerised cognitive rehabilitation |  |  | Computerised cognitive rehabilitation | MoCA (Korean version)  Dementia rating scale (Korean version)  Neuropsychological tests (3): language, attentive and executive functions |
| 133 | 22 | *Effect of Baduanjin exercise on cognitive function in patients with post-stroke cognitive impairment: a randomized controlled trial* | G. Zheng | China | 2020 | To investigate the effectiveness and safety of Baduanjin training on the cognitive function in stroke survivors with cognitive impairment. | RCT | Post-stroke | MCI; dementia | Cognitive impairment according to DSM-V; first ever stroke > 3 months | No | 48 | Baduanjin training |  |  | Best medical treatment | MoCA Neuropsychological tests (4): attentive and executive functions, memory, CDT Barthel Index |
| 134 | 671 | *The efficacy of computerized cognitive training in patients with vascular cognitive impairment, no dementia (the cog-vaccine study): a randomized controlled trial* | Y. Tang | China | 2019 | To assess the efficacy of multidomain, adaptive, computerised cognitive training in subjects with subcortical vascular cognitive impairment no dementia. | RCT | Subcortical vascular | MCI | Clinical and/or radiological data (CDR, MMSE ≤ 20 or ≤ 24 according to education, normal or slighlty impaired IADL/ADL), Fazekas ≥ 2) | No | 60 | Computerized cognitive rehabilitation |  |  | Computerized cognitive rehabilitation | MoCA Neuropsychological tests (3): executive functions, language, memory ADL Neuropsychiatric inventory (NPI) functional MRI |
| 135 | 684 | *Effect of Attention Training in Mild Cognitive Impairment Patients with Subcortical Vascular Changes: The RehAtt Study* | L. Pantoni | Italy | 2017 | To test the effects of cognitive rehabilitation in these patients using the Attention Process Training-II (APT-II) program in a single-blinded, randomized clinical trial. | RCT | Subcortical vascular; small vessel disease | MCI | Clinical and/or radiological data | No | 46 | Attention training |  |  | Best medical treatment | ADL IADL SF-36 EuroQoL EQ-5D questionnaire MMSE MoCA DAD |
| 136 | 789 | *Adaptive conjunctive cognitive training (ACCT) in virtual reality for chronic stroke patients: a randomized controlled pilot trial* | M. Maier | Europe | 2020 | To test a rehabilitation program in virtual reality that trains various cognitive domains in conjunction, by adapting to the patient's disability and while investigating the influence of comorbidities. | RCT | Post-stroke | MCI; dementia | Clinical and/or radiological data (Cognitive impairment due to first-ever stroke, MoCA < 26) | No | 38 | Computerized cognitive rehabilitation |  |  | Cognitive rehabilitation | Neuropsychological tests (5): attentive functions, memory, executive functions, visuospatial domain, other MoCA MMSE |
| 137 | 848 | *Aerobic exercise and vascular cognitive impairment: A randomized controlled trial* | T. Liu-Ambrose | Canada | 2016 | To assess the efficacy of a progressive aerobic exercise training program on cognitive and everyday function among adults with mild subcortical ischemic vascular cognitive impairment. | RCT | Subcortical vascular | MCI | Clinical and/or radiological data; MoCA < 26, MMSE > 20 | No | 70 | Aerobic exercise |  |  | Best medical treatment | ADAS-Cog ADL Neuropsychological tests: executive functions |
| 138 | 1521 | *Training Rehabilitation as an Effective Treatment for Patients With Vascular Cognitive Impairment With No Dementia* | H. Feng | China | 2016 | To investigate the possible therapeutic effect of cognitive training on the cognitive function of patients with vascular cognitive impairment with no dementia. | RCT | Vascular | MCI | HIS | No | 80 | Cognitive rehabilitation |  |  | Best medical treatment | Neuropsychological tests (6): visuospatial domain, memory, language, attentive and executive functions, CDT |
| 139 | 2101 | *Improving Cognitive Function in Patients with Stroke: Can Computerized Training Be the Future?* | R. De Luca | Italy | 2018 | To evaluate the effects of computerised cognitive training with Erica software in patients with stroke. | RCT | Post-stroke | MCI | Other: brain vascular injury + MMSE 12 - 20 | No | 35 | Cognitive rehabilitation Computerized cognitive rehabilitation |  |  | Cognitive rehabilitation | MMSE Neuropsychological tests (4): language, attentive and executive functions, memory, praxis/dexterity Hamilton Rating Scale |
| 140 | 2289 | *Application of Immersive Virtual-Reality-Based Puzzle Games in Elderly Patients with Post-Stroke Cognitive Impairment: A Pilot Study* | M. Chen J. Jia | China | 2022 | To evaluate immersive virtual reality-based puzzle game in treatment of patients with post-stroke cognitive impairment. | RCT | Post-stroke | MCI  Dementia | Other: Chinese Guidlines for Prevention and Treatment of Cerebrovascular Diseases; 18 ≤ MoCa ≤ 26; age 60-90 y | No | 15 | Computerised Cognitive Rehabilitation |  |  | Standard Cognitive Rehabilitation | MoCA  Neuropsychological tests (4): attention, executive functions, memory, language  modified Barthel Index |
| 141 | 2362 | *Effects of a Combined Motor Imagery and Action Observation Intervention on Vascular Cognitive Impairment: A Randomized Pilot Study* | A. Dong | China | 2022 | To evaluate combine motor imagery and action observation therapy on vascular cognitive impairment. | RCT | Vascular | MCI  Dementia | Other: NACCD revised (1995) | No | 10 | Cognitive rehabilitation | Motor imagery with cues | Cognitive rehabilitation  Motor imagery with cues |  | MoCA  Neuropsychological tests: memory  Evoked potentials (P300 wave) |
| 142 | 2559 | *Effects of combined intervention of physical exercise and cognitive training on cognitive function in stroke survivors with vascular cognitive impairment: a randomized controlled trial* | X. P. Wang | China | 2019 | To evaluate the possible effect of the combined intervention of physical exercise and cognitive training on cognitive function in stroke survivals with vascular cognitive impairment. | RCT | Acute/subacute stroke  Post-stroke | MCI  Dementia | NINDS-Canadian Stroke Network criteria | No | 44 | Computerised Cognitive rehabilitation  Aerobic exercise | Aerobic exercise | Computerised Cognitive rehabilitation | Best medical treatment | Neuropsychological tests (3): attention, executive functions, visuospatial functions |
| 143 | 2572 | *A randomized controlled trial of the group reminiscence approach in patients with vascular dementia* | T. Ito | Japan | 2007 | To evaluate the beneficial effect of the group reminiscence approach (GRA) in patients with vascular dementia on the aspect of cognitive and observed behavioral parameters. | RCT | Vascular | Dementia | ADDTC; 10 ≤ MMSE ≤ 24 | No | 18 | Reminescence Therapy | Social contact |  | Best medical treatment | MMSE  Cognitive ability screening Instrument  Multidimensional Observation Scale for Elderly Subjects |
| 144 | 2599 | *Effects of dual-task training in patients with post-stroke cognitive impairment: a randomized controlled trial* | W. Gong | China | 2022 | To analyze the effects of CMDT training on cognitive function, neuron electrophysiology, and frontal lobe hemodynamics in patients with PSCI. | RCT | Post-stroke | MCI  Dementia | Clinical and/or radiological data: IQCODE ≤ 3.3, 9 ≤ MMSE ≤ 27, 9 ≤ MoCA ≤ 26 | No | 17 | Cognitive-motor dual task training |  |  | Standard cognitive rehabilitation | MMSE  MoCA  Evoked potentials (P300 wave)  fNIRS |
| 145 | 2604 | *The Effects of Enriched Rehabilitation on Cognitive Function and Serum Glutamate Levels Post-stroke* | J. Gong | China | 2022 | To explore the effect of enriched rehabilitation on cognitive function and serum glutamate levels in patients with stroke. | RCT | Post-stroke | MCI  Dementia | Clinical and/or radiological data: 18 ≤ MoCA ≤ 23, ≤3 months from stroke | No | 20 | Enhanced cognitive rehabilitation |  |  | Best medical treatment | MoCA  Neuropsychological tests (2): attention, executive functions  Serum glutamate  Malonadehyde  serum TNF-alpha |
| 146 | 3041 | *Based on fNIRS Technology: The Effects of Scalp Acupuncture Combined with iTBS on Cognitive Function After Stroke* | S. Lan | China | 2025 | To explore the impact of integrating scalp acupuncture with iTBS therapy on cognitive impairments post-stroke. | RCT | Post-stroke | MCI | Clinical and/or radiological data: confirmed previous stroke; MoCA < 26 and/or MMSE ≥ 21, CDR < 0.5, disease onset between 2-weeks and 6 months after stroke | No | 60 | Acupuncture | Intermittent Theta Burst Stimulation | Acupuncture  iTBS |  | MoCA MMSE  MR metrics |
| 147 | 3051 | *Effect of Stellate Ganglion Block on Dysphagia and Cognitive Impairment in Cerebral Small Vessel Disease: A Randomized Controlled Study* | X. Zeng | China | 2024 | To explore the clinical effect of stellate ganglion block on airway protection, dysphagia, cognitive impairment, and activities of daily living in cerebral small vessel disease patients. | RCT | Subcortical vascular | MCI Dementia | Clinical and/or radiological data:  MMSE < 24 | No | 84 | Stellate Ganglion block |  |  | Best medical treatment | Primary Aspiration Scale  MMSE  Modified Barthel Index |
| 148 | 3064 | *Using Dual-Target rTMS, Single-Target rTMS, or Sham rTMS on Post-Stroke Cognitive Impairment* | H. Wang | China | 2024 | To evaluate the rTMS on post-stroke cognitive impairment. | RCT | Post-stroke | MCI Dementia | Clinical and/or radiological data:  Cognitive impairment after stroke; 9 ≤ MoCA ≤ 26 | No | 57 | rTMS (dual target, L-DLPFC + M1) | rTMS (single darget, L-DLPFC) |  | Sham treatment | MoCA  Modified Barthel Index  Neuropsychological tests (2): attention and executive function  BDNF  VEGF |
| 149 | 3074 | *Physical Activity in Mild Vascular Cognitive Impairment: Results of the AFIVASC Randomized Controlled Trial at 6 Months* | A. Verdelho | Other: Portugal | 2024 | To evaluate the impact of physical activity on cognition (primary outcome), neurocognitive measures, quality of life, functional status, and physical function (secondary outcomes), in patients with mild VCI. | RCT | Vascular | MCI | Clinical and/or radiological data | No | 104 | Physical exercise |  |  | Best medical treatment | Incident dementia  MoCA  Neuropsychological tests (3): attention and executive function, memory  Quality of Life AD  EQ-5D-VAS |
| 150 | 3085 | *A randomized controlled trial of repetitive transcranial magnetic stimulation plus donepezil vs donepezil alone for mild to moderate cognitive impairment due to small vessel cerebrovascular disease* | X. Chen | China | 2024 | To investigate the clinical efficacy of repetitive transcranial magnetic stimulation combined with donepezil hydrochloride compared to donepezil alone in the treatment of mild-to-moderate cognitive impairment in patients with small vessel cerebrovascular disease. | RCT | Subcortical vascular | MCI Dementia | Other: Chinese guidelines for the diagnosis and treatment of cognitive impairment associated with small vessel cerebrovascular disease | No | 130 | Donepezil  Left DLPFC rTMS |  |  | Donepezil | MoCA  MMSE |
| 151 | 3102 | *Galantamine combined with cognitive rehabilitation on post-stroke cognitive impairment: a proof-of-concept study* | M. Planton | Other: France | 2025 | To evaluate the effects of galantamine treatment versus placebo combined to cognitive rehabilitation after stroke. | RCT | Post-stroke | MCI | Other: VASCOG criteria; at least 3 months after the first symptomatic ischemic stroke | No | 22 | Galantamine  Cognitive rehabilitation |  |  | Cognitive rehabilitation | Incident dementia  Neuropsychological tests (4): attention and executive function, memory, language  BDI  SF-36 |
| 152 | 3110 | *Efficacy of Cognitive Stimulation Therapy for Cognition in Patients with Vascular Cognitive Impairment: A Pilot Randomized Controlled Trial* | Y. Otaka | Other: Japan | 2024 | To investigate the efficacy of cognitive stimulation therapy on cognitive function in patients with VCI admitted to a convalescent rehabilitation ward. | RCT | Post-stroke; acute/subacute stroke | MCI Dementia | Clinical and/or radiological data:  10 ≤ MMSE ≤ 23 | No | 20 | Cognitive stimulation Therapy  Conventional rehabilitation (cognitive + motor) |  |  | Conventional rehabilitation (cognitive + motor) | MMSE  FIM  DBD  NPI  VI  DEMQOL |
| 153 | 3124 | *Electroacupuncture improves vascular cognitive impairment no dementia: A Randomized Clinical Trial* | W. Liu | China | 2024 | To investigate the effect of electroacupuncture on vascular cognitive impairment no-dementia cognitive function  based on the default network. | RCT | Vascular | MCI | Clinical and/or radiological data:  MoCA 18-26; at least 6 months from previous stroke | No | 140 | Electroacupuncture |  |  | Best medical treatment | MoCA  Neuropsychological tests (3): attention, memory, visuospatial functions  resting fMRI metrics |
| 154 | 3133 | *Effect of Repetitive Transcranial Magnetic Stimulation on Post-Stroke Comorbid Cognitive Impairment and Depression: A Randomized Controlled Trial* | R. Feng | China | 2024 | To verify whether repetitive transcranial magnetic stimulation can improve PSCID symptoms and explore the underlying roles of resting-state functional magnetic resonance imaging. | RCT | Post-stroke; acute/subacute stroke | MCI  Dementia | Clinical and/or radiological data:  MMSE < 24; HDRS-17 ≥ 7; first-ever stroke confirmed by imaging (CT or MR); duration of more than 1 week (less than 12 weeks) | No | 30 | Sertraline  rTMS  Acupuncture |  |  | Acupuncture | MMSE  HDRS  resting fMRI metrics  Evoked potentials (P300) |
| 155 | 3154 | *The effects of moderate-intensity aerobic exercise on cognitive function in individuals with stroke-induced mild cognitive impairment: a randomized controlled pilot study* | H. Ou | China | 2024 | To examine the effects of moderate-intensity aerobic exercise on cognitive function, specifically working memory, in individuals with stroke-induced MCI. | RCT | Post-stroke | MCI | Clinical and/or radiological data:  18 ≤ MoCA ≤ 25 | No | 29 | Aerobic Exercise  Motor rehabilitation  Occupational Therapy  Acupuncture |  |  | Motor rehabilitation  Occupational Therapy  Acupuncture | MoCA  MMSE  Neuropsychological tests (1): memory |
| 156 | 3161 | *Effects of Mobile Intelligent Cognitive Training for Patients with Post-Stroke Cognitive Impairment: A 12-Week, Multicenter, Randomized Controlled Study* | H. Zhang | China | 2024 | To investigate the effect of mobile intelligent cognitive training (MICT) on patients with post-stroke cognitive impairment (PSCI). | RCT | Post-stroke; acute/subacute stroke | MCI  Dementia | Clinical and/or radiological data:  MoCA < 26;  10 ≤ MMSE ≤ 27 | No | 518 | Mobile Intelligent Cognitive Training |  |  | Best medical treatment | MoCA  MMSE  Neuropsychological tests (1): memory  modified Barthel Index  FAQ  QLI |
| 157 | 3171 | *Effects of personalized music listening on post-stroke cognitive impairment: A randomized controlled trial* | X. Zhou | China | 2024 | To investigate the effects of personalised music listening on mood improvement, activities of daily living, and cognitive functions in individuals with PSCI. | RCT | Post-stroke | MCI  Dementia | Clinical and/or radiological data:  MoCA ≤ 26 | No | 40 | Personalised Music Playlist Listening |  |  | Placebo (white noise) | MoCA  HARS  HDRS  NIHSS  modified Barthel Index  ZCBI |
| 158 | 3184 | *Computer-aided cognitive training combined with tDCS can improve post-stroke cognitive impairment and cerebral vasomotor function: a randomized controlled trial* | Y. Qu | China | 2024 | To evaluate the effectiveness of both CACT and tDCS on cognitive and cerebrovascular function after stroke, and to explore whether CACT combined with tDCS is more effective. | RCT | Post-stroke | MCI  Dementia | Clinical and/or radiological data | No | 72 | tDCS  Computerised cognitive rehabilitation | Computerised cognitive rehabilitation | tDCS | Cognitive rehabilitation | MoCA  IADL  TC-US |
| 159 | 3197 | *Interactions between tDCS treatment and COMT Val158Met in poststroke cognitive impairment* | Y. Ai | China | 2023 | To explore the effect of catechol-O-methyltransferase Val158Met and brain-derived neurotrophic factor Val66Met to post-stroke cognitive impairment and the interaction with transcranial direct current stimulation . | RCT | Post-stroke; acute/subacute stroke | MCI  Dementia | Clinical and/or radiological data:  MMSE ≤ 26, previous stroke (within 2 weeks -6 months) | No | 80 | tDCS |  |  | Sham treatment | MoCA  Neuropsychological tests (1): executive functions  MR metrics  EEG metrics |
| 160 | 4765 | *Activation changes in patients with post-stroke cognitive impairment receiving intermittent theta burst stimulation: A functional near-infrared spectroscopy study* | J. Chen | China | 2024 | To investigate the potential benefits of using iTBS in patients with PSCI. | RCT | Post-stroke | MCI  Dementia | Clinical and/or radiological data:  first-ever stroke within previous 1-6 months; 11 ≤ MMSE ≤ 26 | No | 44 | iTBS  Cognitive rehabilitation |  |  | Cognitive rehabilitation | MR metrics  MoCA  modified Barthel Index |
| 161 | 4862 | *Evaluation of Metformin on Cognitive Improvement in Patients With Non-dementia Vascular Cognitive Impairment and Abnormal Glucose Metabolism* | D. Zang | China | 2018 | To investigate the effect of metformin on cognitive function in patients with abnormal glucose metabolism and non-dementia vascular cognitive impairment (NDVCI). | RCT | Vascular | MCI | Clinical and/or radiological data:  MMSE ≥ 20; MoCA < 26, “ADL” ≤ 26 (*specific scale unclear*) | No | 100 | Donepezil  Metformine |  |  | Donepezil  Acarbose | ADAS-CoG  Neuropsychological tests (2): memory and executive functions  Carotid US (IMT)  HbA1C |
| 162 | 4898 | *Electroacupuncture combined with cognitive rehabilitation outperforms cognitive rehabilitation alone in treating post-stroke cognitive impairment: a randomized controlled trial* | J. Yao | China | 2025 | To examine whether electroacupuncture combined with cognitive rehabilitation training offers greater benefits than cognitive rehabilitation training alone in improving PSCI. | RCT | Post-stroke | MCI  Dementia | Clinical and/or radiological data:  "*Present of cognitive impariement based on patient's chief complaint or informant's report*" | No | 50 | Electroacupuncture  Cognitive rehabilitation |  |  | Cognitive rehabilitation | MoCA  Neuropsychological tests (3): memory, language and attention  DTI metrics |
| 163 | 5557 | *Enriched rehabilitation on brain functional connectivity in patients with post-stroke cognitive impairment* | J. Wang | China | 2025 | To observe the effect of enrichment rehabilitation on cognitive function in post-stroke patients. | RCT | Post-stroke | MCI  Dementia | Clinical and/or radiological data:  20 ≤ Chinese MoCA version score ≤ 23 | No | 40 | Enriched rehabilitation training |  |  | Conventional cognitive rehabilitation | MoCA  Neuropsychological tests (2): attention and executive functions |
| 164 | 5575 | *Bolstering Cognitive and Locomotor Function in Post-Stroke Dementia Using Human-Robotic Interactive Gait Training* | J. H. You | Other: Korea | 2023 | To compare the effects of human–robotic interactive gait training and conventional physiotherapy on cognitive and sensorimotor functions, trunk balance and coordination, dynamic and static balance, and activities related to daily living performance in patients with post-stroke depression. | Non-randomised experimental study | Post-stroke | Dementia | Clinical and/or radiological data:  10 ≤ MMSE ≤ 25, diagnosis of dementia with previous stroke within 12 months | No | 48 | Human-robotic interface gait training |  |  | Motor rehabilitation | MMSE  FMA  TIS  BBI  modified Barthel Index |
| 165 | 5584 | *Effects of repetitive transcranial magnetic stimulation on cognitive function and hormone levels in early stroke patients with low thyroid hormone levels* | J. Ma | China | 2024 | To observe the effects of repetitive transcranial  magnetic stimulation on cognitive function in early older stroke patients with low thyroid hormone levels. | RCT | Post-stroke | MCI | Clinical and/or radiological data:  MoCA < 26 | No | 40 | rTMS  Cognitive rehabilitation |  |  | Cognitive rehabilitation | MoCA  Neuropsychological tests (2): language and executive functions  modified Barthel Index  serum thyroid hormone  serum TSH |
| 166 | 5593 | *Clinical Effect of Shibing Xingnao Granules on Vascular Dementia Patients and Its Effect on Serum Neuronal Apoptosis Molecules* | P. Li | China | 2023 | To study the clinical effect of Shibing Xingnao Granules on patients with vascular dementia (VD) and to explore its effect on serum neuronal apoptosis molecule levels in VD patients. | RCT | Post-stroke | Dementia | Hachinski Ischemic Score > 7 | No | 78 | Shibing Xingnao granules  Acupuncture |  |  | Acupuncture | MMSE  NIHSS  CDR  ADL |
| 167 | 5655 | *Efficacy and safety of high-dose and personalized TBS on post-stroke cognitive impairment: A randomized controlled trial* | H. Zhang | China | 2025 | To assess the efficacy, dose-dependent effect, and safety of high-dose iTBS targeting personalized frontoparietal cognitive network in short-term PSCI rehabilitation. | RCT | Post-stroke | MCI  Dementia | Clinical and/or radiological data:  Chinese criteria; MoCA ≤ 25 or 10 ≤ MMSE ≤ 26; first-time stroke within previous 1-12 months | No | 45 | iTBS  Computerised cognitive rehabilitation | iTBS  Computerised cognitive rehabilitation |  | Sham treatment  Computerised cognitive rehabilitation | MoCA  WAIS-R IQ  MMSE |
| 168 | 5671 | *Efficacy of home based computerized adaptive cognitive training in patients with post stroke cognitive impairment: a randomized controlled trial* | A. K. Soni | Other: India | 2025 | To assess the effectiveness of a 4-week, remotely delivered, multi-domain, computer-adaptive cognitive retraining (CACT) program in patients with post-cognitive cognitive impairment. | RCT | Post-stroke; acute/subacute stroke | MCI  Dementia | Other: AHA/ASA  Male only; within 3 months after occurrence of stroke | No | 62 | Costumised computerised cognitive rehabilitation |  |  | Best medical treatment | Neuropsychological tests (5): attention and executive functions, language, memory and visuospatial functions  MoCA  IADL  modified Barthel Index |
| 169 | 5719 | *Low frequency-repetitive transcranial magnetic stimulation combined with Xingnao Kaiqiao acupuncture improves post-stroke cognitive impairment and has better clinical efficacy* | D. Li | China | 2024 | To compare and analyse the effects of low-frequency rTMS combined with XNKQ acupuncture on the PSCI efficacy and levels of inflammatory factors in PSCI to provide new insights and potential research avenues for the clinical management of PSCI. | Non-randomised experimental study | Post-stroke | MCI  Dementia | Clinical and/or radiological data:  Expert onsensus on the Management of Cognitive Impairment after Stroke of China; stroke confirmed by imaging; PSCI course < 6 months | No | 192 | Acupuncture  rTMS |  |  | rTMS | MMSE  MoCA  Evoked potentials (P300)  sTNF-alfa  IL-6  IL-1beta  IL-10 |
| 170 | 5723 | *Intermittent theta burst stimulation combined with cognitive training improves cognitive dysfunction and physical dysfunction in patients with post-stroke cognitive impairment* | X. Shu and T. Zhou | China | 2024 | To evaluate the effects of combination of iTBS with cognitive training on physical/cognitive dysfunctions in PSCI patients. | Non-randomised experimental study | Post-stroke | MCI  Dementia | Clinical and/or radiological data:  MMSE ≤ 26; first stroke less than 6 months before enrolment | No | 50 | iTBS  Cognitive rehabilitation |  |  | Sham treatment  Cognitive rehabilitation | MMSE  MoCA  Neuropsychological tests (1): executive functions  Barthel Index  FMA  3D gait analysis |
| 171 | 5730 | *Effect of combined use of Buyang Huanwu decoction and olanzapine on clinical symptoms, neurological function, and degree of dementia in patients with vascular dementia after cerebral ischemic stroke* | N. Chen | China | 2023 | To determine the efficacy and safety of Buyang Huanwu Decoction (BHD) + olanzapine in the treatment of vascular dementia (VD) after cerebral ischemic stroke (CIS). | Non-randomised experimental study | Post-stroke | Dementia | Clinical and/or radiological data:  dementia duration of at least 3 months | No | 90 | Buyang Huanwu decoction  Olanzapine |  |  | Olanzapine | TCM-SS  NIHSS  ADL (Chinese version)  CDR  IL-6  BDNF  IL-10 |
| 172 | 5733 | *The efficacy and safety of nimodipine in acute ischemic stroke patients with mild cognitive impairment: a double-blind, randomized, placebo-controlled trial* | Y. Wang | China | 2019 | To study the efficacy and safety of Nimodipine in treating patients with AIS and VaMCI. | RCT | Acute/subacute stroke | MCI | Clinical and/or radiological data:  acute ischemic stroke within 7 days | No | 654 | Nimodipine |  |  | Placebo | MMSE  ADAS-CoG  MoCA  Neuropsychological tests (1): executive functions |
| 173 | 5736 | *A Preliminary Finding: N-butyl-phthalide Plays a Neuroprotective Role by Blocking the TLR4/HMGB1 Pathway and Improves Mild Cognitive Impairment Induced by Acute Cerebral Infarction* | Y. Lv | China | 2024 | To investigate the clinical efficacy and mechanism of action of NBP for treating ACI-induced MCI. | RCT | Post-stroke; acute/subacute stroke | MCI  Dementia | Clinical and/or radiological data:  Acute-stroke with MRI/CT confirmation; single territory infarct; MCI according to Petersen criteria | No | 86 | Butylphtalide |  |  | Placebo | MoCA  MR metrics (Infarct size)  ADL |

Notes: *Single neuropsychological tests have been aggregated according to the cognitive domains tested (either according to the specifications reported in the study or, if lacking, to test description and classification according to standard neuropsychological toolkits). For studies reporting test spanning more than one domain (n), neuropsychological outcomes are reported as follows: "neuropsychological tests (n): domains tested".

*Abbreviations*: *AD, Alzheimer’s disease;* *ADAS,* Alzheimer's Disease Assessment Scale; *ADCG-CGIC*, Alzheimer's Disease Cooperative Study-Clinical Global Impression of Change; *ADDTC*, *State of California Alzheimer’s Disease Diagnostic and Treatment Centers*; *ADFACS,* Alzheimer’s Disease Functional Assessment and Change Scale; *ADL,* Activities of Daily Living; *ASA,* acetylsalicylic acid; *BBI,* Berg Balance Index; *BDI,* Beck Depression Inventory; *BDNF****,*** brain derived neurotrophic factor; *BEHAVE-AD*, Behavioral Pathology in Alzheimer's; *BGP,* Beurteilungsskala für Geriatrische Patienten; *BPSD*, behavioral and psychological symptoms of dementia; *CCSE,* *Cognitive Capacity Screening Examination*; *CDR,* Clinical Dementia Rating Scale; *CDR-sb,* Clinical Dementia Rating Scale sum of boxes; *CDT,* Clock Drawing Test; *CGI*, Clinical Global Impression; *CIBIC-plus,* Clinician's Interview-Based Impression of Change Plus caregiver input; *CIRS,* Cumulative Illness Rating Scale; *CM-SS*, Chinese Medicine Symptom Scale; *DAD*, Disability Assessment for dementia; *DBD,* Dementia Behaviour Disturbance scale; DEMQoL, Dementia Quality of Life instruments (self-reported by patient)*; DEMQoL-proxy,* DEMQoL as reported by caregiver; *DS*, Blessed Dementia Scale; *DSM,* *Diagnostic and Statistic Manual of Mental Disorders; DTI*, diffusion tensor imaging; *EEG,* electroencephalogram; *EQ-5D-VAS*, EuroQol Visual Analogue Scale; *FAQ,* Functional Activity Questionnaire; *FDG-PET SUVr*, fluorodeoxyglucose - Positron emission tomography standardised uptake values ratio; *FIM,* Functional Independence Measures; *FMA,* Fugl-Meyer assessment; *fMRI*, functional magnetic resonance imaging; *fNIRS,* functional near-infrared spectroscopy; *GBS,* Gottfries-Bråne-Steen Scale; *GDS,* Geriatric Depression Scale; *HAM-D,* Hamilton Depression Rating Scale; *HDS*, Hasegawa Dementia Scales; *HIS*, Hachinski Ischemic Score; *IADL*, Instrumental activity of daily living; *IL,* interleukin; *IMT,* intima-media thickness; *LOTCA*, Loewenstein Occupational Therapy Cognition Assessment; *MDRS*, Mattis Dementia Rating Scale; *MID,* multi-infarct dementia; *MMSE*, Mini-Mental State Examination; *MoCA*, Montreal Cognitive Assessment; *MS*, Matthew Scale; *NAI,* Nuremberg Activity Inventory; *NGF,* Nerve Growth Factor; *NIHSS,* National Institute of Health Stroke Scale; *NINCDS-ADRDA*, National Institute of Neurological and Communicative Diseases and Stroke/Alzheimer's Disease and Related Disorders Association; *NINDS-AIREN*, National Institute of Neurological Disorders and Stroke and the Association Internationale pour la Recherche et l'Enseignement en Neurosciences criteria; *NOSGER,* Nurses Observation Scale for Geriatric Patients; *NPI*, Neuropsychiatric Inventory; *PSQI*, Pittsburgh Sleep Quality Index; *QLI*, Quality of Life Index; *rCBF*, *regional Cerebral Blood Flow*; *RCT, Randomised Controlled Trial*; *RDRS*, Rapid Disability Rating Scale; *RSS,* Relative Stress Scale; *SCAG*, Sandoz Clinical Assessment Geriatric scale; *SDAT*, senile dementia of the Alzheimer type; *SDSVD*, Scale of differentiation of syndromes of vascular dementia; *SF,* Short-form healthy survey; *SKT,* Erzigkeit’s Short Cognitive Performance Test; *SOD,* superoxide dismutase; *tDCS*, transcranial Direct Current Stimulation; *TCM-SS,* Traditional Chinese Medicine – symptom scale; *TIS,* Trunk Impairment Scale; *TUS*, Transcranial Ultrasonic Stimulation; *sTNF-alpha,* serum tumor necrosis factor alpha*;* *VaD*, Vascular dementia; *VaDAS*, Vascular dementia Assessment Scale; *VEGF,* Vascular Endothelial Growth Factor; *VI,* Vitality Index; *WAIS*, Wechsler Adult Intelligence Scale; *WMH,* White matter hyperintensities; *ZCBI,* Zarit Caregiver Burden Inventory.
