# Supplementary material for: Therapeutic strategies in vascular cognitive impairment: A systematic review and meta‐analysis
Source: Alzheimers Dement. 2025 Nov 6;21(11):e70840. doi: 10.1002/alz.70840 (PMC12591988; doi:10.1002/alz.70840)
Supplement: Supplementary file 4 — Supporting Information [file ALZ-21-e70840-s005.docx]

***Supplementary File 4*** – **Consensus quality assessment according to National Institute of Health Quality Assessment Tool of Controlled Intervention Studies for each of the included studies**.

| **Unique ID** | **Study ID** | **1) RANDOMIZ** | **2) RANDOMIZ METHOD** | **3) TREATMENT ALLOC** | **4) PARTICIPANT BLINDING** | **5) OPERATOR BLINDING** | **6) GROUP SIMILARITY** | **7) OVERALL DROP-OUT** | **8) DIFFERENT DROP-OUT** | **9) ADHERENCE** | **10) OTHER INTERV AVOID** | **11) VALID OUTCOMES** | **12) SAMPLE SIZE EST** | **13) PRESPEC ANALYSES** | **14) ITT ANALYSES** | **OVERALL QUALITY** |
| --- | --- | --- | --- | --- | --- | --- | --- | --- | --- | --- | --- | --- | --- | --- | --- | --- |
| 1 | Zhang 2020 |  |  |  |  |  |  |  |  |  |  |  |  |  |  |  |
| 2 | Zhang 2016 |  |  |  |  |  |  |  |  |  |  |  |  |  |  |  |
| 3 | Salvadori 2021 |  |  |  |  |  |  |  |  |  |  |  |  |  |  |  |
| 4 | Saletu 1995 |  |  |  |  |  |  |  |  |  |  |  |  |  |  |  |
| 5 | Saletu 1992 |  |  |  |  |  |  |  |  |  |  |  |  |  |  |  |
| 6 | Qi 2020 |  |  |  |  |  |  |  |  |  |  |  |  |  |  |  |
| 7 | Passeri 1989 |  |  |  |  |  |  |  |  |  |  |  |  |  |  |  |
| 8 | Parnetti 1997 |  |  |  |  |  |  |  |  |  |  |  |  |  |  |  |
| 9 | Parnetti 1996 |  |  |  |  |  |  |  |  |  |  |  |  |  |  |  |
| 10 | Xu 2012 |  |  |  |  |  |  |  |  |  |  |  |  |  |  |  |
| 11 | Whyte 2008 |  |  |  |  |  |  |  |  |  |  |  |  |  |  |  |
| 12 | Wei 2012 |  |  |  |  |  |  |  |  |  |  |  |  |  |  |  |
| 13 | Wang 2019 |  |  |  |  |  |  |  |  |  |  |  |  |  |  |  |
| 14 | Wang 2015 |  |  |  |  |  |  |  |  |  |  |  |  |  |  |  |
| 15 | Tian 2002 |  |  |  |  |  |  |  |  |  |  |  |  |  |  |  |
| 16 | Pantoni 2000 |  |  |  |  |  |  |  |  |  |  |  |  |  |  |  |
| 17 | Pantoni 2005 |  |  |  |  |  |  |  |  |  |  |  |  |  |  |  |
| 18 | Pantoni 2000 |  |  |  |  |  |  |  |  |  |  |  |  |  |  |  |
| 19 | Pan 2018 |  |  |  |  |  |  |  |  |  |  |  |  |  |  |  |
| 20 | Pakdaman 2017 |  |  |  |  |  |  |  |  |  |  |  |  |  |  |  |
| 21 | Orgogozo 2002 |  |  |  |  |  |  |  |  |  |  |  |  |  |  |  |
| 22 | Odinak 2014 |  |  |  |  |  |  |  |  |  |  |  |  |  |  |  |
| 23 | Napryeyenko 2009 |  |  |  |  |  |  |  |  |  |  |  |  |  |  |  |
| 24 | Nadeau 1988 |  |  |  |  |  |  |  |  |  |  |  |  |  |  |  |
| 25 | Marigliano 1992 |  |  |  |  |  |  |  |  |  |  |  |  |  |  |  |
| 26 | Marcusson 1997 |  |  |  |  |  |  |  |  |  |  |  |  |  |  |  |
| 27 | Lu 2020 |  |  |  |  |  |  |  |  |  |  |  |  |  |  |  |
| 28 | Liu 2014 |  |  |  |  |  |  |  |  |  |  |  |  |  |  |  |
| 29 | Xiao 1999 |  |  |  |  |  |  |  |  |  |  |  |  |  |  |  |
| 30 | Winblad 1999 |  |  |  |  |  |  |  |  |  |  |  |  |  |  |  |
| 31 | Wilkinson 2003 |  |  |  |  |  |  |  |  |  |  |  |  |  |  |  |
| 32 | Wilcock 2002 |  |  |  |  |  |  |  |  |  |  |  |  |  |  |  |
| 33 | Rom√°n 2010 |  |  |  |  |  |  |  |  |  |  |  |  |  |  |  |
| 34 | Li 2019 |  |  |  |  |  |  |  |  |  |  |  |  |  |  |  |
| 35 | Leijenaar 2020 |  |  |  |  |  |  |  |  |  |  |  |  |  |  |  |
| 36 | Kanowski 1996 |  |  |  |  |  |  |  |  |  |  |  |  |  |  |  |
| 37 | Kanowski 1990 |  |  |  |  |  |  |  |  |  |  |  |  |  |  |  |
| 38 | Jiang 2014 |  |  |  |  |  |  |  |  |  |  |  |  |  |  |  |
| 39 | Jia 2016 |  |  |  |  |  |  |  |  |  |  |  |  |  |  |  |
| 40 | Jia 2018 |  |  |  |  |  |  |  |  |  |  |  |  |  |  |  |
| 41 | Itoh 1999 |  |  |  |  |  |  |  |  |  |  |  |  |  |  |  |
| 42 | Iranmanesh 2020 |  |  |  |  |  |  |  |  |  |  |  |  |  |  |  |
| 43 | Ihl 2012 |  |  |  |  |  |  |  |  |  |  |  |  |  |  |  |
| 44 | Muresanu 2008 |  |  |  |  |  |  |  |  |  |  |  |  |  |  |  |
| 45 | Muratorio 1992 |  |  |  |  |  |  |  |  |  |  |  |  |  |  |  |
| 46 | Moretti 2008 |  |  |  |  |  |  |  |  |  |  |  |  |  |  |  |
| 46bis | Moretti 2008 |  |  |  |  |  |  |  |  |  |  |  |  |  |  |  |
| 47 | Moretti 2004 |  |  |  |  |  |  |  |  |  |  |  |  |  |  |  |
| 48 | Moretti 2003 |  |  |  |  |  |  |  |  |  |  |  |  |  |  |  |
| 49 | Moretti 2002 |  |  |  |  |  |  |  |  |  |  |  |  |  |  |  |
| 50 | Moretti 2004 |  |  |  |  |  |  |  |  |  |  |  |  |  |  |  |
| 51 | Moretti 2005 |  |  |  |  |  |  |  |  |  |  |  |  |  |  |  |
| 52 | Moretti 2004 |  |  |  |  |  |  |  |  |  |  |  |  |  |  |  |
| 53 | M√∂ller 2001 |  |  |  |  |  |  |  |  |  |  |  |  |  |  |  |
| 54 | Mielke 1996 |  |  |  |  |  |  |  |  |  |  |  |  |  |  |  |
| 55 | Meyer 1989 |  |  |  |  |  |  |  |  |  |  |  |  |  |  |  |
| 56 | Herrmann 1997 |  |  |  |  |  |  |  |  |  |  |  |  |  |  |  |
| 57 | Herrmann 1992 |  |  |  |  |  |  |  |  |  |  |  |  |  |  |  |
| 58 | Hartmann 1985 |  |  |  |  |  |  |  |  |  |  |  |  |  |  |  |
| 59 | Ghose 1987 |  |  |  |  |  |  |  |  |  |  |  |  |  |  |  |
| 60 | Gallai 1991 |  |  |  |  |  |  |  |  |  |  |  |  |  |  |  |
| 61 | Frattola 1991 |  |  |  |  |  |  |  |  |  |  |  |  |  |  |  |
| 62 | Fischhof 1992 |  |  |  |  |  |  |  |  |  |  |  |  |  |  |  |
| 63 | Fischhof 1996 |  |  |  |  |  |  |  |  |  |  |  |  |  |  |  |
| 64 | Erkinjuntti 2003 |  |  |  |  |  |  |  |  |  |  |  |  |  |  |  |
| 65 | Erkinjuntti 2002 |  |  |  |  |  |  |  |  |  |  |  |  |  |  |  |
| 66 | Kittner 1997 |  |  |  |  |  |  |  |  |  |  |  |  |  |  |  |
| 67 | Dichgans 2008 |  |  |  |  |  |  |  |  |  |  |  |  |  |  |  |
| 68 | DiPerri 1991 |  |  |  |  |  |  |  |  |  |  |  |  |  |  |  |
| 69 | Cucinotta 1988 |  |  |  |  |  |  |  |  |  |  |  |  |  |  |  |
| 70 | Cucinotta 1992 |  |  |  |  |  |  |  |  |  |  |  |  |  |  |  |
| 71 | Corona 1989 |  |  |  |  |  |  |  |  |  |  |  |  |  |  |  |
| 72 | Cohen 2003 |  |  |  |  |  |  |  |  |  |  |  |  |  |  |  |
| 73 | Chen 2020 |  |  |  |  |  |  |  |  |  |  |  |  |  |  |  |
| 74 | Hagstadius 1984 |  |  |  |  |  |  |  |  |  |  |  |  |  |  |  |
| 75 | Guekht 2011 |  |  |  |  |  |  |  |  |  |  |  |  |  |  |  |
| 76 | Guekht 2017 |  |  |  |  |  |  |  |  |  |  |  |  |  |  |  |
| 77 | Grossmann 1990 |  |  |  |  |  |  |  |  |  |  |  |  |  |  |  |
| 78 | Black 2003 |  |  |  |  |  |  |  |  |  |  |  |  |  |  |  |
| 79 | Black 1992 |  |  |  |  |  |  |  |  |  |  |  |  |  |  |  |
| 80 | Bergamasco 1992 |  |  |  |  |  |  |  |  |  |  |  |  |  |  |  |
| 81 | Bayer 1996 |  |  |  |  |  |  |  |  |  |  |  |  |  |  |  |
| 82 | Ballard 2008 |  |  |  |  |  |  |  |  |  |  |  |  |  |  |  |
| 83 | Auchus 2007 |  |  |  |  |  |  |  |  |  |  |  |  |  |  |  |
| 84 | Arrigo 1989 |  |  |  |  |  |  |  |  |  |  |  |  |  |  |  |
| 85 | Demarin 2017 |  |  |  |  |  |  |  |  |  |  |  |  |  |  |  |
| 86 | Shi 2020 |  |  |  |  |  |  |  |  |  |  |  |  |  |  |  |
| 87 | Henley 2023 |  |  |  |  |  |  |  |  |  |  |  |  |  |  |  |
| 88 | Lu 2023 |  |  |  |  |  |  |  |  |  |  |  |  |  |  |  |
| 89 | Zhang 2022 |  |  |  |  |  |  |  |  |  |  |  |  |  |  |  |
| 90 | Chen 2023 |  |  |  |  |  |  |  |  |  |  |  |  |  |  |  |
| 91 | Zhu 2023 |  |  |  |  |  |  |  |  |  |  |  |  |  |  |  |
| 92 | Chang 2011 |  |  |  |  |  |  |  |  |  |  |  |  |  |  |  |
| 93 | Narasimhalu 2010 |  |  |  |  |  |  |  |  |  |  |  |  |  |  |  |
| 94 | Chen 2021 |  |  |  |  |  |  |  |  |  |  |  |  |  |  |  |
| 95 | Cotroneo 2013 |  |  |  |  |  |  |  |  |  |  |  |  |  |  |  |
| 96 | Ma 2020 |  |  |  |  |  |  |  |  |  |  |  |  |  |  |  |
| 97 | Mok 2007 |  |  |  |  |  |  |  |  |  |  |  |  |  |  |  |
| 98 | Zhang 2021 |  |  |  |  |  |  |  |  |  |  |  |  |  |  |  |
| 99 | Zhao 2002 |  |  |  |  |  |  |  |  |  |  |  |  |  |  |  |
| 100 | Zhao 2009 |  |  |  |  |  |  |  |  |  |  |  |  |  |  |  |
| 101 | Wang 2016 |  |  |  |  |  |  |  |  |  |  |  |  |  |  |  |
| 102 | Walzl 2000 |  |  |  |  |  |  |  |  |  |  |  |  |  |  |  |
| 103 | Liu 2016 |  |  |  |  |  |  |  |  |  |  |  |  |  |  |  |
| 104 | Yusufu 2022 |  |  |  |  |  |  |  |  |  |  |  |  |  |  |  |
| 105 | Chen 2011 |  |  |  |  |  |  |  |  |  |  |  |  |  |  |  |
| 106 | Shi 2014 |  |  |  |  |  |  |  |  |  |  |  |  |  |  |  |
| 107 | Shi 2015 |  |  |  |  |  |  |  |  |  |  |  |  |  |  |  |
| 108 | Yu 2006 |  |  |  |  |  |  |  |  |  |  |  |  |  |  |  |
| 109 | Yin 2020 |  |  |  |  |  |  |  |  |  |  |  |  |  |  |  |
| 110 | Yang 2019 |  |  |  |  |  |  |  |  |  |  |  |  |  |  |  |
| 111 | Xu 2019 |  |  |  |  |  |  |  |  |  |  |  |  |  |  |  |
| 112 | Wang 2017 |  |  |  |  |  |  |  |  |  |  |  |  |  |  |  |
| 113 | Liao 2019 |  |  |  |  |  |  |  |  |  |  |  |  |  |  |  |
| 114 | Li 2020 |  |  |  |  |  |  |  |  |  |  |  |  |  |  |  |
| 115 | Lai 2005 |  |  |  |  |  |  |  |  |  |  |  |  |  |  |  |
| 116 | Huang 2007 |  |  |  |  |  |  |  |  |  |  |  |  |  |  |  |
| 117 | Mishima 1998 |  |  |  |  |  |  |  |  |  |  |  |  |  |  |  |
| 118 | Graf 2001 |  |  |  |  |  |  |  |  |  |  |  |  |  |  |  |
| 119 | Huang 2021 |  |  |  |  |  |  |  |  |  |  |  |  |  |  |  |
| 120 | Yang 2022 |  |  |  |  |  |  |  |  |  |  |  |  |  |  |  |
| 121 | Chu 2022 |  |  |  |  |  |  |  |  |  |  |  |  |  |  |  |
| 122 | Hu 2023 |  |  |  |  |  |  |  |  |  |  |  |  |  |  |  |
| 123 | Zhang 2023 |  |  |  |  |  |  |  |  |  |  |  |  |  |  |  |
| 124 | André 2016 |  |  |  |  |  |  |  |  |  |  |  |  |  |  |  |
| 125 | Kang 2009 |  |  |  |  |  |  |  |  |  |  |  |  |  |  |  |
| 126 | Li 2020 |  |  |  |  |  |  |  |  |  |  |  |  |  |  |  |
| 127 | Yingli 2022 |  |  |  |  |  |  |  |  |  |  |  |  |  |  |  |
| 128 | Xiong 2020 |  |  |  |  |  |  |  |  |  |  |  |  |  |  |  |
| 129 | Wang 2022 |  |  |  |  |  |  |  |  |  |  |  |  |  |  |  |
| 130 | Zhang 2022 |  |  |  |  |  |  |  |  |  |  |  |  |  |  |  |
| 131 | Zhang 2023 |  |  |  |  |  |  |  |  |  |  |  |  |  |  |  |
| 132 | Ko 2022 |  |  |  |  |  |  |  |  |  |  |  |  |  |  |  |
| 133 | Zheng 2020 |  |  |  |  |  |  |  |  |  |  |  |  |  |  |  |
| 134 | Tang 2018 |  |  |  |  |  |  |  |  |  |  |  |  |  |  |  |
| 135 | Pantoni 2017 |  |  |  |  |  |  |  |  |  |  |  |  |  |  |  |
| 136 | Maier 2020 |  |  |  |  |  |  |  |  |  |  |  |  |  |  |  |
| 137 | Liu-Ambrose 2016 |  |  |  |  |  |  |  |  |  |  |  |  |  |  |  |
| 138 | Feng 2017 |  |  |  |  |  |  |  |  |  |  |  |  |  |  |  |
| 139 | DeLuca 2018 |  |  |  |  |  |  |  |  |  |  |  |  |  |  |  |
| 140 | Liu 2023 |  |  |  |  |  |  |  |  |  |  |  |  |  |  |  |
| 141 | Liu 2022 |  |  |  |  |  |  |  |  |  |  |  |  |  |  |  |
| 142 | Bo 2019 |  |  |  |  |  |  |  |  |  |  |  |  |  |  |  |
| 143 | Ito 2007 |  |  |  |  |  |  |  |  |  |  |  |  |  |  |  |
| 144 | Sun 2022 |  |  |  |  |  |  |  |  |  |  |  |  |  |  |  |
| 145 | Wang 2022 |  |  |  |  |  |  |  |  |  |  |  |  |  |  |  |
| 146 | Zhong 2025 |  |  |  |  |  |  |  |  |  |  |  |  |  |  |  |
| 147 | Zeng 2024 |  |  |  |  |  |  |  |  |  |  |  |  |  |  |  |
| 148 | Xu 2024 |  |  |  |  |  |  |  |  |  |  |  |  |  |  |  |
| 149 | Verdelho 2024 |  |  |  |  |  |  |  |  |  |  |  |  |  |  |  |
| 150 | Shou 2024 |  |  |  |  |  |  |  |  |  |  |  |  |  |  |  |
| 151 | Planton 2025 |  |  |  |  |  |  |  |  |  |  |  |  |  |  |  |
| 152 | Mori 2024 |  |  |  |  |  |  |  |  |  |  |  |  |  |  |  |
| 153 | Lin 2024 |  |  |  |  |  |  |  |  |  |  |  |  |  |  |  |
| 154 | Li 2024 |  |  |  |  |  |  |  |  |  |  |  |  |  |  |  |
| 155 | Huang 2024 |  |  |  |  |  |  |  |  |  |  |  |  |  |  |  |
| 156 | Han 2024 |  |  |  |  |  |  |  |  |  |  |  |  |  |  |  |
| 157 | Fan 2024 |  |  |  |  |  |  |  |  |  |  |  |  |  |  |  |
| 158 | Chen 2024 |  |  |  |  |  |  |  |  |  |  |  |  |  |  |  |
| 159 | Ai 2024 |  |  |  |  |  |  |  |  |  |  |  |  |  |  |  |
| 160 | Yu 2024 |  |  |  |  |  |  |  |  |  |  |  |  |  |  |  |
| 161 | Lin 2018 |  |  |  |  |  |  |  |  |  |  |  |  |  |  |  |
| 162 | Guo 2025 |  |  |  |  |  |  |  |  |  |  |  |  |  |  |  |
| 163 | Huai 2024 |  |  |  |  |  |  |  |  |  |  |  |  |  |  |  |
| 164 | Kim 2023 |  |  |  |  |  |  |  |  |  |  |  |  |  |  |  |
| 165 | Li 2024 |  |  |  |  |  |  |  |  |  |  |  |  |  |  |  |
| 166 | Liu 2023 |  |  |  |  |  |  |  |  |  |  |  |  |  |  |  |
| 167 | Ren 2025 |  |  |  |  |  |  |  |  |  |  |  |  |  |  |  |
| 168 | Soni 2025 |  |  |  |  |  |  |  |  |  |  |  |  |  |  |  |
| 169 | Xun 2025 |  |  |  |  |  |  |  |  |  |  |  |  |  |  |  |
| 170 | Yu 2024 |  |  |  |  |  |  |  |  |  |  |  |  |  |  |  |
| 171 | Zhang 2023 |  |  |  |  |  |  |  |  |  |  |  |  |  |  |  |
| 172 | Zheng 2019 |  |  |  |  |  |  |  |  |  |  |  |  |  |  |  |
| 173 | Zhou 2024 |  |  |  |  |  |  |  |  |  |  |  |  |  |  |  |

Notes: for each single item the consensus rating was colour coded as following: *green (=yes), red (=no), yellow (= cannot determine or not assessable); grey (=not relevant)*. For the overall quality rating (*last column on the right-hand side*): *green* (=good), *yellow* (=fair), *red* (=poor).
Abbreviations: Other Interv. Avoid., other intervention avoided; sample size est, sample size estimation; prespec. Analyses, prespecified statistical analyses; ITT, intention-to-treat.
