# Supplementary material for: Therapeutic strategies in vascular cognitive impairment: A systematic review and meta‐analysis
Source: Alzheimers Dement. 2025 Nov 6;21(11):e70840. doi: 10.1002/alz.70840 (PMC12591988; doi:10.1002/alz.70840)
Supplement: Supplementary file 5 — Supporting Information [file ALZ-21-e70840-s002.docx]

***Supplementary File 5***: **Summary of qualitative summarised studies excluded from semi-quantitative estimation.**

| **Study ID** | **Covidence ID** | **Title** | **Authors** | **Year** | **Design** | **VCI label** | **VCI severity** | **Intervention 1** | **Intervention 2** | **Intervention 3** | **Comparator** | **Reason for exclusion** |
| --- | --- | --- | --- | --- | --- | --- | --- | --- | --- | --- | --- | --- |
| 1 | 58 | *Shenmayizhi Formula Combined with Ginkgo Extract Tablets for the Treatment of Vascular Dementia: A Randomized, Double-Blind, Controlled Trial* | H. Zhang | 2020 | RCT | Vascular | Dementia | Ginkgo Biloba and Shenmayizhi formula |  |  | Placebo and Ginkgo Biloba | Intervention compared against active treatment |
| 2 | 59 | *A clinical trial investigating the effect of trimetazidine combined with oxiracetam in patients with vascular dementia* | C. Zhang | 2016 | RCT | Vascular | Dementia | Trimtazidine and cerebrolysin |  |  | Oxiracetam | Intervention compared against active treatment |
| 3 | 260 | *Efficacy and Safety of the Association of Nimodipine and Choline Alphoscerate in the Treatment of Cognitive Impairment in Patients with Cerebral Small Vessel Disease. The CONIVaD Trial* | E. Salvadori | 2021 | RCT | Subcortical vascular; small vessel disease | MCI; dementia | Nimodipine and and alfa-Glycerylphosphorylcholine/Choline alfoscerate |  |  | Placebo and Nimodipine | Intervention compared against active treatment |
| 4 | 270 | *Nicergoline in senile dementia of Alzheimer type and multi-infarct dementia: A double blind, placebo controlled, clinical and EEG/ERP mapping study* | B. Saletu | 1995 | RCT | Multi-infarct | Dementia | Nicergoline |  |  | Placebo | Not enough data reported for estimation |
| 6 | 350 | *Effects of Butyphthalide Combined with Idebenone on Inflammatory Cytokines and Vascular Endothelial Functions of Patients with Vascular Dementia* | F. Qi | 2020 | RCT | Vascular | Dementia | Idebenone and piracetam and butylphtalide |  |  | Piracetam Idebenone | Intervention compared against active treatment |
| 7 | 420 | *Ateroid in the clinical treatment of multi-infarct dementia* | M. Passeri | 1989 | RCT | Multi-infarct | Dementia | Sulfomucopolysaccharides |  |  | Placebo | Not enough data reported for estimation |
| 8 | 424 | *Vascular dementia italian sulodexide study (VA.D.I.S.S.) clinical and biological results* | L. Parnetti | 1997 | RCT | Vascular | Dementia | Sulodexide |  |  | Pentoxifylline | Intervention compared against active treatment |
| 9 | 427 | *Posatirelin in the treatment of vascular dementia: A double-blind multicentre study vs placebo* | L. Parnetti | 1996 | RCT | Vascular | Dementia | Posatirelin |  |  | Placebo | Not enough data reported for estimation |
| 10 | 486 | *Treatment with Huperzine A Improves Cognition in Vascular Dementia Patients* | Z. Xu | 2012 | RCT | Vascular | Dementia | Huperzine A |  |  | Vitamin C | Intervention compared against active treatment |
| 12 | 511 | *Effects of Chinese medicine for promoting blood circulation and removing blood stasis in treating patients with mild to moderate vascular dementia: A randomized, double-blind and parallel-controlled trial* | M. Wei | 2012 | RCT | Vascular | Dementia | Danshen (Radix Salviae Miltiorrhiae) and Sanqi (Radix Notoginseng) |  |  | Placebo | Combination of interventions |
| 13 | 532 | *Efficacy of butylphthalide soft capsules in patients with vascular dementia and the relevant antioxidative mechanism* | S. Wang | 2019 | RCT | Vascular | Dementia | Butylphtalide |  |  | Donepezil | Intervention compared against active treatment |
| 15 | 646 | *Jian Nao Ning for treatment of memory impairment in patients with mild or moderate multi-infarct dementia* | J. Tian | 2002 | RCT | Multi-infarct | Dementia | Jin Nao Ning |  |  | Duxil | Intervention compared against active treatment |
| 19 | 692 | *Effect of Yin-Xing-Tong-Zhi Tablets on Improving Vascular Cognitive Impairment No Dementia* | W. Pan | 2018 | RCT | Vascular | MCI | Yin-Xing-Tong-Zhi |  |  | Placebo | Not enough data reported for estimation |
| 24 | 749 | *A crossover trial of bromocriptine in the treatment of vascular dementia* | S. E. Nadeau | 1988 | RCT | Vascular | Dementia | Bromocriptine |  |  | Placebo | Not enough data reported for estimation |
| 25 | 762 | *Randomized, double-blind, placebo controlled, multicentre study of idebenone in patients suffering from multi-infarct dementia* | V. Marigliano | 1992 | RCT | Multi-infarct | Dementia | Idebenone |  |  | Placebo | Not enough data reported for estimation |
| 34 | 982 | *Effect of Pushen capsule for treating vascular mild cognitive impairment: a pilot observational study* | S. Li | 2019 | RCT | Vascular | MCI | Pushen capsule |  |  | Ginkgo Biloba | Intervention compared against active treatment |
| 35 | 1004 | *Methylphenidate and galantamine in patients with vascular cognitive impairment-the proof-of-principle study STREAM-VCI* | J. F. Leijenaar | 2020 | RCT | Vascular | MCI; dementia | Methylphenydate | Galantamine |  | Placebo | Not enough data reported for estimation |
| 37 | 1096 | *Efficacy of xantinolnicotinate in patients with dementia* | S. Kanowski | 1990 | RCT | Multi-infarct | Dementia | Xantinolnicotinate |  |  | Placebo | Not enough data reported for estimation |
| 38 | 1119 | *Intervention effect of folic acid and vitamin B12 on vascular cognitive impairment complicated with hyperhomocysteinemia* | B. Jiang | 2014 | RCT | Vascular | MCI | Folic acid Vitamin B12 |  |  | Best medical treatment | Combination of interventions |
| 40 | 1123 | *Efficacy and safety of the compound Chinese medicine SaiLuoTong in vascular dementia: A randomized clinical trial* | J. Jia | 2018 | RCT | Vascular | Dementia | SaiLuo Tong | SaiLuo Tong |  | Placebo Galantamine | Intervention compared against active treatment |
| 45 | 1203 | *A neurotropic approach to the treatment of multi-infarct dementia using L-glycerylphosphorylchlorine* | A. Muratorio | 1992 | RCT | Multi-infarct | Dementia | Alfa-Glycerylphosphorylcholine/Choline alfoscerate |  |  | Citicoline (CDP-Choline) | Intervention compared against active treatment |
| 50 | 1236 | *Olanzapine as a possible treatment for anxiety due to vascular dementia: An open study* | R. Moretti | 2004 | RCT | Vascular | Dementia | Olanzapine |  |  | Bromazepam | Intervention compared against active treatment |
| 51 | 1237 | *Olanzapine as a possible treatment of behavioral symptoms in vascular dementia: Risks of cerebrovascular events - A controlled, open-label study* | R. Moretti | 2005 | Non-randomised experimental study | Vascular | Dementia | Olanzapine |  |  | Promazine, haloperidol | Intervention compared against active treatment |
| 53 | 1255 | *Naftidrofuryl in the treatment of vascular dementia* | H. J. Moller | 2001 | RCT | Vascular | Dementia | Naftidrofuryl | Naftidrofuryl |  | Placebo | Not enough data reported for estimation |
| 55 | 1290 | *Randomized clinical trial of daily aspirin therapy in multi-infarct dementia. A pilot study* | J. Stirling Meyer | 1989 | RCT | Multi-infarct | Dementia | ASA |  |  | Best medical treatment | Not enough data reported for estimation |
| 57 | 1366 | *Moving from the question of efficacy to the question of therapeutic relevance: an exploratory reanalysis of a controlled clinical study of 130 inpatients with dementia syndrome taking piracetam* | W. M. Herrmann | 1992 | RCT | Multi-infarct | Dementia | Piracetam |  |  | Placebo | Not enough data reported for estimation |
| 60 | 1449 | *Study of the P300 and cerebral maps in subjects with multi-infarct dementia treated with cytidine* | V. Gallai | 1991 | Other: Non-randomised controlled trial | Multi-infarct | Dementia | Citidina |  |  | Placebo | Not enough data reported for estimation |
| 61 | 1468 | *Multicenter clinical comparison of the effects of choline alfoscerate and cytidine diphosphocholine in the treatment of multi-infarct dementia* | L. Frattola | 1991 | RCT | Multi-infarct | Dementia | Alfa-Glycerylphosphorylcholine/Choline alfoscerate |  |  | Citicoline (CDP-Choline) | Intervention compared against active treatment |
| 68 | 1661 | *A multicentre trial to evaluate the efficacy and tolerability of alpha-glycerylphosphorylcholine versus cytosine diphosphocholine in patients with vascular dementia* | R. Di Perri | 1991 | RCT | Multi-infarct | Dementia | Alfa-Glycerylphosphorylcholine/Choline alfoscerate |  |  | Citicoline (CDP-Choline) | Intervention compared against active treatment |
| 69 | 1685 | *Comparison of sulfomucopolysaccharides and cytidine diphosphocholine in the treatment of multi-infarct dementia. A randomized double-blind test* | D. Cucinotta | 1988 | RCT | Multi-infarct | Dementia | Sulfomucopolysaccharides |  |  | Citicoline | Intervention compared against active treatment |
| 70 | 1687 | *Multicentre clinical placebo-controlled study with buflomedil in the treatment of mild dementia of vascular origin* | D. Cucinotta | 1989 | RCT | Vascular | Dementia | Buflomedil then buflomedil then buflomedil | Buflomedil then buflomedil then no treatment | No treatment then buflomedil then buflomedil | No treatment then Buflomedil then no treatment | Intervention compared against active treatment |
| 71 | 1696 | *Clinical and biochemical responses to therapy in Alzheimer's disease and multi-infarct dementia* | G. L. Corona | 1989 | RCT | Multi-infarct | Dementia | Citicoline (CDP-Choline) or Piracetam |  |  | Citicoline (CDP-Choline) Piracetam | Intervention compared against active treatment |
| 73 | 1759 | *Effects of butylphthalide combined with naofukang on cognitive function and the expression of serum neurotrophic factor in patients with vascular dementia* | Y. Chen | 2020 | RCT | Vascular | Dementia | Butylphtalide Piracetam |  |  | Piracetam | Intervention compared against active treatment |
| 74 | 1832 | *The effects of bromvincamine and vincamine on regional cerebral blood flow and mental functions in patients with multi-infarct dementia* | S. Hagstadius | 1984 | RCT | Multi-infarct | Dementia | Brovincamine | Vincamine |  | Placebo | Not enough data reported for estimation |
| 77 | 1856 | *Naftidrofuryl in the treatment of mild senile dementia. A double-blind study* | W. M. Grossmann | 1990 | RCT | Multi-infarct | Dementia | Naftidrofuryl |  |  | Placebo | Not enough data reported for estimation |
| 80 | 1968 | *Idebenone in the treatment of multi-infarct dementia: A randomised, double-blind, placebo controlled multicentre trial* | B. Bergamasco | 1992 | RCT | Multi-infarct | Dementia | Idebenone |  |  | Placebo | Not enough data reported for estimation |
| 87 | 2252 | Effects of candesartan on cerebral microvascular function in mild cognitive impairment: Results of two clinical trials | I. Hajjar | 2023 | RCT | Vascular | MCI | Candesartan |  |  | Lisinopril | Intervention compared against active treatment |
| 88 | 2291 | Naoxin’an capsules protect brain function and structure in patients with vascular cognitive impairment | J. Zhang | 2023 | RCT | Vascular | MCI | Naoxin’an |  |  | *Ginkgo Biloba extracts* | Intervention compared against active treatment |
| 89 | 2381 | The Effect of Guilingji Capsules on Vascular Mild Cognitive Impairment: A Randomized, Double-Blind, Controlled Trial | L. Ma | 2022 | RCT | Vascular | MCI | Guilingji |  |  | *Ginkgo Biloba extracts* | Intervention compared against active treatment |
| 90 | 2407 | Augmentation therapy with tandospirone citrate in vascular depression patients with mild cognitive impairment: A prospective randomized clinical trial | N. Liu, Y. Xiao, H. Chen | 2022 | RCT | Vascular | MCI | Tandospirone  Escitalopram |  |  | Escitalopram | Intervention compared against active treatment |
| 91 | 2534 | Modified Suanzaoren decoction in treating post-stroke cognitive impairment with comorbid insomnia symptoms: A clinical trial | M. Zhu | 2023 | RCT | Post-stroke | MCI  Dementia | Modified Suanzaoren decoction (M-SZRD) | Promazine |  | Zolpidem | Intervention compared against active treatment |
| 94 | 2562 | NEURoaid II (MLC901) in cognitively Impaired not demenTEd patientS (NEURITES): a pilot double blind, placebo-controlled randomized trial | C. L. H. Chen | 2020 | RCT | Post-stroke | MCI | MLC901(NEURoaid II) |  |  | Placebo | Not enough data reported for estimation |
| 96 | 2584 | Effects of Dengzhan Shengmai Capsule combined with butylphthalide soft capsule on oxidative stress indexes and serum Hcy and CRP levels in patients with vascular dementia | W. Sun | 2020 | RCT | Vascular | Dementia | Butylphtalide | Dengzhan Shengmai | Butylphtalide  Dengzhan Shengmai |  | Intervention compared against active treatment |
| 99 | 2612 | Clinical study on effect of Xianjong Capsule in treating senile vascular dementia | Y. Zhao | 2002 | RCT | Post-stroke | Dementia | Xialong capsule |  |  | Hydergine | Intervention compared against active treatment |
| 100 | 34 | *Electroacupuncture on the head points for improving Gnosia in patients with vascular dementia* | Z. Ling | 2009 | RCT | Vascular | Dementia | Nimodipine and ‘yi qi tiao xue, fu ben pei yuan’ (acupuncture) | Electro-acupuncture |  | Nimodipine | Intervention compared against active treatment |
| 101 | 530 | *Efficacy and safety assessment of acupuncture and nimodipine to treat mild cognitive impairment after cerebral infarction: A randomized controlled trial* | S. Wang | 2016 | RCT | Post-stroke; acute/subacute stroke | MCI | Nimodipine and acupuncture | Acupuncture |  | Nimodipine | Intervention compared against active treatment |
| 102 | 555 | *A promising approach to the treatment of multi-infarct dementia* | M. Walzl | 2000 | RCT | Multi-infarct | Dementia | Pentoxifylline  & Heparin-induced extracorporeal LDL/fibrinogen precipitation (HELP) |  |  | Pentoxifylline | Intervention compared against active treatment |
| 103 | 867 | *Neuroprotection against vascular dementia after acupuncture combined with donepezil hydrochloride: P300 event related potential* | Q. Liu | 2016 | RCT | Vascular | Dementia | Donepezil and acupuncture |  |  | Donepezil | Intervention compared against active treatment |
| 104 | 2328 | Effect of acupuncture on cerebral blood flow, serum S100β and ALP in the patients with post-stroke mild cognitive impairment in the convalescence stage | W. Shi | 2022 | RCT | Post-stroke | MCI | Acupunture  Oxiracetam |  |  | Oxiracetam | Intervention compared against active treatment |
| 105 | 1775 | *Clinical research on comprehensive treatment of senile vascular dementia* | L. Chen | 2011 | RCT | Vascular | Dementia | Chinese medicine combinations and cognitive rehabilitation | Chinese medicine combinations and acupuncture | Chinese medicine combinations and a cupuncture amd cognitive rehabilitation | Piracetam | Intervention compared against active treatment |
| 106 | 167 | *Effects of acupuncture on Chinese medicine syndromes of vascular dementia* | G. Shi | 2014 | RCT | Vascular | Dementia | Acupuncture |  |  | Cognitive rehabilitation | Intervention compared against active treatment |
| 107 | 169 | *Acupuncture for Vascular Dementia: A Pragmatic Randomized Clinical Trial* | G. Shi | 2015 | RCT | Vascular | Dementia | Acupuncture |  |  | Best medical treatment | Not enough data reported for estimation |
| 108 | 449 | *Effect of acupuncture treatment on vascular dementia* | J. Yu | 2006 | RCT | Vascular | Dementia | ‘yi qi tiao xue, fu ben pei yuan’ (acupuncture) |  |  | Regular acupuncture | Intervention compared against active treatment |
| 109 | 457 | *Effects of rTMS Treatment on Cognitive Impairment and Resting-State Brain Activity in Stroke Patients: A Randomized Clinical Trial* | M. Yin | 2020 | RCT | Post-stroke; acute/subacute stroke | MCI | Transcranial Magnetic Stimulation (TMS) and cognitive rehabilitation |  |  | Cognitive rehabilitation | Intervention compared against active treatment |
| 110 | 469 | *Effectiveness of acupuncture for vascular cognitive impairment no dementia: a randomized controlled trial* | JW Yang | 2019 | RCT | Vascular | MCI | Acupuncture |  |  | Citicoline (CDP-Choline) | Intervention compared against active treatment |
| 111 | 488 | *Protective Effect of Hyperbaric Oxygen Therapy on Cognitive Function in Patients with Vascular Dementia* | Y. Xu | 2019 | RCT trial | Vascular | Dementia | Hyperbaric oxygen Donepezil |  |  | Donepezil | Intervention compared against active treatment |
| 115 | 1033 | *A comparative study on the acupoints of specialty of Baihui, Shuigou and Shenmen in treating vascular dementia* | X. Lai | 2005 | RCT | Post-stroke | Dementia | Acupuncture |  |  |  | Intervention compared against active treatment |
| 116 | 1183 | *Comparative study of the specificities of needling acupoints DU20, DU26 and HT7 in intervening vascular dementia in different areas in the brain on the basis of scale assessment and cerebral functional imaging* | H. Yong | 2007 | RCT | Vascular | Dementia | Acupuncture |  |  | Acupuncture | Intervention compared against active treatment |
| 117 | 1270 | *Randomized, dim light controlled, crossover test of morning bright light therapy for rest-activity rhythm disorders in patients with vascular dementia and dementia of alzheimer's type* | K. Mishima | 1998 | RCT | Vascular | Dementia | Light Therapy |  |  | Light Therapy | Intervention compared against active treatment |
| 118 | 1870 | *The effects of light therapy on mini-mental state examination scores in demented patients* | A. Graf | 2001 | RCT | Vascular | Dementia | Light Therapy |  |  | Light Therapy | Intervention compared against active treatment |
| 120 | 2329 | Clinical observation of taking acupuncture at the acupoints based on "four seas theory" for post-stroke cognitive impairment 基于“四海理论”组穴针刺治疗脑卒中后认知障碍的临床观察 | J. Yang | 2022 | RCT | Post-stroke | MCI  Dementia | Acupuncture  Cognitive rehabilitation |  |  | Cognitive rehabilitation | Intervention compared against active treatment |
| 121 | 2337 | Efficacy of Intermittent Theta-Burst Stimulation and Transcranial Direct Current Stimulation in Treatment of Post-Stroke Cognitive Impairment | J. Chen | 2022 | RCT | Post-stroke | MCI  Dementia | Intermittent Theta Burst Stimulation  Computerised cognitive rehabilitation | tDCS  Computerised cognitive rehabilitation |  | Computerised cognitive rehabilitation | Intervention compared against active treatment |
| 123 | 2549 | Impact of transcranial direct current stimulation combined with motor-cognitive intervention on post-stroke cognitive impairment | L. Zhang | 2023 | RCT | Post-stroke | MCI  Dementia | tDCS | Cognitive Rehabilitation  Physiotherapy | tDCS  Cognitive Rehabilitation  Physiotherapy |  | Intervention compared against active treatment |
| 125 | 2574 | Non-invasive cortical stimulation improves post-stroke attention decline | N. Paik | 2009 | RCT | Post-stroke | MCI  Dementia | tDCS |  |  | Placebo | Not enough data reported for estimation |
| 127 | 2608 | Cerebral activity manipulation of low-frequency repetitive transcranial magnetic stimulation in post-stroke patients with cognitive impairment | W. Shiyan | 2022 | RCT | Post-stroke | MCI  Dementia | rTMS |  |  | Placebo | Not enough data reported for estimation |
| 128 | 909 | *The effect of combined scalp acupuncture and cognitive training in patients with stroke on cognitive and motor functions* | J. Xiong | 2020 | RCT | Post-stroke; acute/subacute stroke | MCI | Acupuncture and cognitive rehabilitation |  |  | Placebo and cognitive rehabilitation | Intervention compared against active treatment |
| 129 | 2344 | The effects and mechanisms of transcranial ultrasound stimulation combined with cognitive rehabilitation on post-stroke cognitive impairment | S. Chen | 2022 | RCT | Post-stroke | MCI | Transcranial ultrasonic stimulation (TUS)  Cognitive rehabilitation |  |  | Cognitive rehabilitation  Sham TUS | Intervention compared against active treatment |
| 130 | 2369 | Effect of Interactive Dynamic Scalp Acupuncture on Post-Stroke Cognitive Function, Depression, and Anxiety: A Multicenter, Randomized, Controlled Trial | Y. Wang | 2022 | RCT | Post-stroke | MCI  Dementia | Dynamic Scalp Acupuncture  Computerised Cognitive Rehabilitation | Acupuncture (morning) + Computerised Cognitive Rehabilitation (afternoon) | Acupuncture |  | Intervention compared against active treatment |
| 131 | 2539 | Effects of combined use of intermittent theta burst stimulation and cognitive training on post-stroke cognitive impairment : a single-blind randomized controlled trial | J. Chen | 2022 | RCT | Post-stroke | MCI  Dementia | Intermittent Theta-burst stimulation |  |  | Computerised Cognitive Rehabilitation | Intervention compared against active treatment |
| 132 | 2576 | Home-Based Transcranial Direct Current Stimulation to Enhance Cognition in Stroke: randomized Controlled Trial | Y. Kim | 2022 | RCT | Post-stroke | MCI  Dementia | tDCS  Computerised cognitive rehabilitation |  |  | Computerised cognitive rehabilitation | Intervention compared against active treatment |
| 136 | 789 | *Adaptive conjunctive cognitive training (ACCT) in virtual reality for chronic stroke patients: a randomized controlled pilot trial* | M. Maier | 2020 | RCT | Post-stroke | MCI; dementia | Computerized cognitive rehabilitation |  |  | Cognitive rehabilitation | Intervention compared against active treatment |
| 140 | 2289 | Application of Immersive Virtual-Reality-Based Puzzle Games in Elderly Patients with Post-Stroke Cognitive Impairment: A Pilot Study | M. Chen J. Jia | 2022 | RCT | Post-stroke | MCI  Dementia | Computerised Cognitive Rehabilitation |  |  | Standard Cognitive Rehabilitation | Intervention compared against active treatment |
| 141 | 2362 | Effects of a Combined Motor Imagery and Action Observation Intervention on Vascular Cognitive Impairment: A Randomized Pilot Study | A. Dong | 2022 | RCT | Vascular | MCI  Dementia | Cognitive rehabilitation | Motor imagery with cues | Cognitive rehabilitation Motor imagery with cues |  | Intervention compared against active treatment |
| 144 | 2599 | Effects of dual-task training in patients with post-stroke cognitive impairment: a randomized controlled trial | W. Gong | 2022 | RCT | Post-stroke | MCI  Dementia | Cognitive-motor dual task training |  |  | Standard cognitive rehabilitation | Intervention compared against active treatment |
| 146 | 3041 | *Based on fNIRS Technology: The Effects of Scalp Acupuncture Combined with iTBS on Cognitive Function After Stroke* | S. Lan | 2025 | RCT | Post-stroke | MCI | Acupuncture | Intermittent Theta Burst Stimulation | Acupuncture  Intermittent Theta Burst Stimulation |  | Intervention compared against active treatment |
| 147 | 3051 | *Effect of Stellate Ganglion Block on Dysphagia and Cognitive Impairment in Cerebral Small Vessel Disease: A Randomized Controlled Study* | X. Zeng | 2024 | RCT | Subcortical vascular | MCI Dementia | Stellate Ganglion block |  |  | Best medical treatment | Not enough data reported for estimation |
| 150 | 3085 | *A randomized controlled trial of repetitive transcranial magnetic stimulation plus donepezil vs donepezil alone for mild to moderate cognitive impairment due to small vessel cerebrovascular disease* | X. Chen | 2024 | RCT | Subcortical vascular | MCI Dementia | Donepezil Left DLPFC rTMS |  |  | Donepezil | Intervention compared against active treatment |
| 151 | 3102 | *Galantamine combined with cognitive rehabilitation on post-stroke cognitive impairment: a proof-of-concept study* | M. Planton | 2025 | RCT | Post-stroke | MCI | Galantamine Cognitive rehabilitation |  |  | Cognitive rehabilitation | Intervention compared against active treatment |
| 152 | 3110 | *Efficacy of Cognitive Stimulation Therapy for Cognition in Patients with Vascular Cognitive Impairment: A Pilot Randomized Controlled Trial* | Y. Otaka | 2024 | RCT | Post-stroke; acute/subacute stroke | MCI Dementia | Cognitive stimulation Therapy Conventional rehabilitation (cognitive + motor) |  |  | Conventional rehabilitation (cognitive + motor) | Intervention compared against active treatment |
| 153 | 3124 | *Electroacupuncture improves vascular cognitive impairment no dementia: A Randomized Clinical Trial* | W. Liu | 2024 | RCT | Vascular | MCI | Electroacupuncture |  |  | Best medical treatment | Not enough data reported for estimation |
| 154 | 3133 | *Effect of Repetitive Transcranial Magnetic Stimulation on Post-Stroke Comorbid Cognitive Impairment and Depression: A Randomized Controlled Trial* | R. Feng | 2024 | RCT | Post-stroke; acute/subacute stroke | MCI  Dementia | Sertraline rTMS Acupuncture |  |  | Acupuncture | Intervention compared against active treatment |
| 155 | 3154 | *The effects of moderate-intensity aerobic exercise on cognitive function in individuals with stroke-induced mild cognitive impairment: a randomized controlled pilot study* | H. Ou | 2024 | RCT | Post-stroke | MCI | Aerobic Exercise Motor rehabilitation Occupational Therapy Acupuncture |  |  | Motor rehabilitation  Occupational Therapy  Acupuncture | Intervention compared against active treatment |
| 158 | 3184 | *Computer-aided cognitive training combined with tDCS can improve post-stroke cognitive impairment and cerebral vasomotor function: a randomized controlled trial* | Y. Qu | 2024 | RCT | Post-stroke | MCI  Dementia | tDCS Computerised cognitive rehabilitation | Computerised cognitive rehabilitation | tDCS | Cognitive rehabilitation | Intervention compared against active treatment |
| 160 | 4765 | *Activation changes in patients with post-stroke cognitive impairment receiving intermittent theta burst stimulation: A functional near-infrared spectroscopy study* | J. Chen | 2024 | RCT | Post-stroke | MCI  Dementia | iTBS Cognitive rehabilitation |  |  | Cognitive rehabilitation | Intervention compared against active treatment |
| 162 | 4898 | *Electroacupuncture combined with cognitive rehabilitation outperforms cognitive rehabilitation alone in treating post-stroke cognitive impairment: a randomized controlled trial* | J. Yao | 2025 | RCT | Post-stroke | MCI  Dementia | Electroacupuncture Cognitive rehabilitation |  |  | Cognitive rehabilitation | Intervention compared against active treatment |
| 165 | 5584 | *Effects of repetitive transcranial magnetic stimulation on cognitive function and hormone levels in early stroke patients with low thyroid hormone levels* | J. Ma | 2024 | RCT | Post-stroke | MCI | rTMS  Cognitive rehabilitation |  |  | Cognitive rehabilitation | Intervention compared against active treatment |
| 166 | 5593 | *Clinical Effect of Shibing Xingnao Granules on Vascular Dementia Patients and Its Effect on Serum Neuronal Apoptosis Molecules* | P. Li | 2023 | RCT | Post-stroke | Dementia | Shibing Xingnao granules  Acupuncture |  |  | Acupuncture | Intervention compared against active treatment |
| 167 | 5655 | *Efficacy and safety of high-dose and personalized TBS on post-stroke cognitive impairment: A randomized controlled trial* | H. Zhang | 2025 | RCT | Post-stroke | MCI  Dementia | iTBS Computerised cognitive rehabilitation | iTBS Computerised cognitive rehabilitation |  | Sham treatment Computerised cognitive rehabilitation | Intervention compared against active treatment |
| 169 | 5719 | *Low frequency-repetitive transcranial magnetic stimulation combined with Xingnao Kaiqiao acupuncture improves post-stroke cognitive impairment and has better clinical efficacy* | D. Li | 2024 | Non-randomised experimental study | Post-stroke | MCI  Dementia | Acupuncture rTMS |  |  | rTMS | Intervention compared against active treatment |
| 170 | 5723 | *Intermittent theta burst stimulation combined with cognitive training improves cognitive dysfunction and physical dysfunction in patients with post-stroke cognitive impairment* | X. Shu and T. Zhou | 2024 | Non-randomised experimental study | Post-stroke | MCI  Dementia | iTBS Cognitive rehabilitation |  |  | Sham treatment Cognitive rehabilitation | Intervention compared against active treatment |
| 171 | 5730 | *Effect of combined use of Buyang Huanwu decoction and olanzapine on clinical symptoms, neurological function, and degree of dementia in patients with vascular dementia after cerebral ischemic stroke* | N. Chen | 2023 | Non-randomised experimental study | Post-stroke | Dementia | Buyang Huanwu decoction Olanzapine |  |  | Olanzapine | Intervention compared against active treatment |

Notes: *Single neuropsychological tests have been aggregated according to the cognitive domains tested (either according to the specifications reported in the study or, if lacking, to test description and classification according to standard neuropsychological toolkits). For studies reporting test spanning more than one domain (n), neuropsychological outcomes are reported as follows: "neuropsychological tests (n): domains tested".

*Abbreviations*: *BPSD*, behavioral and psychological symptoms of dementia; *RCT, Randomised Controlled Trial*.
