# Supplementary material for: Therapeutic strategies in vascular cognitive impairment: A systematic review and meta‐analysis
Source: Alzheimers Dement. 2025 Nov 6;21(11):e70840. doi: 10.1002/alz.70840 (PMC12591988; doi:10.1002/alz.70840)
Supplement: Supplementary file 6 — Supporting Information [file ALZ-21-e70840-s007.pdf]

SUPPLEMENTARY MATERIALS

Therapeutic strategies in Vascular Cognitive Impairment: a systematic review and meta-analysis

Federico Masserini MD[1,3], Claudia Gendarini MD[1,2], Giacomo Baso MD[1,2], Emilia Salvadori PhD[1], Leonardo Pantoni MD, PhD[1,4]

Affiliations

[1]Neuroscience Research Center, Department of Biomedical and Clinical Sciences, University of Milan – Via Giovanni Battista Grassi 74, 20157, Milan, Italy

[2]Neurology Residency Program, University of Milan – Via Francesco Sforza 35, 20122, Milan, Italy

[3]Department of Radiology and Nuclear Medicine, Amsterdam University Medical Centre, Vrije Universiteit, Amsterdam Neuroscience – De Boelelaan 1117, 1081 HV Amsterdam, the Netherlands

[4]Department of Neurorehabilitation Sciences, Casa di Cura Igea – Via G. Dezza 48, 20144, Milan, Italy

Corresponding author:

Leonardo Pantoni, MD, PhD

Neuroscience Research Center

Department of Biomedical and Clinical Sciences, University of Milan

Via Giovanni Battista Grassi 74, 20157 Milano, Italy.

E-mail address: leonardo.pantoni@unimi.it

ORCID: 0000-0001-7357-8530

TABLE OF CONTENTS

1. SUPPLEMENTARY METHODS 6

1.1 Extended Systematic Review methodology 6
1.1.1 Systematic Review 6

1.1.2 Meta-analysis 9

1.2 Complete Research Strings 13
1.2.1 Pubmed/Medline 13
1.2.2 Embase 13
1.2.3 CENTRAL (Cochrane Library) 13

2A. SUPPLEMENTARY RESULTS: DESCRIPTIVE REVIEW OF STUDIES CHARACTERISTICS 14

2A.1 Characteristics of included population(s) according to major nosological entities 15

2A.2 Patient enrolment criteria 15

2A.3 Investigated interventions 16
2A.3-1 Intervention duration and follow-up time 16

2A.4 Comparators 17

2A.5 Outcomes 17

2A.6 Study quality assessment 17

2A.7 Overall intervention efficacy rating 18

2A.8 Supplementary Results Tables 19
eTable 1: VCI labels stratified by degree of cognitive impairment used in included interventional studies. 19

2A.9 Supplementary Results Figures 20
eFigure 1: Time trend of the use of classes of criteria for patient enrolment 20
eFigure 2: Study size distribution 21
eFigure 3: Distribution of study size, colour-coded according to intervention class, over time 22
eFigure 4: VCI label use over time 23
eFigure 5: Number of studies evaluating each intervention class along with their comparator 24
eFigure 6: Most frequently reported intervention and intervention classes 25
eFigure 7: Outcome and outcome classes reported most frequently 26
eFigure 8: Distribution of intervention duration 27
eFigure 9: Main global cognitive efficiency outcomes employed throughout the years 28
eFigure 10: Different intervention category tested throughout the years 29
eFigure 11: Overall rated efficacy of studies 30
eFigure 12: Study global quality ratings and item-specific risk of bias according to NIH-QAT 31

eFigure 13: Overall study quality assessment score for studies stratified according to year of publication 32

2B. SUPPLEMENTARY RESULTS: QUALITATIVELY SUMMARIZED INTERVENTIONS 33

2B.1 PHARMACOLOGICAL INTERVENTIONS 33

2B.2 REHABILITATIVE STRATEGIES 33

2B.3 NON-REHABILITATIVE NON-PHARMACOLOGICAL (PHYSICAL DEVICE APPLICATION) INTERVENTIONS 33

2B.4 OTHER INTERVENTIONS 34

2C. SUPPLEMENTARY RESULTS: META-ANALYSES 35

2C.1 Interventions candidate to meta-analysis 35

2C.2 Gingko Biloba 37
2C.2-1 Description of studies and meta-analysis main results 37
2C.2-2 Characteristic of studies 38
2C.2-3 Summary of findings and figures for meta-analyses 41
2C.2-4 Sensitivity Analyses 47

2C.3 AcetilCholinesterase Inhibitors – Rivastigmine 51
2C.3-1 Description of studies and meta-analysis main results 51
2C.3-2 Characteristic of studies 53
2C.3-3 Summary of findings and figures for meta-analyses 56
2C.3-4 Sensitivity Analyses 61

2C.4 AcetilCholinesterase Inhibitors – Galantamine 65
2C.4-1 Description of studies and meta-analysis main results 65
2C.4-2 Characteristic of studies 67
2C.4-3 Summary of findings and figures for meta-analyses 70

2C.5 AcetilCholinesterase Inhibitors – Donepezil 75
2C.5-1 Description of studies and meta-analysis main results 75
2C.5-2 Characteristic of studies 76
2C.5-3 Summary of findings and figures for meta-analyses 79
2C.5-4 Sensitivity Analyses 98

2C.6 Memantine 103
2C.6-1 Description of studies and meta-analysis main results 103
2C.6-2 Characteristic of studies 104
2C.6-3 Summary of findings and figures for meta-analyses 106
2C.6-4 Sensitivity Analyses 111

2C.7 Pentoxifylline 114
2C.7-1 Description of studies and meta-analysis main results 114
2C.7-2 Characteristic of studies 115
2C.7-3 Summary of findings and figures for meta-analyses 117
2C.7-4 Sensitivity Analyses 121

2C.8 Propentofylline **123**
2C.8-1 Description of studies and meta-analysis main results 123
2C.8-2 Characteristic of studies 124
2C.8-3 Summary of findings and figures for meta-analyses 126

2C.9 Cerebrolysin **129**
2C.9-1 Description of studies and meta-analysis main results 129
2C.9-2 Characteristic of studies 130
2C.9-3 Summary of findings and figures for meta-analyses 132
2C.9-4 Sensitivity Analyses 135

2C.10 Nimodipine **138**
2C.10-1 Description of studies and meta-analysis main results 138
2C.10-2 Characteristic of studies included in the meta-analysis 140
2C.10-3 Summary of findings and figures for meta-analyses 143
2C.10-4 Sensitivity Analyses 154

2C.11 Remote ischemic conditioning **158**
2C.11-1 Description of studies and meta-analysis main results 158
2C.11-2 Characteristic of studies considered for meta-analysis 159
2C.11-3 Summary of findings and figures for meta-analyses 161
2C.11-4 Sensitivity Analyses 164

2C.12 Cognitive Training **166**
2C.12-1 Description of studies and meta-analysis main results 166
2C.12-2 Characteristic of studies 167
2C.12-3 Summary of findings and figures for meta-analysis 170
2C.12-4 Sensitivity Analyses 173

2C.13 Repetitive Transcranial Magnetic Stimulation **175**
2C.13-1 Description of studies and meta-analysis main results 175
2C.13-2 Characteristic of studies considered for meta-analysis 176
2C.13-3 Summary of findings and figures for meta-analyses 180

2C.14 Transcranial Direct Current Stimulation **183**
2C.14-1 Description of studies and meta-analysis main results 183
2C.14-2 Characteristic of studies considered for meta-analysis 184
2C.14-3 Summary of findings and figures for meta-analyses 187
2C.14-4 Sensitivity Analyses 190

2C.15 Physical Exercise **192**
2C.15-1 Description of studies and meta-analysis main results 192
2C.15-2 Characteristic of studies considered for meta-analysis 193
2C.15-3 Summary of findings and figures for meta-analyses 196
2C.15-4 Sensitivity Analyses 199

2C.16 All-treatment meta-analysis on safety outcomes **202**

3. SUPPLEMENTARY TABLES 203

3.1 All treatments (not included in meta-analyses) in alphabetical order **203**
3.1.1 *eTable 2* – Qualitative summary of study characteristics and efficacy of pharmacological interventions 203

3.1.2 *eTable 3* - Qualitative summary of study characteristics and efficacy of rehabilitative interventions 219
3.1.3 *eTable 4* - Qualitative summary of study characteristics and efficacy of non-rehabilitative non-pharmacological interventions 222
3.1.4 *eTable 5* – Qualitative summary of study characteristics and efficacy of other interventions 233

3.2 Interventions by VCI label 234
3.2.1 *eTable 6*: Interventions, not included in meta-analyses, evaluated in post-stroke cognitive impairment (including acute/subacute stroke and multi-infarct dementia) 234
3.2.2 *eTable 7* - Interventions, not included in meta-analyses, evaluated in subcortical ischemic (SVD-related) cognitive impairment 252

4. SUPPLEMENTARY FILES 255

Supplementary File 1: List of Cochrane Reviews retrieved and screened by systematic search 255

Supplementary File 2: List of excluded full texts 255

Supplementary File 3: List of all included studies 255

Supplementary File 4: Quality assessment according to NIH-QAT for controlled studies 255

Supplementary File 5: List of studies not included in semi-quantitative estimation 255

5. SUPPLEMENTARY BIBLIOGRAPHY 256

1. SUPPLEMENTARY METHODS

1.1 Extended Systematic Review methodology

1.1.1 Systematic Review

This work was performed according to the Prisma Guidelines for Systematic Reviews[1] and was registered on PROSPERO (CRD: 4202127093). We conducted a systematic review to identify all randomised and non-randomised clinical trials investigating therapeutic interventions for vascular cognitive impairment (VCI).

Search strategy

We searched for any interventional study that: 1) enrolled patients with any degree of cognitive impairment due to any kinds of vascular substrate; 2) tested any intervention either versus placebo or versus any other treatment, evaluated according to any type of outcome. We excluded prevention studies (i.e., studies enrolling subjects with a cerebrovascular condition but who were not cognitively impaired at baseline), studies not including human subjects, studies that included also patients with other types of dementia (e.g., Alzheimer's disease, AD) and did not report results for VCI patients separately (i.e., studies on patients with "mixed dementia" are excluded). Moreover, we excluded studies not published in English.

We searched three databases: Medline® (PubMed, available at www.pubmed.ncbi.nlm.nih.gov), Embase® (available at www.embase.com), and CENTRAL (available at www.cochranelibrary.com) from inception to 23 June 2025. We employed combinations of keywords structured into complex search strings tailored to each database syntax (reported below, *Supplementary Method section 1.2*): 'vascular', 'small vessel', 'small vessel disease', 'post-stroke', 'multi-infarct', 'subcortical vascular', 'subcortical ischemic', 'subcortical ischemic vascular', 'cognitive impairment', 'mild cognitive impairment', 'dementia', 'VAD', 'therapy', 'management', 'prevent', 'clinical trial', 'meta-analysis', 'randomized controlled trial'.

Study Selection

Search results were uploaded to Covidence systematic review software (Veritas Health Innovation, Melbourne, Australia; available at www.covidence.org). Duplicate entries were automatically identified and removed before screening began. Two reviewers, randomly assigned from a pool of three (CG, GB, and FM), independently assessed abstracts of the retrieved entries for adherence to inclusion/exclusion criteria. Full texts of relevant abstracts were retrieved. Studies for which the full text was unavailable (online, printed, or after contacting the corresponding author) were excluded. Finally, reviewers working in randomised pairs independently assessed the retrieved full-text reports for adherence, extracted data from included reports, and collected them in an electronic database. Disagreements at any stage were resolved by consensus with a fourth expert investigator (LP). All logs pertaining to each search step were documented.

Data extraction

The following data were extracted:

- Study details: title, lead author, country, publication year, study aim, design, start and end years of the trial.
- Participant details: degree of cognitive impairment, VCI subtype, inclusion/exclusion criteria, presence of other dementias, total number of patients.
- Intervention details: intervention class (pharmacological, rehabilitative, non-pharmacological non-rehabilitative aka physical-device application-requiring, other), specific intervention, dosage/details of intervention, treatment duration, comparator employed (placebo or other intervention).
- Baseline participant demographic characteristics: overall and for each arm (at least: total participants for each arm, age, gender distribution, education level).
- Outcomes: assessed outcomes and corresponding data, collected at different study time points, classified into four categories: cognitive outcomes (i.e., tests specifically measuring cognitive abilities, as cognitive screening test, e.g. MoCA, cognitive scales, e.g. ADAS-CoG, or neuropsychological tests), functional outcomes (i.e., non-strictly cognitive tests measuring real

world abilities and preservation of autonomy in daily living including hard outcomes, e.g., ADL, IADL, modified Barthel Index), instrumental outcomes (i.e., outcomes related to measurements of biological markers of disease, e.g. MR markers, blood values etc), and patient-centred outcomes (i.e., outcomes that reflect aspects of care and well-being that are most meaningful to patient themselves, e.g. quality of life scales, symptom burden).

Possible degrees of cognitive impairment included "dementia," "mild cognitive impairment", and "cognitive impairment" (assigned when unspecified in the report). Considered vascular aetiology labels were "multi-infarct," "post-stroke", "acute/subacute stroke", "subcortical vascular", "small vessel disease", and "vascular" (used when vascular aetiology was defined but the subtype was not specified).

For each study, we extracted a maximum of seven outcomes, prioritising primary outcomes and then selecting additional outcomes (favouring cognitive ones if more than seven were present). To enhance result homogeneity, cognitive outcomes were extracted as a single outcome for global cognitive efficiency assessments (e.g., MMSE, MoCA) and as an aggregated outcome for multiple tests measuring the same cognitive domain (e.g., memory).

Studies were considered as controlled against inactive treatment (either “placebo” or “sham treatment” as well as best medical treatment/care) only when no other kind of background interventions (pharmacological, rehabilitative, or other) – outside what can be considered the standard bundle of care – were reported in the study control arm. As possible synergies between different treatment strategies are difficult to ascertain *a priori*, all studies have been considered as controlled against another intervention when other treatments beyond inactive treatment strategies are reported in the comparator group.

Efficacy Assessment

During data extraction, the efficacy of the intervention was categorised into four tiers: "*in favour of treatment*", "*partially in favour of treatment*", "*neutral*" and "*not in favour of treatment*". These categories were assigned based on the numerical results reported in each study for the relevant outcomes, as rated independently by the two raters assigned in randomised pairs.

A trial could be classified as "*in favour of treatment*" only when the majority of primary outcomes (if specified) or the majority of all outcomes (if no primary outcomes were defined) demonstrated a positive treatment effect. Importantly, other outcomes should either be neutral, showing neither benefit nor harm, or not support the intervention. A study could be categorised as "*partially in favour of treatment*" if the majority of primary outcomes were neutral while at the same time the majority of secondary outcomes exhibited positive effects, indicating a potential benefit of the intervention. A study was categorised as "*neutral*" when the majority of outcomes were neutral, showing neither a positive nor a negative impact of the treatment. Finally, a study was categorised as "*not in favour of treatment*" if the majority of primary outcomes, or the majority of all outcomes, demonstrated a negative treatment effect.

Three reviewers working independently in the same randomised pairs used for data extraction assessed the quality of each included study. The assessment tool employed was the National Institute of Health Quality Assessment Tool for Controlled Intervention Studies (NIH-QAT) (available at www.nhlbi.nih.gov/health-topics/study-quality-assessment-tools; last updated July 2021). Any disagreements were resolved with the help of the fourth expert reviewer. The tool comprises fourteen questions (e.g., "Was the study described as randomised, a randomised trial, a randomised clinical trial, or an RCT?"). Answer options for each question included "Yes", "No", "Cannot determine (CD)", "Not assessable (NA)", or "Not relevant (NR)". For analysis purposes, these answers were re-categorised into three groups: low risk of bias ("Yes"), unclear risk of bias ("CD" or "NR"), and high risk of bias ("NA" and "No").

Data synthesis and quantitative analysis were performed on the extracted data. Descriptive analyses for the reported variables were then conducted using IBM SPSS Statistics software (version 29.0).

1.1.2 Meta-analysis

Single interventions were considered suitable for meta-analysis if they were assessed in at least three studies. These studies must have been designed to administer the target intervention as monotherapy in at least one arm and tested the intervention against inactive treatment, which could include placebo, no

treatment, or best medical treatment. Additionally, these studies were required to provide sufficient data for meta-analysis.

Interventions deemed unsuitable for meta-analysis were subjected to narrative synthesis, as described previously, and the reasons for their exclusion from quantitative synthesis were reported in detail. For studies not included into meta-analyses, as not fitting the criteria mentioned above, but reporting effect of intervention in monotherapy against inactive treatment, we also provided, for illustrative purposes only, a point estimate of Cohen's *d* effect size for global cognitive efficiency metrics.

For each intervention candidate selected for meta-analysis, studies were included in the final analysis if they met the following criteria:

- The study investigated the target intervention with similar treatment parameters, specifically with a similar dosage and duration, allowing for a 33% tolerance limit from the median dosage and duration.
- The study was deemed to be at low-to-moderate risk of bias, with a quality rating of "fair" or higher according to the NIH-QAT.

The impact of excluding studies from the final meta-analysis at this stage was further evaluated through *ad hoc* sensitivity analyses.

For each candidate intervention, meta-analysis was planned for the following outcome classes: global cognitive function metrics, functional parameters, patient-centred outcomes (including quality of life, self-reported measures of change, and auto-perception), and treatment safety (including adverse events and severe adverse events). Where feasible, meta-analysis was preferably conducted on a single, consistent outcome within each class.

If a specific outcome was reported by only one study included in the final meta-analysis, the data from that study were still incorporated into the summary of findings table to ensure all available evidence for the treatment target was presented. Furthermore, if data on a specific outcome were reported by the majority of studies for a particular treatment, separate meta-analyses were performed and reported for that outcome.

For each meta-analysed treatment, the *Description of studies section* and *Characteristics of included studies table* in the *Supplementary Results* provide an overview of the clinical diversity and methodological variability of the included studies.

When available, meta-analyses were performed using data from intention-to-treat populations. If intention-to-treat data were unavailable, the next most robust available population was utilised, such as "opportunity-to-complete", "as-treated," and finally "per-protocol" populations.

In meta-analysing measure of change, pre-post correlation ($Corr_{Pre\text{-}post}$) is derived from the study when available. If not directly available, it is calculated for the specific outcome from the standard deviations (SD) of the measurements at baseline (SD_{Pre}), at end of treatment (SD_{Post}), and of change (SD_{change}) according to the following formula:[2]

$$Corr_{Pre-Post} = \frac{SD_{Pre}^2 + SD_{Post}^2 - SD_{Change}^2}{2 \cdot SD_{Pre} \cdot SD_{Post}}$$

The final pre-post correlation score was calculated as the average between the $Corr_{Pre\text{-}post}$ in the treatment arm and in the control arm. If, for a given outcome in a meta-analysis for the same intervention, the pre-post correlation was known and derivable, this value was used as a surrogate estimate of the pre-post correlation for all studies employing the same outcome.

When $Corr_{Pre\text{-}post}$ could not be derived due to insufficient information (eg, *no* SD_{change} *reported*), a conservative assumption of a pre-post correlation of 0.5 was made. Sensitivity analyses were then conducted by varying the coefficient within a sufficiently broad range (0-0.8) to assess the potential impact of this assumption on the results.

For studies reporting only the measure of change for the placebo and treatment groups, the effect size was calculated directly as Cohen's *d*, along with its standard error and confidence interval.

Statistical heterogeneity was assessed in each meta-analysis using the I^2 statistic. Heterogeneity was considered probably not important if I^2 was less than 40%, moderate if I^2 was between 40% and 60%, and substantial if I^2 was greater than 60%, as per established guidelines[2]. Random-effect models were used if

heterogeneity statistical heterogeneity was estimated to be ≥ 40%, while fixed effect models were used if heterogeneity was estimated to be probably not important. As no intervention was investigated in ten or more studies, constructing funnel plots to explore publication bias was not feasible.

For continuous meta-analysed outcomes, results are presented as effect sizes. Cohen's *d* was used when single outcomes differed within an outcome class, while Cohen's *d* and unstandardised mean difference were employed when the single outcome did not vary between studies within the same outcome class. Effect size magnitude was graded according to a common framework[3]. Dichotomous outcomes (e.g., *increase in WMH-volume*) are presented as odds ratio, while safety outcomes are presented as rate ratio. We report 95% confidence intervals (CIs) for all estimates.

To assess the certainty of evidence for outcome classes amenable to meta-analysis, we employed the GRADE approach, as outlined below and in the GRADE Handbook[4]. Evidence for each meta-analysed outcome was assessed and graded using a four-tier system:

- High: we are very certain that the true effect lies close to that of the estimate of the effect.
- Moderate: we are moderately certain in the effect estimate; the true effect is likely to be close to the estimate of the effect, but there is a possibility that it is substantially different.
- Low: our certainty in the effect estimate is limited; the true effect may be substantially different from the estimate of the effect.
- Very low: we have very little certainty in the effect estimate; the true effect is likely to be substantially different from the estimate of effect.

Evidence from randomised controlled trials (RCTs) was initially considered to be of high certainty. However, the certainty of evidence was downgraded by one level for serious limitations (or two levels for very serious limitations) arising from study design (risk of bias), inconsistency across studies, indirectness of evidence, and imprecision of estimates. Reasons for downgrading the certainty of evidence are reported alongside the evidence grade in each meta-analysis summary of findings table. Meta-analysis was performed with Prometa (v. 3, Internovi, 2015).

1.2 Complete Research Strings

1.2.1 Pubmed/Medline

(((("Vascular"[Title/Abstract] OR "small vessel"[Title/Abstract] OR "small vessel disease"[Title/Abstract] OR "post-stroke"[Title/Abstract] OR "multi infarct*"[Title/Abstract] OR "subcortical vascular"[Title/Abstract] OR "subcortical ischemic"[Title/Abstract] OR "subcortical ischemic vascular"[Title/Abstract]) AND ("cognitive impairment"[Title/Abstract] OR "mild cognitive impairment"[Title/Abstract] OR "dementia"[Title/Abstract])) OR (("VCI"[Title/Abstract] OR "VAD"[Title/Abstract]) NOT ("ventric*"[Title/Abstract] AND "assist*"[Title/Abstract]))) AND ("treat*"[Title/Abstract] OR "therap*"[Title/Abstract] OR "management"[Title/Abstract] OR "prevent*"[Title/Abstract])) AND (clinicaltrial[Filter] OR meta-analysis[Filter] OR randomizedcontrolledtrial[Filter] OR systematicreview[Filter])

1.2.2 Embase

((((((vascular OR 'small vessel' OR 'small vessel disease' OR 'post-stroke' OR 'multi-infarct*' OR 'subcortical vascular' OR 'subcortical ischemic' OR 'subcortical ischemic vascular') NEXT/2 ('cognitive impairment' OR 'mild cognitive impairment' OR dementia)):ab,ti) OR (('vci':ab,ti OR 'vad':ab,ti) NOT 'ventricular':ab,ti)) AND (treat*:ab,ti OR therap*:ab,ti OR manag*:ab,ti OR prevent*:ab,ti)) AND 'human'/de AND ('clinical trial'/de OR 'meta analysis'/de OR 'phase 2 clinical trial'/de OR 'phase 3 clinical trial'/de OR 'randomized controlled trial'/de OR 'systematic review'/de))

1.2.3 CENTRAL (Cochrane Library)

((((vascular OR (small vessel) OR (small vessel disease) OR (post stroke) OR (multi infarct*) OR (subcortical vascular) OR (subcortical ischemic) OR (subcortical ischemic vascular)) NEXT ((cognitive impairment) OR (mild cognitive impairment) OR (dementia))) OR (((vci) OR (vad)) NOT (ventricular))) AND ((treat*) OR (therap*) OR (manag*) OR (prevent*) OR (rehab*))):ab,ti,kw

2A. SUPPLEMENTARY RESULTS: DESCRIPTIVE REVIEW OF STUDIES CHARACTERISTICS

According to the research strategy and after Cochrane Reviews search (*n=68 Cochrane Reviews retrieved with the same keyword-based research strategy on CENTRAL were reviewed, a complete list of reviewed Cochrane publications is available in Supplementary* file 1), we recovered 5190 entries and obtained 3360 unique publications after duplicate removal. After reviewing the titles and abstracts, we retrieved 429 full-text articles. Of these, 146 were excluded because they were systematic reviews or meta-analyses; 44 were excluded as the study populations did not meet the inclusion criteria (primarily because participants with mixed dementia or Alzheimer's disease were included without distinction from those with VCI, or because the studies focused on secondary prevention, i.e. enrolling participants post-stroke to assess interventions aimed at preventing cognitive impairment); 26 were excluded as they were not interventional studies (e.g. retrospective studies, case–control studies, or studies lacking a control arm); and ten were excluded because they did not include any cognitive-related outcomes. In addition, 24 studies were excluded as they were not published in English, and six were excluded because they were study protocols. A complete report of the systematic search steps according to PRISMA Guidelines for Systematic Reviews is depicted in *Figure 1*; the complete table reporting bibliographic data of studies excluded at the full-text stage (including studies for which full-text was not retrievable) is reported in *Supplementary file 2*.

Data from 173 studies published between December 1984 and June 2025 were extracted, cumulatively including 22,347 patients; of these, 160 were randomised controlled trials, including 20,637 patients, 13 were non-randomised studies, including 1710 patients. The average number of patients per study was 129 [SD 159, median 72, range 7-974]. Distribution of study size and study size over time is depicted in *eFigure 2* and *eFigure 3*.

All studies included in this review are listed in *Supplementary file 3*. This list features bibliographic data, population, intervention, comparator, and outcomes, as well as providing a unique study code that is referenced throughout tables.

2A.1 Characteristics of included population(s) according to major nosological entities

Included patients differed both in terms of nosological entity (i.e., the vascular "substrate" underlying cognitive decline) and degree of cognitive impairment (mild cognitive impairment or dementia). Most studies included only patients with one subtype of VCI (n=98), sometimes including specific subcategories within the broader VCI label (e.g. acute/subacute stroke within the post-stroke cognitive impairment category). The "multi-infarct" label was used 28 times, "small vessel disease" label 18 times, "post-stroke" label 52 times, and acute/subacute stroke 15 times, while the "vascular" general label was employed 75 times. Nineteen studies included other dementing nosological entities (AD or mixed dementia), but they reported and analysed separately data regarding patients with VCI.

Frequency of label use evolved progressively through time to include pathophysiologically nuanced labels that replaced older, more generic, ones (e.g., from multi-infarct dementia or vascular dementia in general towards post-stroke cognitive impairment, small vessel disease-related cognitive impairment etc); the frequency of use through time of different nosological labels is reported in *eFigure 4*.

Concerning the degree of cognitive impairment, most studies (*n*=134) included a specific degree of cognitive impairment (either MCI or Dementia) while the remainder included the whole spectrum of cognitive impairment severity. One-hundred-thirty-five studies included demented patients while seventy-nine included MCI patients. *eTable 1* shows the patients distribution according to both etiological labels and degree of included cognitive impairment.

2A.2 Patient enrolment criteria

Diagnostic criteria for each of the above reported labels varied over the years. Inclusion criteria ranged from sets of criteria such as the NINDS-AIREN criteria or Diagnostic and statistical manual of mental

disorders (DSM, in its various iterations), to clinical scales such as the Hachinski Ischemic Score or various combination of measurements of cognitive decline (Mini-Mental State Examination, Montreal Cognitive Assessment, Alzheimer's Disease Assessment Scale – cognitive subscale, etc.) and neuroimaging features, and combination of all the above. The most frequently reported criteria for classification and diagnosis of vascular cognitive decline were the Hachinski Ischemic Score (*n* = 52), the DSM-based criteria (III, III-R, IV, and V; *n* = 45), and the NINDS-AIREN criteria (*n* = 40). These criteria were often combined with pre-specified score intervals on short cognitive tests, the most commonly MMSE (*n* = 80) and MoCA (*n* = 29), or with *ad hoc* formulated clinical and radiological criteria. Some degree of *ad hoc* clinical or radiological inclusion criteria was reported in the majority of studies (n = 96). Inclusion criteria categories, their combination, and evolution of their use through time is shown in *eFigure 1*.

2A.3 Investigated interventions

eFigure 5 displays studies testing different classes of interventions (and combinations thereof) along with their comparators. Overall, 91 different therapeutic strategies were evaluated, including pharmacological interventions (*n* = 61), physical devices interventions (*n* = 11), rehabilitative interventions (*n* = 18), and other (n = 1). Most studies investigated one intervention (*n* = 117), some studies investigated two interventions (n = 48), while few studies (n = 8) investigated three or more interventions (never against inactive treatment but always in head-to-head comparisons).

The most-commonly investigated pharmacological, device-related, and rehabilitative strategies are reported in *eFigure 6*.

2A.3-1 Intervention duration and follow-up time

Overall, average duration of intervention was 18.01 weeks (SD 21.01), with a minimum of one day (single-shot interventions) and a maximum of 156 weeks *(distribution represented in eFigure 8)*. Thirty-three studies (19.1 %) reported a period of follow-up after the end of intervention (mostly non-pharmacological studies). On average, follow-up time was 16.93 weeks (SD 15.4, max 52 weeks).

2A.4 Comparators

Placebo was reported as the only comparator in 71 (41.0%) studies, best medical treatment was reported in 21 (12.1%) while other interventions were employed as comparators in 81 (46.8%) studies, either alone or in combination with inactive treatment. In the latter, the three most frequently reported comparators where cognitive rehabilitation (*n* = 17), acupuncture (n = 8), citicoline (n=6), and donepezil (*n* = 6).

2A.5 Outcomes

A total of 145 different outcomes were assessed, grouped into four classes: cognitive measures (*n* = 49), functional outcomes (*n* = 40), instrumental parameters (*n* = 45), and patient-centred measures (*n* = 11). A detailed list of the most frequently employed outcomes for each of three categories, along with their absolute and relative frequency, is reported in *eFigure 7*.

Eighty-nine studies (51.4%) clearly identified one or more primary outcomes (44 identified one, 34 identified two, four identified three, while seven identified four or more). Primary outcomes belonged to the class of cognitive measures in 81.3% of cases, to the functional outcome class in 14.0% of cases, to the instrumental class in 0.7% of cases, and to the patient-centred outcome class in 0.7% of cases. Frequency of main cognitive efficiency outcomes employment throughout the years is depicted in *eFigure 9*.

2A.6 Study quality assessment

The quality of the studies included, as rated according to the NIH Quality assessment tool for controlled intervention studies is reported in *Supplementary file 4*. Overall, 75/173 studies (43.4%) were of good, 68 (39.3%) were of fair, and 30 (17.3%) of poor quality. The five highest categories at risk of bias were sample size estimation techniques (70% of studies rated as high risk of bias in this item), participant or operator blinding (52% of studies rated at high or unclear risk of bias), treatment adherence (51% of studies rated at high or unclear risk of bias), blinding of outcome assessors (48% of studies rated at high or unclear risk of bias), and randomisation methods employed (47% of studies rated at high or unclear risk of bias). Time

trend of study quality is depicted in *eFigure 12* while a bar chart depicting overall quality ratings, as well as ratings broken down by NIH quality assessment tools items, is reported in *eFigure 13*.

2A.7 Overall intervention efficacy rating

According to extracted outcomes, studies reported efficacy of assessed intervention(s) in 57% of cases and reported partial efficacy (i.e., *primary outcome not effective but most secondary outcomes effective, some outcomes effective but not the majority with other outcomes neutral, see Supplementary methods for further details*) in 29% of studies. In the remaining studies interventions were demonstrated to be either neutral or detrimental. Proportion of studies reporting efficacy, as detailed above, was depicted in *eFigure 11*.

2A.8 Supplementary Results Tables

eTable 1: **VCI labels stratified by degree of cognitive impairment used in included interventional studies.**
Legend: VCI labels stratified by degree of cognitive impairment. The number of studies including only a specific combination of label/CI severity is reported in brackets. Abbreviations: CI, cognitive impairment; MCI, mild cognitive impairment.

	Post-stroke	Multi-infarct	Acute/subacute stroke	Vascular	Subcortical vascular	Total
**Dementia**	38 (5)	28 (28)	9 (0)	57 (54)	13 (9)	145
**MCI**	47 (10)	0	15 (2)	21 (18)	9 (5)	92
**Total**	85	28	26	75	22	237

2A.9 Supplementary Results Figures

***eFigure 1:* Time trend of the use of classes of criteria for patient enrolment**

Legend: Bar chart depicting the proportion of use of classes of diagnostic criteria according to year of study publication (years are grouped in 5-year bins from 1984 to 2023. Diagnostic criteria have been grouped into 5 major classes according to frequency: NINDS-AIREN criteria, Hachinski ischemic score, Diagnostic and Statistical Manual of Mental Disorders-based criteria – including versions III, IIIR, IV and V – criteria based on cognitive tests (global cognitive efficiency tests or more in-depth evaluations), criteria based on a mix of clinical and neuroradiological parameters, and other criteria (older and newer criteria including e.g., ADDTC, VASCOG, VICCCS-2 etc)

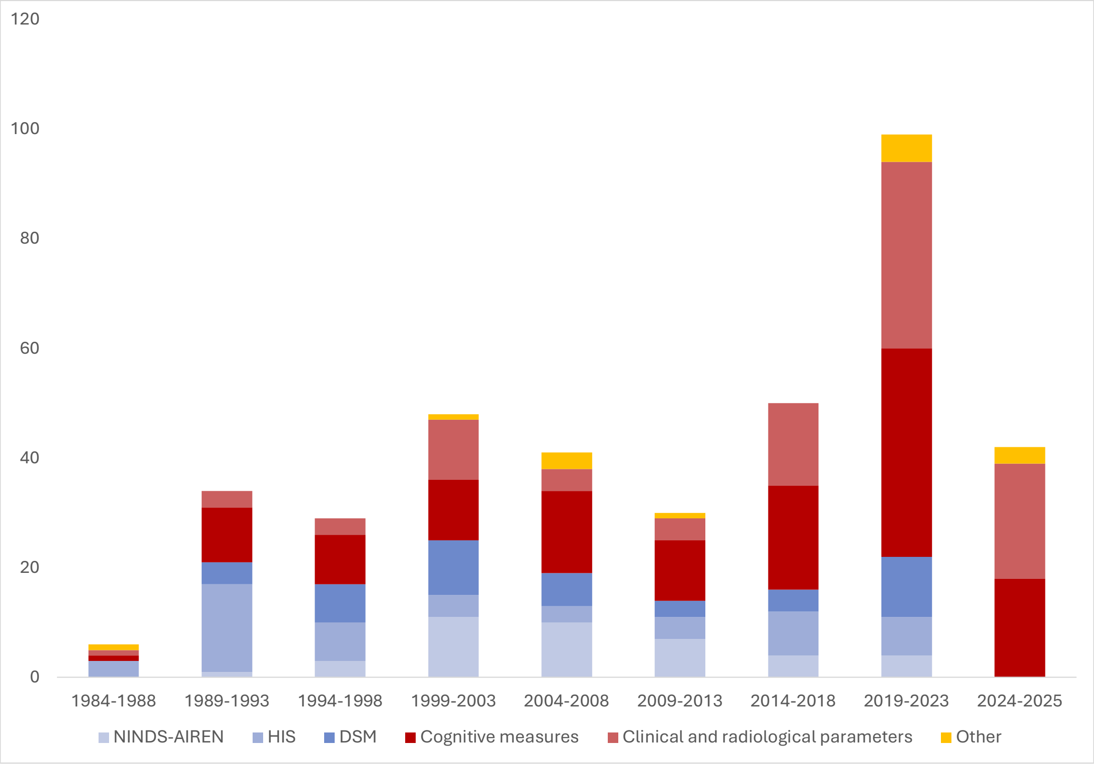

***eFigure 2:* Study size distribution**

Legend: histogram depicting the distribution of the different numbers of enrolled patients in included studies.

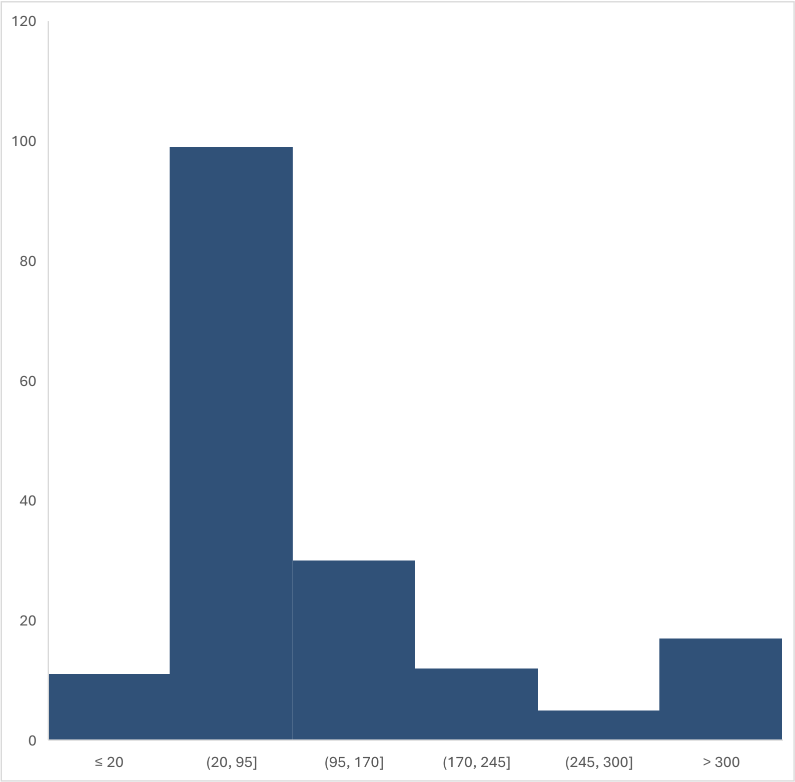

***eFigure 3:* Distribution of study size, colour-coded according to intervention class, over time**

Legend: Distribution of studies according to size, year of study publication, and class of intervention tested. Bubble plot depicting distribution of studies according to their size (patient number, bubble dimension, and Y position is proportional to study size), year of publication, and class of tested intervention tested (coded according to colour, as outlined in the legend below). The Y axis units are represented in a logarithmic scale.

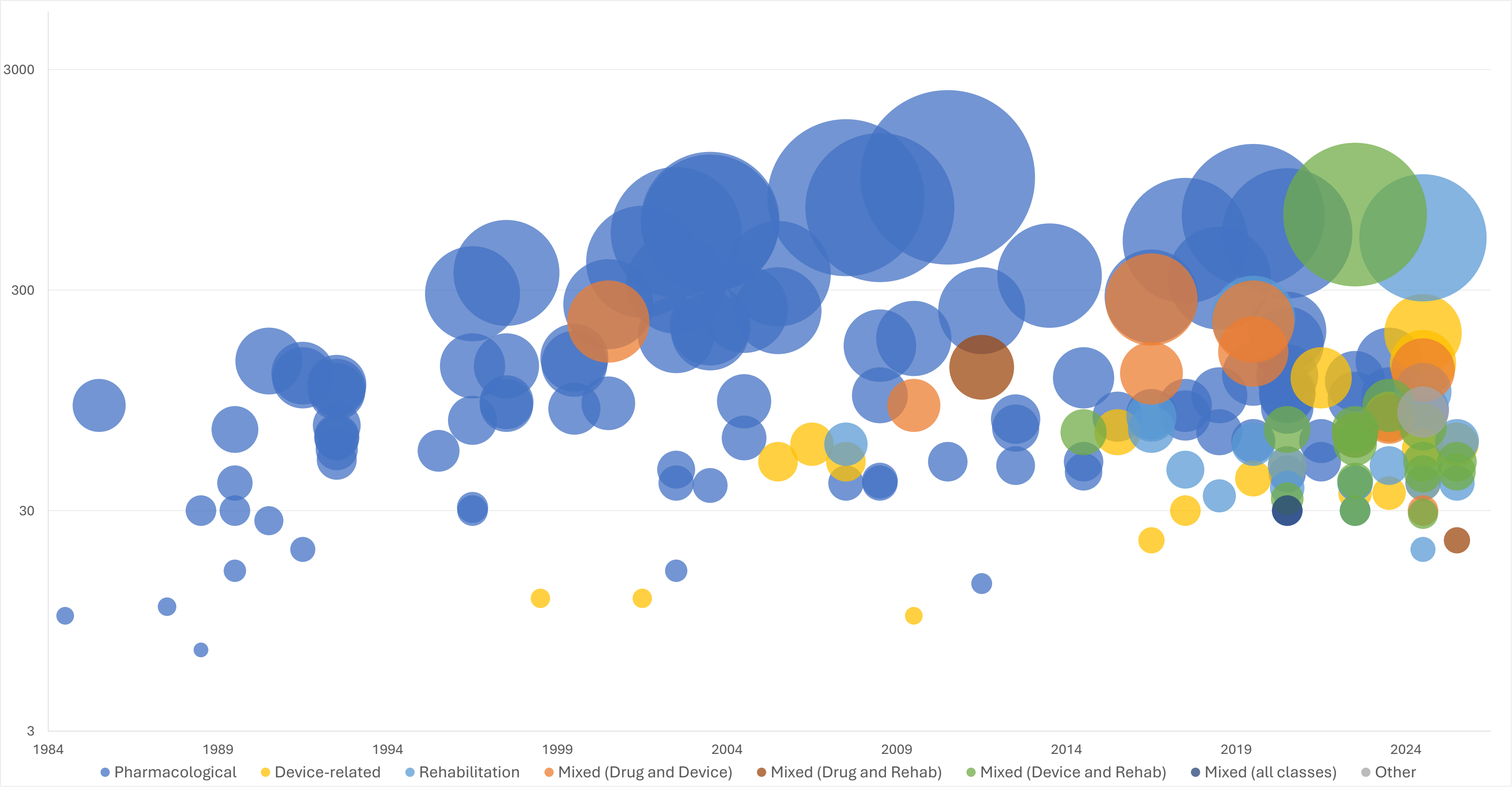

eFigure 4: **VCI label use over time**

Legend: Bar chart depicting the proportion of use of diagnostic labels according to year of study publication (years are grouped in 5-year bins from 1984 to 2023).

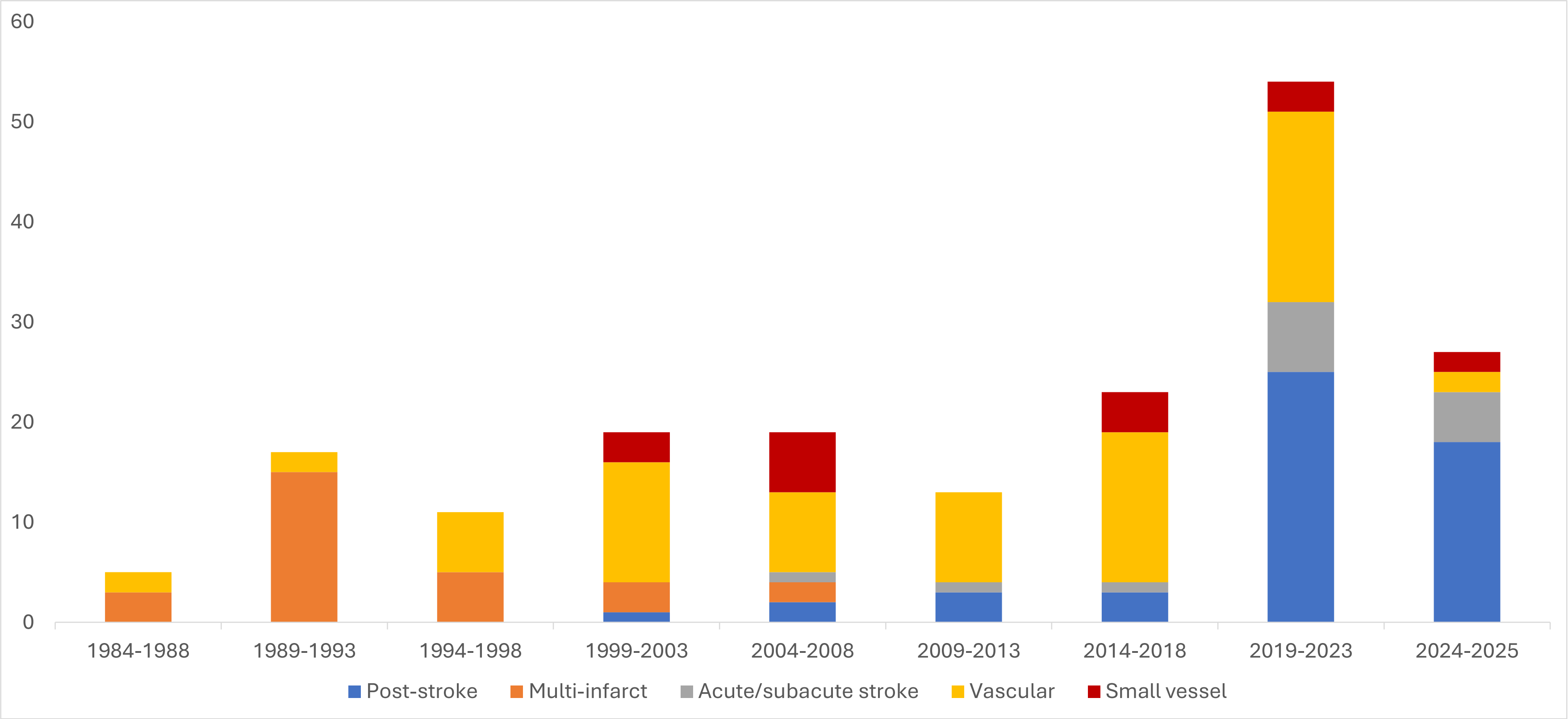

***eFigure 5:* Number of studies evaluating each intervention class along with their comparator**

Legend: Venn diagram depicting the number of studies evaluating the different classes of intervention (pharmacological, requiring device application or rehabilitative), alone or in combination, stratified by comparator. *Abbreviations*: *BMT, Best Medical Treatment*.

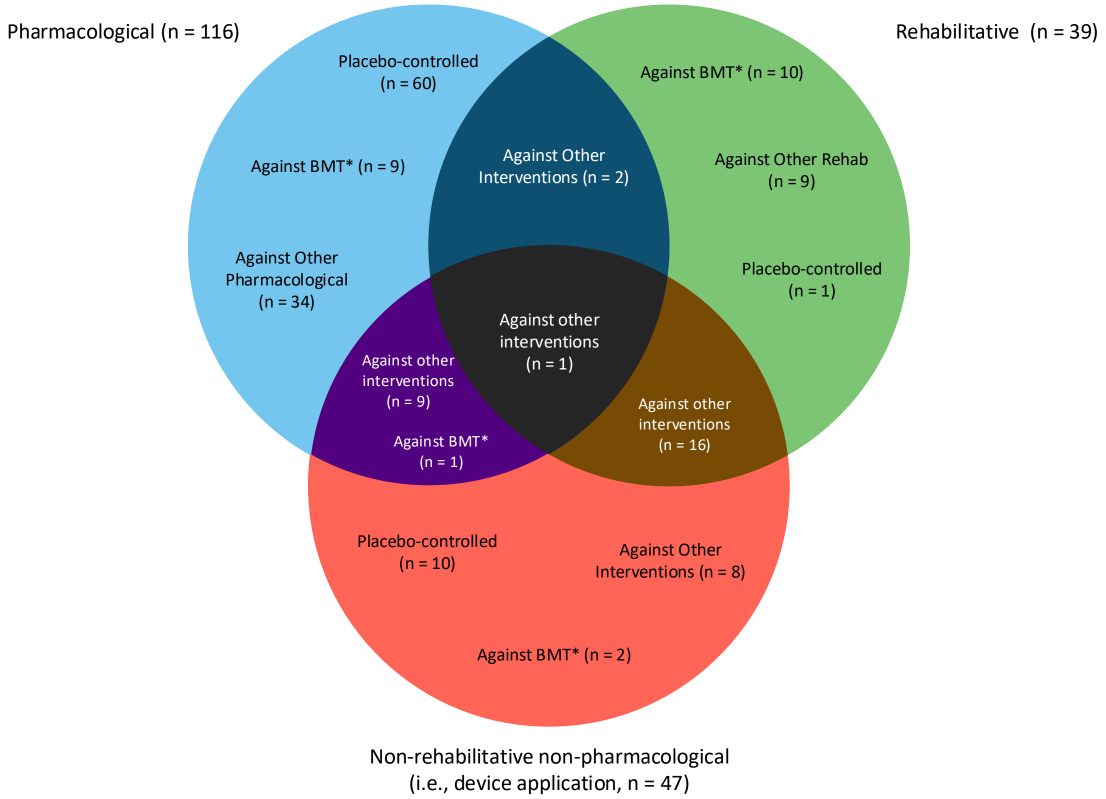

***eFigure 6:* Most frequently reported intervention and intervention classes**

Legend: Sunburst chart of intervention and intervention classes reported most frequently, depicted according to their relative proportion. *Abbreviations: ASA: acetylsalicylic acid; cond., conditioning; iTBS, intermittent Theta-Burst Stimulation; tDCS, transcranial Direct Current Stimulation; TMS, transcranial magnetic stimulation.*

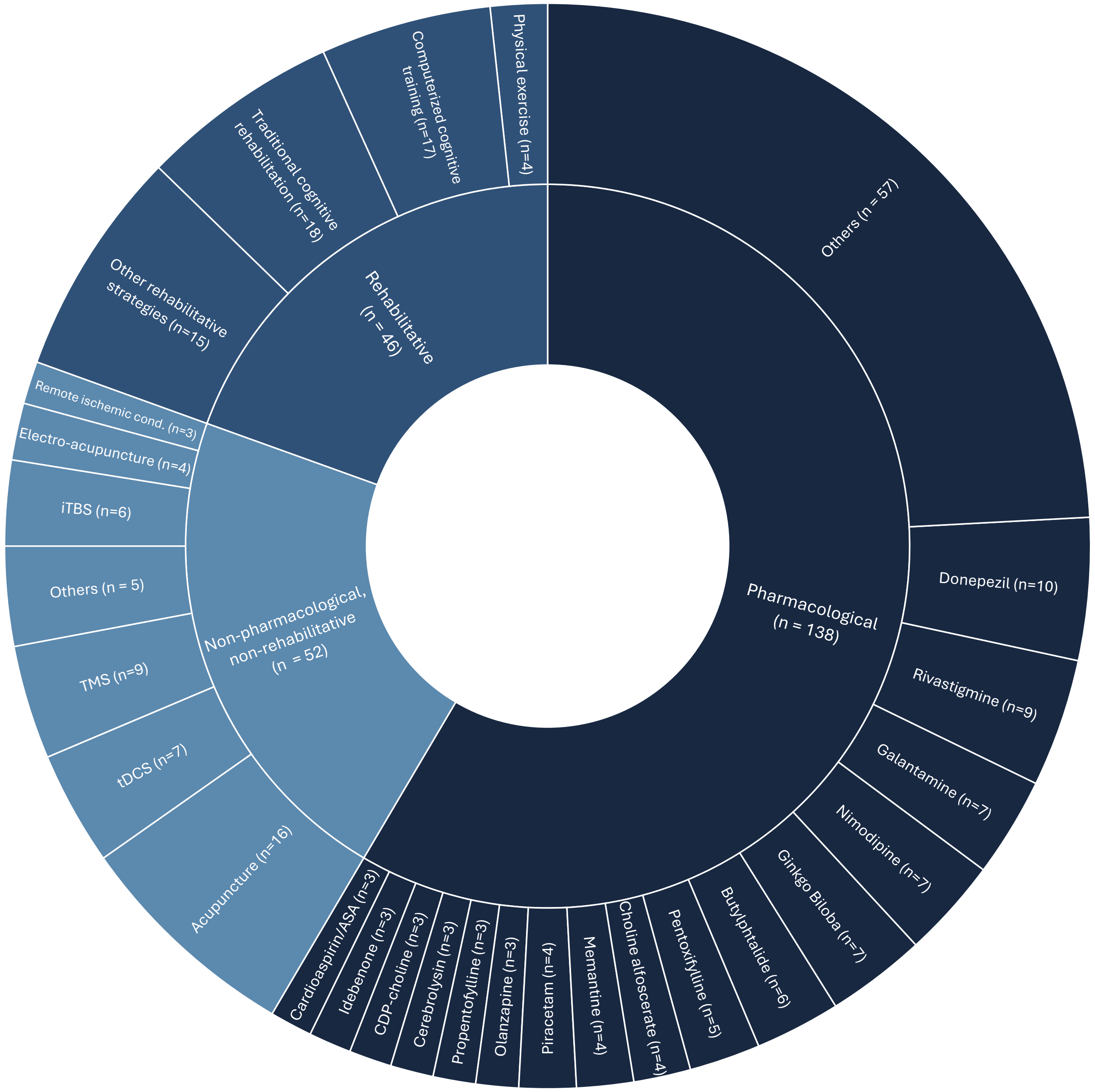

eFigure 7: Outcome and outcome classes reported most frequently

Legend: Outcome and outcome classes reported most frequently depicted according to their relative proportion. *Abbreviations: ADAS, Alzheimer's Disease Assessment Scale; ADCG-CGIC, Alzheimer's Disease Cooperative Study-Clinical Global Impression of Change; ADL, activities of daily living; BDNF, brain-derived neurotrophic factor; BGP, Beurteilungsskala für Geriatrische Patienten; BI, Barthel index; CDR-s, Clinical Dementia Rating scale; CDT, Clock Drawing Test; CIBIC-plus, Clinician's Interview-Based Impression of Change Plus caregiver input; CIRS, Cumulative Illness Rating Scale; DADs, Disability Assessment for Dementia; EEG, electroencephalography; EP, evoked potentials; FDG-PET SUVr, Fluorodeoxyglucose–positron emission tomography standardized uptake value ratio; fMRI, functional magnetic resonance imaging; fNIRS; functional Near Infrared Spectroscopy; GBS, Gottfries-Bråne-Steen Scale; HCT, hematocrit; HDRS, Hamilton Depression Rating Scale; IADL, instrumental activities of daily living; mBI, modified Barthel Index; MMSE, Mini-Mental State Examination; MoCA, Montreal Cognitive Assessment; MR, magnetic resonance; NOSGER, Nurses Observation Scale for Geriatric Patients; NPI, Neuropsychiatric Inventory; NPS, neuropsychological testing; pMDA, plasma malondialdehyde; pSOD, plasma superoxide dismutase; rCBF, regional cerebral blood flow; SCAG, Sandoz Clinical Assessment-Geriatric scale; sTNF-alpha, serum tumor necrosis factor alpha; sIL-6, serum interleukin 6; TC US, transcranial ultrasound; WMH, white matter hyperintensity*.

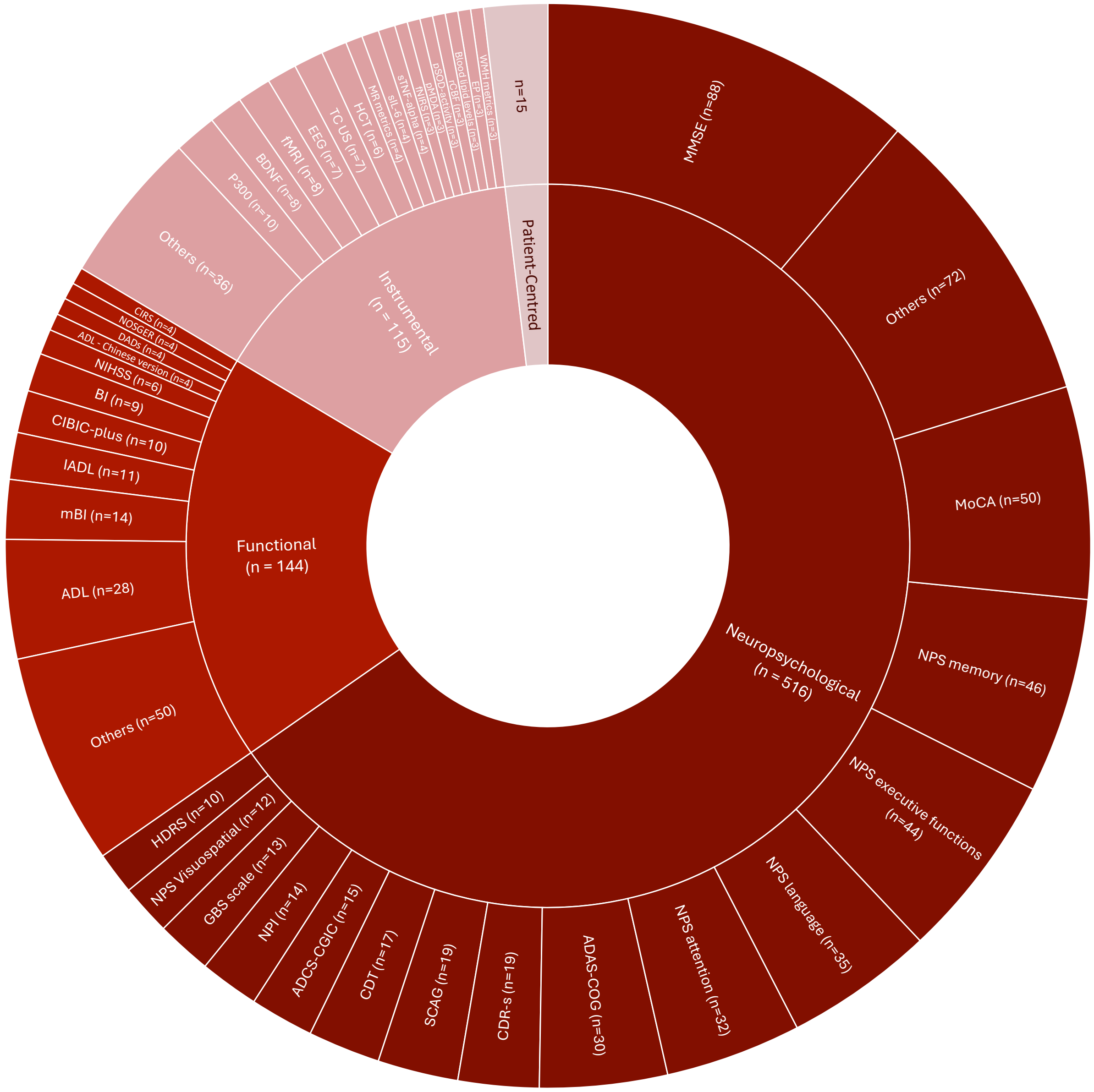

***eFigure 8:* Distribution of intervention duration**
Legend: Histogram depicting the distribution of intervention duration in included studies.

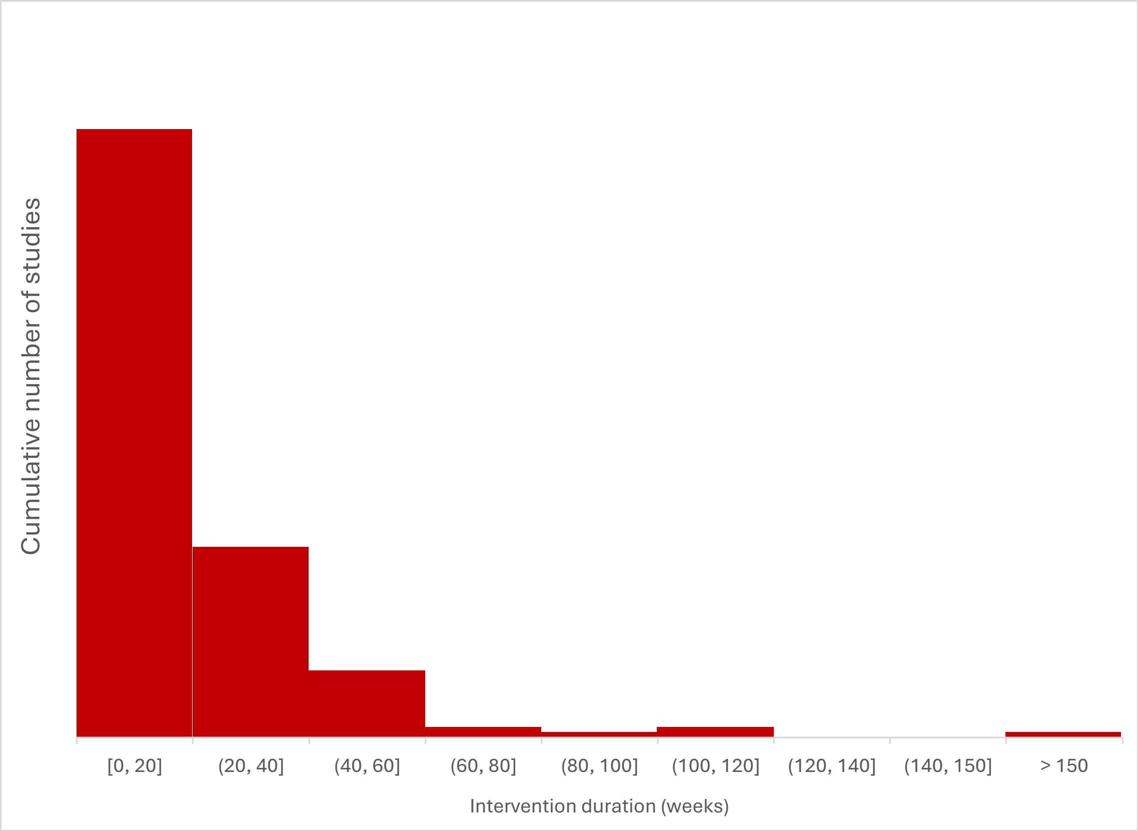

***eFigure 9*: Main global cognitive efficiency outcomes employed throughout the years**
Legend: Bar chart depicting the absolute number of studies employing one of the main global cognitive efficiency outcomes (*colour-coded*) throughout the years *(single years have been grouped into 5- or 4-year bins*).

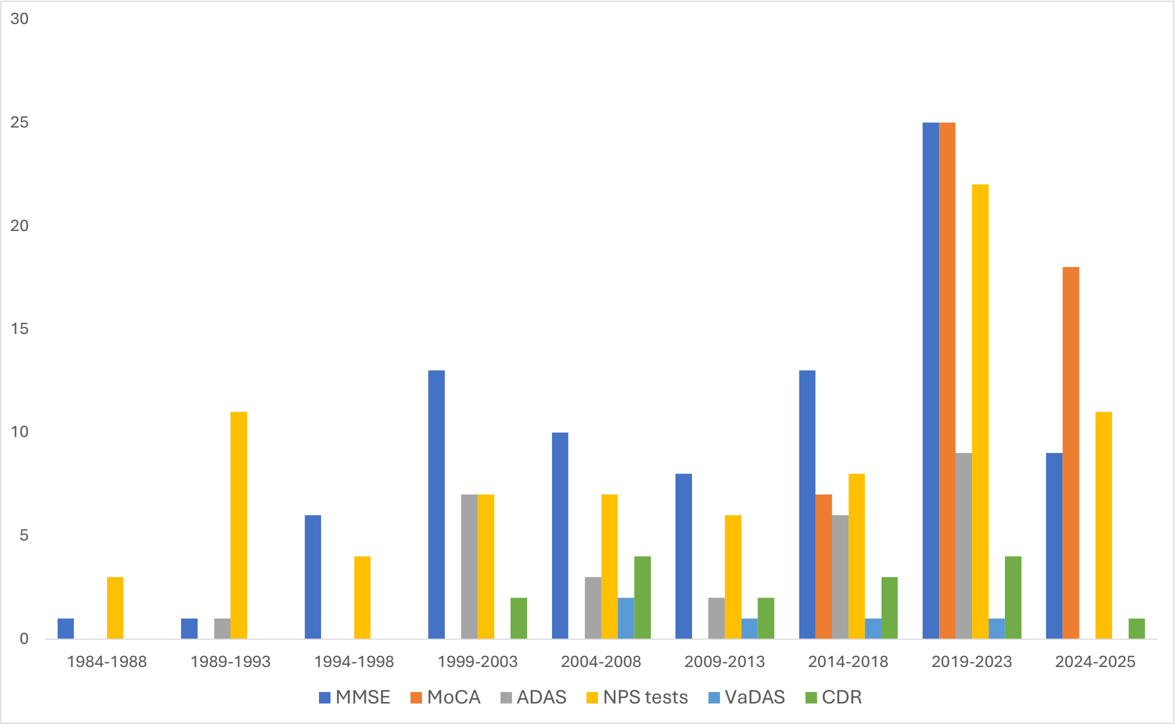

***eFigure 10:* Different intervention category tested throughout the years**

Legend: Bar chart depicting the absolute number and the relative proportion of studies published throughout the years *(single years have been grouped into 5- or 4-year intervals*) according to category of intervention tested (colour-coded)

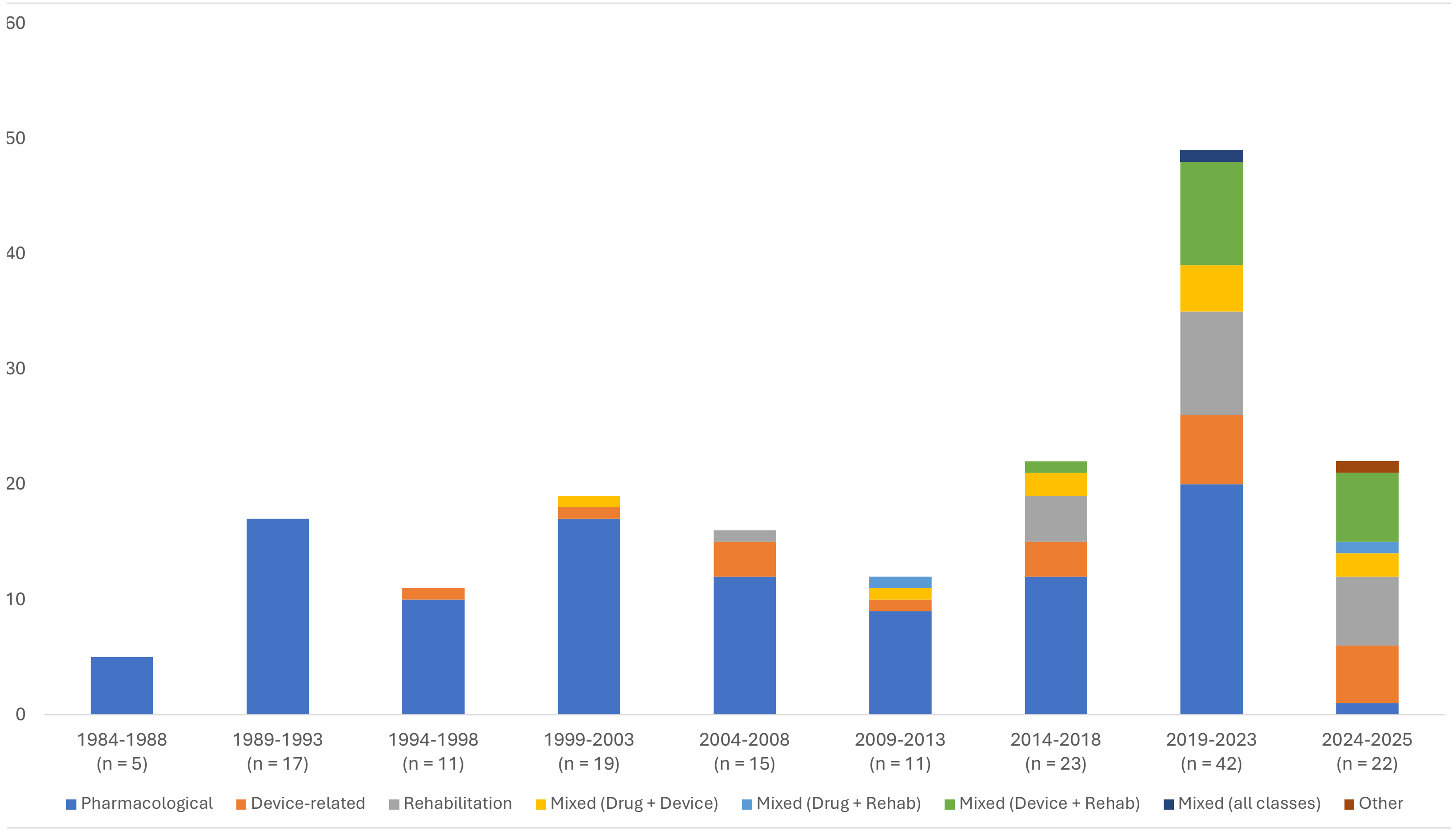

***eFigure 11*: Overall rated efficacy of studies**

Legend: Pie chart depicting the proportion of study efficacy (as rated according to reported outcomes, see methods for rating specifics for each efficacy category).

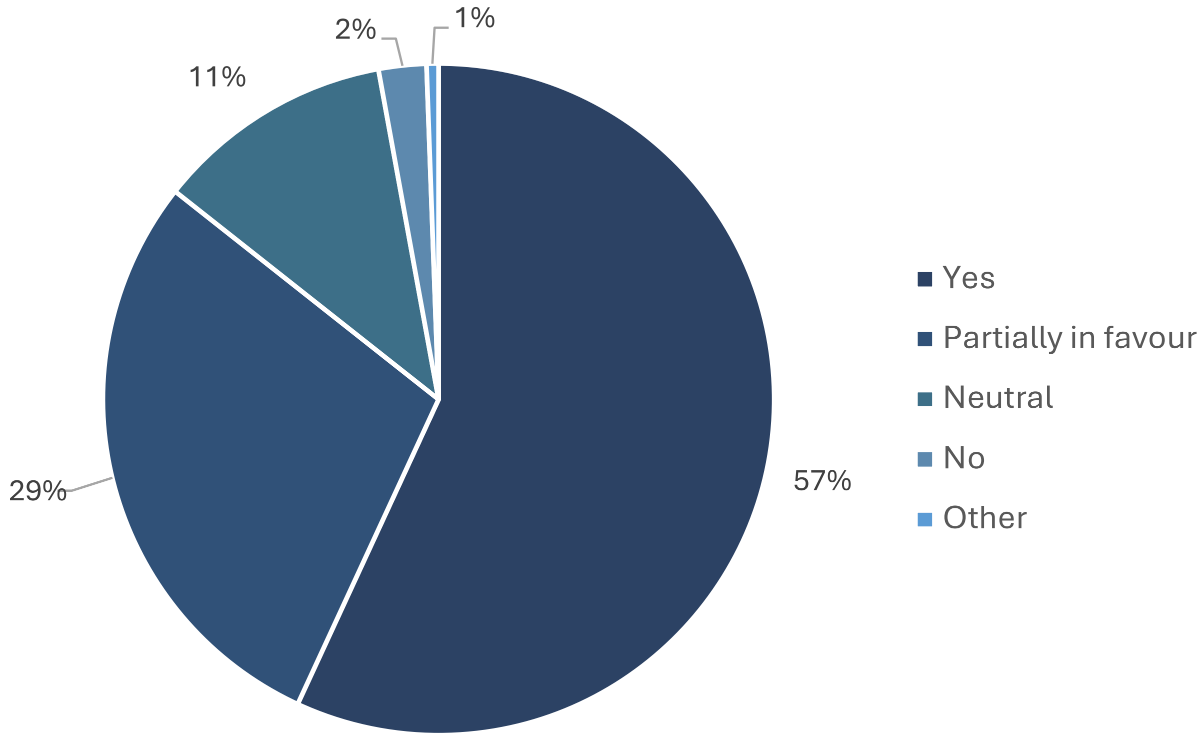

***eFigure 12*: Study global quality ratings and item-specific risk of bias according to NIH-QAT**

Legend: bar chart displaying the overall quality ratings according to the NIH Quality Assessment Tools (NIH-QAT) for Controlled Intervention Studies, as well as the risk of bias for each one of the quality-scale subitems.

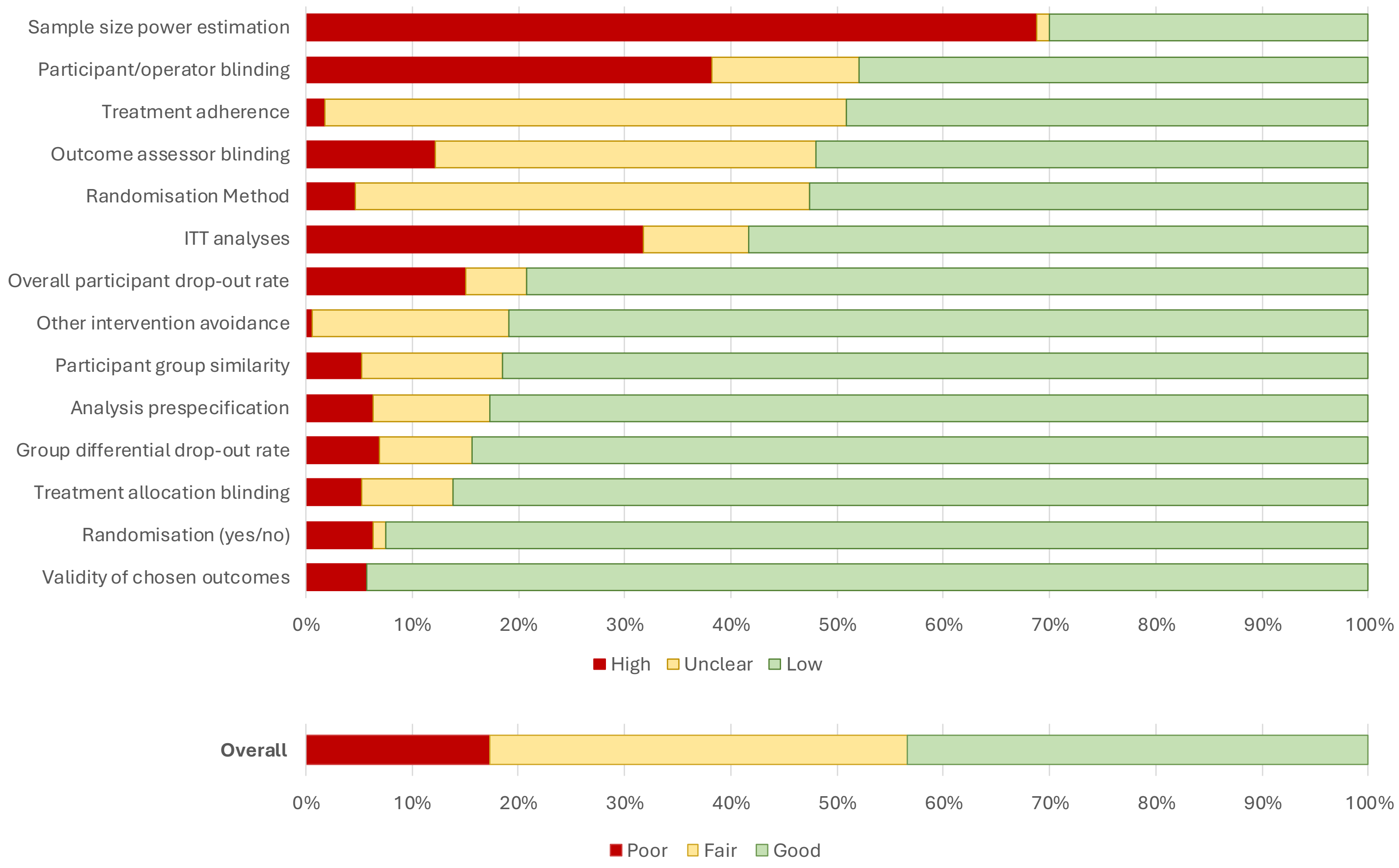

***eFigure 13*: Overall study quality assessment score for studies stratified according to year of publication**
Legend: Bar chart depicting the absolute number of studies published throughout the years *(single years have been grouped into 5- or 4-year intervals*) stratified according to overall study quality assessment score.

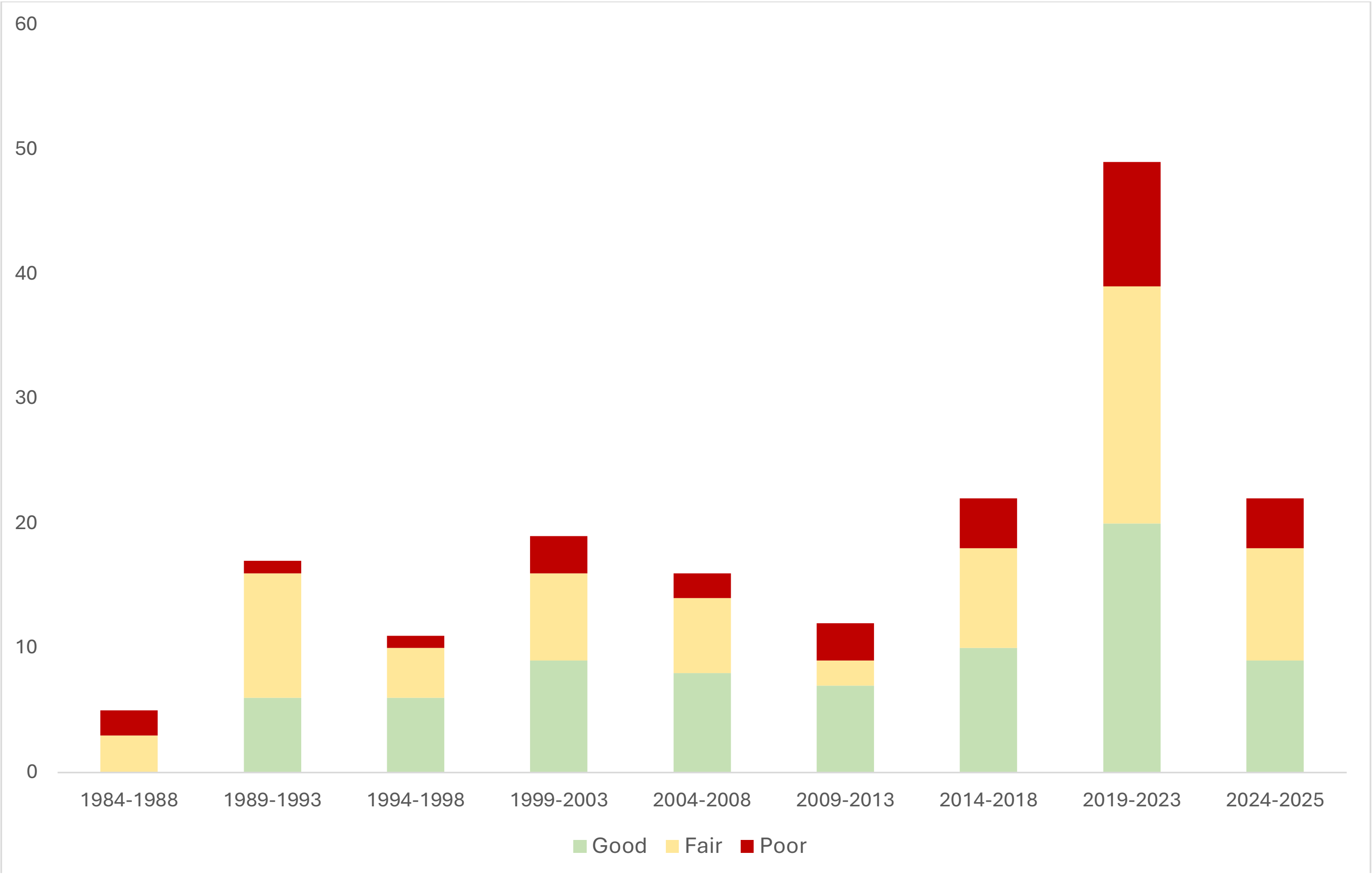

2B. SUPPLEMENTARY RESULTS: QUALITATIVELY SUMMARIZED INTERVENTIONS

2B.1 PHARMACOLOGICAL INTERVENTIONS

eTable 2 reports all the pharmacological reviewed studies not included for meta-analysis *(n = 64)*. The table details population, intervention, comparator, outcomes, and efficacy results along with quality metrics.

Among the 52 pharmacological interventions evaluated, eight studies reported absences of clear positive effect, whereas one was incomplete about results of efficacy. The remaining studies showed positive effect on cognition, and none reported serious adverse events. Pharmacological interventions include diverse drugs categories (sartans, selective serotonin reuptake inhibitors, azapirones, selective antagonist of 5-HT_2 receptors, ergot derivatives, atypical antipsychotics, racetams, piperazine derivates, indole alkaloid), essential elements (vitamins, synthetic analogue of coenzyme Q10, sulfomucopolysaccharides, glycosaminoglycans) and manifold complementary alternative medicine compounds. A positive judgment from study quality assessment was reached in 22/64 articles.

2B.2 REHABILITATIVE STRATEGIES

eTable 3 reports rehabilitative strategies studies not included in meta-analysis *(n* = 10*)*. The table details population, intervention, comparator, outcomes and efficacy result along with quality metrics. Among the rehabilitative strategies, virtual reality *(n = 2)* showed positive results, as cognitive stimulation and traditional cognitive training did when combined *(n* = 3*)*. None reported serious adverse events. A positive judgment from study quality assessment was reached in 3/10 articles.

2B.3 NON-REHABILITATIVE NON-PHARMACOLOGICAL (PHYSICAL DEVICE APPLICATION) INTERVENTIONS

eTable 4 reports all the reviewed studies on physical device application interventions (*n* = 36). The table details population, intervention, comparator, outcomes and efficacy result along with quality metrics. In

the device-related interventions acupuncture and neurostimulation technique prevailed, and none reported serious adverse events. Traditional acupuncture (n = 16), irrespectively of the protocol adopted, showed consistent good results, even though it was frequently investigated in combination with other interventions (often rehabilitative) and was rarely compared against inactive treatment. Light therapy was also shown to ameliorate cognition. A positive judgment from study quality assessment was reached in 12/36 articles.

2B.4 OTHER INTERVENTIONS

One study reported an intervention that did not fall into any of the aforementioned categories. This study [147] described the pharmacological blockade of the stellate ganglion via local chemical agent infiltration for the treatment of dysphagia in patients with subcortical vascular cognitive impairment, with global cognitive efficiency reported as a secondary outcome. Due to the unique nature of this intervention compared to the others, the study is presented separately in *eTable 5*.

2C. SUPPLEMENTARY RESULTS: META-ANALYSES

2C.1 Interventions candidate to meta-analysis

Of the 22 treatments identified for potential meta-analysis, each studied in at least three trials, fourteen met our inclusion criteria (i.e., evaluated as monotherapy against inactive treatment in ≥3 studies): Ginkgo Biloba, Donepezil, Galantamine, Rivastigmine, Memantine, Cerebrolysin, Pentoxifylline, Propentofylline, Nimodipine, remote ischemic conditioning, cognitive rehabilitation, rTMS, tDCS and physical exercise.

Excluded treatments:

- **Acupuncture:** 22 studies, including 16 studies on conventional acupuncture, 4 studies on electroacupuncture, and 2 studies on 'yi qi tiao xue, fu ben pei yuan', with only 4 as monotherapy against inactive treatment (2 conventional acupuncture, 2 electroacupuncture).
- **Butylphtalide:** 6 studies, only 2 as monotherapy against placebo.
- **Intermittent Theta Burst stimulation:** 6 studies, only one investigating it as monotherapy (in other studies it is always combined with rehabilitative strategies) and always against other interventions.
- **Piracetam:** 4 studies, primarily in combination with other treatment (3 of 4).
- **Choline alfoscerate:** 4 studies, none in monotherapy against placebo.
- **CDP-choline:** 3 studies, 2 as monotherapy against inactive treatment and one against other pharmacological treatments.
- **Idebenone:** 3 studies, 2 as monotherapy against inactive treatment and 1 in combination with piracetam.
- **Olanzapine**: 3 studies, 2 as monotherapy but always against other active interventions (other antipsychotic medications).

For intervention that met our meta-analysis inclusion criteria, we report characteristics of studies, summary of findings on pre-specified outcome classes (global cognitive efficiency, functional outcomes,

patient-centred outcomes and safety outcomes), alongside their relative meta-analyses. Sensitivity analyses are also reported in a dedicated paragraph at the end of main meta-analysis results and plots.

2C.2 Gingko Biloba

2C.2-1 Description of studies and meta-analysis main results

We retrieved n=7 studies evaluating *Gingko Biloba extracts* in the therapy of VCI, 5 studies employing it as monotherapy while two employing it in combination with other pharmacological strategies (integrated Chinese medicine strategies). Only studies evaluating this target intervention as monotherapy are further considered for meta-analysis.

Among these five, four employed the same dosage (240 mg) while one employed half the dosage (120 mg). Duration of treatment was similar (~ 24 weeks) except for one study which evaluated treatment effect over 12 weeks. All studies reported global cognitive efficiency measures within the outcomes: SKT (3), MMSE (2); 3 studies reported further neuropsychological evaluation. One study reported functional outcomes (ADL), while no studies reported patient-centred outcomes.

Quality of studies was rated as good in 3 studies, fair in 1 study and poor in 1 study (the latter due to significant risk of bias in randomisation methods, baseline participant characteristics and overall statistical design of the study). Safety outcomes were available in 2/5 studies.

Final meta-analysis was performed among studies of "fair" or better quality and employing similar treatment dose (within the 33% tolerance limit) on global cognitive efficiency metrics, functional metrics, and safety outcomes (overall adverse events, and severe adverse events). It showed moderate effects of Gingko Biloba extracts on global cognitive efficiency metrics (Cohen's d 0.83, 95% CI 0.00 – 1.67, p = 0.049), and low-to-moderate effect on functional outcomes (Cohen's d 0.50, 95% CI 0.25-0.75, p < 0.001). Rates of adverse events and severe adverse events were not shown to be significantly different between treatment arms. Results of meta-analyses for each outcome classes are summarised and depicted in the *Summary of Findings table* and in their relative forest plots.

2C.2-2 Characteristic of studies

Table Caption: Characteristics of studies assessing *Gingko Biloba* for Vascular Cognitive Impairment.

Setting: hospital and clinics
Intervention: *Gingko Biloba extracts*

Studies included in meta-analysis:

VCI population (label)	Treatment arms	Treatment duration/follow-up	Outcomes	Efficacy	Safety	Quality score*	Study
Vascular dementia	*Gingko biloba extracts 240 mg (94)*<br><br>*vs*<br><br>*Placebo (87)*	*22 weeks (NP)*	***<u>Primary outcomes</u>:***<br>*Yes (SKT)*<br><br>*<u>Other outcomes</u>:*<br>*Cognitive: yes*<br>*Functional: no*<br>*Patient-centred: no*	*In favour of treatment*	*Rate of AE and SAE equally represented in both arms*	***Overall**: Good*<br><br>***QI**:*<br>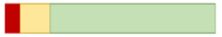	[23]
MID	*Gingko biloba extracts 240 mg (18)*<br><br>*vs*<br><br>*Placebo (13)*	*24 weeks (NP)*	***<u>Primary outcomes</u>:***<br>*Yes (SKT, ADCS-CGIC)*<br><br>*<u>Other outcomes</u>:*<br>*Cognitive: yes*<br>*Functional: no*<br>*Patient-centred: no*	*Partially in favour of treatment*	*Safety outcomes not reported for MID subgroup separately*	***Overall**: Good*<br><br>***QI:***<br>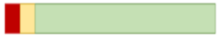	[36]
Vascular dementia	*Gingko biloba extracts 240 mg (39)*	*24 weeks (NP)*	***<u>Primary outcomes</u>:***<br>*Yes (SKT, NPI)*	*In favour of treatment*	*Rate of AE and SAE balanced in both arms*	***Overall**: Good*<br><br>***QI:***<br>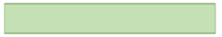	[43]

	vs<br><br>*Placebo (32)*		*<u>Other outcomes</u>:*<br>*Cognitive: yes*<br>*Functional: yes*<br>*Patient-centred: yes*				

Studies included only in sensitivity analyses or excluded:

VCI population (label)	Treatment arms	Treatment duration/follow-up	Outcomes	Efficacy	Safety	Quality score*	Study
Vascular MCI	*Gingko biloba extracts 240 mg (30)*<br><br>*vs*<br><br>*best medical treatment (15)*	*12 weeks (NP)*	***<u>Primary outcomes</u>*:**<br>*None reported*<br><br>*<u>Other outcomes</u>:*<br>*Cognitive: yes*<br>*Functional: no*<br>*Patient-centred: no*	*Neutral*	*Safety outcomes not reported*	**Overall**: Poor<br><br>***QI***: 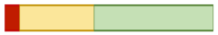	[22]
Vascular Cognitive Impairment	*Gingko biloba extracts 120 mg (30)*<br><br>*vs*<br><br>*Placebo (30)*	*36 weeks (NP)*	***<u>Primary outcomes</u>*:**<br>*Yes (MDRS, ADCS-CGIC, SCAG, MMSE)*<br><br>*<u>Other outcomes</u>:*<br>*Cognitive: yes*<br>*Functional: no*<br>*Patient-centred: no*<br>*Instrumental: yes (TC-US)*	*Neutral*	*Safety outcomes not reported*	**Overall**: Fair<br><br>***QI***: 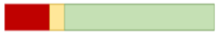	[85]

Vascular MCI	*Gingko biloba extracts 120 mg + Acetilsalicylic acid 225 mg (40)*<br><br>*vs*<br><br>*Acetilsalicylic acid 225 mg (40)*	*12 weeks (NP)*	***Primary outcomes**:*<br>*None reported*<br><br>*Other outcomes:*<br>*Cognitive: yes*<br>*Functional: no*<br>*Patient-centred: no*<br>*Instrumental: yes (TC-US)*	*In favour of treatment*	*Safety outcomes not reported*	**Overall**: Fair<br><br>**QI**:<br>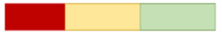	[14]
Vascular dementia	*Shenmayizhi Formula 9.6 g + Gingko Biloba 3 capsules (85)*<br><br>*vs*<br><br>*Placebo + Ginkgo Biloba 3 capsules (87)*	*12 weeks (NP)*	***Primary outcomes:***<br>*Cognitive: MMSE*<br>*Functional: CM-SS*<br>*Instrumental: ET-1, NO, vWF, NSE, BDNF*<br><br>*Other outcomes:*<br>*Patient-centred: no*	*In favour of treatment*	*Safety outcomes not reported*	**Overall**: Good<br><br>**QI**:<br>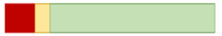	[1]

Abbreviations: ADCG-CGIC, Alzheimer's Disease Cooperative Study-Clinical Global Impression of change; AE, adverse events; CM-SS, Chinese Medicine Symptom Scale; ET-1, serum endothelin-1; BDNF, plasma brain-derived neurotrophic growth factor; MDRS, Mattis Dementia Rating Scale; MMSE, Mini-mental State Exam; NO, plasma nitric oxidase; NSE, serum neuronal specific enolase; TC-US, transcranial ultrasound; SAE, severe adverse events; SCAG, Sandoz Clinical Assessment-Geriatric vWF; plasma von-Willebrand Factor.

Notes:
*Overall quality as rated according to the NIH Quality Assessment tools for controlled intervention studies is reported here. QI (Quality Index) is a graphical, colour-coded representation of the number of items on the scale rated respectively as at high-risk (red), unclear risk (yellow) or low-risk (green) of bias.

2C.2-3 Summary of findings and figures for meta-analyses

Table Caption: Summary of findings for the main comparisons. *Gingko Biloba* for Vascular Cognitive Impairment.

Gingko Biloba for Vascular Cognitive impairment

Setting: hospital and clinics
Intervention: *Gingko Biloba extracts*
Comparator: *placebo*

Outcomes	N° of participants (studies)	VCI population (label)	Efficacy measure	Quality of evidence (GRADE)	Statistical heterogeneity	Studies
Global cognitive efficiency<br>*SKT*<br><br>***Treatment duration:*** *22-24 weeks*<br><br>***Follow-up after treatment:*** *none*	283 (3 RCTs)	Vascular Dementia<br>MID	**Cohen's d:**<br>0.83<br>95% CI (0.00 – 1.67)<br><br>**Mean difference:**<br>+ 2.56<br>95% CI (-0.17, 5.29)	⊕○○○ Very low[2,3,4]	I^2= 87.71	[23]<br>[36]<br>[43]
Functional outcomes<br>*GBS-ADL subscale and ADL-IS*<br><br>***Treatment duration:*** *22-24 weeks*<br><br>***Follow-up after treatment:*** *none*	252 (2 RCTs)	Vascular Dementia	**Cohen's d:**<br>0.50<br>95% CI (0.25 – 0.75)	⊕⊕○○ Low[5,6]	I^2= 0	[23]<br>[43]

Patient-centred outcomes<br>*Not reported*	*See note[1]*	*See note[1]*	*See note[1]*			
***Safety outcomes** (AE and SAE)*	252 (2 RCTs)	Vascular Dementia	**AE (rate ratio):**<br>0.98<br>*95% CI (0.58 – 1.66)*<br><br>**SAE (rate ratio):**<br>0.15<br>*95% CI (0.13 – 2.34)*	**AE:**<br>⊕⊕○○ Low[6,7]<br><br><br>**SAE:**<br>⊕⊕○○ Low[3]	 I^2= 82.61<br><br><br><br>I^2= 0	[23]<br>[43]

Abbreviations: ADL, Activities of Daily Living; ADL-IS, Activities of Daily Living International Scale; AE, adverse events; GBS, Göttfries-Brane-Steen Scale; SKT, Short Cognitive Performance Test; SAE, Severe Adverse events.

Notes:
[1] No studies among the one included in meta-analysis reported patient-centred outcomes.

GRADE Working Group grades of evidence:

High certainty: We are very confident that the true effect lies close to that of the estimate of the effect.

Moderate certainty: We are moderately confident in the effect estimate: the true effect is likely to be close to the estimate of the effect, but there is a possibility that it is substantially different.

Low certainty: Our confidence in the effect estimate is limited: the true effect may be substantially different from the estimate of the effect.

Very low certainty: We have very little confidence in the effect estimate: the true effect is likely to be substantially different from the estimate of effect.

---

[2]Low generalisability due to inclusion of different VCI populations (downgraded once for indirectness)
[3]Downgraded twice due to imprecision: the 95% CI includes a result that would not be considered clinically important and a result that would be considered important.
[4]Two or more items at uncertain (or greater) risk of bias (downgraded once).
[5]Different outcome measures employed (downgraded once).
[6]Some inconsistency in point estimates (downgraded once).
[7]Downgraded once due to imprecision (wide confidence interval)

eFigure 14: *Forest plot representing meta-analysis of Gingko biloba effect in VCI on global cognitive efficiency outcomes (SKT). Panel a reports effect size expressed as Cohen's d while panel b reports effect size expressed as unstandardised mean difference; random effect models were used for both estimations.*

a

	ES	95% CI	W	SE	Sig.	N	N1	N2
Ihl 2012	0.49	0.01 / 0.96	34.08%	0.24	0.043	71	39	32
Kanowski 1996	0.36	-0.36 / 1.08	29.80%	0.37	0.327	31	18	13
Napryeyenko 2009	1.55	1.22 / 1.88	36.11%	0.17	0.000	181	94	87
Overall (random-effects model)	0.83	0.00 / 1.67	100.00%	0.42	0.049	283	151	132

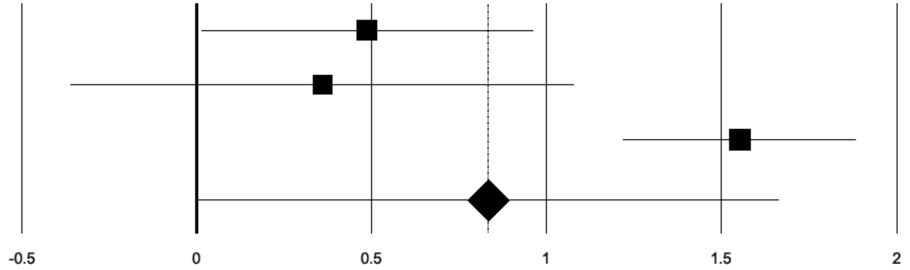

Heterogeneity: Cochran's Q = 17.54, df = 2 (p < 0.001), Tau^2 = 0.47, I^2 = 88.60

b

	ES	95% CI	W	SE	Sig.	N	N1	N2
Ihl 2012	1.40	0.06 / 2.74	34.19%	0.68	0.040	71	39	32
Kanowski 1996	1.10	-1.08 / 3.28	30.08%	1.11	0.323	31	18	13
Napryeyenko 2009	4.90	3.98 / 5.82	35.73%	0.47	0.000	181	94	87
Overall (random-effects model)	2.56	-0.17 / 5.29	100.00%	1.39	0.066	283	151	132

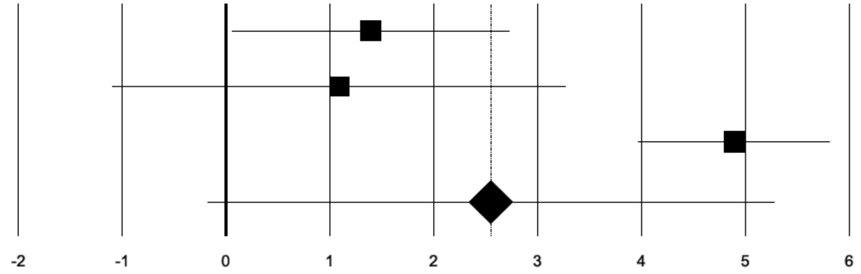

Heterogeneity: Cochran's Q = 23.00, df = 2 ($p < 0.001$), Tau^2 = 5.20, I^2 = 91.30

eFigure 15: *Forest plot representing meta-analysis of Gingko biloba effect in VCI on functional outcomes (Göttfries-Brane-Steele ADL subscore and ADL International Scale). Effect size is reported as Cohen's d; fixed effect model was used for estimation.*

	ES	95% CI	W	SE	Sig.	N	N1	N2
Ihl 2012	0.37	-0.10 / 0.84	28.42%	0.24	0.125	71	39	32
Napryeyenko 2009	0.55	0.25 / 0.85	71.58%	0.15	0.000	181	94	87
Overall (fixed-effect model)	0.50	0.25 / 0.75	100.00%	0.13	0.000	252	133	119

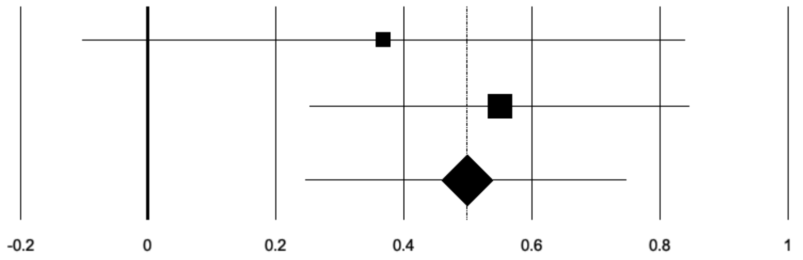

Heterogeneity: Cochran's Q = 0.41, df = 1 (p = 0.524), $Tau^2 = 0$, $I^2 = 0$

eFigure 16: *Forest plot representing Gingko biloba safety in VCI (adverse events, panel a, and severe adverse events, panel b). Effect size is reported as rate ratio when not otherwise specified; random or fixed effect models were used for estimation as appropriate.*

a

Heterogeneity: Cochran's Q = 5.75, df = 1 (p = 0.016), Tau^2 = 0.12, I^2 = 82.61

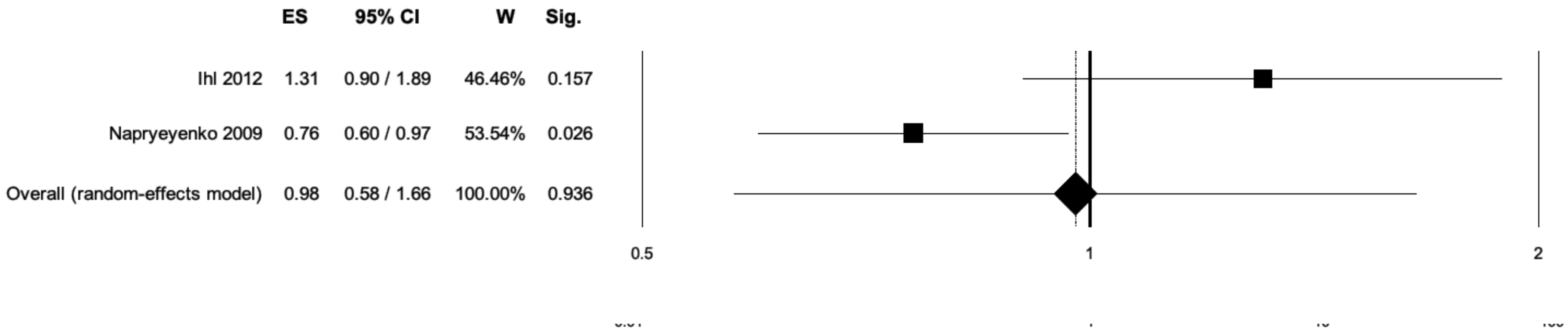

b

Heterogeneity: Cochran's Q = 0.15, df = 1 (p = 0.703), Tau^2 = 0, I^2 = 0

2C.2-4 Sensitivity Analyses

We performed sensitivity analyses varying $Corr_{pre\text{-}post}$ for functional outcomes, as $Corr_{pre\text{-}post}$ was not reported nor inferable from previous studies for this class of outcomes. We performed also sensitivity analyses including studies with differing *Ginkgo Biloba* dosage [85], and including studies rated at high risk of bias according to quality rating instruments [22].

Effect size was overall superimposable for $Corr_{pre\text{-}post}$ variation (effect size variation, ***Cohen's d***, - 0.06 ie, ± 12% in proportion with main analysis). Forest plots for these analyses as well as their relative heterogeneity statistics are reported in *eFigure 17*.

Effect size was overall superimposable when performing meta-analysis including one study [85] with different Ginkgo Biloba dosage (120 mg instead of 240 mg; effect size variation for global cognitive efficiency *Cohen's d*, -0.18 ie, -21% in proportion with main analysis, and for adverse events rate ratio, -0.04 ie, -8% compared to main analysis). A further sensitivity analysis including one study [22] at high risk of bias and differing treatment duration (12 weeks instead of 24 weeks) is reported below. Forest plots for these analyses as well as their relative heterogeneity statistics are reported in *eFigures 18* and *19*, respectively; characteristics of studies included in sensitivity analyses are reported in the Characteristics of Studies table.

***eFigure 17**: Forest plot representing sensitivity-analyses of Gingko biloba effect in VCI on functional outcomes (Göttfries-Brane-Steele ADL subscore and ADL International Scale), variating pre-post correlation coefficients between 0 and 0.8 (panel **a** and panel **b** respectively). Effect sizes are reported as Cohen's d; random or fixed effect models was used for estimation as appropriate.*

a

	ES	95% CI	W	SE	Sig.	N	N1	N2
Ihl 2012	0.58	0.10 / 1.06	27.93%	0.24	0.017	71	39	32
Napryeyenko 2009	0.55	0.25 / 0.85	72.07%	0.15	0.000	181	94	87
Overall (fixed-effect model)	0.56	0.31 / 0.81	100.00%	0.13	0.000	252	133	119

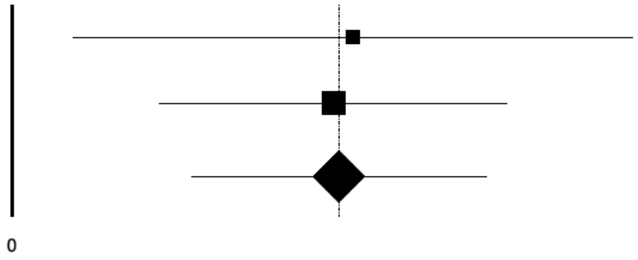

Heterogeneity: Cochran's Q = 0.01, df = 1 (p = 0.911), Tau^2 = 0.00, I^2 = 0.00

	ES	95% CI	W	SE	Sig.	N	N1	N2
Ihl 2012	0.26	-0.21 / 0.73	29.40%	0.24	0.276	71	39	32
Napryeyenko 2009	0.55	0.25 / 0.85	70.60%	0.15	0.000	181	94	87
Overall (fixed-effect model)	0.46	0.21 / 0.72	100.00%	0.13	0.000	252	133	119

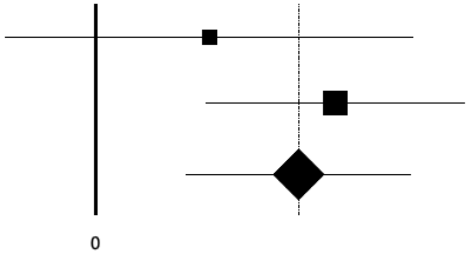

Heterogeneity: Cochran's Q = 1.04, df = 1 (p = 0.308), Tau^2 = 0.00, I^2 = 3.80

__eFigure 18__: Forest plot representing sensitivity-analyses of Gingko biloba effect on global cognitive efficiency (panel a) and safety (adverse events, panel b) including studies with different treatment dosage [85]. Effect sizes are reported as Cohen's d; random effect models were used for estimation.

a

	ES	95% CI	W	SE	Sig.	N	N1	N2
Demarin 2017	0.08	-0.55 / 0.71	24.02%	0.32	0.808	39	20	19
Ihl 2012	0.49	0.01 / 0.96	25.86%	0.24	0.043	71	39	32
Kanowski 1996	0.36	-0.36 / 1.08	22.84%	0.37	0.327	31	18	13
Napryeyenko 2009	1.55	1.22 / 1.88	27.28%	0.17	0.000	181	94	87
Overall (random-effects model)	0.65	-0.10 / 1.41	100.00%	0.39	0.091	322	171	151

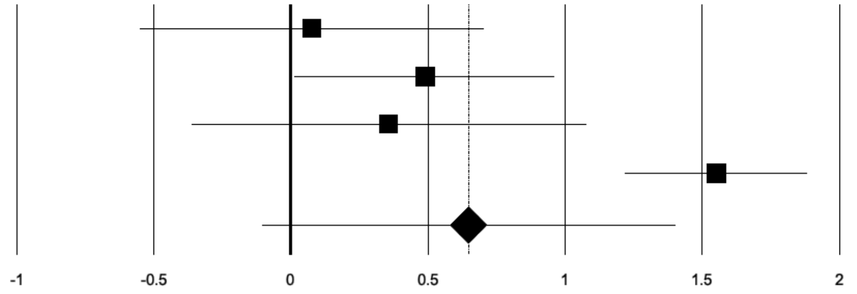

Heterogeneity: Cochran's Q = 22.26, df = 3 (p < 0.0001), Tau^2 = 0.52, I^2 = 88.56

b

	ES	95% CI	W	Sig.
Demarin 2017	0.48	0.20 / 1.11	19.14%	0.086
Ihl 2012	1.31	0.90 / 1.89	37.59%	0.157
Napryeyenko 2009	0.76	0.60 / 0.97	43.27%	0.026
Overall (random-effects model)	0.85	0.53 / 1.37	100.00%	0.511

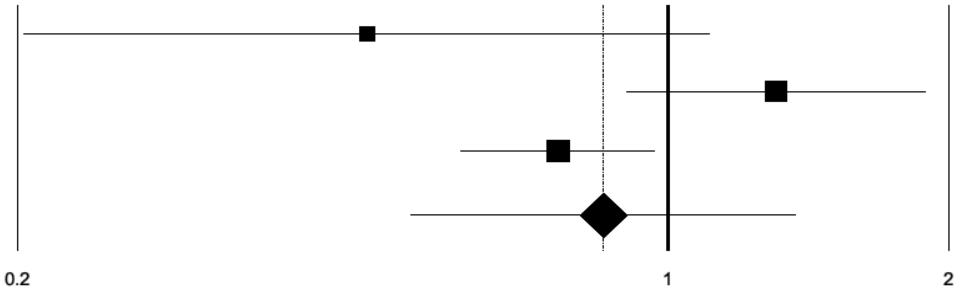

Heterogeneity: Cochran's Q = 7.77, df = 3 (p = 0.021), Tau^2 = 0.12, I^2 = 74.25

***eFigure 19**: Forest plot representing sensitivity-analyses of Gingko biloba effect on global cognitive efficiency only including a study with high risk of bias. Effect sizes are reported as Cohen's d; a random effect model was used for estimation.*

	ES	95% CI	W	SE	Sig.	N	N1	N2
Ihl 2012	0.89	0.03 / 1.75	25.86%	0.44	0.043	71	39	32
Kanowski 1996	0.65	-0.65 / 1.96	22.76%	0.67	0.327	31	18	13
Napryeyenko 2009	2.82	2.21 / 3.42	27.32%	0.31	0.000	181	94	87
Odinak 2014	0.22	-0.90 / 1.35	24.07%	0.57	0.701	45	30	15
Overall (random-effects model)	1.20	-0.15 / 2.55	100.00%	0.69	0.082	328	181	147

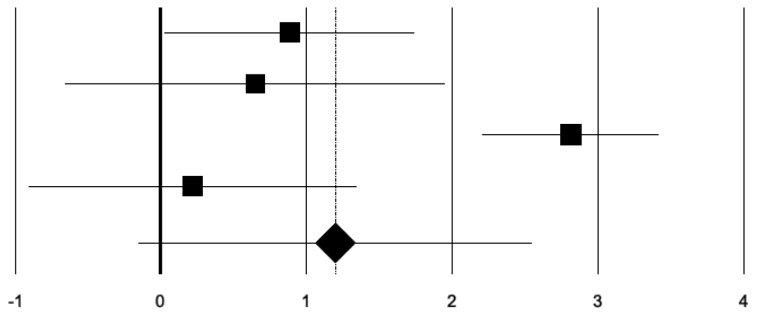

Heterogeneity: Cochran's Q = 25.66, df = 3 (p < 0.0001), Tau² = 1.64, I² = 88.31

2C.3 AcetilCholinesterase Inhibitors – Rivastigmine

2C.3-1 Description of studies and meta-analysis main results

We identified nine studies evaluating rivastigmine in the treatment of vascular cognitive impairment (VCI), all of which employed it as monotherapy. Six studies compared rivastigmine to an inactive treatment (placebo), while three compared it to other active treatment strategies. For the meta-analysis, only studies that assessed rivastigmine monotherapy against placebo were considered.

The quality of the included studies was assessed, with three studies rated as good, two as fair, and one as poor. The poor-quality study was excluded due to significant risk of bias arising from its non-randomised, open-label design and inadequate statistical methodology.

Among the five studies of fair or better quality, the median target dosage of rivastigmine was 6 mg daily. Due to the frequent nausea experienced by patients at the beginning of treatment, a dose titration period was often implemented (8 out of 9 studies) to reach the target dosage. The median treatment duration was 26 weeks. Two studies had longer treatment durations (12-24 months). One of these studies [48] was included only in sensitivity analyses, while the other [52] reported data solely against behavioural outcomes and therefore provided no data relevant to the meta-analysis.

All studies reported measures of global cognitive efficiency; three studies reported additional neuropsychological assessments. One study reported efficacy data on functional outcomes, specifically activities of daily living (ADLs), while no studies reported data on patient-centred outcomes. Safety data were available in three out of five studies, including general adverse events in all three and severe adverse events in one.

The final meta-analysis included studies of fair or better quality that employed similar treatment doses and durations (within ±33% of the median) and assessed global cognitive efficiency, functional outcomes, and safety (overall adverse events and severe adverse events).

The meta-analysis revealed no significant effect of rivastigmine on global cognitive efficiency (Cohen's d -0.01, 95% CI -0.30 – 0.29, $p > 0.05$) or on functional outcomes (Cohen's d 0.04, 95% CI -0.10 – 0.18, $p > 0.05$). There was no significant difference in the rates of adverse events between the treatment groups.

Results of meta-analyses for each outcome classes are summarised and depicted in the *Summary of Findings table* and in their relative forest plots.

2C.3-2 Characteristic of studies

Table Caption: Characteristics of studies assessing *rivastigmine* for Vascular Cognitive Impairment.

Setting: hospital and clinics
Intervention: *rivastigmine (oral tablets)*

Studies included in meta-analysis:

VCI population (label)	Treatment arms	Treatment duration/follow-up	Outcomes	Efficacy	Safety	Quality score*	Study
Subcortical vascular dementia	*Rivastigmine up to 6 mg daily (20)*<br><br>***vs***<br><br>*Placebo (20)*	*26 weeks (NP)*	***Primary outcomes**:*<br>*None reported*<br><br>*Other outcomes:*<br>*Cognitive: yes*<br>*Functional: yes*<br>*Patient-centred: no*	*Neutral*	*Overall rate of AE and SAE not significantly different between treatment arm.*<br><br>*Titration needed by some patients to avoid AE and not all patients were able to reach target dose.*	***Overall**: Good*<br><br>***QI**:*	[97]
Post-stroke MCI	*Rivastigmine up to 9 mg daily (25)*<br><br>***vs***<br><br>*Placebo (25)*	*26 weeks (NP)*	***Primary outcomes**:*<br>*Yes (CDT, NPS executive functions)*<br><br>*Other outcomes:*<br>*Cognitive: yes*<br>*Functional: yes*<br>*Patient-centred: no*	*Neutral*	*Overall rate of AE and SAE not significantly different between treatment arms.*<br><br>*Titration needed by some patients to avoid AE and not all patients were able to reach target dose.*	***Overall**: Good*<br><br>***QI:***	[93]
Vascular dementia	*Rivastigmine up to 12 mg daily (365)*<br><br>***vs***	*24 weeks (NP)*	***Primary outcomes**:*<br>*Yes (VaDAS, ADCS-CGIC)*	*Partially in favour of treatment*	*Rate of AE was significantly different increased in the treatment group. Rate*	***Overall**: Good*<br><br>***QI:***	[82]

	Placebo (345)		*<u>Other outcomes</u>:*<br>*Cognitive: yes*<br>*Functional: yes*<br>*Patient-centred: no*		*of SAE due to cerebrovascular events was significantly increased in treatment group.*		

Studies included only in sensitivity analyses or excluded:

VCI population (label)	Treatment arms	Treatment duration/follow-up	Outcomes	Efficacy	Safety	Quality score*	Study
Subcortical vascular dementia	*Rivastigmine up to 6 mg daily (104)*<br><br>***vs***<br><br>*Best medical treatment (104)*	*52 weeks (NP)*	***<u>Primary outcomes</u>**:*<br>*None reported*<br><br>*<u>Other outcomes</u>:*<br>*Cognitive: yes*<br>*Functional: no*<br>*Patient-centred: no*	*In favour of treatment*	*Overall rate of AE not significantly different between treatment arms.*<br>*SAE rate not reported.*	**Overall**: Fair<br><br>***QI:***<br>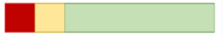	[48]
Subcortical vascular dementia and MID	*Rivastigmine up to 6 mg daily (67)*<br><br>***vs***<br><br>*Best medical treatment (75)*	*104 weeks (NP)*	***<u>Primary outcomes</u>**:*<br>*None reported*<br><br>*<u>Other outcomes</u>:*<br>*Cognitive: yes*<br>*Functional: no*<br>*Patient-centred: no*	*In favour of treatment*	*Overall rate of AE not significantly different between treatment arms.*<br>*SAE rate not reported.*	**Overall**: Fair<br><br>***QI:***<br>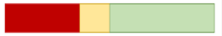	[52]$
Subcortical vascular dementia<br><br>***MID***	*Rivastigmine up to 6 mg daily (50 SVD + 50 MID)*<br><br>***vs***<br><br>*Nimodipine 60 mg (50 SVD + 50 MID)*	*14 months (NP)*	***<u>Primary outcomes</u>**:*<br>*Yes (BEHAVE-AD)*<br><br>*<u>Other outcomes</u>:*<br>*Cognitive: yes*<br>*Functional: no*<br>*Patient-centred: no*	*In favour of treatment (SVD)*<br><br>*Partially in favour of treatment (MID)*	*Overall rate of AE and SAE not significantly different between treatment arms.*	**Overall**: Good<br><br>***QI***:<br>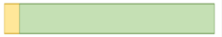	[46]<br>[46bis]
Subcortical vascular dementia	*Rivastigmine up to 6 mg daily*	*16 months (NP)*	***<u>Primary outcomes</u>**:*<br>*None reported*	*In favour of treatment*	*Overall rate of AE (specifically nausea)*	**Overall**: Fair	[47]$

	(32)<br><br>***vs***<br><br>*Nimodipine 60 mg (32)*		*<u>Other outcomes</u>:*<br>*Cognitive: yes*<br>*Functional: yes*<br>*Patient-centred: no*		*increased in active treatment group. No difference in rate of SAE between treatment arms.*	***QI***:	
Subcortical vascular dementia	*Rivastigmine up to 6 mg daily (8)*<br><br>***vs***<br><br>*Best medical treatment (8)*	*22 months (NP)*	***<u>Primary outcomes</u>***:<br>*None reported*<br><br>*<u>Other outcomes</u>:*<br>*Cognitive: yes*<br>*Functional: yes*<br>*Patient-centred: no*	*Partial in favour of treatment*	*No difference in rate of SAE between treatment arm was reported.*	**Overall**: Poor<br><br>***QI***:	[49]$
Vascular dementia	*Memantine 10 mg (11)*<br>***vs***<br>*Rivastigmine 8 mg (11)*<br>***vs***<br>*Galantamine 8 mg (11)*<br>***vs***<br>*Donepezil 8 mg (11)*	*3 months (NP)*	***<u>Primary outcomes</u>***:<br>*None reported*<br><br>*<u>Other outcomes</u>:*<br>*Cognitive: yes*<br>*Functional: no*<br>*Patient-centred: no*<br>*Instrumental: TC-US*	*Partially in favour of treatment*	*Safety outcomes not reported*	**Overall**: Poor<br><br>***QI***:	[42]$

Abbreviations: ADCS-CGIC, Alzheimer's Disease Cooperative Study – Clinical Global Impression of Change; AE, adverse events; BEHAVE-AD, behavioural pathology in Alzheimer's Disease; CDT, Clock Drawing Test; NP, not performed; NPS, neuropsychological scores; SAE, severe adverse events; TC-US, transcranial ultrasound; VaDAS, Vascular Dementia Assessment Scale.

Notes:
*Overall quality as rated according to the NIH Quality Assessment tools for controlled intervention studies is reported here. QI (Quality Index) is a graphical, colour-coded representation of the number of items on the scale rated respectively as at high-risk (red), unclear risk (yellow) or low-risk (green) of bias.
Some of the studies were non-randomised experimental studies; they have been identified with a ($) close to their study number.

2C.3-3 Summary of findings and figures for meta-analyses

Table Caption: Summary of findings for the main comparisons.

Setting: hospital and clinics
Intervention: *rivastigmine (oral tablets)*
Comparator: *placebo*

Outcomes	N° of participants (n of studies)	VCI population label	Efficacy measure	Quality of evidence (GRADE)	Statistical heterogeneity	Studies
Global cognitive efficiency<br>*ADAS-CoG, MMSE*<br><br>***Treatment duration:*** *24-26 weeks*<br><br>***Follow-up after treatment:*** *none*	787 (3 RCTs)	Subcortical vascular dementia, Vascular dementia, post-stroke MCI	**Cohen's d:**<br>-0.01<br>95% CI (-0.30 - 0.29)	⊕○○○ Very Low[3,4,5,6,7]	I^2=39.10	[97]<br>[93]<br>[82]
Functional outcomes<br>*ADCS-ADL, IADL*	799 (3 RCTs)	Subcortical vascular dementia, Vascular dementia, post-stroke MCI	**Cohen's d:**<br>0.04<br>95% CI (-0.10 - 0.18)	⊕○○○Very Low[3,5,7]	I^2=0	[97]<br>[93]<br>[82]
Patient-centred outcomes<br>***Not reported***	See note[1]	See note[1]	*See note[1]*			
Safety outcomes<br>***AE and SAE[3]***	AE:<br>797 (3 RCTs)<br><br><br><br>SA2[3]:	AE:<br>Subcortical vascular dementia, Vascular dementia, post-stroke MCI<br><br>SAE[2]:<br>Post-stroke MCI	**AE (rate ratio):**<br>1.63<br>95% CI (0.67 – 3.96)<br><br><br><br>**SAE (rate ratio)[2]:**	**AE:**<br>⊕○○○Very Low[3,5,6]<br><br><br><br><br>**SAE:**	**AE:** I^2= 81.95	[97]<br>[93]<br>[82]<br><br><br><br>[93]

	50 (1 RCTs)		1.00<br>95% CI (0.29-3.45)	⊕○○○ Very Low[8,9]

Abbreviations: ADAS-CoG, Alzheimer's Disease Assessment Scale – cognitive subscale; ADCS-ADL, Alzheimer's Disease Cooperative Study – Activities of Daily Living; AE, adverse events; 95%CI, 95% Confidence Interval; IADL, Instrumental Activities of Daily Living; MMSE, Mini-Mental State Examination; SAE, severe adverse events.

[1] No studies among the one included in meta-analysis reported data on patient-centred outcomes.
[2] As data on SAE were reported only in [93] the rate ratio reported reflects only the data reported in this single study (no-meta-analysis has been performed for this outcome).

GRADE Working Group grades of evidence:

High certainty: We are very confident that the true effect lies close to that of the estimate of the effect.

Moderate certainty: We are moderately confident in the effect estimate: the true effect is likely to be close to the estimate of the effect, but there is a possibility that it is substantially different.

Low certainty: Our confidence in the effect estimate is limited: the true effect may be substantially different from the estimate of the effect.

Very low certainty: We have very little confidence in the effect estimate: the true effect is likely to be substantially different from the estimate of effect.

---

[3]Low generalisability due to inclusion of different VCI populations (downgraded once for indirectness)
[4]Some inconsistency in point estimates (downgraded once).
[5]Two or more items at uncertain (or greater) risk of bias (downgraded once).
[6]Some imprecision (wide 95% confidence interval, downgraded once).
[7]Different outcome measures employed (downgraded once).
[8]Only one trial reporting on the outcome (downgraded once).
[9]Imprecision (wide 95% confidence interval, downgraded twice)

eFigure 20: Forest plot representing meta-analysis of rivastigmine effect in VCI on global cognitive efficiency outcomes (ADAS-CoG, MMSE). A random effects model has been used for estimation.

	ES	95% CI	W	SE	Sig.	N	N1	N2
Ballard 2008	-0.15	-0.30 / -0.01	61.95%	0.08	0.041	698	360	338
Mok 2007	0.12	-0.51 / 0.75	17.02%	0.32	0.708	39	20	19
Narasimhalu 2010	0.32	-0.22 / 0.87	21.02%	0.28	0.246	50	25	25
Overall (random-effects model)	-0.01	-0.30 / 0.29	100.00%	0.15	0.959	787	405	382

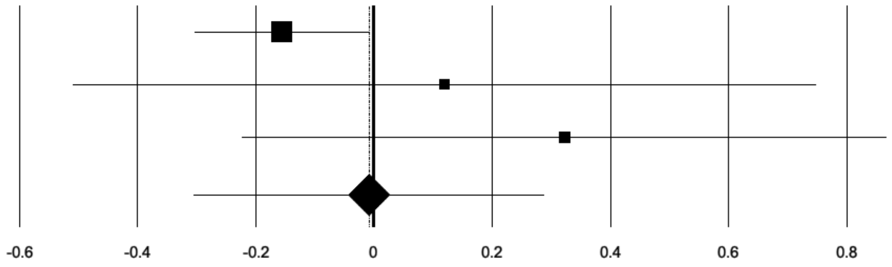

Heterogeneity: Cochran's Q = 3.28, df = 2 (p = 0.194), Tau^2 = 0.03, I^2 = 39.10

eFigure 21: *Forest plot representing meta-analysis of rivastigmine effect in VCI on functional outcomes (ADCS-ADL and). Effect size is reported as Cohen's d; fixed effects model is used for estimation.*

	ES	95% CI	W	SE	Sig.	N	N1	N2
Ballard 2008	0.05	-0.09 / 0.20	88.65%	0.07	0.475	710	365	345
Mok 2007	0.15	-0.48 / 0.78	4.84%	0.32	0.643	39	20	19
Narasimhalu 2010	-0.22	-0.76 / 0.32	6.50%	0.28	0.429	50	25	25
Overall (fixed-effect model)	0.04	-0.10 / 0.18	100.00%	0.07	0.567	799	410	389

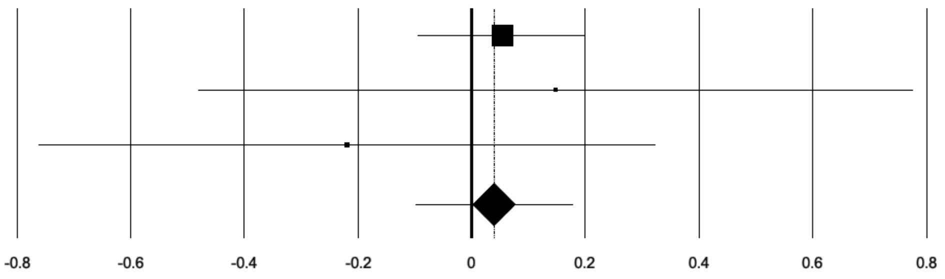

Heterogeneity: Cochran's Q = 1.02, df = 2 (p = 0.600), Tau^2 = 0, I^2 = 0

eFigure 22: *Forest plot representing meta-analysis of rivastigmine effect in VCI on safety outcomes (rate of general adverse events as rate of severe adverse events was reported in only one study [93] it was not meta-analysed). Effect size is reported as rate ratio when not otherwise specified; random effects model is used for estimation.*

	ES	95% CI	W	Sig.
Ballard 2008	3.16	2.51 / 3.97	40.55%	0.000
Mok 2007	1.20	0.52 / 2.78	30.33%	0.670
Narasimhalu 2010	0.90	0.37 / 2.21	29.12%	0.819
Overall (random-effects model)	1.63	0.67 / 3.96	100.00%	0.277

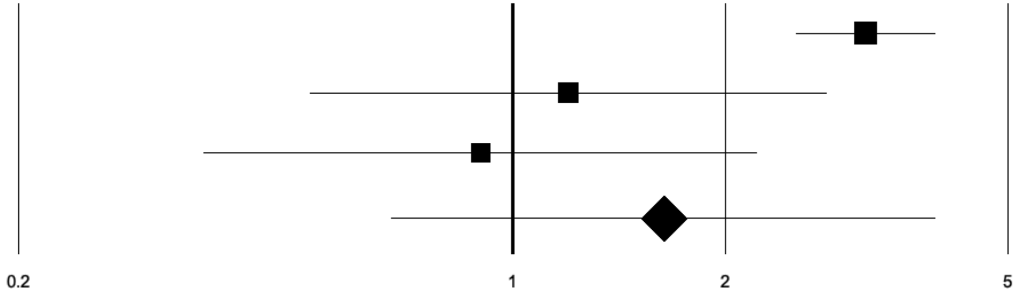

Heterogeneity: Cochran's Q = 11.08, df = 2 (p = 0.004), Tau^2 = 0.49, I^2 = 81.95

2C.3-4 Sensitivity Analyses

We performed sensitivity analyses varying $Corr_{pre\text{-}post}$ for global cognitive efficiency, as $Corr_{pre\text{-}post}$ was not reported nor inferable from previous studies for this class of outcomes. We performed also sensitivity analyses including studies with differing duration of treatment [48], and including studies rated at high risk of bias according to quality rating instruments [49].

Effect size was overall superimposable for $Corr_{pre\text{-}post}$ variation (effect size variation, ***Cohen's d***, + 0.03/-0.01 compared with main analysis). Forest plots for these analyses as well as their relative heterogeneity statistics are reported in *eFigure 23*.

Effect size was overall superimposable when performing meta-analysis including one study [48] with different duration of treatment (12 months compared with a median of 26 weeks; effect size variation for global cognitive efficiency *Cohen's d*, -0.08 compared with main analysis). A further sensitivity analysis including one study [49] at high risk of bias and differing treatment duration (22 months compared to a median of 26 weeks) is reported below. Forest plots for these analyses as well as their relative heterogeneity statistics are reported in *eFigures 24 and 25*, respectively; characteristics of studies included in sensitivity analyses are reported in the *Characteristics of Studies table*.

eFigure 23: *Forest plot representing sensitivity-analyses of rivastigmine effect in VCI on global cognitive efficiency outcomes (ADAS-CoG, MMSE), variating pre-post correlation coefficients between 0 and 0.8 (panel a and panel b respectively). Random effects models were used for estimation.*

a

	ES	95% CI	W	SE	Sig.	N	N1	N2
Ballard 2008	-0.15	-0.30 / -0.01	63.55%	0.08	0.041	698	360	338
Mok 2007	0.09	-0.54 / 0.71	16.26%	0.32	0.789	39	20	19
Narasimhalu 2010	0.32	-0.22 / 0.87	20.19%	0.28	0.246	50	25	25
Overall (random-effects model)	-0.02	-0.30 / 0.27	100.00%	0.15	0.894	787	405	382

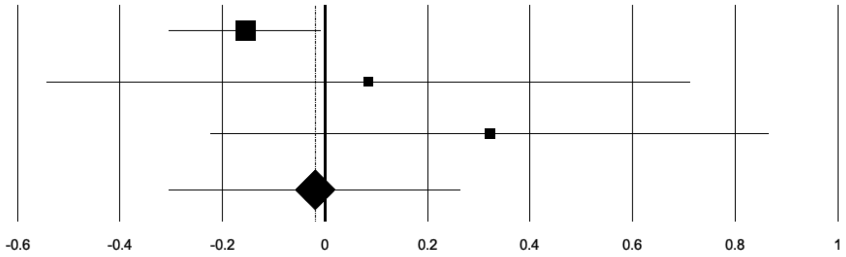

Heterogeneity: Cochran's Q = 3.14, df = 2 (p = 0.204), Tau^2 = 0.03, I^2 = 36.36

b

	ES	95% CI	W	SE	Sig.	N	N1	N2
Ballard 2008	-0.15	-0.30 / -0.01	58.92%	0.08	0.041	698	360	338
Mok 2007	0.18	-0.45 / 0.81	18.49%	0.32	0.568	39	20	19
Narasimhalu 2010	0.32	-0.22 / 0.87	22.59%	0.28	0.246	50	25	25
Overall (random-effects model)	0.02	-0.30 / 0.33	100.00%	0.16	0.924	787	405	382

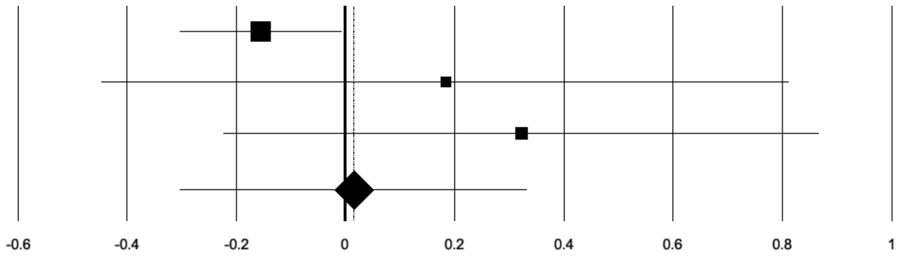

Heterogeneity: Cochran's Q = 3.60, df = 2 (p = 0.165), Tau^2 = 0.04, I^2 = 44.48

***eFigure 24**: Forest plot representing sensitivity-analyses of rivastigmine effect in VCI on functional outcomes (ADCS-ADL, IADL), variating pre-post correlation coefficients between 0 and 0.8 (panel a and panel b respectively); fixed effect models were used for estimation.*

a

	ES	95% CI	W	SE	Sig.	N	N1	N2
Ballard 2008	0.05	-0.09 / 0.20	88.65%	0.07	0.475	710	365	345
Mok 2007	0.11	-0.52 / 0.74	4.85%	0.32	0.738	39	20	19
Narasimhalu 2010	-0.22	-0.76 / 0.32	6.50%	0.28	0.429	50	25	25
Overall (fixed-effect model)	0.04	-0.10 / 0.18	100.00%	0.07	0.586	799	410	389

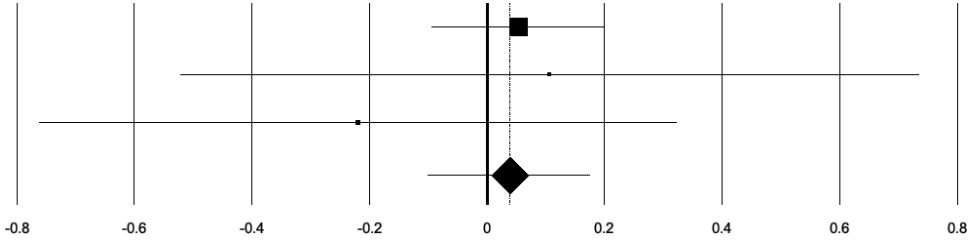

Heterogeneity: Cochran's Q = 0.95, df = 2 (p = 0.622), $Tau^2 = 0$, $I^2 = 0$

b

	ES	95% CI	W	SE	Sig.	N	N1	N2
Ballard 2008	0.05	-0.09 / 0.20	88.67%	0.07	0.475	710	365	345
Mok 2007	0.22	-0.41 / 0.85	4.83%	0.32	0.492	39	20	19
Narasimhalu 2010	-0.22	-0.76 / 0.32	6.51%	0.28	0.429	50	25	25
Overall (fixed-effect model)	0.04	-0.09 / 0.18	100.00%	0.07	0.534	799	410	389

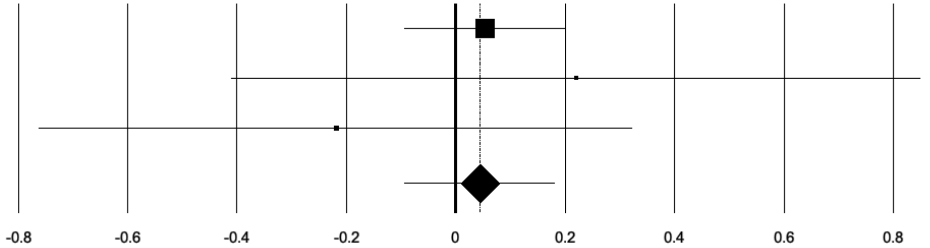

Heterogeneity: Cochran's Q = 1.22, df = 2 (p = 0.543), $Tau^2 = 0$, $I^2 = 0$

__eFigure 25__: Forest plot representing sensitivity-analyses of rivastigmine effect on global cognitive efficiency (panel a) including a study with different treatment duration [48], __panel a__, and non-randomised study rated as at high risk of bias [49], __panel b__. Fixed and random effects model were used for estimation as appropriate.

a

	ES	95% CI	W	SE	Sig.	N	N1	N2
Ballard 2008	-0.15	-0.30 / -0.01	77.44%	0.08	0.041	698	360	338
Mok 2007	0.12	-0.51 / 0.75	4.32%	0.32	0.708	39	20	19
Moretti 2003	-0.05	-0.42 / 0.32	12.49%	0.19	0.802	110	56	54
Narasimhalu 2010	0.32	-0.22 / 0.87	5.75%	0.28	0.246	50	25	25
Overall (fixed-effect model)	-0.10	-0.23 / 0.03	100.00%	0.07	0.126	897	461	436

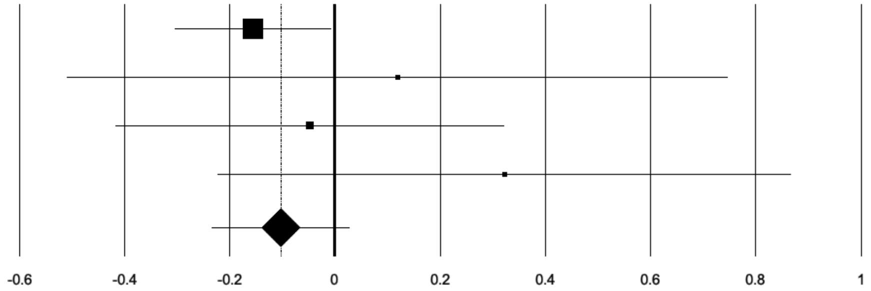

Heterogeneity: Cochran's Q = 3.38, df = 3 (p = 0.337), Tau^2 = 0.00, I^2 = 11.26

b

	ES	95% CI	W	SE	Sig.	N	N1	N2
Ballard 2008	-0.15	-0.30 / -0.01	49.54%	0.08	0.041	698	360	338
Mok 2007	0.12	-0.51 / 0.75	18.26%	0.32	0.708	39	20	19
Moretti 2002	0.60	-0.32 / 1.52	10.31%	0.47	0.202	16	8	8
Narasimhalu 2010	0.32	-0.22 / 0.87	21.89%	0.28	0.246	50	25	25
Overall (random-effects model)	0.08	-0.25 / 0.41	100.00%	0.17	0.643	803	413	390

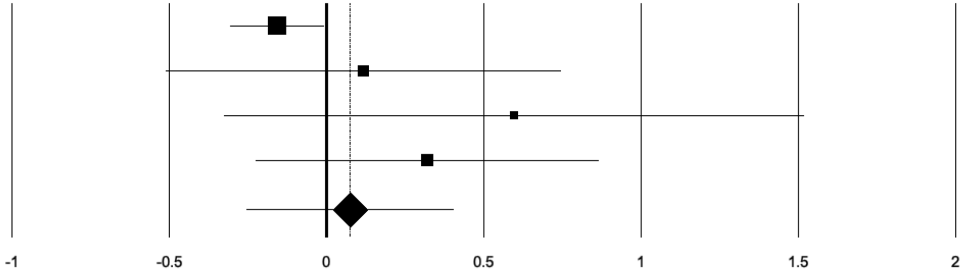

Heterogeneity: Cochran's Q = 5.51, df = 3 (p = 0.138), Tau^2 = 0.05, I^2 = 45.55

2C.4 AcetilCholinesterase Inhibitors – Galantamine

2C.4-1 Description of studies and meta-analysis main results

We retrieved six studies evaluating galantamine as a therapy for vascular cognitive impairment (VCI), see *Characteristics of Studies table*. Five employed it as monotherapy against inactive treatment and one [42] employed it as monotherapy against other active treatments (memantine, rivastigmine, donepezil). Only studies employing galantamine as monotherapy against inactive treatment were included in the meta-analyses.

Among these five, [64] is an open-label extension of [65] and a report of post-hoc analyses, while [35] administered the drug as a single-shot to investigate short-term changes in neuropsychological outcomes and EEG spectral metrics. These two studies were excluded from the meta-analysis.

The median galantamine dose across the remaining three studies was 24 mg. It should be noted that due to the frequent emergence of side effects in the initial phase (with nausea being the most common), a titration phase was incorporated into all study protocols. In some cases, escalation to the highest dose was only possible if no side effects occurred during titration to an intermediate dose (typically 16 mg). The median treatment duration was 26 weeks.

Study [11] did not provide data on any of the meta-analysed outcomes, as it only reported data on specific neuropsychological tests and a functional motor metric that could not be aggregated with other functional measures. Furthermore, it employed a different treatment duration (12 weeks vs 26 weeks) and was assessed as having a high potential risk of bias mainly due to its non-randomised, open-label design featuring a historical control group. It was therefore excluded from the final analyses.

Among the included studies, all reported global cognitive efficiency measures, primarily using the Alzheimer's Disease Assessment Scale-Cognitive Subscale (ADAS-Cog) (n=2). One study reported further neuropsychological evaluations. One study reported functional outcomes (Activities of Daily Living – ADL), and two studies reported data on the Clinical Global Impression of Change – Plus (CIBIC-Plus). No studies reported patient-centred outcomes. All studies included in the final analyses reported data on galantamine safety. Quality rating was good for both studies.

Meta-analysis demonstrated a low effect of galantamine on global cognitive efficiency measures (Cohen's d 0.28, 95% CI 0.15 – 0.41) and no effect on functional outcomes (-0.07, 95% CI -0.21 – 0.08). Rates of adverse events and serious adverse events were not shown to be significantly different between treatment arms.

Results of meta-analyses for each outcome classes are summarised and depicted in the *Summary of Findings table* (along with evidence grading according to the GRADE framework) and in their respective forest plots.

2C.4-2 Characteristic of studies

Table Caption: Characteristics of studies assessing *galantamine* for Vascular Cognitive Impairment.

Setting: hospital and clinics

Intervention: *galantamine (oral tablets)*

Studies included in meta-analysis:

VCI population (label)	Treatment arms	Treatment duration/follow-up	Outcomes	Efficacy	Safety	Quality score*	Study
***Vascular dementia*†**	*Galantamine 24 mg daily (119)*<br><br>*vs*<br><br>*Placebo (69)*	*6 months (NP)*	***<u>Primary outcomes</u>**: Yes (ADAS-CoG, CIBIC-plus)*<br><br>*<u>Other outcomes</u>:*<br>*Cognitive: yes*<br>*Functional: yes*<br>*Patient-centred: no*	*Partially in favour of treatment*	*Overall rate of AE was higher in the treatment group compared to placebo.*<br>*No overall rate of SAE was reported.*	***Overall**: Good*<br><br>***QI**:*<br>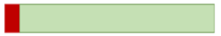	[65]
Vascular dementia	*Galantamine up to 24 mg daily (396)*<br><br>*vs*<br><br>*Placebo (390)*	*26 weeks (NP)*	***<u>Primary outcomes</u>**: Yes (ADAS-CoG)*<br><br>*<u>Other outcomes</u>:*<br>*Cognitive: yes*<br>*Functional: yes*<br>*Patient-centred: no*	*Partially in favour of treatment*	*Overall rate of AE and SAE not significantly different between treatment arms.*	***Overall**: Good*<br><br>***QI:***<br>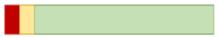	[83]

Studies included only in sensitivity analyses or excluded:

VCI population (label)	Treatment arms	Treatment duration (follow-up)	Outcomes	Efficacy	Safety	Quality score*	Study
Vascular Cognitive Impairment	*Methylphenydate 10 mg*<br><br>*vs*<br><br>*Galantamine 16 mg daily (60)*<br><br>*vs*<br><br>*Placebo (60)*	*1 day (single-shot administration, follow-up: NP)*	***<u>Primary outcomes</u>***: *None reported*<br><br>*<u>Other outcomes</u>: Cognitive: yes Functional: no Patient-centred: no Instrumental: EEG power spectral metrics*	*In favour of treatment (methylphenydate)*	*Overall rate of AE and SAE not reported. Enrichment of treatment-specific AE reported for methylphenydate (hyperarousal) and galantamine (nausea).*	**Overall**: Fair<br><br>**QI**:<br>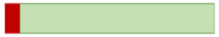	[35]$
Vascular Dementia	*Galantamine 24 mg daily (125)*<br><br>*vs*<br><br>*Placebo (70)*	*6+6 months (follow-up: NP)*<br><br>*6-month open label extension of [65]*	***<u>Primary outcomes</u>***: *Yes (ADAS-CoG)*<br><br>*<u>Other outcomes</u>: Cognitive: no Functional: no Patient-centred: no*	*In favour of treatment*	*Overall rate of AE and not significantly different between treatment arms. No overall rate of SAE was reported.*	**Overall**: Fair<br><br>**QI**:<br>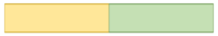	[64]
Post-stroke Cognitive impairment	*Galantamine 24 mg daily (13)*<br><br>*vs*<br><br>*donepezil 10 mg (13)*<br><br>*vs*<br><br>*control (historical comparator, 98)*	*12 weeks (follow-up: NP)*	***<u>Primary outcomes</u>***: *None reported*<br><br>*<u>Other outcomes</u>: Cognitive: yes Functional: yes Patient-centred: no*	*Partially in favour of treatment (donepezil)*	*Safety outcomes analysed but not reported*	**Overall**: Poor<br><br>**QI**:<br>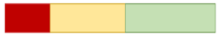	[11]$
Vascular dementia	*Memantine 10 mg (11)*<br><br>***vs***<br><br>*Rivastigmine 8 mg (11)*	*3 months (NP)*	***<u>Primary outcomes</u>***: *None reported*<br><br>*<u>Other outcomes</u>: Cognitive: yes*	*Partially in favour of treatment*	*Safety outcomes not reported*	**Overall**: Poor<br><br>**QI**:<br>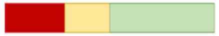	[42]$

	vs *Galantamine 8 mg (11)* ***vs*** *Donepezil 8 mg (11)*	*Functional: no*<br>*Patient-centred: no*<br>*Instrumental: TC-US*		

Abbreviations: ADAS-CoG, Alzheimer's Disease Assessment Scale Cognition, AE, adverse events; CIBIC-Plus,Clinician's Interview-Based Impression of Change (Plus caregiver input); EEG, electroencephalogram; NP, not performed; SAE, severe adverse events, TC-US, transcranial ultrasound.

Notes:
*Overall quality as rated according to the NIH Quality Assessment tools for controlled intervention studies is reported here. QI (Quality Index) is a graphical, colour-coded representation of the number of items on the scale rated respectively as at high-risk (red), unclear risk (yellow) or low-risk (green) of bias.
$Some of the studies were non-randomised experimental studies; they have been identified with a ($) close to their study number.
†This trial included also participants with mixed dementia (i.e., Alzheimer's disease with vascular dementia). Only efficacy results relative to "pure" vascular dementia participants are considered for meta-analysis.

2C.4-3 Summary of findings and figures for meta-analyses

Table Caption: Summary of findings for the main comparisons.

Setting: hospital and clinics
Intervention: *galantamine (oral tablets)*
Comparator: *placebo*

Outcomes	N° of participants (n of studies)	VCI population label	Efficacy measure	Quality of evidence (GRADE)	Statistical heterogeneity	Studies
Global cognitive efficiency *ADAS-CoG*<br><br>***Treatment duration:*** *6 months*<br><br>***Follow-up after treatment:*** *none*	924 (2 RCTs)	*Vascular Dementia*	**Cohen's d:** 0.28<br>95% CI (0.15 – 0.41)	⊕⊕⊕○Moderate[3]	I^2=0	[65]<br>[83]
Functional outcomes *ADCS-ADL*<br><br>***Treatment duration:*** *6 months*<br><br>***Follow-up after treatment:*** *none*	740 (1 RCTs)[1]	*Vascular Dementia*	**Cohen's d[1]:** -0.07<br>95% CI (-0.21 – 0.08)	⊕⊕⊕○Moderate[6]		[83]
Patient-centred outcomes *Not reported*	*See note[2]*	*See note[2]*	See note[2]			
Clinical impression of change: *CIBIC-plus*	922 (2 RCTs)	*Vascular Dementia*	**Cohen's d:** 0.15<br>95% CI (0.00-0.30)	⊕⊕⊕○Moderate[4]		[65]<br>[83]

Treatment duration: *6 months*<br><br>***Follow-up after treatment:*** *none*						
Safety outcomes<br>*AE and SAE*	AE: 974 (2 RCTs)<br><br><br>SAE: 740 (1 RCTs)	*Vascular Dementia*	**AE (rate ratio):**<br>1.23<br>95% CI (0.92 – 1.65)<br><br>**SAE:**<br>1.09<br>95% CI (0.80 – 1.50)	**AE:**<br>⊕⊕○○Low[4,5]<br><br><br>**SAE:**<br>⊕⊕○○Low[5,6]	**AE**: I^2= 80.53<br><br><br>**SAE**: *not applicable*	[65]<br>[83]<br><br>[83]

Abbreviations: ADAS-CoG, Alzheimer's Disease Assessment Scale Cognition, ADCS-ADL, Alzheimer's Disease Cooperative Study – Activities of Daily Living; AE, adverse events; CIBIC-Plus, Clinician's Interview-Based Impression of Change (Plus caregiver input); 95%CI, 95% Confidence Interval; SAE, severe adverse events.

Notes:

[1] As data on SAE were reported only in [83] the rate ratio reported reflects only the data reported in this single study (no-meta-analysis has been performed for this outcome).

[2] No studies among the one included in meta-analysis reported patient-centred outcomes.

GRADE Working Group grades of evidence:

High certainty: We are very confident that the true effect lies close to that of the estimate of the effect.

Moderate certainty: We are moderately confident in the effect estimate: the true effect is likely to be close to the estimate of the effect, but there is a possibility that it is substantially different.

Low certainty: Our confidence in the effect estimate is limited: the true effect may be substantially different from the estimate of the effect.

Very low certainty: We have very little confidence in the effect estimate: the true effect is likely to be substantially different from the estimate of effect.

[3]Some inconsistency in point estimates (downgraded once).

[4]Some imprecision (wide 95% confidence interval, downgraded once).

[5]Downgraded once due to imprecision: the 95% CI includes a result that would not be considered clinically important and a result that would be considered important.

[6]Only one trial reporting on the outcome (downgraded once).

eFigure 26: Forest plot representing meta-analysis of galantamine effect in VCI on global cognitive efficiency outcomes (ADAS-CoG). Panel a represents Cohen's d; a fixed effects model has been used for estimation.

	ES	95% CI	W	SE	Sig.	N	N1	N2
Auchus 2007	0.27	0.12 / 0.41	80.73%	0.07	0.000	736	364	372
Erkinjunnti 2002	0.31	0.01 / 0.61	19.27%	0.15	0.041	188	119	69
Overall (fixed-effect model)	0.28	0.15 / 0.41	100.00%	0.07	0.000	924	483	441

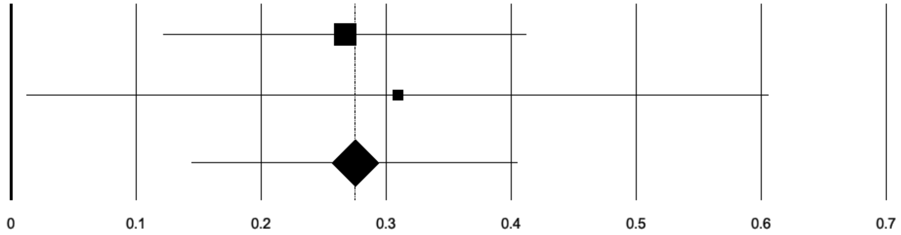

Heterogeneity: Cochran's Q = 0.06, df = 1 (p = 0.801), $Tau^2 = 0$, $I^2 = 0$

__eFigure 27__: Forest plot representing meta-analysis of galantamine effect in VCI on CIBIC-plus. Effect size is reported as Cohen's d; fixed effects model is used for estimation.

	ES	95% CI	W	SE	Sig.	N	N1	N2
Auchus 2007	0.14	-0.03 / 0.30	83.45%	0.09	0.108	734	363	371
Erkinjunnti 2002	0.22	-0.15 / 0.60	16.55%	0.19	0.247	188	119	69
Overall (fixed-effect model)	0.15	-0.00 / 0.30	100.00%	0.08	0.053	922	482	440

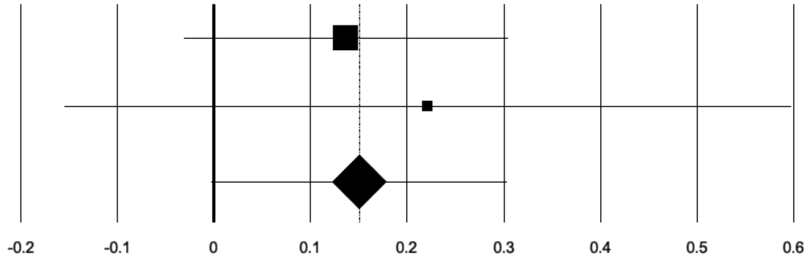

Heterogeneity: Cochran's Q = 0.16, df = 1 (p = 0.687), Tau^2 = 0, I^2 = 0

eFigure 28: *Forest plot representing meta-analysis of galantamine safety in VCI populations (overall rate of adverse events). Effect size is reported as rate ratio when not otherwise specified; random effects model is used for estimation.*

	ES	95% CI	W	Sig.
Auchus 2007	1.07	0.91 / 1.26	52.02%	0.440
Erkinjunnti 2002	1.44	1.18 / 1.76	47.98%	0.000
Overall (random-effects model)	1.23	0.92 / 1.65	100.00%	0.165

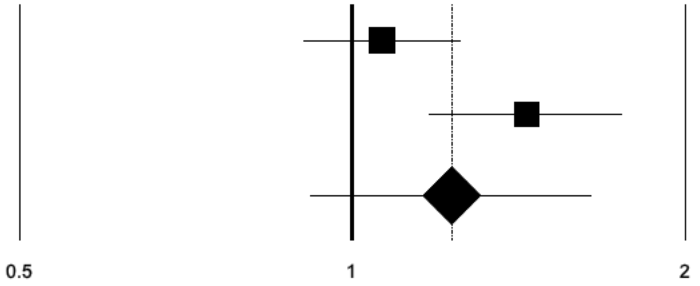

Heterogeneity: Cochran's Q = 5.14, df = 1 (p = 0.023), Tau^2 = 0.04, I^2 = 80.53

2C.5 AcetilCholinesterase Inhibitors – Donepezil

2C.5-1 Description of studies and meta-analysis main results

Eight studies were identified that evaluated donepezil for the treatment of vascular cognitive impairment (VCI). All studies but two [150] and [161], in at least one arm, compared donepezil monotherapy with placebo. One study (study [86]) also included a separate arm testing Tianzhi granules, a traditional Chinese medicine. The median study duration was 24 weeks.

Study [92] was excluded from the primary analysis due to its short treatment period (4 weeks of treatment followed by 4 weeks of follow-up), and study [78] was excluded as it exclusively enrolled patients with CADASIL (Cerebral Autosomal Dominant Arteriopathy with Subcortical Infarcts and Leukoencephalopathy), a specific genetic cause of small vessel disease and stroke-related VCI. These studies were included in sensitivity analyses.

The remaining four studies, included in the final analysis, all assessed the efficacy of donepezil 5 mg. Two of these also evaluated the 10 mg dose. Treatment duration was consistently 24 weeks across all four studies. All studies reported measures of global cognitive function, including ADAS-Cog (2 studies), VaDAS-Cog (2 studies), and MMSE (4 studies). Functional outcomes were assessed using ADFACS (1 study), DAD (2 studies), and ADL (1 study), alongside CIBIC-Plus and safety outcomes. Two studies included further neuropsychological assessments. No study reported patient-centred outcomes. The quality of all studies included in the final analysis was rated as good.

Meta-analysis demonstrated a statistically significant, small effect of donepezil on global cognitive function metrics (Cohen's d: 0.24 to 0.37) and MMSE (Cohen's d: 0.24 to 0.28) across all doses considered. Minimal to negligible effects were observed on functional outcomes (Cohen's d: 0.11 to 0.12), and a small effect was observed on CIBIC-Plus (Cohen's d: 0.06 to 0.28). No significant differences in rates of adverse events or severe adverse events were found between treatment arms. The results of the meta-analyses for each outcome category are summarised and presented in the *Summary of Findings table* and corresponding forest plots (*eFigures 29-35*).

2C.5-2 Characteristic of studies

Table Caption: Characteristics of studies assessing *donepezil* for Vascular Cognitive Impairment.

Setting: hospital and clinics
Intervention: *donepezil (oral tablets)*

Studies included in meta-analysis:

VCI population (label)	Treatment arms	Treatment duration/follow-up	Outcomes	Efficacy	Safety	Quality score*	Study
Vascular dementia	*Donepezil 10 mg daily (215)*<br><br>*vs*<br><br>*Donepezil 5 mg daily (208)*<br><br>*vs*<br><br>*Placebo (193)*	*24 weeks (NP)*	***Primary outcomes**: Yes (ADAS-Cog, CIBIC-Plus)*<br><br>*Other outcomes:*<br>*Cognitive: yes*<br>*Functional: yes*<br>*Patient-centred: no*	*In favour of treatment*	*Overall rate of AE and SAE not significantly different between treatment arms*	**Overall**: Good<br><br>**QI**:<br>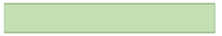	[31]
Vascular dementia	*Donepezil 5 mg daily (398)*<br><br>*vs*<br><br>*Placebo (326)*	*24 weeks (NP)*	***Primary outcomes**: Yes (VaDAS-Cog, CIBIC-Plus)*<br><br>*Other outcomes:*<br>*Cognitive: yes*<br>*Functional: yes*<br>*Patient-centred: no*	*Partially in favour of treatment*	*Overall rate of AE and SAE not significantly different between treatment arms*	**Overall**: Good<br><br>**QI:**<br>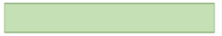	[33]
Vascular dementia	*Donepezil 10 mg daily (216)*<br><br>*vs*<br><br>*Donepezil 5 mg daily (198)*	*24 weeks (NP)*	***Primary outcomes**: Yes (CIBIC-plus)*<br><br>*Other outcomes:*<br>*Cognitive: yes*<br>*Functional: yes*<br>*Patient-centred: no*	*In favour of treatment*	*Overall rate of AE and SAE not significantly different between treatment arms.*	**Overall**: Good<br><br>**QI:**<br>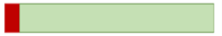	[78]

	vs<br><br>*Placebo (199)*						
Vascular dementia	*Donepezil 5 mg daily (233)*<br><br>*vs*<br><br>*Tianzhi granules 15 g daily (232)*<br><br>*vs*<br><br>*Placebo (55)*	*26 weeks (NP)*	***Primary outcomes**: Yes (VADAS-CoG, CIBIC-Plus)*<br><br>*Other outcomes: Cognitive: yes Functional: yes Patient-centred: no*	*In favour of treatment*	*Overall rate of AE and SAE not significantly different between treatment arms.*	**Overall**: Good<br><br>**QI:**	[86]

Studies included only in sensitivity analyses or excluded:

VCI population (label)	Treatment arms	Treatment duration/follow-up	Outcomes	Efficacy	Safety	Quality score*	Study
Small vessel disease cognitive impairment (CADASIL)	*Donepezil 10 mg daily (86)*<br><br>*vs*<br><br>*Placebo (82)*	*18 weeks (NP)*	***Primary outcomes**: Yes (VaDAS-Cog)*<br><br>*Other outcomes: Cognitive: yes Functional: yes Patient-centred: no*	*Neutral*	*Overall rate of AE and SAE not signifincatly different between treatment arms*	**Overall**: Good<br><br>**QI:**	[67]
Post-stroke MCI	*Donepezil 5 mg daily (7)*<br><br>*vs*<br><br>*Placebo (7)*	*4 weeks (follow-up after treatment: 4 weeks)*	***Primary outcomes**: None reported*<br><br>*Other outcomes: Cognitive: yes Functional: no Patient-centred: no*	*In favour of treatment (donepezil and Tianzhi granules)*	*Overall rate of AE not significantly different between treatment arms. SAE rate not reported.*	**Overall**: Fair<br><br>**QI**:	[92]
Subcortical vascular cognitive impairment	*rTMS, left DLFPC + Donepezil 10 mg (58)*	*4-6 weeks (4 weeks)*	*Primary outcomes Cognitive: MMSE, MoCA*	*In favour of the combined therapy*	*No statistically significant difference in the incidence of*	**Overall**: *Fair*<br><br>**QI:**	[150]

	Vs<br><br>*Donepezil 10 mg (57)*	*[total: 28-42 sessions rTMS]*	*Functional: no*<br>*Patient-centred outcomes: no*<br><br>*No secondary outcomes*		*adverse events in the two groups.*		
Vascular MCI	*Metformin 500 mg + Donepezil 10 mg (48)*<br><br>*Vs*<br><br>*Acarbose 50 mg + Donepezil 10 mg (46)*	*52 weeks (NP)*	*No primary outcomes*<br><br>*Cognitive: yes*<br>*Functional: no*<br>*Patient-centred outcomes: no*<br>*Instrumental: HbA1c, carotid US*	*In favour of treatment*	*No available data on safety*	***Overall**: Good*<br><br>***QI**:*	[161]

Abbreviations: ADAS-CoG, Alzheimer's Disease Assessment Scale cognitive subscale; AE, adverse events; CIBIC-Plus, Clinician's Interview-Based Impression of Change (Plus caregiver input); DFLPC, dorsolateral prefrontal cortex; NP, not performed; rTMS, repetitive transcranial stimulation; SAE, severe adverse events; VaDAS-CoG, Vascular Dementia Assessment Scale cognitive subscale.

Notes:
*Overall quality as rated according to the NIH Quality Assessment tools for controlled intervention studies is reported here. QI (Quality Index) is a graphical, colour-coded representation of the number of items on the scale rated respectively as at high-risk (red), unclear risk (yellow) or low-risk (green) of bias.

2C.5-3 Summary of findings and figures for meta-analyses

Table Caption: Summary of findings for the main comparisons (donepezil all doses)

Setting: hospital and clinics
VCI population: Vascular dementia
Intervention: *donepezil (oral tablets) all doses*
Comparator: *placebo*
Treatment duration: 24 weeks
Follow-up after treatment: none

Outcomes	N° of participants (n of studies)	VCI population label	Efficacy measure	Quality of evidence (GRADE)	Statistical heterogeneity	Studies
Global cognitive efficiency *ADAS-CoG, VaDAS-Cog*<br><br>***Treatment duration:*** *24-26 weeks*<br><br>***Follow-up after treatment:*** *none*	2456 (4 RCTs)	Vascular dementia	**Cohen's d:** 0.29<br>95% CI (0.17-0.41)	⊕⊕⊕○Moderate[2]	I^2= 54.40	[31]<br>[33]<br>[78]<br>[86]
Other cognitive: *MMSE*<br><br>***Treatment duration:*** *24-26 weeks*<br><br>***Follow-up after treatment:*** *none*	2001 (3 RCTs)	Vascular dementia	**Cohen's d:** 0.24<br>95% CI (0.12-0.37)	⊕⊕⊕⊕ High	I^2= 58.97	[33]<br>[78]<br>[86]
Functional outcomes *ADFACS, DAD, ADL*	2456 (4 RCTs)	Vascular dementia	**Cohen's d:** 0.11	⊕⊕⊕○ Moderate[2]	I^2= 0	[31]<br>[33]

Treatment duration:** 24-26 weeks*<br><br>Follow-up after treatment:** none*			95% CI (0.04-0.19)			[78]<br>[86]
Patient-centred outcomes<br>*Not reported*	*See note[1]*	*See note[1]*	*See note[1]*			
Other outcomes: CIBIC-Plus<br><br>***Treatment duration:** 24-26 weeks*<br><br>***Follow-up after treatment:** none*	2456 (4 RCTs)	Vascular dementia	**Cohen's d:**<br>0.23<br>95% CI (0.06-0.39)	⊕⊕⊕⊕ High	I^2= 65.46	[31]<br>[33]<br>[78]<br>[86]
Safety outcomes AE and SAE	AE: 2481 (4 RCTs)<br><br>SAE: 2193 (3 RCTs)	Vascular dementia	**AE (rate ratio):**<br>*1.04*<br>*95% CI (0.96-1.14)*<br><br>**SAE:**<br>1.03<br>*95% CI (0.85-1.26)*	**AE:**<br>⊕⊕⊕⊕ High<br><br>**SAE:**<br>⊕⊕⊕⊕ High	**AE**: I^2= 0<br><br>**SAE**: I^2= 6.77	[31]<br>[33]<br>[78]<br>[86]

Abbreviations: ADAS-Cog, Alzheimer's Disease Assessment Scale cognitive subscale; ADFACS, Alzheimer's Disease Functional Assessment and Change Scale; AE, adverse events; 95%CI, 95% Confidence Interval; CIBIC-Plus, Clinician's Interview-Based Impression of Change (Plus caregiver input); DAD, Disability Assessment for Dementia; MMSE, Mini-Mental State Examination; RCT, randomised controlled trial; SAE, severe adverse events; VaDAS-CoG, Vascular Dementia Assessment Scale cognitive subscale.

Notes:

[1] No studies among the one included in meta-analysis reported patient-centred outcomes.

GRADE Working Group grades of evidence:

High certainty: We are very confident that the true effect lies close to that of the estimate of the effect.

Moderate certainty: We are moderately confident in the effect estimate: the true effect is likely to be close to the estimate of the effect, but there is a possibility that it is substantially different.

Low certainty: Our confidence in the effect estimate is limited: the true effect may be substantially different from the estimate of the effect.

Very low certainty: We have very little confidence in the effect estimate: the true effect is likely to be substantially different from the estimate of effect.

---

[2]Different measures for the same outcomes employed (downgraded once).

Table Caption: Summary of findings for the main comparisons (donepezil 5 mg).

Setting: hospital and clinics
Intervention: *donepezil 5 mg (oral tablets)*
Comparator: *placebo*

Outcomes	N° of participants (n of studies)	VCI population label	Efficacy measure	Quality of evidence (GRADE)	Statistical heterogeneity	Studies
Global cognitive efficiency *ADAS-CoG, VaDAS-Cog*<br><br>***Treatment duration:*** *24-26 weeks*<br><br>***Follow-up after treatment:*** *none*	2035 (4 RCTs)	Vascular dementia	**Cohen's d:**<br>0.24<br>95% CI (0.15-0.33)	⊕⊕⊕○ Moderate[2]	I^2= 33.80	[31]<br>[33]<br>[78]<br>[86]
Other cognitive: *MMSE*<br><br>***Treatment duration:*** *24-26 weeks*<br><br>***Follow-up after treatment:*** *none*	1634 (3 RCTs)	Vascular dementia	**Cohen's d:**<br>0.24<br>95% CI (0.07-0.40)	⊕⊕⊕⊕ High	I^2= 51.43	[33]<br>[78]<br>[86]
Functional outcomes *ADFACS, DAD, ADL*<br><br>***Treatment duration:*** *24-26 weeks*<br><br>***Follow-up after treatment:*** *none*	2035 (4 RCTs)	Vascular dementia	**Cohen's d:**<br>0.11<br>95% CI (0.02-0.20)	⊕⊕⊕○ Moderate[2]	I^2= 0	[31]<br>[33]<br>[78]<br>[86]

Patient-centred outcomes<br>*Not reported*	*See note[1]*	*See note[1]*	*See note[1]*			
Other outcomes: *CIBIC-Plus*<br><br>***Treatment duration:*** *24-26 weeks*<br><br>***Follow-up after treatment:*** *none*	2035 (4 RCTs)	Vascular dementia	**Cohen's d:**<br>0.28<br>95% CI (0.12-0.44)	⊕⊕⊕⊕ High	I^2= 46.35	[31]<br>[33]<br>[78]<br>[86]
Safety outcomes AE and SAE	**AE**: 2060 (4 RCTs)<br><br>**SAE**: 1772 (3 RCTs)	Vascular dementia	**AE (rate ratio):**<br>*1.03*<br>*95% CI (0.93-1.15)*<br><br>**SAE:**<br>0.97<br>*95% CI (0.76-1.25)*	**AE:**<br>⊕⊕⊕⊕ High<br><br>**SAE:**<br>⊕⊕⊕⊕ High	**AE**: I^2= 0<br><br>**SAE**: I^2= 0	[31]<br>[33]<br>[78]<br>[86]<br><br>[31]<br>[33]<br>[78]

Abbreviations: ADAS-Cog, Alzheimer's Disease Assessment Scale cognitive subscale; ADFACS, Alzheimer's Disease Functional Assessment and Change Scale; AE, adverse events; 95%CI, 95% Confidence Interval; CIBIC-Plus, Clinician's Interview-Based Impression of Change (Plus caregiver input); DAD, Disability Assessment for Dementia; MMSE, Mini-Mental State Examination; RCT, randomised controlled trial; SAE, severe adverse events; VaDAS-CoG, Vascular Dementia Assessment Scale cognitive subscale.

Notes:
[1] No studies among the one included in meta-analysis reported patient-centred outcomes.

GRADE Working Group grades of evidence:

High certainty: We are very confident that the true effect lies close to that of the estimate of the effect.

Moderate certainty: We are moderately confident in the effect estimate: the true effect is likely to be close to the estimate of the effect, but there is a possibility that it is substantially different.

Low certainty: Our confidence in the effect estimate is limited: the true effect may be substantially different from the estimate of the effect.

Very low certainty: We have very little confidence in the effect estimate: the true effect is likely to be substantially different from the estimate of effect.

---

[2]Some inconsistency in point estimates (downgraded once).

Table Caption: Summary of findings for the main comparisons (donepezil 10 mg).

Setting: hospital and clinics
Intervention: *donepezil 10 mg (oral tablets)*
Comparator: *placebo*

Outcomes	N° of participants (n of studies)	VCI population label	Efficacy measure	Quality of evidence (GRADE)	Statistical heterogeneity	Studies
Global cognitive efficiency *ADAS-CoG*<br><br>***Treatment duration:*** *26 weeks*<br><br>***Follow-up after treatment:*** *none*	813 (2 RCTs)	Vascular dementia	**Cohen's d:**<br>0.37<br>95% CI (0.23-0.50)	⊕⊕⊕⊕ High	I^2= 54.14	[31]<br>[78]
Other cognitive: *MMSE*<br><br>***Treatment duration:*** *26 weeks*<br><br>***Follow-up after treatment:*** *none*	405 (1 RCTs)	Vascular dementia	**Cohen's d:**<br>0.28<br>95% CI (0.15-0.42)	⊕⊕⊕O Moderate[2]	I^2= 58.97	[78]
Functional outcomes *ADFACS, ADL*<br><br>***Treatment duration:*** *26 weeks*<br><br>***Follow-up after treatment:*** *none*	813 (2 RCTs)	Vascular dementia	**Cohen's d:**<br>0.12<br>95% CI (-0.02-0.26)	⊕⊕⊕O Moderate[3]	I^2= 0	[31]<br>[78]

Patient-centred outcomes<br>*Not reported*	*See note[1]*	*See note[1]*	*See note[1]*			
Other outcomes: *CIBIC-Plus*<br><br>***Treatment duration:*** *26 weeks*<br><br>***Follow-up after treatment:*** *none*	813 (2 RCTs)	Vascular dementia	**Cohen's d:**<br>0.06<br>95% CI (-0.19-0.32)	⊕⊕⊕○ Moderate[3]	I^2= 56.96	[31]<br>[78]
Safety outcomes<br>***AE and SAE***	813 (2 RCTs)	Vascular dementia	**AE (rate ratio):**<br>*1.08*<br>*95% CI (0.94-1.23)*<br><br>**SAE:**<br>1.21<br>*95% CI (0.88-1.64)*	**AE:**<br>⊕⊕⊕⊕ High<br><br>**SAE:**<br>⊕⊕⊕○ Moderate[4]	**AE**: I^2= 0<br><br>**SAE**: I^2= 19.13	[31]<br>[78]

Abbreviations: ADAS-Cog, Alzheimer's Disease Assessment Scale cognitive subscale; ADFACS, Alzheimer's Disease Functional Assessment and Change Scale; AE, adverse events; 95%CI, 95% Confidence Interval; CIBIC-Plus, Clinician's Interview-Based Impression of Change (Plus caregiver input); MMSE, Mini-Mental State Examination; RCT, randomised controlled trial; SAE, severe adverse events.

Notes:

[1] No studies among the one included in meta-analysis reported patient-centred outcomes.

GRADE Working Group grades of evidence:

High certainty: We are very confident that the true effect lies close to that of the estimate of the effect.

Moderate certainty: We are moderately confident in the effect estimate: the true effect is likely to be close to the estimate of the effect, but there is a possibility that it is substantially different.

Low certainty: Our confidence in the effect estimate is limited: the true effect may be substantially different from the estimate of the effect.

Very low certainty: We have very little confidence in the effect estimate: the true effect is likely to be substantially different from the estimate of effect.

GRADE notes:

[2]Only one trial reporting on the outcome (downgraded once).

[3]Some inconsistency in point estimates (downgraded once).

[4]Downgraded once due to imprecision: the 95% CI includes a result that would not be considered clinically important and a result that would be considered important.

eFigure 29: *Forest plot representing meta-analysis of donepezil effect in VCI on global cognitive efficiency outcomes (ADAS-CoG, VaDAS-CoG). Panels **a**, **b**, and **c** represents respectively meta-analysis for all doses combined, donepezil 5 mg and donepezil 10 mg. Effect size has been reported as Cohen's d; random or fixed effect models were used for estimation as appropriate.*

a

	ES	95% CI	W	SE	Sig.	N	N1	N2
Black 2003	0.35	0.21 / 0.49	28.94%	0.07	0.000	802	404	398
Romann 2010	0.14	0.00 / 0.27	29.76%	0.07	0.048	949	628	321
Shi 2020	0.41	0.11 / 0.70	12.16%	0.15	0.007	288	233	55
Wilkinson 2003	0.32	0.19 / 0.46	29.14%	0.07	0.000	809	423	386
Overall (random-effects model)	0.29	0.17 / 0.41	100.00%	0.06	0.000	2848	1688	1160

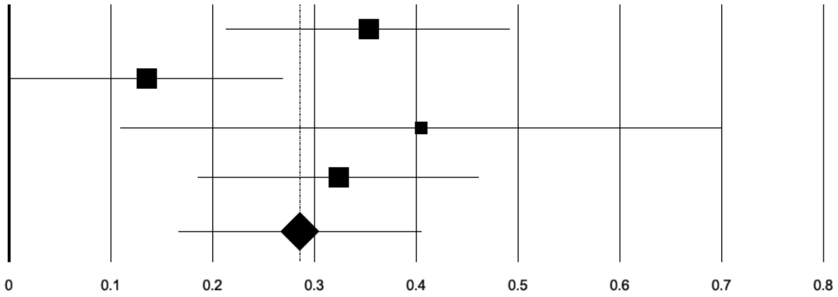

Heterogeneity: Cochran's Q = 6.58, df = 3 (p = 0.087), Tau^2 = 0.01, I^2 = 54.40

b

	ES	95% CI	W	SE	Sig.	N	N1	N2
Black 2003	0.31	0.12 / 0.51	21.58%	0.10	0.002	397	198	199
Romann 2010	0.14	0.00 / 0.27	46.64%	0.07	0.048	949	628	321
Shi 2020	0.41	0.11 / 0.70	9.65%	0.15	0.007	288	233	55
Wilkinson 2003	0.31	0.11 / 0.50	22.12%	0.10	0.002	401	208	193
Overall (fixed-effect model)	0.24	0.15 / 0.33	100.00%	0.05	0.000	2035	1267	768

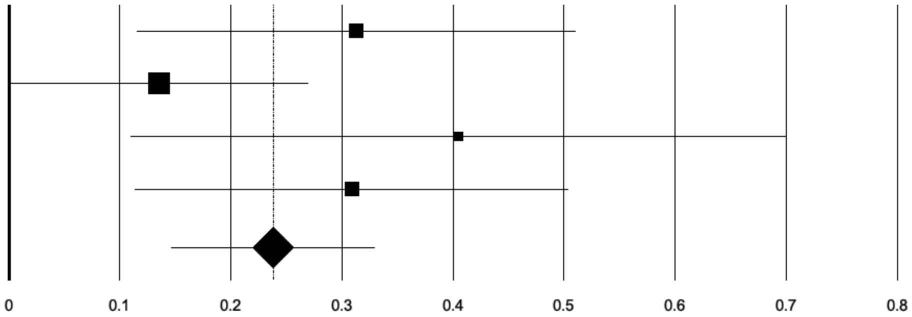

Heterogeneity: Cochran's Q = 4.53, df = 3 (p = 0.210), Tau^2 = 0.00, I^2 = 33.80

c

	ES	95% CI	W	SE	Sig.	N	N1	N2
Black 2003	0.39	0.20 / 0.59	49.72%	0.10	0.000	405	206	199
Wilkinson 2003	0.34	0.14 / 0.53	50.28%	0.10	0.001	408	215	193
Overall (fixed-effect model)	0.37	0.23 / 0.50	100.00%	0.07	0.000	813	421	392

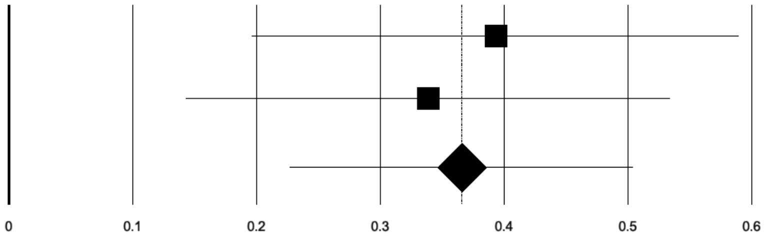

Heterogeneity: Cochran's Q = 0.15, df = 1 (p = 0.700), $Tau^2 = 0$*,* $I^2 = 0$

eFigure 30: *Forest plot representing meta-analysis of donepezil effect in VCI on MMSE. Panels* ***a*** *and* ***b*** *represents respectively meta-analysis for all donepezil doses combined and donepezil 5 mg (no meta-analysis was performed for the highest dose, as only one study reported data on MMSE). Effect size has been reported as Cohen's d; random effects model was used for estimation.*

a

	ES	95% CI	W	SE	Sig.	N	N1	N2
Black 2003	0.28	0.15 / 0.42	40.15%	0.07	0.000	802	404	398
Romann 2010	0.14	0.01 / 0.28	40.97%	0.07	0.035	949	628	321
Shi 2020	0.48	0.19 / 0.78	18.87%	0.15	0.001	288	233	55
Overall (random-effects model)	0.26	0.11 / 0.42	100.00%	0.08	0.001	2039	1265	774

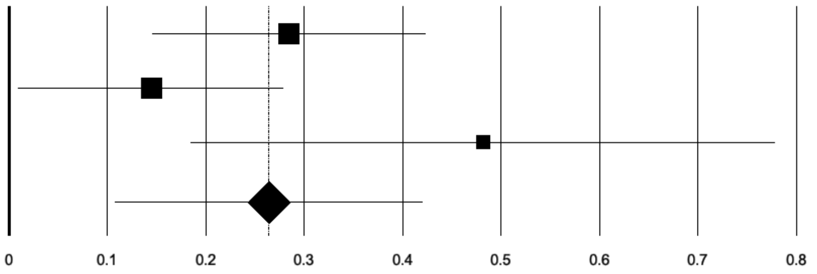

Heterogeneity: Cochran's Q = 4.12, df = 2 (p = 0.128), Tau² = 0.01, I² = 51.43

b

	ES	95% CI	W	SE	Sig.	N	N1	N2
Black 2003	0.21	0.01 / 0.41	33.73%	0.10	0.037	397	198	199
Romann 2010	0.14	0.01 / 0.28	45.37%	0.07	0.035	949	628	321
Shi 2020	0.48	0.19 / 0.78	20.89%	0.15	0.001	288	233	55
Overall (random-effects model)	0.24	0.07 / 0.40	100.00%	0.08	0.005	1634	1059	575

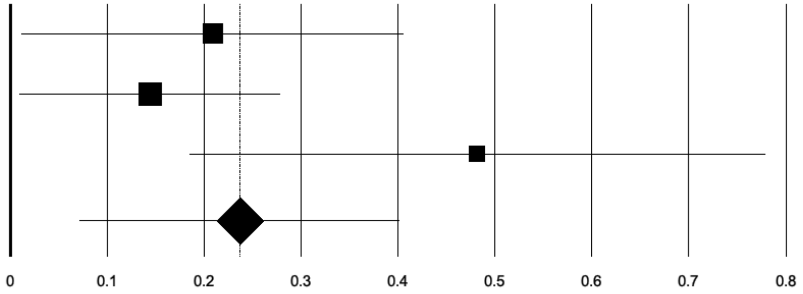

Heterogeneity: Cochran's Q = 4.87, df = 2 (p = 0.087), Tau² = 0.01, I² = 58.97

eFigure 31: *Forest plot representing meta-analysis of donepezil effect in VCI on functional outcomes (ADFACS, DAD, ADL). Panels **a**, **b**, and **c** represents respectively meta-analysis for all doses combined, donepezil 5 mg and donepezil 10 mg. Effect size has been reported as Cohen's d; random or fixed effect models were used for estimation as appropriate.*

a

	ES	95% CI	W	SE	Sig.	N	N1	N2
Black 2003	0.15	0.01 / 0.29	30.40%	0.07	0.031	802	404	398
Romann 2010	0.13	-0.01 / 0.26	32.13%	0.07	0.065	949	628	321
Shi 2020	0.14	-0.15 / 0.44	6.77%	0.15	0.333	288	233	55
Wilkinson 2003	0.05	-0.08 / 0.19	30.70%	0.07	0.444	809	423	386
Overall (fixed-effect model)	0.11	0.04 / 0.19	100.00%	0.04	0.004	2848	1688	1160

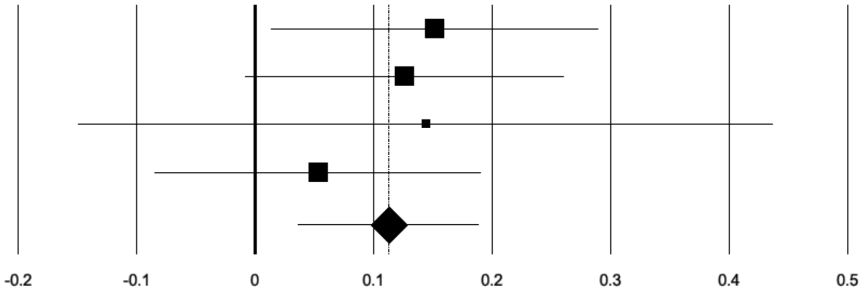

Heterogeneity: Cochran's Q = 1.11, df = 3 (p = 0.774), Tau^2 = 0, I^2 = 0

b

	ES	95% CI	W	SE	Sig.	N	N1	N2
Black 2003	0.15	-0.05 / 0.34	21.76%	0.10	0.148	397	198	199
Romann 2010	0.13	-0.01 / 0.26	46.44%	0.07	0.065	949	628	321
Shi 2020	0.14	-0.15 / 0.44	9.79%	0.15	0.333	288	233	55
Wilkinson 2003	0.03	-0.17 / 0.22	22.02%	0.10	0.794	401	208	193
Overall (fixed-effect model)	0.11	0.02 / 0.20	100.00%	0.05	0.018	2035	1267	768

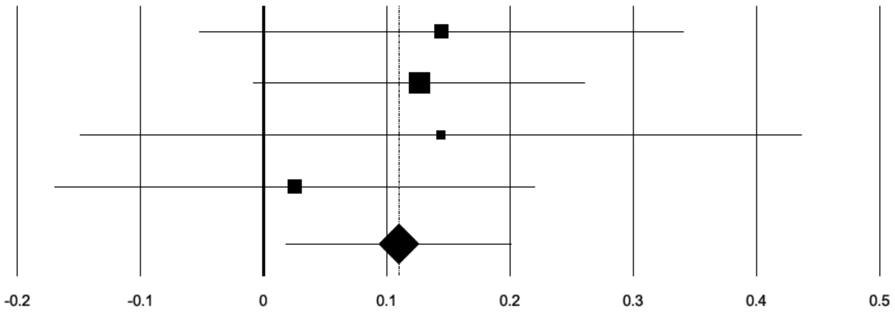

Heterogeneity: Cochran's Q = 0.95, df = 3 (p = 0.814), Tau^2 = 0, I^2 = 0

c

	ES	95% CI	W	SE	Sig.	N	N1	N2
Black 2003	0.16	-0.03 / 0.35	49.80%	0.10	0.107	405	206	199
Wilkinson 2003	0.08	-0.11 / 0.27	50.20%	0.10	0.412	408	215	193
Overall (random-effects model)	0.12	-0.02 / 0.26	100.00%	0.07	0.086	813	421	392

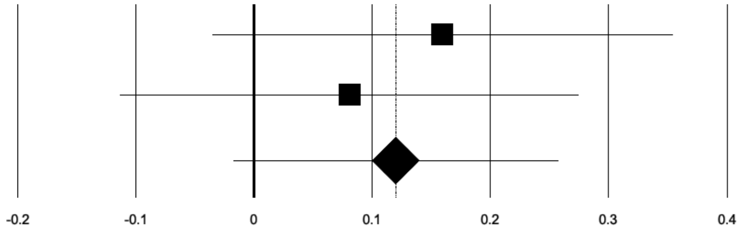

Heterogeneity: Cochran's Q = 0.32, df = 1 (p = 0.574), Tau^2 = 0, I^2 = 0

eFigure 32: *Forest plot representing meta-analysis of donepezil effect in VCI on CIBIC-plus. Panels **a**, **b**, and **c** represents respectively meta-analysis for all doses combined, donepezil 5 mg and donepezil 10 mg. Effect size has been reported as Cohen's d; random effect models have been used for estimations.*

a

Heterogeneity: Cochran's Q = 8.69, df = 3 (p = 0.034), Tau^2 = 0.02, I^2 = 65.46

	ES	95% CI	W	SE	Sig.	N	N1	N2
Black 2003	0.05	-0.12 / 0.22	28.33%	0.09	0.572	802	404	398
Romann 2010	0.18	0.02 / 0.34	28.89%	0.08	0.027	949	628	321
Shi 2020	0.58	0.23 / 0.92	14.58%	0.18	0.001	288	233	55
Wilkinson 2003	0.28	0.11 / 0.45	28.20%	0.09	0.001	809	423	386
Overall (random-effects model)	0.23	0.06 / 0.39	100.00%	0.08	0.006	2848	1688	1160

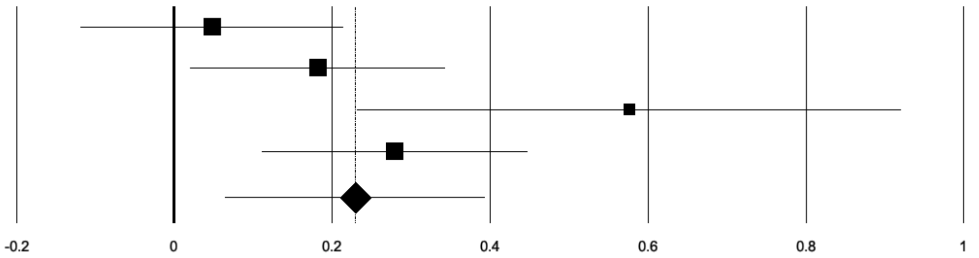

b

	ES	95% CI	W	SE	Sig.	N	N1	N2
Black 2003	0.16	-0.08 / 0.39	25.10%	0.12	0.186	397	198	199
Romann 2010	0.18	0.02 / 0.34	34.95%	0.08	0.027	949	628	321
Shi 2020	0.58	0.23 / 0.92	15.29%	0.18	0.001	288	233	55
Wilkinson 2003	0.36	0.12 / 0.60	24.66%	0.12	0.003	401	208	193
Overall (random-effects model)	0.28	0.12 / 0.44	100.00%	0.08	0.001	2035	1267	768

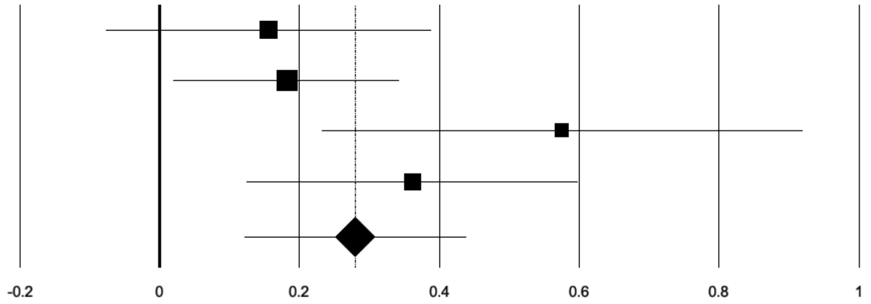

Heterogeneity: Cochran's Q = 5.59, df = 3 (p = 0.133), Tau^2 = 0.01, I^2 = 46.35

c

	ES	95% CI	W	SE	Sig.	N	N1	N2
Black 2003	-0.07	-0.31 / 0.17	49.99%	0.12	0.583	405	206	199
Wilkinson 2003	0.20	-0.04 / 0.44	50.01%	0.12	0.108	408	215	193
Overall (random-effects model)	0.06	-0.19 / 0.32	100.00%	0.13	0.623	813	421	392

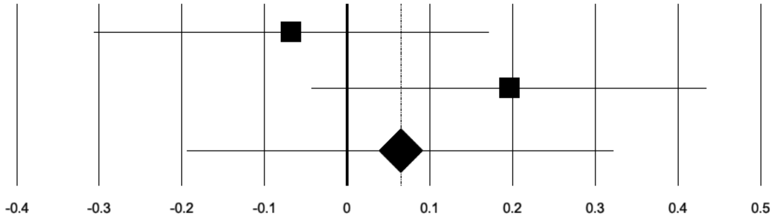

Heterogeneity: Cochran's Q = 2.32, df = 1 (p = 0.127), Tau^2 = 0.02, I^2 = 56.96

***eFigure 33:** Forest plot representing meta-analysis of donepezil safety in VCI populations for all doses combined (adverse events, panel a, and severe adverse events, panel b). Effect size is reported as rate ratio when not otherwise specified; fixed effect models were used for estimation.*

a

	ES	95% CI	W	Sig.
Black 2003	1.04	0.90 / 1.20	34.02%	0.616
Romann 2010	1.04	0.90 / 1.21	32.14%	0.609
Shi 2020	0.71	0.07 / 6.81	0.14%	0.765
Wilkinson 2003	1.05	0.91 / 1.22	33.70%	0.499
Overall (fixed-effect model)	1.04	0.96 / 1.14	100.00%	0.335

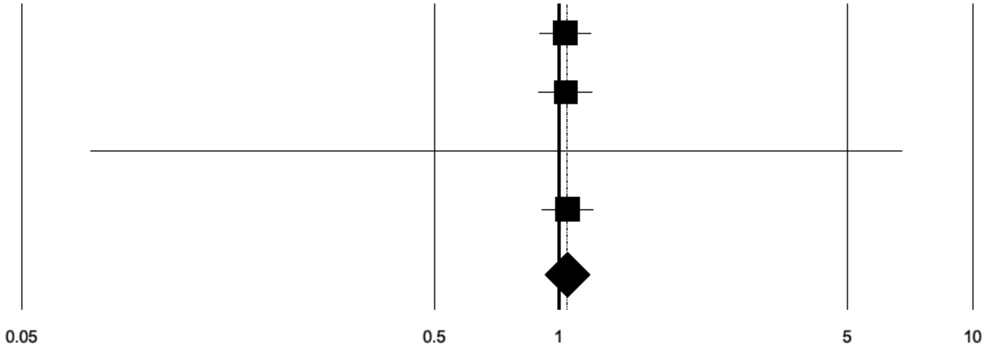

Heterogeneity: Cochran's Q = 0.13, df =3 (p = 0.988), $Tau^2 = 0$, $I^2 = 0$

b

	ES	95% CI	W	Sig.
Black 2003	1.26	0.90 / 1.77	34.24%	0.180
Romann 2010	1.01	0.71 / 1.43	32.04%	0.973
Shi 2020	0.24	0.01 / 3.77	0.51%	0.307
Wilkinson 2003	0.88	0.63 / 1.25	33.21%	0.485
Overall (fixed-effect model)	1.03	0.85 / 1.26	100.00%	0.743

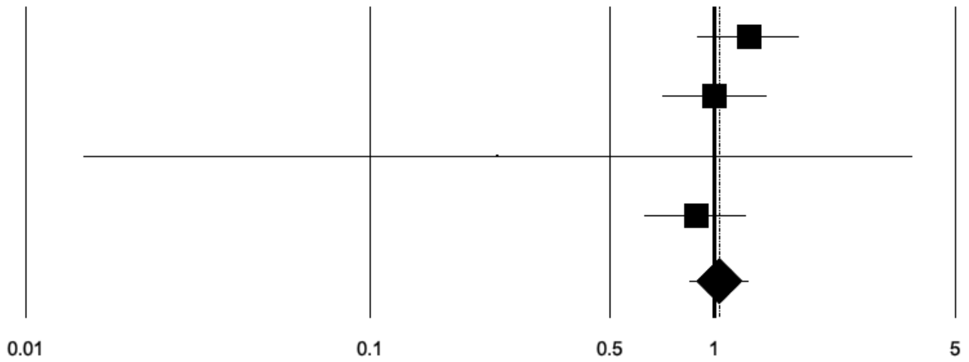

Heterogeneity: Cochran's Q = 3.22, df = 3 (p = 0.359), $Tau^2 = 0$, $I^2 = 6.77$

eFigure 34: *Forest plot representing meta-analysis of donepezil safety in VCI populations for the low (5 mg) dose (adverse events, panel a, and severe adverse events, panel b). Effect size is reported as rate ratio; fixed effects models were used for estimation.*

a

	ES	95% CI	W	Sig.
Black 2003	1.01	0.82 / 1.24	25.31%	0.962
Romann 2010	1.04	0.90 / 1.21	49.04%	0.609
Shi 2020	0.71	0.07 / 6.81	0.22%	0.765
Wilkinson 2003	1.04	0.85 / 1.29	25.44%	0.682
Overall (fixed-effect model)	1.03	0.93 / 1.15	100.00%	0.565

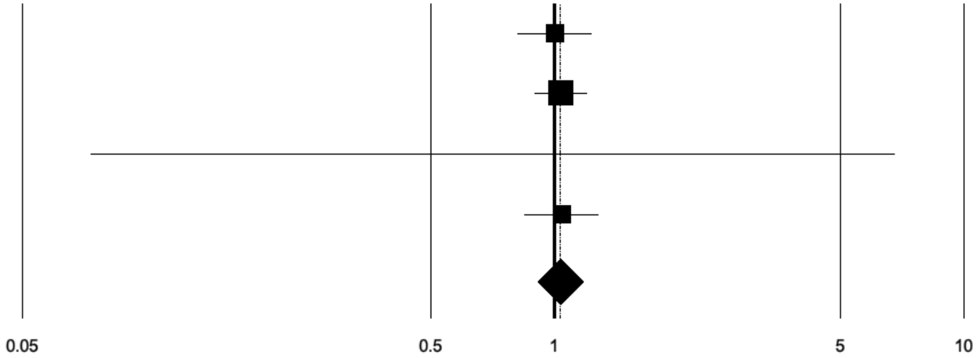

Heterogeneity: Cochran's Q = 0.19, df = 3 (p = 0.979), Tau² = 0, I² = 0

b

	ES	95% CI	W	Sig.
Black 2003	1.07	0.65 / 1.76	24.46%	0.784
Romann 2010	1.01	0.71 / 1.43	49.50%	0.973
Shi 2020	0.24	0.01 / 3.77	0.79%	0.307
Wilkinson 2003	0.87	0.53 / 1.42	25.25%	0.583
Overall (fixed-effect model)	0.97	0.76 / 1.25	100.00%	0.836

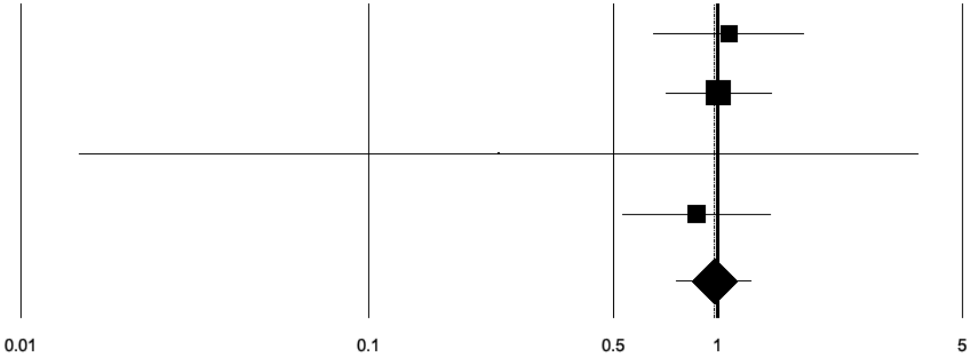

Heterogeneity: Cochran's Q = 1.38, df = 3 (p = 0.711), Tau² = 0, I² = 0

__eFigure 35:__ Forest plot representing meta-analysis of donepezil safety in VCI populations for the high (10 mg) dose (adverse events, panel a, and severe adverse events, panel b). Effect size is reported as rate ratio when not otherwise specified; fixed effects model were used for estimation.

a

	ES	95% CI	W	Sig.
Black 2003	1.01	0.82 / 1.24	25.31%	0.962
Romann 2010	1.04	0.90 / 1.21	49.04%	0.609
Shi 2020	0.71	0.07 / 6.81	0.22%	0.765
Wilkinson 2003	1.04	0.85 / 1.29	25.44%	0.682
Overall (fixed-effect model)	1.03	0.93 / 1.15	100.00%	0.565

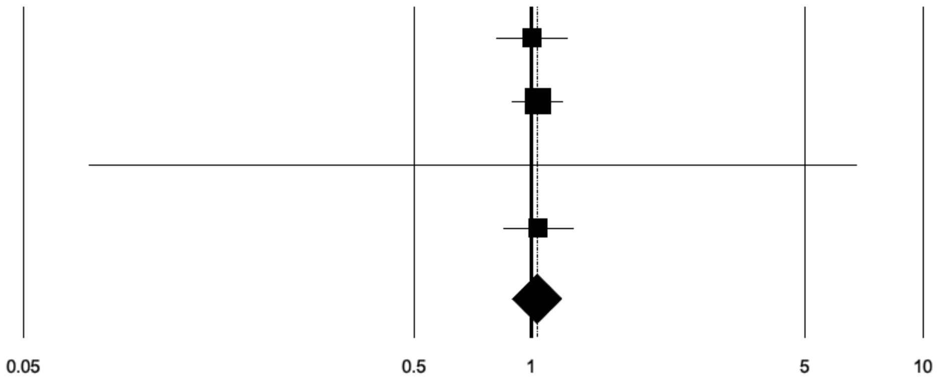

Heterogeneity: Cochran's Q = 0.19, df = 3 (p = 0.979), $Tau^2 = 0$, $I^2 = 0$

b

	ES	95% CI	W	Sig.
Black 2003	1.07	0.65 / 1.76	24.46%	0.784
Romann 2010	1.01	0.71 / 1.43	49.50%	0.973
Shi 2020	0.24	0.01 / 3.77	0.79%	0.307
Wilkinson 2003	0.87	0.53 / 1.42	25.25%	0.583
Overall (fixed-effect model)	0.97	0.76 / 1.25	100.00%	0.836

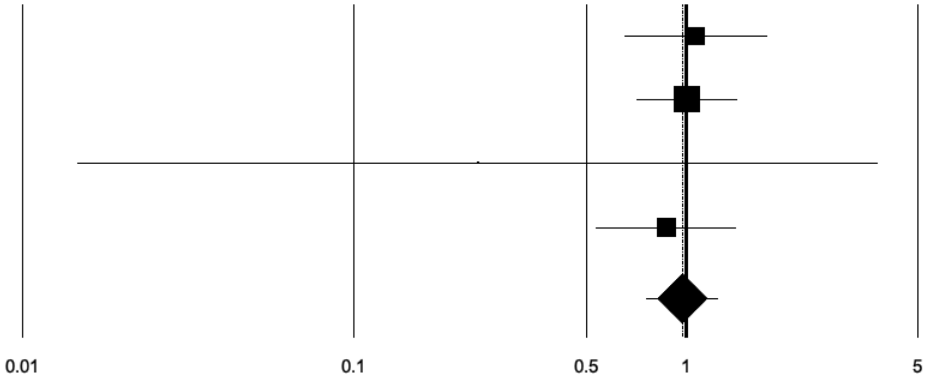

Heterogeneity: Cochran's Q = 1.38, df = 3 (p = 0.711), $Tau^2 = 0$, $I^2 = 0$

2C.5-4 Sensitivity Analyses

Two studies were excluded from the primary analysis and included only in sensitivity analyses. Study [92] was excluded due to its short treatment duration (4 weeks of treatment with 4 weeks of subsequent follow-up). Study [78] was excluded as it included only patients with Cerebral Autosomal Dominant Arteriopathy with Subcortical Infarcts and Leukoencephalopathy (CADASIL), a specific genetic cause of small vessel disease and stroke-related VCI.

Study [92] investigated the effect of donepezil 5 mg in patients with post-stroke mild cognitive impairment (see Table *Characteristics of studies* for further details) and reported data solely for global cognitive function metrics, specifically MMSE. The inclusion of this study in the meta-analysis resulted in largely unchanged effect size estimates (see *eFigure 36* below).

Study [78] investigated the effect of donepezil 10 mg in patients with a genetic diagnosis of CADASIL (see Table *Characteristics of studies*) and reported data for global cognitive function metrics, MMSE, functional outcomes, CIBIC-Plus, and safety outcomes. The inclusion of this study in the meta-analysis resulted in largely unchanged effect size estimates for all evaluated metrics (see *eFigure 37* reported below).

***eFigure 36**: Forest plot representing sensitivity-analyses of donepezil effect in VCI on global cognitive efficiency outcomes (**panel a**) and MMSE (**panel b**), including a study [92] with different treatment duration. Effect sizes are reported as Cohen's d; random effect models have been used for estimation.*

a

	ES	95% CI	W	SE	Sig.	N	N1	N2
Black 2003	0.35	0.21 / 0.49	27.88%	0.07	0.000	802	404	398
Chang 2011	3.45	0.74 / 6.15	0.32%	1.38	0.012	10	6	4
Romann 2010	0.14	0.00 / 0.27	28.33%	0.07	0.048	949	628	321
Shi 2020	0.41	0.11 / 0.70	15.47%	0.15	0.007	288	233	55
Wilkinson 2003	0.32	0.19 / 0.46	27.99%	0.07	0.000	809	423	386
Overall (random-effects model)	0.30	0.15 / 0.46	100.00%	0.08	0.000	2858	1694	1164

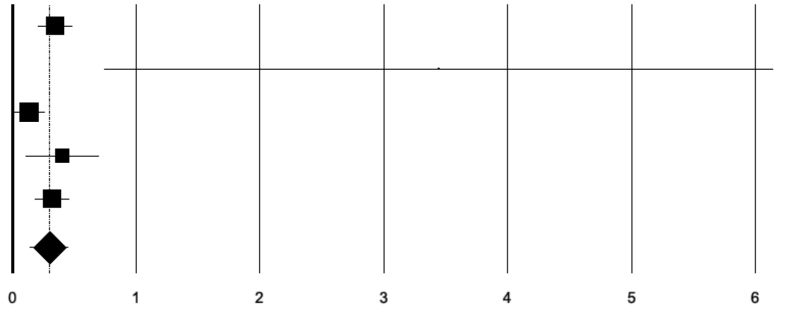

Heterogeneity: Cochran's Q = 0.30, df = 4 (p = 0.018), Tau^2 = 0.02, I^2 = 66.26

b

	ES	95% CI	W	SE	Sig.	N	N1	N2
Black 2003	0.28	0.15 / 0.42	40.15%	0.07	0.000	802	404	398
Romann 2010	0.14	0.01 / 0.28	40.97%	0.07	0.035	949	628	321
Shi 2020	0.48	0.19 / 0.78	18.87%	0.15	0.001	288	233	55
Overall (random-effects model)	0.26	0.11 / 0.42	100.00%	0.08	0.001	2039	1265	774

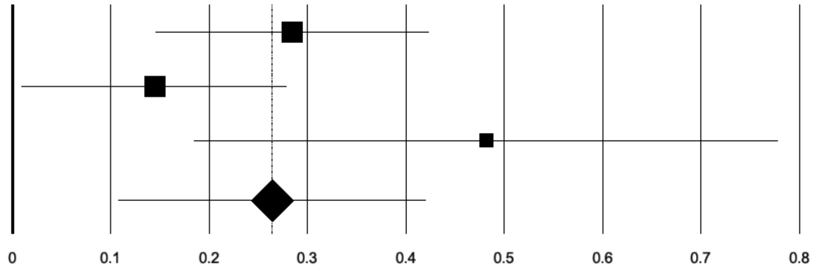

Heterogeneity: Cochran's Q = 10.28, df = 3 (p = 0.016), Tau^2 = 0.03, I^2 = 70.82

***eFigure 37**: Forest plot representing sensitivity-analyses of donepezil effect in VCI on global cognitive efficiency outcomes (panel a), MMSE (panel b), functional outcomes (panel c), and safety outcomes (panel d and e) including a study [78] performed on patients with CADASIL. Effect sizes are reported as Cohen's d for all outcomes except safety outcomes where it is reported as rate ratio; fixed or random effects model were used for estimation as appropriate.*

a

	ES	95% CI	W	SE	Sig.	N	N1	N2
Black 2003	0.35	0.21 / 0.49	25.42%	0.07	0.000	802	404	398
Dichgans 2008	0.01	-0.29 / 0.32	11.20%	0.16	0.932	161	84	77
Romann 2010	0.14	0.00 / 0.27	26.01%	0.07	0.048	949	628	321
Shi 2020	0.41	0.11 / 0.70	11.80%	0.15	0.007	288	233	55
Wilkinson 2003	0.32	0.19 / 0.46	25.56%	0.07	0.000	809	423	386
Overall (random-effects model)	0.26	0.13 / 0.38	100.00%	0.06	0.000	3009	1772	1237

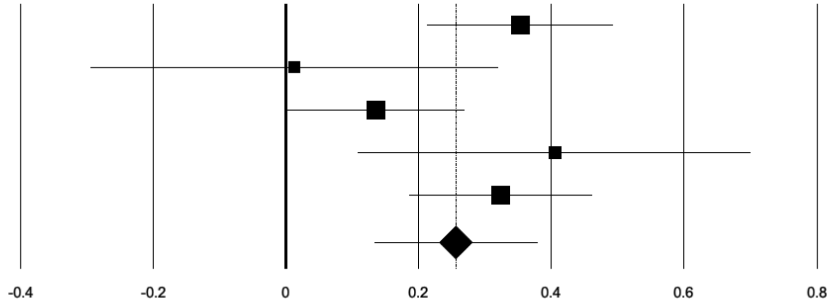

Heterogeneity: Cochran's Q = 9.25, df = 4 (p = 0.055), Tau^2 = 0.01, I^2 = 56.78

b

	ES	95% CI	W	SE	Sig.	N	N1	N2
Black 2003	0.28	0.15 / 0.42	35.79%	0.07	0.000	802	404	398
Dichgans 2008	0.16	-0.15 / 0.47	13.33%	0.16	0.304	161	84	77
Romann 2010	0.14	0.01 / 0.28	36.79%	0.07	0.035	949	628	321
Shi 2020	0.48	0.19 / 0.78	14.08%	0.15	0.001	288	233	55
Overall (random-effects model)	0.24	0.12 / 0.37	100.00%	0.06	0.000	2200	1349	851

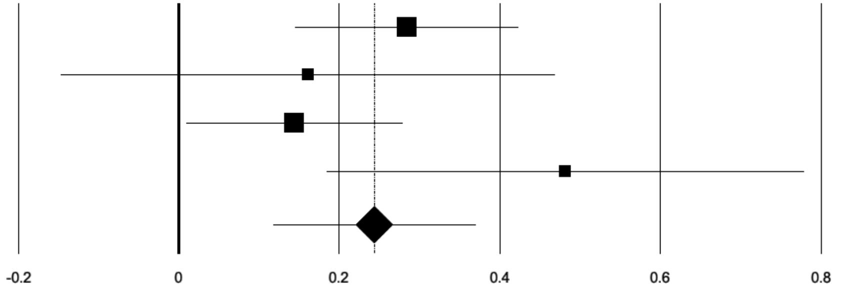

Heterogeneity: Cochran's Q = 5.09, df = 3 (p = 0.165), Tau^2 = 0.01, I^2 = 41.11

c

	ES	95% CI	W	SE	Sig.	N	N1	N2
Black 2003	0.15	0.01 / 0.29	28.63%	0.07	0.031	802	404	398
Dichgans 2008	0.05	-0.25 / 0.36	5.81%	0.16	0.729	161	84	77
Romann 2010	0.13	-0.01 / 0.26	30.27%	0.07	0.065	949	628	321
Shi 2020	0.14	-0.15 / 0.44	6.38%	0.15	0.333	288	233	55
Wilkinson 2003	0.05	-0.08 / 0.19	28.92%	0.07	0.444	809	423	386
Overall (fixed-effect model)	0.11	0.04 / 0.18	100.00%	0.04	0.004	3009	1772	1237

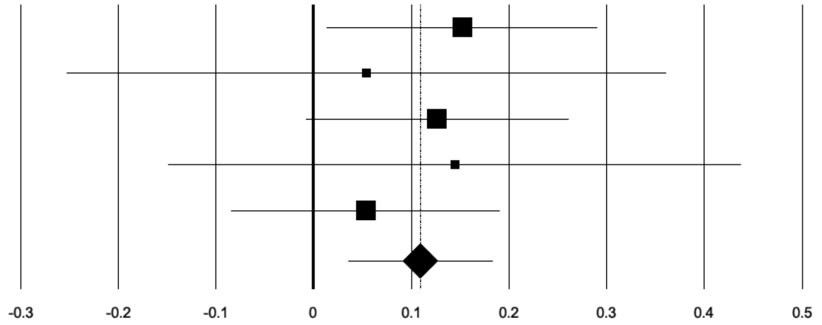

Heterogeneity: Cochran's Q = 1.25, df = 4 (p = 0.870), Tau^2 = 0.00, I^2 = 0.00

d

	ES	95% CI	W	Sig.
Black 2003	1.04	0.90 / 1.20	32.10%	0.616
Dichgans 2008	1.15	0.81 / 1.63	5.64%	0.429
Romann 2010	1.04	0.90 / 1.21	30.32%	0.609
Shi 2020	0.71	0.07 / 6.81	0.13%	0.765
Wilkinson 2003	1.05	0.91 / 1.22	31.80%	0.499
Overall (fixed-effect model)	1.05	0.97 / 1.14	100.00%	0.261

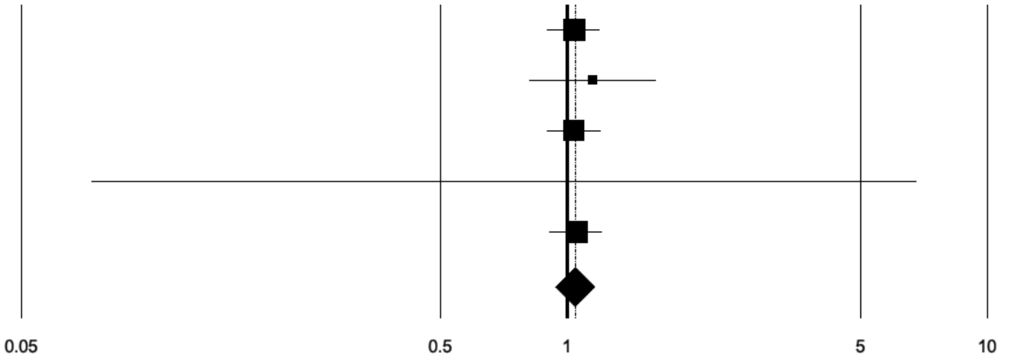

Heterogeneity: Cochran's Q = 0.42, df = 4 (p = 0.981), Tau^2 = 0.00, I^2 = 0.00

e

	ES	95% CI	W	Sig.
Black 2003	1.26	0.90 / 1.77	32.37%	0.180
Dichgans 2008	1.59	0.70 / 3.63	5.44%	0.272
Romann 2010	1.01	0.71 / 1.43	30.30%	0.973
Shi 2020	0.24	0.01 / 3.77	0.48%	0.307
Wilkinson 2003	0.88	0.63 / 1.25	31.41%	0.485
Overall (fixed-effect model)	1.06	0.87 / 1.28	100.00%	0.565

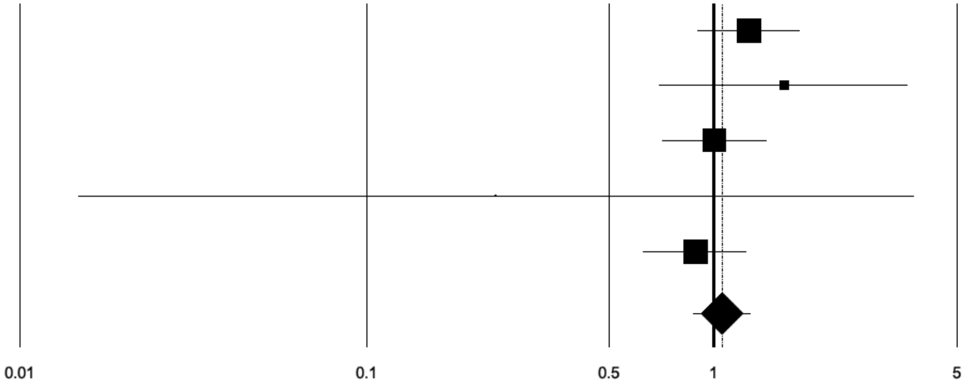

Heterogeneity: Cochran's Q = 4.20, df = 4 (p = 0.379), Tau^2 = 0.00, I^2 = 4.72

2C.6 Memantine

2C.6-1 Description of studies and meta-analysis main results

We retrieved four studies evaluating memantine in the treatment of VCI. Three studies employed memantine monotherapy against placebo, while one study compared it with other active treatments (acetylcholinesterase inhibitors). Only studies evaluating memantine monotherapy were considered for meta-analysis.

Of these three studies, two used a 20 mg dosage, and one used a 10 mg dosage. Treatment duration varied; the studies using the higher dosage assessed the intervention for 28 weeks, while the other assessed it for 12 weeks. Only two studies reported global cognitive function measures (ADAS in both cases). All studies reported functional outcomes (CIBIC-Plus, NOSGER, BGP nursing rating), and none reported patient-centred outcomes. Risk of bias was rated as low in all studies. All studies reported safety outcomes.

The final meta-analysis was performed on the two studies with similar treatment protocols (dose and duration, i.e., [21] and [32]). A sensitivity analysis including the third study was conducted for functional outcomes.

Meta-analysis of global cognitive function outcomes demonstrated a small effect size on global cognitive function metrics (Cohen's d 0.33, 95% CI 0.11–0.55, $p = 0.004$). Meta-analysis of functional outcome data showed no significant difference between memantine and placebo (Cohen's d -0.08, 95% CI -0.25–0.09, $p = 0.37$). Rates of adverse events and severe adverse events did not differ significantly between treatment arms. Results of the meta-analyses for each outcome category are summarised and presented in the *Summary of Findings* table and in corresponding forest plots (*eFigures 38-40*).

2C.6-2 Characteristic of studies

Table Caption: Characteristics of studies assessing *memantine* for Vascular Cognitive Impairment.

Setting: hospital and clinics
Intervention: *memantine (oral tablets)*

Studies included in meta-analysis:

VCI population (label)	Treatment arms	Treatment duration/follow-up	Outcomes	Efficacy	Safety	Quality score*	Study
Vascular dementia	*Memantine 20 mg (147)*<br><br>*vs*<br><br>*Placebo (141)*	*28 weeks (NP)*	***<u>Primary outcomes</u>****:* *Yes (ADAS, CIBIC-plus)*<br><br>*<u>Other outcomes</u>:*<br>*Cognitive: yes*<br>*Functional: yes*<br>*Patient-centred: no*	*In favour of treatment*	*Overall rate of AE and SAE not significantly different between treatment arms*	***Overall***: Good<br><br>***QI***:<br>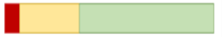	[21]
Vascular dementia	*Memantine 20 mg (143)*<br><br>*vs*<br><br>*Placebo (138)*	*28 weeks (NP)*	***<u>Primary outcomes</u>****:* *Yes (ADAS, CGI-C)*<br><br>*<u>Other outcomes</u>:*<br>*Cognitive: yes*<br>*Functional: yes*<br>*Patient-centred: no*	*Partially in favour of treatment*	*Overall rate of AE and SAE incidence slightly lower in treatment arm compared to placebo.*	***Overall***: Good<br><br>***QI:***<br>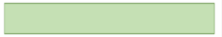	[32]

Studies included only in sensitivity analyses or excluded:

VCI population (label)	Treatment arms	Treatment duration/follow-up	Outcomes	Efficacy	Safety	Quality score*	Study
Vascular dementia	*Memantine 10 mg (41)*<br><br>*vs*<br><br>*Placebo (46)*	*12 weeks (NP)*	***<u>Primary outcomes</u>**:*<br>*Yes (CGI-C, BGP nursing rating)*<br><br>*<u>Other outcomes</u>:*<br>*Cognitive: no*<br>*Functional: no*<br>*Patient-centred: no*	*Partially in favour of treatment*	*Overall rate of AE and SAE not signifincatly different between treatment arms*	**Overall**: Good<br><br>**QI**:	[30]
Vascular dementia	*Memantine 10 mg (11)*<br>***vs***<br>*Rivastigmine 8 mg (11)*<br>***vs***<br>*Galantamine 8 mg (11)*<br>***vs***<br>*Donepezil 8 mg (11)*	*3 months (NP)*	***<u>Primary outcomes</u>**:*<br>*None reported*<br><br>*<u>Other outcomes</u>:*<br>*Cognitive: yes*<br>*Functional: no*<br>*Patient-centred: no*<br>*Instrumental: TC-US*	*Partially in favour of treatment*	*Safety outcomes not reported*	**Overall**: Poor<br><br>**QI**:	[42]$

Abbreviations: ADAS, Alzheimer's Disease Assessment Scale; AE, adverse events; BGP, Beurteilungsskala für Geriatrische Patienten; CGI-C, Clinical Global Impression of Change; CIBIC-plus, Clinician's Interview-Based Impression of Change Plus caregiver input; NOSGER, Nurses Observation Scale for Geriatric Patients; NP, not performed; SAE, severe adverse events; TC-US, transcranial ultrasound parameters.

Notes: * Overall quality, as rated according to the NIH Quality Assessment tools for controlled intervention studies, is reported here. QI (Quality Index) is a graphical, colour-coded representation of the number of items on the scale rated as high-risk (red), unclear risk (yellow) or low-risk (green) of bias.
Some of the studies were non-randomised experimental studies; they have been identified with a ($) close to their study number.

2C.6-3 Summary of findings and figures for meta-analyses

Table Caption: Summary of findings for the main comparisons. *Memantine* for Vascular Cognitive Impairment.

Memantine for Vascular Cognitive impairment

Setting: hospital and clinics
Intervention: *memantine (oral tablets)*
Comparator: *placebo*

Outcomes	N° of participants (n of studies)	VCI population label	Efficacy measure	Quality of evidence (GRADE)	Statistical heterogeneity	Studies
Global cognitive efficiency *ADAS* <br>***Treatment duration:*** *28 weeks* <br>***Follow-up after treatment:*** *none*	*525 (2 RCTs)*	*Vascular Dementia (2 RCT)*	**Cohen's d:** 0.33 95%CI (0.11-0.55)	⊕⊕⊕○ Moderate[2]	I^2= 52.54	[21] [32]
Functional outcomes *NOSGER* <br>***Treatment duration:*** *28 weeks* <br>***Follow-up after treatment:*** *none*	*525 (2 RCTs)*	*Vascular Dementia (2 RCT)*	**Cohen's d:** -0.08 95%CI (-0.25-0.09)	⊕⊕⊕⊕ High	I^2 = 0	[21] [32]
Patient-centred outcomes *Not reported*	*See note[1]*	*See note[1]*	*See note[1]*			

Safety outcomes AE and SAE	*525 (2 RCTs)*	*Vascular Dementia (2 RCT)*	**AE (rate ratio):** 0.92 *95%CI (0.79-1.07)* **SAE:** 0.88 *95%CI (0.42-1.84)*	**AE:** ⊕⊕⊕⊕ High **SAE:** ⊕⊕⊕○ Moderate[2]	**AE:** I^2= 0 **SAE:** I^2= 84.07	[21] [32]

Abbreviations: ADAS, Alzheimer's Disease Assessment Scale; AE, adverse events; NOSGER, Nurses Observation Scale for Geriatric Patients; SAE, severe adverse events.

Notes:
[1] No studies among the one included in meta-analysis reported patient-centred outcomes.

GRADE Working Group grades of evidence:

High certainty: We are very confident that the true effect lies close to that of the estimate of the effect.

Moderate certainty: We are moderately confident in the effect estimate: the true effect is likely to be close to the estimate of the effect, but there is a possibility that it is substantially different.

Low certainty: Our confidence in the effect estimate is limited: the true effect may be substantially different from the estimate of the effect.

Very low certainty: We have very little confidence in the effect estimate: the true effect is likely to be substantially different from the estimate of effect.

[2]Some inconsistency in point estimates (downgraded once).

eFigure 38: *Forest plot representing meta-analysis of memantine on global cognitive efficiency primary outcomes (ADAS). Effect size is reported as Cohen's d; a random effects model was used for estimation.*

	ES	95% CI	W	SE	Sig.	N	N1	N2
Orgogozo 2002	0.47	0.21 / 0.73	40.25%	0.14	0.001	225	111	114
Wilcock 2002	0.24	0.07 / 0.41	59.75%	0.09	0.007	527	266	261
Overall (random-effects model)	0.33	0.11 / 0.55	100.00%	0.11	0.004	752	377	375

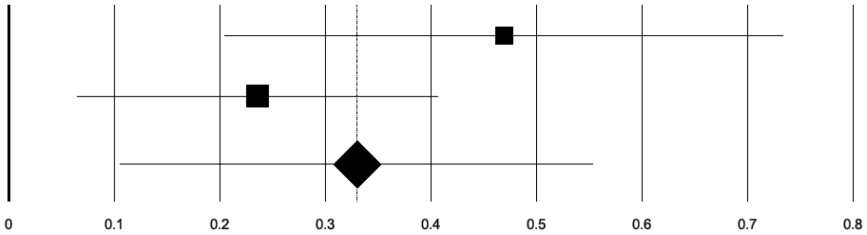

Heterogeneity: Cochran's Q = 2.11, df = 1 (p = 0.15), Tau² = 0.01, I² = 52.54

eFigure 39: *Forest plot representing meta-analysis of nimodipine on functional outcomes (NOSGER). Effect size is reported as Cohen's d; a fixed effects model was used for estimation.*

	ES	95% CI	W	SE	Sig.	N	N1	N2
Orgogozo 2002	-0.04	-0.32 / 0.23	39.29%	0.14	0.757	205	104	101
Wilcock 2002	-0.10	-0.32 / 0.12	60.71%	0.11	0.362	320	171	149
Overall (fixed-effect model)	-0.08	-0.25 / 0.09	100.00%	0.09	0.366	525	275	250

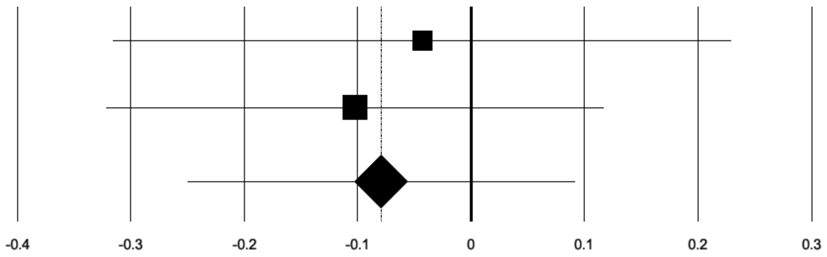

Heterogeneity: Cochran's Q = 0.11, df = 1 (p = 0.74), Tau^2 = 0, I^2 = 0

eFigure 40: *Forest plot representing meta-analysis of the rate of adverse events between intervention arm and placebo (overall rate adverse events, panel a, and rate of severe adverse events, panel b). Effect size is reported as rate ratio when not otherwise specified; fixed- or random-effects models were used for estimation as appropriate.*

a

Heterogeneity: Cochran's Q = 0.06, df = 1 (p = 0.81), Tau² = 0, I² = 0

	ES	95% CI	W	Sig.
Orgogozo 2002	0.95	0.74 / 1.23	35.38%	0.705
Wilcock 2002	0.90	0.75 / 1.09	64.62%	0.286
Overall (fixed-effect model)	0.92	0.79 / 1.07	100.00%	0.279

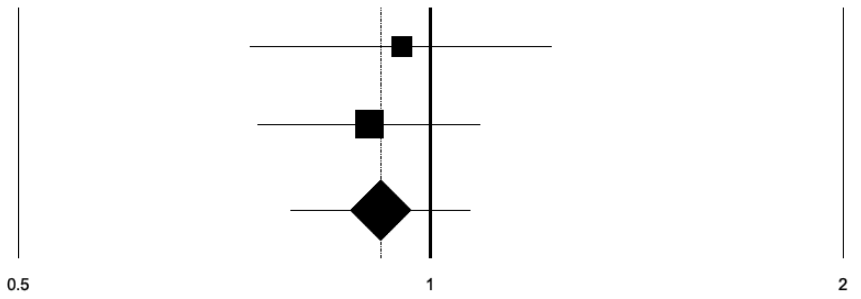

b

	ES	95% CI	W	Sig.
Orgogozo 2002	1.31	0.81 / 2.12	47.11%	0.267
Wilcock 2002	0.62	0.45 / 0.85	52.89%	0.003
Overall (random-effects model)	0.88	0.42 / 1.84	100.00%	0.735

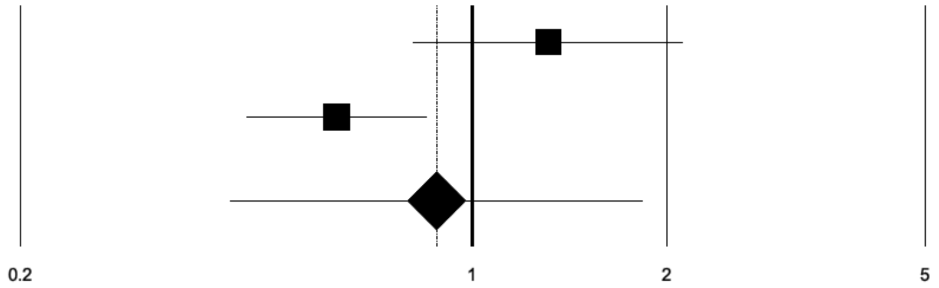

Heterogeneity: Cochran's Q = 6.28, df = 1 (p = 0.012), Tau² = 0.23, I² = 84.07

2C.6-4 Sensitivity Analyses

In sensitivity analyses, we included the study with differing treatment parameters (lower memantine daily dosage, 10 mg instead of 20 mg, and shorter study duration, 12 weeks instead of 28 weeks) and repeated the meta-analysis for functional and safety outcomes.

Effect sizes were comparable between the analyses. Forest plots for these analyses, along with their respective heterogeneity statistics, are presented in *eFigures 41 and 42*, while characteristics of studies included in sensitivity analyses are reported in the

Characteristics of studies table).

***eFigure 41**: Sensitivity analysis showing memantine efficacy on functional outcomes (NOSGER, BGP nursing rating) after including the study with different treatment parameters. Effect size is reported as Cohen's d; a fixed effects model was used for estimation.*

	ES	95% CI	W	SE	Sig.	N	N1	N2
Orgogozo 2002	-0.04	-0.32 / 0.23	29.93%	0.14	0.757	205	104	101
Wilcock 2002	-0.10	-0.32 / 0.12	46.25%	0.11	0.362	320	171	149
Winblad 1999	0.22	-0.09 / 0.52	23.82%	0.16	0.158	166	82	84
Overall (fixed-effect model)	-0.01	-0.16 / 0.14	100.00%	0.08	0.920	691	357	334

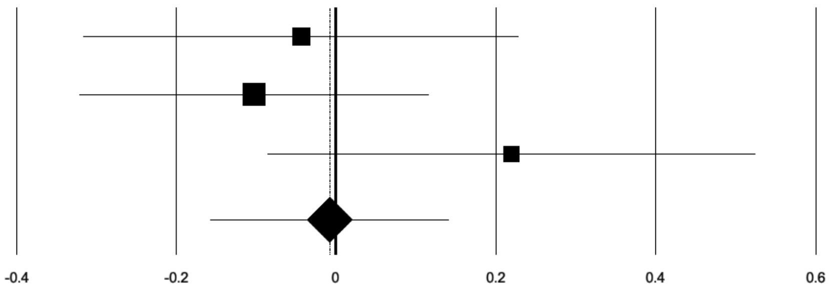

Heterogeneity: Cochran's Q = 2.90, df = 2 (p = 0.234), Tau^2 = 0.01, I^2 = 31.13

eFigure 42: Sensitivity analysis showing the rate of adverse events between intervention arm and placebo (overall rate adverse events, panel a, and rate of severe adverse events, panel b) after including in meta-analysis the study with different treatment parameters. Effect size is reported as rate ratio when not otherwise specified; fixed- or random-effects models were used for estimation as appropriate.

a

	ES	95% CI	W	Sig.
Orgogozo 2002	0.95	0.74 / 1.23	33.60%	0.705
Wilcock 2002	0.90	0.75 / 1.09	61.36%	0.286
Winblad 1999	1.02	0.53 / 1.97	5.05%	0.942
Overall (fixed-effect model)	0.93	0.80 / 1.07	100.00%	0.299

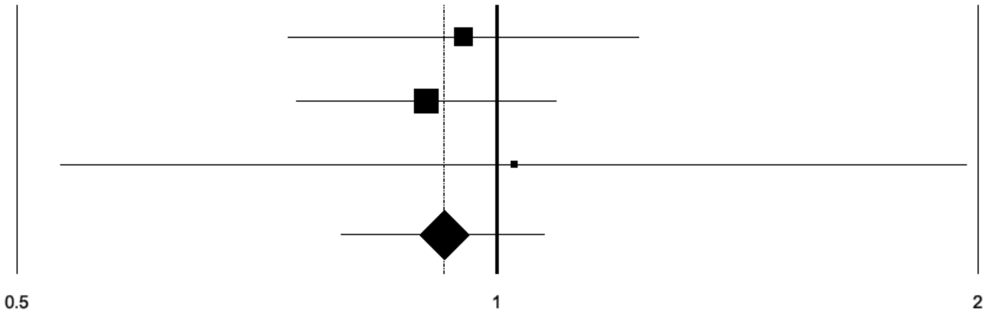

Heterogeneity: Cochran's Q = 0.17, df = 2 (p = 0.92), Tau^2 = 0.01, I^2 = 0

b

	ES	95% CI	W	Sig.
Orgogozo 2002	1.31	0.81 / 2.12	39.42%	0.267
Wilcock 2002	0.62	0.45 / 0.85	45.92%	0.003
Winblad 1999	0.82	0.22 / 3.05	14.66%	0.767
Overall (random-effects model)	0.87	0.48 / 1.57	100.00%	0.635

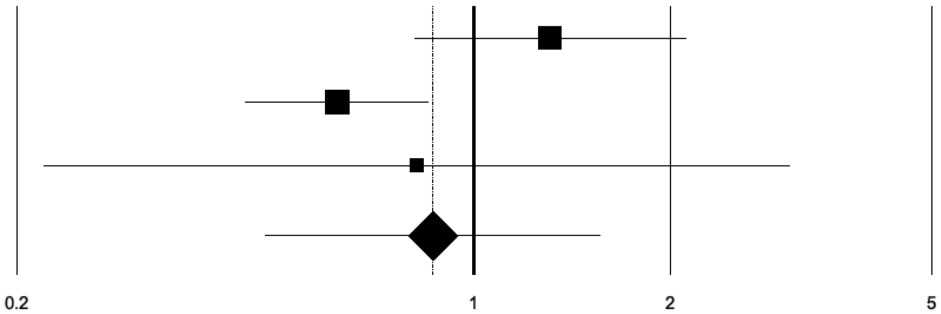

Heterogeneity: Cochran's Q = 6.28, df = 2 (p = 0.043), Tau^2 = 0.16, I^2 = 68.17

2C.7 Pentoxifylline

2C.7-1 Description of studies and meta-analysis main results

We retrieved four studies evaluating pentoxifylline in the treatment of VCI, all employing it as monotherapy against placebo or no treatment.

One study [58] was excluded as it was a preliminary pilot study focusing solely on the efficacy of treatment on surrogate markers (regional cerebral blood flow), and the reported data were insufficient for meta-analysis with the other studies. The remaining three studies used the same cumulative daily dosage (1200 mg). Treatment duration was similar (36–38.5 weeks) except for one study [59], which evaluated the treatment effect over 12 weeks. Study [59] was also rated as having a high risk of bias and was therefore excluded from the main analysis, being re-evaluated only in sensitivity analyses.

The two studies included in the main analysis (i.e., [79] and [81]) reported global cognitive function measures as primary outcomes (ADAS and GBS). No studies reported data on functional or patient-centred outcomes.

Risk of bias was low in both studies included in the main analysis. The overall rate of adverse events was reported, although the rate of severe adverse events was specifically reported only in study [81].

The final meta-analysis was performed on global cognitive function metrics and available safety outcomes (overall rate of adverse events). The meta-analysis showed no significant effect on global cognitive function metrics (Cohen's d 1.00, 95% CI -1.02–3.72, $p > 0.05$), with substantial statistical heterogeneity between studies (I^2 98.74). Rates of adverse events and severe adverse events did not differ significantly between treatment arms. Results of the meta-analyses for each outcome category are summarised and presented in the *Summary of Findings* table and in corresponding forest plots.

2C.7-2 Characteristic of studies

Table Caption: Characteristics of studies assessing *pentoxifylline* for Vascular Cognitive Impairment.

Setting: hospital and clinics
Intervention: *pentoxifylline (oral tablets)*

Studies included in meta-analysis:

VCI population (label)	Treatment arms	Treatment duration/follow-up	Outcomes	Efficacy	Safety	Quality score*	Study
Vascular dementia	*Pentoxifylline 1200 mg, 400 mg/3 times daily (32)*<br><br>*vs*<br><br>*Placebo (32)*	*36 weeks (NP)*	***Primary outcomes**:*<br>*Yes (ADAS)*<br><br>*Other outcomes:*<br>*Cognitive: yes*<br>*Functional: no*<br>*Patient-centred: no*	*In favour of treatment*	*Overall rate of AE and SAE not significantly different between treatment arms*	***Overall**: Good*<br><br>***QI**:*<br>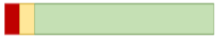	[79]
MID	*Pentoxifylline 1200 mg, 400 mg/3 times daily (137)*<br><br>*vs*<br><br>*Placebo (132)*	*38.5 weeks (NP)*	***Primary outcomes**:*<br>*Yes (GBS)*<br><br>*Other outcomes:*<br>*Cognitive: yes*<br>*Functional: no*<br>*Patient-centred: no*	*Partially in favour of treatment*	*Overall rate of AE not significantly different between treatment arms.*<br>*SAE rate not reported.*	***Overall**: Good*<br><br>***QI:***<br>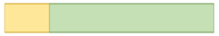	[81]

Studies included only in sensitivity analyses or excluded:

VCI population (label)	Treatment arms	Treatment duration/follow-up	Outcomes	Efficacy	Safety	Quality score*	Study
MID	*Pentoxifylline 1200 mg, 400 mg/3 times daily (4)*<br><br>*vs*<br><br>*Placebo (7)*	*12 weeks (NP)*	***<u>Primary outcomes</u>**:*<br>*None reported*<br><br>*<u>Other outcomes</u>:*<br>*Cognitive: yes*<br>*Functional: no*<br>*Patient-centred: no*	*Partially in favour of treatment*	*Safety outcomes not reported*	**Overall**: Poor<br><br>**QI**:<br>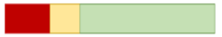	[59]
Vascular dementia	*Pentoxifylline 1200 mg, 400 mg/3 times daily (30)*<br><br>*vs*<br><br>*no treatment (30)*	*8 weeks (NP)*	***<u>Primary outcomes</u>**:*<br>*Yes (rCBF)*<br><br>*<u>Other outcomes</u>:*<br>*Cognitive: no*<br>*Functional: no*<br>*Patient-centred: no*<br>*Instrumental: regional cerebral blood flow*	*In favour of treatment*	*Safety outcomes not reported*	**Overall**: Poor<br><br>**QI**:<br>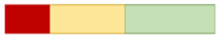	[58]

Abbreviations: ADAS, Alzheimer's Disease Assessment Scale; AE, adverse events; GBS, Göttfries-Brane-Steen scale; NP, not performed; rCBF, regional Cerebral Blood Flow; SAE, severe adverse events.

Notes:
*Overall quality as rated according to the NIH Quality Assessment tools for controlled intervention studies is reported here. QI (Quality Index) is a graphical, colour-coded representation of the number of items on the scale rated respectively as at high-risk (red), unclear risk (yellow) or low-risk (green) of bias.

2C.7-3 Summary of findings and figures for meta-analyses

Table Caption: Summary of findings for the main comparisons.

Setting: hospital and clinics
Intervention: *pentoxifylline (oral tablets)*
Comparator: *placebo*

Outcomes	N° of participants (n of studies)	VCI population label	Efficacy measure	Quality of evidence (GRADE)	Statistical heterogeneity	Studies
Global cognitive efficiency *ADAS, GBS*<br><br>***Treatment duration****: 36-38.5 weeks*<br><br>***Follow-up after treatment:*** *none*	333 (2 RCTs)	Vascular Dementia MID	**Cohen's d:** 1.35 95% CI (-1.02 – 3.72)	⊕○○○ Very Low[3,4,5]	I^2= 98.74	[79] [81]
Functional outcomes *Not reported*	*See note[1]*	*See note[1]*	*See note[1]*			
Patient-centred outcomes *Not reported*	*See note[1]*	*See note[1]*	*See note[1]*			
Safety outcomes *AE and SAE[2]*	AE: 333 (2 RCTs)<br><br>SAE[2]: 269 (1 RCT)	AE: Vascular Dementia - MID<br><br>SAE[2]: MID)	**AE (rate ratio):** 1.63 *95% CI (0.46 – 5.78)*<br><br>**SAE[2]:** *1.13* *95% CI (0.59 – 2.15)*	**AE:** ⊕○○○ Very Low[3,4]<br><br>**SAE:** ⊕○○○ Very Low[3,4,6]	**AE**: I^2= 64.81<br><br>**SAE**: see note[2]	[79] [81]<br><br>[81]

Abbreviations: ADAS, Alzheimer's Disease Assessment Scale; AE, adverse events; 95% CI, 95% confidence interval; GBS, Göttfries-Brane-Steen scale; SAE, severe adverse events.

Notes:

[1] No studies among the one included in meta-analysis reported functional-related or patient-centred outcomes.

[2] As data on SAE were reported only in [81] the rate ratio reported reflects only the data reported in this single study (no-meta-analysis has been performed for this outcome).

GRADE Working Group grades of evidence:

High certainty: We are very confident that the true effect lies close to that of the estimate of the effect.

Moderate certainty: We are moderately confident in the effect estimate: the true effect is likely to be close to the estimate of the effect, but there is a possibility that it is substantially different.

Low certainty: Our confidence in the effect estimate is limited: the true effect may be substantially different from the estimate of the effect.

Very low certainty: We have very little confidence in the effect estimate: the true effect is likely to be substantially different from the estimate of effect.

---

[3]Low generalisability due to inclusion of different VCI populations (downgraded once for indirectness)

[4]Imprecision (wide 95% confidence interval, downgraded twice)

[5]Different outcome measures employed (downgraded once).

[6]Only one trial reporting on the outcome (downgraded once).

eFigure 43: Forest plot representing meta-analysis of pentoxifylline effect on global cognitive efficiency outcomes (ADAS, GBS). Effect size is reported as Cohen's d; a random effects model was used for estimation.

	ES	95% CI	W	SE	Sig.	N	N1	N2
Bayer 1996	0.15	-0.09 / 0.39	50.38%	0.12	0.228	269	137	132
Black 1992	2.57	2.09 / 3.04	49.62%	0.24	0.000	64	32	32
Overall (random-effects model)	1.35	-1.02 / 3.72	100.00%	1.21	0.265	333	169	164

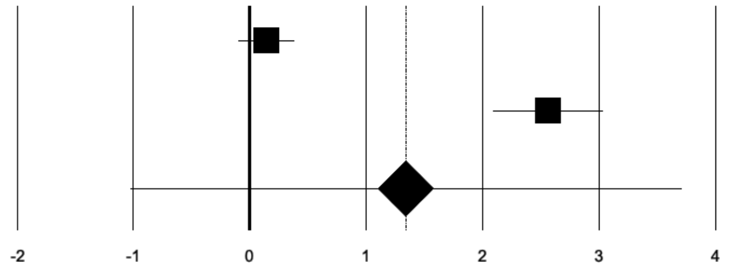

Heterogeneity: Cochran's Q = 79.56, df = 1 (p < 0.0001), Tau^2 = 2.89, I^2 = 98.74

__eFigure 44:__ Forest plot representing meta-analysis of the rate of adverse events between intervention arm and placebo (overall rate of adverse events). Effect size is reported as rate ratio when not otherwise specified; a random effects model was used for estimation.

	ES	95% CI	W	Sig.
Bayer 1996	1.02	0.72 / 1.44	65.92%	0.907
Black 1992	4.00	0.85 / 18.84	34.08%	0.080
Overall (random-effects model)	1.63	0.46 / 5.78	100.00%	0.453

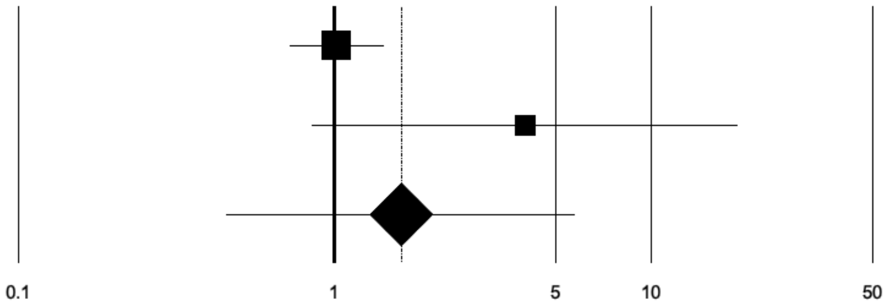

Heterogeneity: Cochran's Q = 2.84, df = 1 (p = 0.092), Tau^2 = 0.60, I^2 = 64.81

2C.7-4 Sensitivity Analyses

We conducted a sensitivity analysis, performing a meta-analysis of global cognitive function outcomes (ADAS, GBS, and SCAG), including one study with a different treatment duration (12 weeks vs. a median of approximately 37 weeks) and rated as having a high risk of bias ([59]). Study [59] also reported MMSE among its cognitive outcomes: unfortunately, however, the data reported in [59] were insufficient for data pooling for this outcome.

The effect size magnitude was largely comparable to the main meta-analysis, showing no statistically significant effect of pentoxifylline on global cognitive function (Cohen's d 1.02, 95% CI -0.82–2.86, $p > 0.05$, $I^2 = 97.50$).

Forest plots for this analysis, along with its complete heterogeneity statistics, are presented in *eFigure 45*. Characteristics of the studies included in the sensitivity analysis are detailed in the Table *Characteristics of Studies*.

***eFigure 45**: Forest plot representing sensitivity-analyses of pentoxifylline effect on global cognitive efficiency (GBS, ADAS, SCAG), including a study with high risk of bias and different treatment duration. Effect size is reported as Cohen's d; a random effects model was used for estimation.*

	ES	95% CI	W	SE	Sig.	N	N1	N2
Bayer 1996	0.15	-0.09 / 0.39	35.08%	0.12	0.228	269	137	132
Black 1992	2.57	2.09 / 3.04	34.48%	0.24	0.000	64	32	32
Ghose 1987	0.28	-0.96 / 1.51	30.44%	0.63	0.659	11	4	7
Overall (random-effects model)	1.02	-0.82 / 2.86	100.00%	0.94	0.276	344	173	171

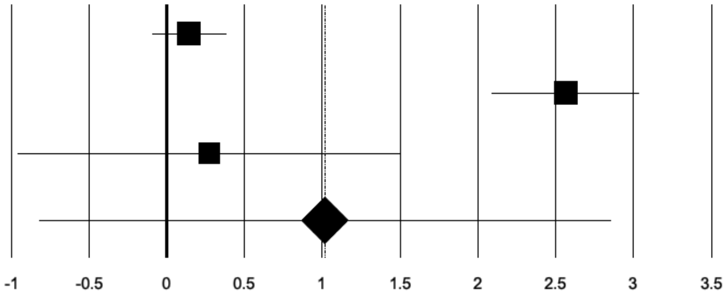

Heterogeneity: Cochran's Q = 79.96, df = 2 (p < 0.0001), Tau^2 = 2.49, I^2 = 97.50

Abbreviations: ADAS, Alzheimer's Disease Assessment Scale; GBS, Göttfries-Brane-Steen scale; SCAG, Sandoz Clinical Assessment-Geriatric scale.

2C.8 Propentofylline

2C.8-1 Description of studies and meta-analysis main results

Three studies evaluating propentofylline monotherapy against placebo were retrieved. Two studies included patients with Alzheimer's dementia alongside those with VCI but reported data separately for the two subgroups. Unfortunately, study [66] did not provide sufficient data for meta-analysis and was therefore excluded.

The two remaining studies tested propentofylline at the same dosage, although over different durations (12 weeks and 52 weeks). Both studies reported global cognitive function metrics (MMSE and SKT). No functional or patient-centred outcomes were reported.

Meta-analysis demonstrated a small to moderate effect size in favour of propentofylline treatment (Cohen’s d 0.44, 95% CI 0.06–0.82, p = 0.024).

Safety outcomes (overall and serious adverse event rates) were not reported for the specific vascular dementia subgroup.

The results of the meta-analysis for global cognitive function are summarised and presented in the *Summary of Findings* table and corresponding forest plot (*eFigure 46*).

2C.8-2 Characteristic of studies

Table Caption: Characteristics of studies assessing *propentofylline* for Vascular Cognitive Impairment.

Setting: hospital and clinics
Intervention: *propentofylline (oral tablets)*

Studies included in meta-analysis:

VCI population (label)	Treatment arms	Treatment duration/follow-up	Outcomes	Efficacy	Safety	Quality score*	Study
Vascular dementia (AD dementia also included but as separate population)	*Propentofylline 900 mg, 300 mg/3 times daily (48)*<br><br>*vs*<br><br>*Placebo (42)*	*52 weeks (NP)*	***Primary outcomes**: Yes (GBS, CGI, SKT)*<br><br>*Other outcomes: Cognitive: no Functional: no Patient-centred: no*	*In favour of treatment*	*Overall rate of AE and SAE not specific for VaD subpopulation.*<br><br>*Rate of overall AE increased for treated patients (RR 2.03 95%CI 1.23-3.35). Rate of SAE overall equal between treatment arms.*	***Overall**: Good*<br><br>***QI**:* 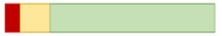	[26]
Vascular dementia	*Pentoxifylline 1200 mg, 400 mg/3 times daily (12)*<br><br>*vs*<br><br>*Placebo (14)*	*12 weeks (NP)*	***Primary outcomes**: No*<br><br>*Other outcomes: Cognitive: yes Functional: no Patient-centred: no Instrumental: FDG-PET regional metabolism*	*Partially in favour of treatment*	*Safety outcomes not reported*	***Overall**: Fair*<br><br>***QI:*** 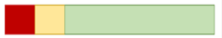	[54]
Vascular dementia	*Pentoxifylline 1200 mg, 400 mg/3 times daily (175)*	*6 months (NP)*	***Primary outcomes**: Yes (GBS, SKT)*	*Partially in favour of treatment*	*Overall rate of AE and SAE not specific for VaD subpopulation.*	***Overall**: Fair*<br><br>***QI**:*	[66]

(AD dementia also included but as separate population)	*vs*<br><br>*Placebo (174)*		*<u>Other outcomes</u>:*<br>*Cognitive: yes*<br>*Functional: yes*<br>*Patient-centred: no*		*Rate of AE slightly increased in treatment arm.*<br>*Rate of SAE not reported.*		

Studies excluded from meta-analysis:

VCI population (label)	Treatment arms	Treatment duration/follow-up	Outcomes	Efficacy	Safety	Quality score*	Study
Vascular dementia (AD dementia also included but as separate population)	*Pentoxifylline 1200 mg, 400 mg/3 times daily (175)*<br><br>*vs*<br><br>*Placebo (174)*	*6 months (NP)*	***<u>Primary outcomes</u>:***<br>*Yes (GBS, SKT)*<br><br>*<u>Other outcomes</u>:*<br>*Cognitive: yes*<br>*Functional: yes*<br>*Patient-centred: no*	*Partially in favour of treatment*	*Overall rate of AE and SAE not specific for VaD subpopulation.*<br><br>*Rate of AE slightly increased in treatment arm.*<br>*Rate of SAE not reported.*	**Overall**: Fair<br><br>***QI***:	[66]

Abbreviations: AE, adverse events; CGI, Clinical Global Impression of change; FDG-PET, fluorodeoxyglucose Positron Emission Tomography; GBS, Göttfries-Brane-Steen scale; NP, not present; SAE, severe adverse events; SKT, Short Cognitive Performance test.

Notes:*Overall quality as rated according to the NIH Quality Assessment tools for controlled intervention studies is reported here. QI (Quality Index) is a graphical, colour-coded representation of the number of items on the scale rated respectively as at high-risk (red), unclear risk (yellow) or low-risk (green) of bias.

2C.8-3 Summary of findings and figures for meta-analyses

Table Caption: Summary of findings for the main comparisons.

Setting: hospital and clinics
Intervention: *propentofylline (oral tablets)*
Comparator: *placebo*

Outcomes	N° of participants (n of studies)	VCI population label	Efficacy measure	Quality of evidence (GRADE)	Statistical heterogeneity	Studies
Global cognitive efficiency *SKT, MMSE*<br><br>***Treatment duration:*** *12 – 52 weeks*<br><br>***Follow-up after treatment:*** *none*	116 (2 RCTs)	Vascular Dementia	**Cohen's d:** 0.44 95% CI (0.06 – 0.82)	⊕⊕○○ Low[3,4]	I^2= 0	[26] [54]
Functional outcomes *Not reported*	*See note[1]*	*See note[1]*	*See note[1]*			
Patient-centred outcomes *Not reported*	*See note[1]*	*See note[1]*	*See note[1]*			
Safety outcomes *AE and SAE*	*See note[2]*	*See note[2]*	*See note[2]*			

Abbreviations: AE, adverse events; MMSE, Mini-Mental State examination; NP, not present; SAE, severe adverse events; SKT, Short Cognitive Performance test.

Notes:
[1] No studies among the one included in meta-analysis reported functional-related or patient-centred outcomes.

[2] Data on overall rate of AE and SAE were not reported for specific VaD subpopulation ([26] and [54] report data aggregated between AD dementia and VaD population).

GRADE Working Group grades of evidence:

High certainty: We are very confident that the true effect lies close to that of the estimate of the effect.

Moderate certainty: We are moderately confident in the effect estimate: the true effect is likely to be close to the estimate of the effect, but there is a possibility that it is substantially different.

Low certainty: Our confidence in the effect estimate is limited: the true effect may be substantially different from the estimate of the effect.

Very low certainty: We have very little confidence in the effect estimate: the true effect is likely to be substantially different from the estimate of effect.

[3]Some imprecision (wide 95% confidence interval, downgraded once).
[4]Different outcome measures employed (downgraded once).

eFigure 46: *Forest plot representing meta-analysis of propentofylline on global cognitive efficiency outcomes (SKT, MMSE). Effect size is reported as Cohen's d; a fixed effects model was used for estimation.*

	ES	95% CI	W	SE	Sig.	N	N1	N2
Marcusson 1997	0.36	-0.08 / 0.81	74.41%	0.23	0.111	77	40	37
Mielke 1996	0.67	-0.09 / 1.43	25.59%	0.39	0.084	26	12	14
Overall (random-effects model)	0.44	0.06 / 0.82	100.00%	0.20	0.024	103	52	51

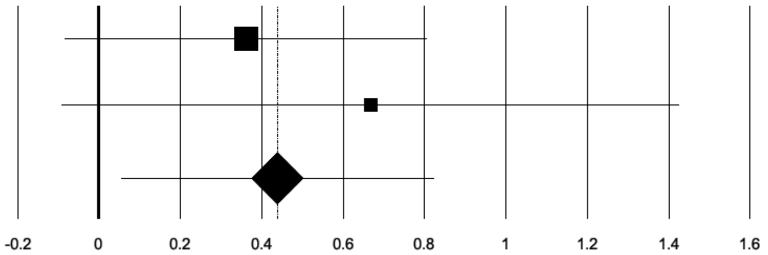

Heterogeneity: Cochran's Q = 0.47, df = 1 (p = 0.494), Tau^2 = 0, I^2 = 0

2C.9 Cerebrolysin

2C.9-1 Description of studies and meta-analysis main results

We retrieved three studies evaluating cerebrolysin® monotherapy in the treatment of VCI. Two studies used the same dosage (30 mL, [29] and the highest treatment arm of [44]), while the third [75] used a lower dosage (20 mL). Study [75] also differed in its administration protocol and treatment duration: it administered two 4-week treatment cycles separated by a 12-week intervention-free interval, compared to the single 4-week cycle used in the other two studies. Study outcomes were measured at the end of treatment in study [29], and eight weeks post-treatment in both [44] and [75]. All studies reported global cognitive function measures: ADAS-Cog (2) and MMSE (2). Two studies also reported further neuropsychological evaluations. Two studies reported functional outcomes (ADCS-ADL and ADL), but none reported patient-centred outcomes. Risk of bias was low for all studies. Safety outcomes were reported only by study [29] and is reported in the final meta-analysis.

The final meta-analysis was performed on studies using similar treatment doses (± 33%) for global cognitive function metrics (i.e., studies [29] and [44]). It demonstrated a small effect size of cerebrolysin® on global cognitive function metrics (Cohen's d 0.35, 95% CI 0.03–0.52, p = 0.027). The results of the meta-analysis for global cognitive function are summarised and presented in the *Summary of Findings table* and corresponding forest plot (*eFigure 47*).

2C.9-2 Characteristic of studies

Table Caption: Characteristics of studies assessing *cerebrolysin®* for Vascular Cognitive Impairment.

Setting: hospital and clinics
Intervention: *cerebrolysin®* (IV solution)

Studies included in meta-analysis:

VCI population (label)	Treatment arms	Treatment duration/follow-up	Outcomes	Efficacy	Safety	Quality score*	Study
Vascular dementia	*Cerebrolysin® 30 mL mg (75)*<br>***vs***<br>*Placebo (72)*	*4 weeks (NP)*	***<u>Primary outcomes</u>:***<br>*Yes (MMSE, CGI)*<br><br>*<u>Other outcomes</u>:*<br>*Cognitive: yes*<br>*Functional: yes*<br>*Patient-centred: no*	*Partially in favour of treatment*	*Comparable rate of AE between treatment and placebo.*<br><br>*No SAE occurrence reported*	***Overall***: Good<br><br>***QI***:<br>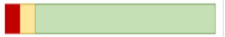	[29]
Vascular dementia	*Cerebrolysin® 30 mL (16)*<br>***vs***<br>*Cerebrolysin® 10 mL (15)*<br>***vs***<br>*Placebo (10)*	*4 weeks (12 weeks)*	***<u>Primary outcomes</u>:***<br>*Yes (ADAS-Cog)*<br><br>*<u>Other outcomes</u>:*<br>*Cognitive: yes*<br>*Functional: no*<br>*Patient-centred: no*<br>*Instrumental (EEG power spectrum)*	*In favour of treatment*	*Comparable rate of AE between treatment and placebo reported*	***Overall***: Good<br><br>***QI***:<br>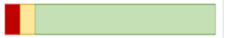	[44]

Studies included only in sensitivity analyses:

VCI population (label)	Treatment arms	Treatment duration/follow-up	Outcomes	Efficacy	Safety	Quality score*	Study
Vascular dementia	*Cerebrolysin® 10 mL (117)*<br>***vs***<br>*Placebo (115)*	*Two 4-week cycles (20 doses each) with 12 weeks interval*<br><br>*Endpoint measurement at 24 weeks from trial beginning*	***<u>Primary outcomes</u>:***<br>*Yes (ADAS-Cog+, CIBIC-plus)*<br><br>*<u>Other outcomes</u>:*<br>*Cognitive: yes*<br>*Functional: yes*<br>*Patient-centred: no*	*In favour of treatment*	*Comparable rate of AE between treatment and placebo.*<br><br>*Three SAE occurred in treatment arm (reported as not plausibly related to cerebrolysin)*	***Overall***: Good<br><br>***QI:***<br>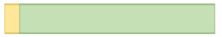	[75]

Abbreviations: ADAS-Cog, Alzheimer's Dementia Assessment Scale – Cognitive subscale; AE, adverse events; CGI, Clinical Global Impression; CIBIC-plus, Clinician's Interview-Based Impression of Change (Plus caregiver input); MMSE, Mini-mental State Exam; SAE, severe adverse events.

Notes: * Overall quality as rated according to the NIH Quality Assessment tools for controlled intervention studies is reported here. QI (Quality Index) is a graphical, colour-coded representation of the number of items on the scale rated respectively as at high-risk (red), unclear risk (yellow) or low-risk (green) of bias.

2C.9-3 Summary of findings and figures for meta-analyses

Table Caption: Summary of findings for the main comparisons.

Setting: hospital and clinics
VCI population: Vascular dementia
Intervention: *cerebrolysin®*
Comparator: placebo

Outcomes	N° of participants (studies)	VCI population (label)	Efficacy measure	Quality of evidence (GRADE)	Statistical heterogeneity	Studies
Global cognitive efficiency *(MMSE, ADAS-CoG)*<br><br>***Treatment duration:*** *4 weeks*<br><br>***Follow-up after treatment:*** *Only 1 RCT reported data after follow-up (not analysed)*	173 (2 RCTs)	Vascular Dementia	**Cohen's d:**<br>0.35<br>95% CI (0.03 – 0.52)	⊕⊕○○ Low[4,5]	I^2= 33.43	[29]<br>[44] (only high dose arm)
Functional outcomes[1] *(ADL)*	147 (1 RCT)[1]	Vascular dementia[1]	**Cohen's d**[1]:<br>0.08<br>95% CI (-0.24 – 0.40)	⊕⊕○○ Low[4,6]	*See note*[1]	[29]
Patient-centred outcomes *None reported*	*See note*[2]	*See note*[2]	*See note*[2]			
Safety outcomes *(AE and SAE)*	147 (1 RCTs)	Vascular Dementia	**AE (rate ratio)**[3]:<br>0.48<br>*95% CI (0.14 – 1.59)*	⊕○○○ Very low[7,8]	*See note*[3]	[29]

Abbreviations: ADAS-CoG, Alzheimer's Disease Assessment Scale – cognitive subscale; ADL, activities of daily living; AE, adverse events; MMSE, Mini-Mental State examination; SAE, severe adverse events.

Notes:

[1]Only 1 RCT included in final analyses [29] reported functional outcomes. The meta-analysis of functional outcomes, including a study with differing dosage and treatment duration [75] is reported within sensitivity analyses.

[2]No RCTs reported data on patient-centred outcomes.

[3]Only 1 RCT included in final analyses [29] reported numerical data for safety outcomes: this rate ratio therefore reflects data from this unique study only. Since no severe adverse events were reported in either group no estimate is provided for severe adverse events. Further analysis of safety outcomes, including data from a study with differing dosage and treatment duration [75] is reported within sensitivity analyses.

GRADE Working Group grades of evidence:

High certainty: We are very confident that the true effect lies close to that of the estimate of the effect.

Moderate certainty: We are moderately confident in the effect estimate: the true effect is likely to be close to the estimate of the effect, but there is a possibility that it is substantially different.

Low certainty: Our confidence in the effect estimate is limited: the true effect may be substantially different from the estimate of the effect.

Very low certainty: We have very little confidence in the effect estimate: the true effect is likely to be substantially different from the estimate of effect.

[4]Some imprecision (wide 95% confidence interval, downgraded once).

[5]Different outcome measures employed (downgraded once).

[6]Some inconsistency in point estimates (downgraded once).

[7]Only one trial reporting on the outcome (downgraded once).

[8]Imprecision (wide 95% confidence interval, downgraded twice)

eFigure 47: *Forest plot representing meta-analysis of cerebrolysin effect in VCI on global cognitive efficiency outcomes (MMSE, ADAS-CoG). Effect size is reported as Cohen's d; a fixed effects model was used for estimation.*

	ES	95% CI	W	SE	Sig.	N	N1	N2
Guekht 2011	0.23	-0.03 / 0.49	91.14%	0.13	0.081	232	117	115
Muresanu High-Dose 2008	0.77	-0.06 / 1.60	8.86%	0.42	0.068	25	15	10
Overall (fixed-effect model)	0.28	0.03 / 0.52	100.00%	0.13	0.027	257	132	125

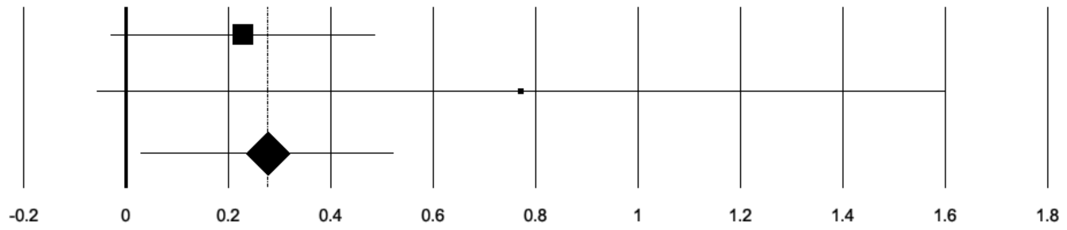

Heterogeneity: Cochran's Q = 1.50, df = 1 (p < 0.220), Tau^2 = 0.05, I^2 = 33.43

2C.9-4 Sensitivity Analyses

We performed sensitivity analyses on global cognitive efficiency outcomes, including study [75], which differed in both dose and treatment protocol, and the low-dose arm of study [44]. The effect size magnitude was largely comparable (Cohen's d = 0.42, 95% CI 0.13–0.71, compared to 0.35, 95% CI 0.03–0.52 in the main analysis).

Meta-analysis of the effect of cerebrolysin® on functional outcomes showed no significant effect (Cohen's d = 0.43, 95% CI -0.25–1.11, $p > 0.05$). Rates of adverse events were also not significantly more prevalent in the treatment group compared to placebo (Rate ratio 1.37, 95% CI 0.20–9.58, $p > 0.05$). Substantial statistical heterogeneity was observed in both analyses (I^2 = 90.30 and 86.87, respectively).

Forest plots for these analyses, along with their complete heterogeneity statistics, are presented in *eFigures 48-49*. Characteristics of the studies included in the sensitivity analyses are detailed in the *Characteristics of Studies* table.

***eFigure 48**: Forest plot representing sensitivity-analyses of Gingko biloba effect on global cognitive efficiency (MMSE, ADAS-Cog, ADAS-Cog+) including a study with different treatment dosage/duration ([76]) and the low-dose arm of study [44]. Effect size is expressed as Cohen's d; a fixed effects model was used for estimation.*

	ES	95% CI	W	SE	Sig.	N	N1	N2
Guekht 2011	0.23	-0.03 / 0.49	55.02%	0.13	0.081	232	117	115
Muresanu High-Dose 2008	0.77	-0.06 / 1.60	5.35%	0.42	0.068	25	15	10
Muresanu Low-Dose 2008	1.11	0.26 / 1.95	5.13%	0.43	0.010	26	16	10
Xiao 1999	0.37	0.04 / 0.69	34.50%	0.17	0.028	147	75	72
Overall (fixed-effect model)	0.35	0.16 / 0.54	100.00%	0.10	0.000	430	223	207

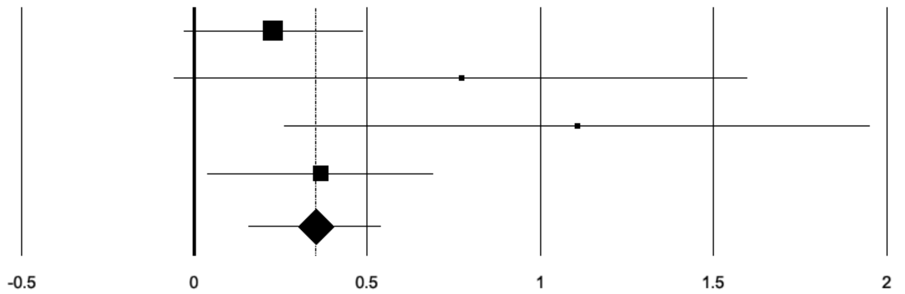

Heterogeneity: Cochran's Q = 4.93, df = 3 (p = 0.177), Tau^2 = 0.03, I^2 = 39.11

***eFigure 49**: Forest plot representing metanalysis of cerebrolysin effect on functional outcomes (Alzheimer’s Disease Cooperative Study-Activities of Daily Living, ADCS-ADL, and Activities of Daily Living scale, **panel a**) and safety outcomes (rate of adverse events, **panel b**) including a study with different treatment dosage/duration [76] and the low-dose arm of study [44]. Effect size is reported as Cohen’s d for global cognitive efficiency and as rate ratio for adverse events rate; random-effect models were used for estimation.*

a

	ES	95% CI	W	SE	Sig.	N	N1	N2
Guekht 2011	0.08	-0.24 / 0.40	49.14%	0.17	0.630	147	75	72
Xiao 1999	0.77	0.50 / 1.04	50.86%	0.14	0.000	229	117	112
Overall (random-effects model)	0.43	-0.25 / 1.11	100.00%	0.35	0.212	376	192	184

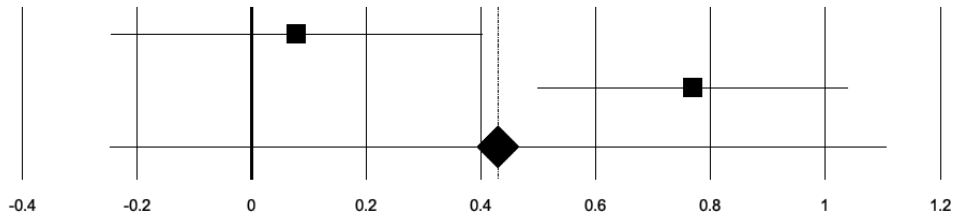

Heterogeneity: Cochran’s Q = 10.31, df = 1 (p = 0.001), Tau^2 = 0.22, I^2 = 90.30

b

	ES	95% CI	W	Sig.
Guekht 2011	0.48	0.14 / 1.59	47.05%	0.231
Xiao 1999	3.49	1.67 / 7.32	52.95%	0.001
Overall (random-effects model)	1.37	0.20 / 9.58	100.00%	0.749

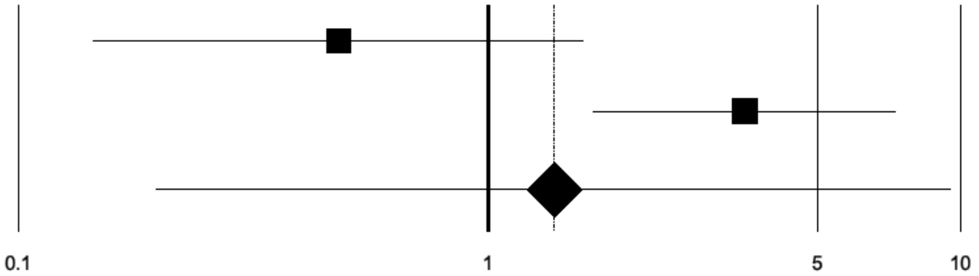

Heterogeneity: Cochran’s Q = 7.62, df = 1 (p = 0.006), Tau^2 = 1.71, I^2 = 86.87

2C.10 Nimodipine

2C.10-1 Description of studies and meta-analysis main results

We retrieved six randomised controlled trials (RCTs) evaluating nimodipine for the treatment of vascular cognitive impairment (VCI). Three trials assessed nimodipine as monotherapy compared with placebo, while the remaining three evaluated it in combination with other pharmacological treatments or acupuncture.

Of the three studies investigating monotherapy ([17], [18], [172]), one included a published post-hoc analysis [16] that stratified participants by VCI subtype (multi-infarct dementia and subcortical ischaemic dementia). As a result, study [18] was separated into two separate entries for the meta-analysis.

All studies employed the same dosage regimen (nimodipine 90 mg daily, administered as 30 mg three times per day), though treatment durations varied: study [18] evaluated it over 26 weeks, study [172] over six months, and study [17] over 52 weeks.

Each study reported measures of global cognitive function: SCAG (1 study), GBS (1), ADAS-Cog (1), and MMSE (3). Studies [17] and [18] also provided data on functional outcomes. None of the trials reported patient-centred outcomes. The risk of bias was rated as low for studies [17] and [18], and moderate for study [172]. Notably, study [172] reported global cognitive efficiency outcomes only as the proportion of patients reaching a predefined, arbitrary threshold; continuous measures of change were unavailable. Consequently, study [172] contributed to the meta-analysis solely with safety data.

The final meta-analysis included global cognitive function metrics, functional outcomes, and safety outcomes (both overall and severe adverse events). An additional meta-analysis was conducted, restricted to studies (and sub-studies) involving only subcortical vascular dementia.

The results of the meta-analyses for each outcome category are summarised and presented in the *Summary of Findings* table and corresponding forest plots.

Nimodipine showed no significant effect on either global cognitive function metrics (Cohen's d 0.09 95% CI 0.00 – 0.27) or functional outcomes (Cohen's d 0.07 95% CI -0.11 – 0.25). Similarly, no effect was

demonstrated when the analysis was restricted to subjects with subcortical vascular dementia (see *Summary of Findings* table). Rates of adverse events and severe adverse events did not differ significantly between treatment arms, indicating that nimodipine was generally safe and well-tolerated.

2C.10-2 Characteristic of studies included in the meta-analysis

Table Caption: Characteristics of studies assessing *nimodipine* for Vascular Cognitive Impairment.

Setting: hospital and clinics
Intervention: *nimodipine (oral tablets)*

VCI population (label)	Treatment arms	Treatment duration/follow-up	Outcomes	Efficacy	Safety	Quality score*	Study
Subcortical vascular dementia	*Nimodipine 90 mg, 30 mg 3/daily (121)*<br><br>*vs*<br><br>*Placebo (109)*	*52 weeks (NP)*	***<u>Primary outcomes</u>*:** *Yes (SCAG)*<br><br>*<u>Other outcomes</u>:*<br>*Cognitive: yes*<br>*Functional: yes*<br>*Patient-centred: no*	*Partially in favour of treatment*	*Rate of AE and SAE equally represented in both arms*	***Overall***: Good<br><br>***QI***:<br>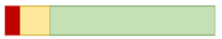	[17]
Post stroke MCI	*Nimodipine 90 mg, 30 mg 3/daily (287)*<br><br>*vs*<br><br>*Placebo (291)*	*6 months (NP)*	***<u>Primary outcomes</u>*:** *Yes (MMSE, ADAS-CoG)*<br><br>*<u>Other outcomes</u>:*<br>*Cognitive: yes*<br>*Functional: no*<br>*Patient-centred: no*	*Partially in favour of treatment*	*Rate of AE and SAE equally represented in both arms*	***Overall***: Fair<br><br>***QI***:<br>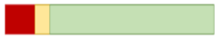	[172]
Vascular Dementia (MID arm)	*Nimodipine 90 mg, 30 mg 3/daily (82)*<br><br>*vs*<br><br>*Placebo (82)*	*26 weeks (NP)*	***<u>Primary outcomes</u>*:** *Yes (GBS)*<br><br>*<u>Other outcomes</u>:*<br>*Cognitive: yes*<br>*Functional: yes*<br>*Patient-centred: no*	*Neutral*	*Safety outcomes not reported for MID subgroup separately*	***Overall***: Good<br><br>***QI:***<br>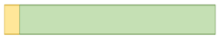	[18] ([16])
Vascular Dementia (subcortical vascular dementia arm)	*Nimodipine 90 mg, 30 mg 3/daily (43)*<br><br>*vs*	*26 weeks (NP)*	*See above*	*Partially in favour of treatment*	*See above*	*See above*	[18] ([16])

	Placebo (44)				

Abbreviations: AE, Adverse events; GBS, Göttfries-Brane-Steen Scale; IADL, Instrumental Activities of Daily Living; MID, multi-infarct dementia; MMSE, Mini-mental state exam; NOSGER, Nurses' Observation Scale for Geriatric Patients; SCAG, Sandoz Clinical Assessment-Geriatric Scale; SAE, Severe Adverse events.

Notes: * Overall quality as rated according to the NIH Quality Assessment tools for controlled intervention studies is reported here. QI (Quality Index) is a graphical, colour-coded representation of the number of items on the scale rated respectively as at high-risk (red), unclear risk (yellow) or low-risk (green) of bias.

Studies excluded from meta-analysis:

VCI population (label)	Treatment arms	Treatment duration/follow-up	Outcomes	Efficacy	Safety	Quality score*	Study
Subcortical Vascular dementia	*Choline alphoscerate 1200 mg + Nimodipine 90 mg (24)*<br><br>***vs***<br><br>*Placebo + Nimodipine 90 mg (24)*	52 weeks *(NP)*	***<u>Primary outcomes</u>***<br>*Cognitive: MoCA*<br>*Functional: no*<br>*Patient-centred outcomes: no*<br><br>*<u>Other outcomes</u>:*<br>*Cognitive: yes*<br>*Functional: yes*<br>*Patient-centred outcomes: yes*	*Against treatment*	*No difference rate of AE between treatment arms.*<br>*No SAE reported.*	***Overall***: Good<br><br>***QI:***<br>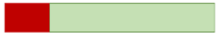	[3]
Vascular dementia	*Arm 1: Electroacupuncture + Nimodipine 60 mg (26)*<br>***vs***<br>*Arm 2: Electroacupuncture (23)*<br>***vs***<br>*Arm 3: Nimodipine 60 mg (24)*	*6 weeks (NP)*<br><br>*[total: 30 acupuncture sessions]*	***<u>Primary outcomes:</u>***<br>*None reported*<br><br>*<u>Other Outcomes</u>*<br>*Cognitive: yes*<br>*Functional: no*<br>*Patient-centred outcomes: no*	*Neutral*	*No adverse reactions reported in any treatment arm*	***Overall:*** *Poor*<br><br>***QI:***<br>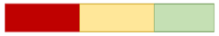	[100]
Post-stroke MCI	*Arm 1: Acupuncture 30 minutes 6 days/week + Nimodipine 90 mg (40)*<br><br>***vs***<br><br>*Arm 2: Acupuncture 30 minutes 6 days/week*<br><br>***vs***<br><br>*Arm 3: Nimodipine 90 mg (40)*	*3 months (follow-up: 3 months)*<br><br>*[total: 72 acupuncture sessions]*	*<u>Primary outcomes</u>*<br>*Yes (MoCA)*<br><br>*<u>Other outcomes</u>:*<br>*Cognitive: no*<br>*Functional: no*<br>*Patient-centred: no*	*In favour of combined treatment (arm 1)*	*Safety outcome not reported*	***Overall:*** *Good*<br><br>***QI:***<br>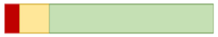	[101]

Abbreviations: AE, adverse events; MCI, Mild Cognitive Impairment; MoCA, Montreal Cognitive Assessment; NP, not present; SAE, severe adverse events.

Notes: * Overall quality as rated according to the NIH Quality Assessment tools for controlled intervention studies is reported here. QI (Quality Index) is a graphical, colour-coded representation of the number of items on the scale rated respectively as at high-risk (red), unclear risk (yellow) or low-risk (green) of bias.

2C.10-3 Summary of findings and figures for meta-analyses

Table Caption: Summary of findings for the main comparisons.

Setting: hospital and clinics
Intervention: *nimodipine (oral tablets)*
Comparator: *placebo*

Outcomes	N° of participants (studies)	VCI population (label)	Efficacy measure	Quality of evidence (GRADE)	Statistical heterogeneity	Studies
Global cognitive efficiency *SCAG, GBS-intellectual*<br><br>***Treatment duration:*** *26-52 weeks*<br><br>***Follow-up after treatment:*** *none*	481 (2 RCTs)	Subcortical Vascular Dementia – MID	**Cohen's d:** 0.09<br>95% CI (-0.00 – 0.27)	⊕⊕◯◯ Low[3,4]	I^2= 0	[17]<br>[18][1]<br>([16])
Global cognitive efficiency *MMSE*	481 (2 RCTs)	Subcortical Vascular Dementia – MID	**Cohen's d:** 0.07<br>95% CI (-0.11 – 0.25)<br><br>**Mean difference:** + 0.41<br>95% CI (-0.48, 1.30)	⊕⊕⊕◯ Moderate[3]	I^2= 0	[17]<br>[18]<br>([16])
Functional outcomes *IADL, NOSGER*	481 (2 RCTs)	Subcortical Vascular Dementia – MID	**Cohen's d:** 0.07<br>95% CI (-0.13 – 0.27)	⊕⊕◯◯ Low[3,4]	I^2= 0	[17]<br>[18]<br>([16])

Patient-centred outcomes *Not reported*	*See note[2]*	*See note[2]*	*See note[3]*			
Safety outcomes *(AE and SAE rate)*	1061 (3 RCTs)	Subcortical Vascular Dementia – MID – Post-stroke MCI	**AE:** 0.84 95% CI (0.63 – 1.13)	**AE:** ⊕⊕○○ Low[3,5]	I^2= 78.39	[17] [18] [172]
			**SAE:** 0.85 *95% CI (0.47 – 1.52)*	**SAE:** ⊕⊕○○ Low[3,5]	I^2= 67.73	

Abbreviations: AE, Adverse events; GBS, Göttfries-Brane-Steen Scale; IADL, Instrumental Activities of Daily Living; MID, multi-infarct dementia; MMSE, Mini-mental state exam; NOSGER, Nurses' Observation Scale for Geriatric Patients; SCAG, Sandoz Clinical Assessment-Geriatric Scale; SAE, Severe Adverse events.

Notes:
[1]Study [16] reports a post-hoc analysis, separated by VCI label, of trial [18].
[2] No studies among the one included in meta-analysis reported patient-centred outcomes.

GRADE Working Group grades of evidence:

High certainty: We are very confident that the true effect lies close to that of the estimate of the effect.

Moderate certainty: We are moderately confident in the effect estimate: the true effect is likely to be close to the estimate of the effect, but there is a possibility that it is substantially different.

Low certainty: Our confidence in the effect estimate is limited: the true effect may be substantially different from the estimate of the effect.

Very low certainty: We have very little confidence in the effect estimate: the true effect is likely to be substantially different from the estimate of effect.

[3]Low generalisability due to inclusion of different VCI populations (downgraded once for indirectness)
[4]Different outcome measures employed (downgraded once).
[5]Some inconsistency in estimates (downgraded once).

Table Caption: Summary of findings for the comparisons only between trials including participants with subcortical vascular dementia

Setting: hospital and clinics
VCI label: subcortical vascular dementia
Intervention: *nimodipine (oral tablets)*
Comparator: *placebo*

Outcomes	N° of participants (studies)	VCI population (label)	Efficacy measure	Quality of evidence (GRADE)	Statistical heterogeneity	Studies
Global cognitive efficiency *SCAG, GBS-intellectual* <br>***Treatment duration:*** *26-52 weeks* <br>***Follow-up after treatment:*** *none*	317 (2 RCTs)	Subcortical Vascular Dementia	**Cohen's d:** 0.07 95% CI (-0.15 – 0.29)	⊕⊕⊕○ Moderate[2]	I^2= 0	[17] [18][1] ([16])
Global cognitive efficiency *MMSE*	317 (2 RCTs)	Subcortical Vascular Dementia	**Cohen's d:** 0.08 95% CI (-0.14 – 0.30) <br>**Mean difference:** + 0.41 95% CI (-0.63, 1.41)	⊕⊕⊕⊕ High	I^2= 0	[17] [18] ([16])
Functional outcomes *NOSGER, IADL*	317 (2 RCTs)	Subcortical Vascular Dementia	**Cohen's d:** 0.09 95% CI (-0.17 – 0.35)	⊕⊕⊕○ Moderate [2]	I^2= 0	[[17] [18] ([16])

Abbreviations: AE, Adverse events; GBS, Göttfries-Brane-Steen Scale; IADL, Instrumental Activities of Daily Living; MID, multi-infarct dementia; MMSE, Mini-mental state exam; NOSGER, Nurses' Observation Scale for Geriatric Patients; SCAG, Sandoz Clinical Assessment-Geriatric Scale; SAE, Severe Adverse events.

Notes:

[1]Study [16] reports a post-hoc analysis, separated by VCI label, of trial [18].

GRADE Working Group grades of evidence:

High certainty: We are very confident that the true effect lies close to that of the estimate of the effect.

Moderate certainty: We are moderately confident in the effect estimate: the true effect is likely to be close to the estimate of the effect, but there is a possibility that it is substantially different.

Low certainty: Our confidence in the effect estimate is limited: the true effect may be substantially different from the estimate of the effect.

Very low certainty: We have very little confidence in the effect estimate: the true effect is likely to be substantially different from the estimate of effect.

[2]Different outcome measures employed (downgraded once).

eFigure 50: *Forest plot representing meta-analysis of nimodipine efficacy on global cognitive efficiency primary outcomes (SCAG, GBS-intellectual subscale). Effect size is reported as Cohen's d; a fixed effects model was used for estimation.*

	ES	95% CI	W	SE	Sig.	N	N1	N2
Pantoni 2000 MID	0.13	-0.18 / 0.43	34.11%	0.16	0.418	164	82	82
Pantoni 2000 SVD	0.09	-0.33 / 0.51	18.11%	0.21	0.666	87	43	44
Pantoni 2005	0.06	-0.20 / 0.31	47.78%	0.13	0.672	230	121	109
Overall (fixed-effect model)	0.09	-0.09 / 0.27	100.00%	0.09	0.343	481	246	235

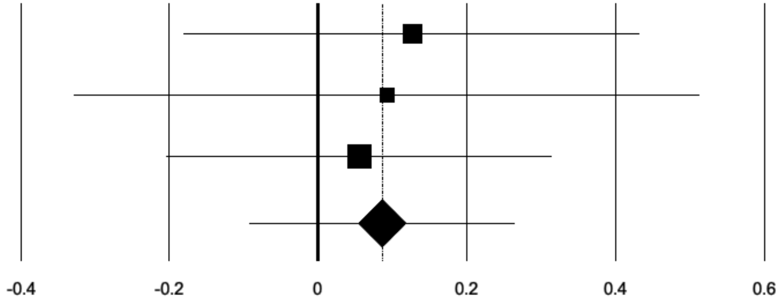

Heterogeneity: Cochran's Q = 0.12, df = 2 (p = 0.942), Tau^2 = 0, I^2 = 0

__eFigure 51:__ Forest plot representing meta-analysis of nimodipine efficacy on Mini-Mental State Examination. Effect size is reported as Cohen's d (__panel a__) and as unstandardised mean difference (__panel b__); fixed effects models were used for estimations.

a

	ES	95% CI	W	SE	Sig.	N	N1	N2
Pantoni 2000 MID	0.07	-0.24 / 0.37	34.15%	0.16	0.662	164	82	82
Pantoni 2000 SVD	0.12	-0.30 / 0.54	18.09%	0.21	0.569	87	43	44
Pantoni 2005	0.06	-0.20 / 0.32	47.76%	0.13	0.653	230	121	109
Overall (fixed-effect model)	0.07	-0.11 / 0.25	100.00%	0.09	0.419	481	246	235

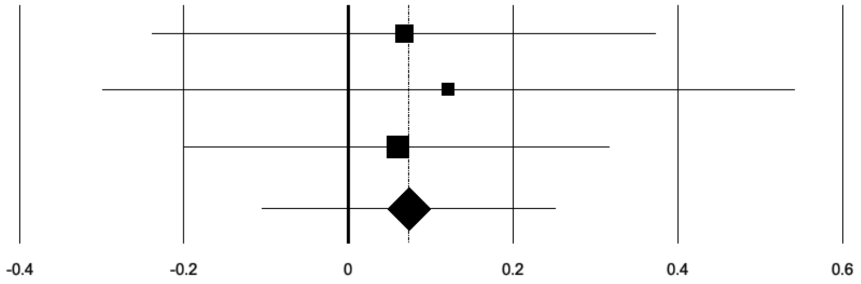

Heterogeneity: Cochran's Q = 0.13, df = 2 (p = 0.937), Tau^2 = 0, I^2 = 0

b

	ES	95% CI	W	SE	Sig.	N	N1	N2
Pantoni 2000 MID	0.47	-1.37 / 2.31	23.53%	0.94	0.617	164	82	82
Pantoni 2000 SVD	0.75	-1.53 / 3.03	15.39%	1.16	0.518	87	43	44
Pantoni 2005	0.30	-0.84 / 1.44	61.08%	0.58	0.607	230	121	109
Overall (fixed-effect model)	0.41	-0.48 / 1.30	100.00%	0.46	0.369	481	246	235

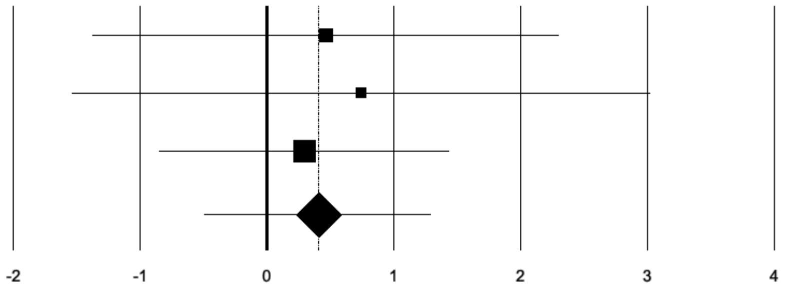

Heterogeneity: Cochran's Q = 0.15, df = 2 (p = 0.926), Tau^2 = 0, I^2 = 0

eFigure 52: *Forest plot representing meta-analysis of nimodipine efficacy on functional outcomes (IADL, NOSGER). Effect size is reported as Cohen's d; a fixed effects model was used for estimation.*

	ES	95% CI	W	SE	Sig.	N	N1	N2
Pantoni 2000 MID	0.04	-0.26 / 0.35	42.10%	0.16	0.788	164	82	82
Pantoni 2000 SVD	0.16	-0.26 / 0.58	22.26%	0.21	0.448	87	43	44
Pantoni 2005	0.05	-0.29 / 0.38	35.63%	0.17	0.779	149	94	55
Overall (fixed-effect model)	0.07	-0.13 / 0.27	100.00%	0.10	0.484	400	219	181

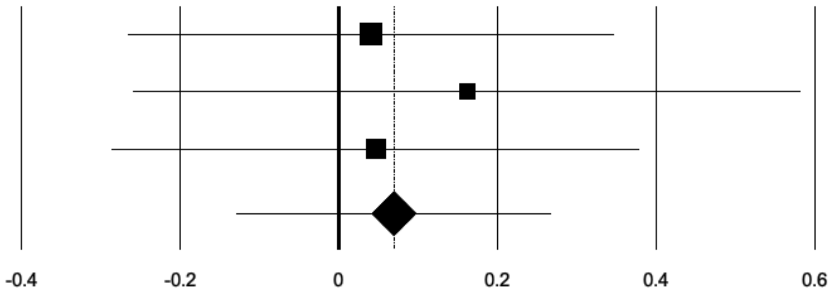

Heterogeneity: Cochran's Q = 0.23, df = 2 (p = 0.893), Tau^2 = 0, I^2 = 0

__eFigure 53:__ Forest plot representing meta-analysis of the rate of adverse events between intervention arm and placebo (overall rate adverse events, panel a, and rate of severe adverse events, panel b). Effect size is reported as rate ratio; random effects models were used for estimation.

a

	ES	95% CI	W	Sig.
Pantoni 2000	0.84	0.57 / 1.24	25.18%	0.389
Pantoni 2005	0.68	0.54 / 0.84	35.31%	0.001
Zheng 2019	1.03	0.88 / 1.20	39.51%	0.739
Overall (random-effects model)	0.84	0.63 / 1.13	100.00%	0.257

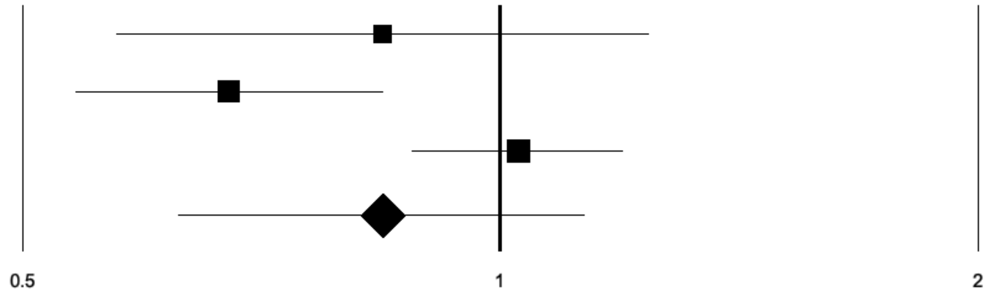

Heterogeneity: Cochran's Q 9.26, df = 2 (p = 0.010), $Tau^2 = 0.05$, $I^2 = 78.39$

b

	ES	95% CI	W	Sig.
Pantoni 2000	0.74	0.39 / 1.42	31.06%	0.369
Pantoni 2005	0.59	0.40 / 0.86	40.94%	0.007
Zheng 2019	1.68	0.81 / 3.49	28.00%	0.166
Overall (random-effects model)	0.85	0.47 / 1.52	100.00%	0.581

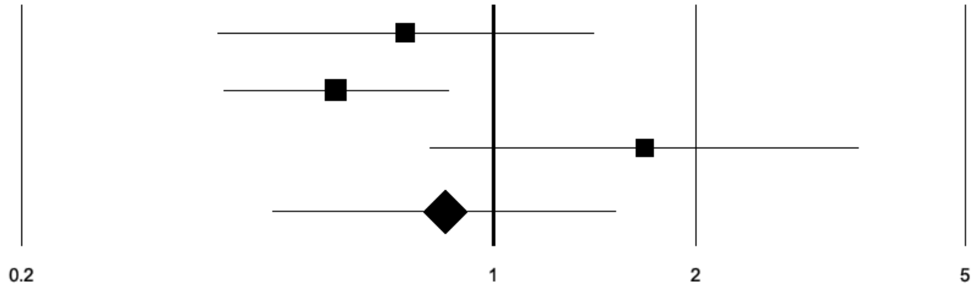

Heterogeneity: Cochran's Q = 6.20, df = 2 (p = 0.045), $Tau^2 = 0.18$, $I^2 = 67.73$

eFigure 54: *Forest plot representing meta-analysis of nimodipine efficacy on global cognitive efficiency primary outcomes (SCAG, GBS-intellectual subscale) in participants with* ***subcortical vascular dementia****. Effect size is reported as Cohen's d; a fixed effects model was used for estimation.*

	ES	95% CI	W	SE	Sig.	N	N1	N2
Pantoni 2000 SVD	0.09	-0.33 / 0.51	27.48%	0.21	0.666	87	43	44
Pantoni 2005	0.06	-0.20 / 0.31	72.52%	0.13	0.672	230	121	109
Overall (fixed-effect model)	0.07	-0.15 / 0.29	100.00%	0.11	0.557	317	164	153

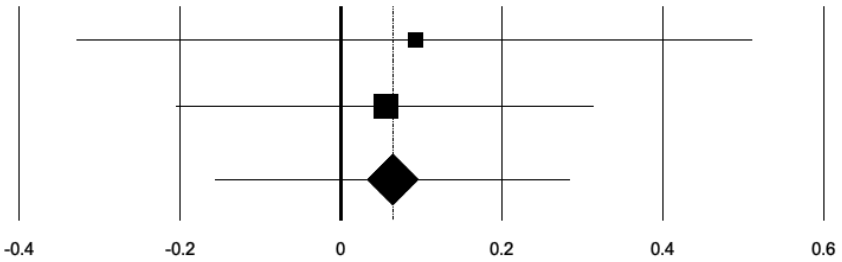

Heterogeneity: Cochran's Q = 0.02, df = 2 (p = 0.884), Tau^2 = 0, I^2 = 0

__eFigure 55:__ Forest plot representing meta-analysis of nimodipine efficacy on Mini-Mental State Examination in participants with __subcortical vascular dementia__. Effect size is reported as Cohen's d (__panel a__) and as unstandardised mean difference (__panel b__); fixed effects models were used for estimations.

a

	ES	95% CI	W	SE	Sig.	N	N1	N2
Pantoni 2000 SVD	0.12	-0.30 / 0.54	27.47%	0.21	0.569	87	43	44
Pantoni 2005	0.06	-0.20 / 0.32	72.53%	0.13	0.653	230	121	109
Overall (fixed-effect model)	0.08	-0.14 / 0.30	100.00%	0.11	0.496	317	164	153

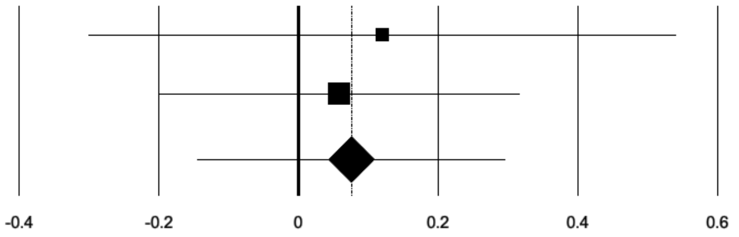

Heterogeneity: Cochran's Q = 0.12, df = 2 (p = 0.724), Tau^2 = 0, I^2 = 0

b

	ES	95% CI	W	SE	Sig.	N	N1	N2
Pantoni 2000 SVD	0.75	-1.53 / 3.03	20.13%	1.16	0.518	87	43	44
Pantoni 2005	0.30	-0.84 / 1.44	79.87%	0.58	0.607	230	121	109
Overall (fixed-effect model)	0.39	-0.63 / 1.41	100.00%	0.52	0.454	317	164	153

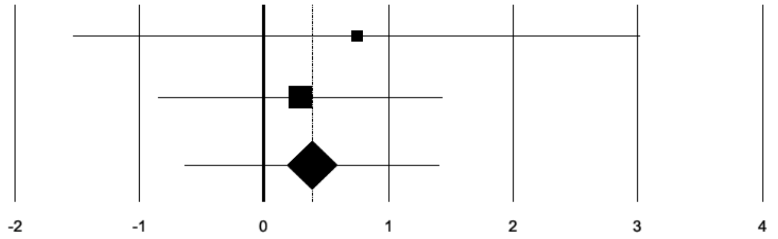

Heterogeneity: Cochran's Q = 0.15, df = 2 (p = 0.697), Tau^2 = 0, I^2 = 0

eFigure 56: *Forest plot representing meta-analysis of nimodipine efficacy on functional outcomes (IADL, NOSGER). Effect size is reported as Cohen's d; a fixed effects model was used for estimation.*

	ES	95% CI	W	SE	Sig.	N	N1	N2
Pantoni 2000 SVD	0.16	-0.26 / 0.58	38.46%	0.21	0.448	87	43	44
Pantoni 2005	0.05	-0.29 / 0.38	61.54%	0.17	0.779	149	94	55
Overall (fixed-effect model)	0.09	-0.17 / 0.35	100.00%	0.13	0.490	236	137	99

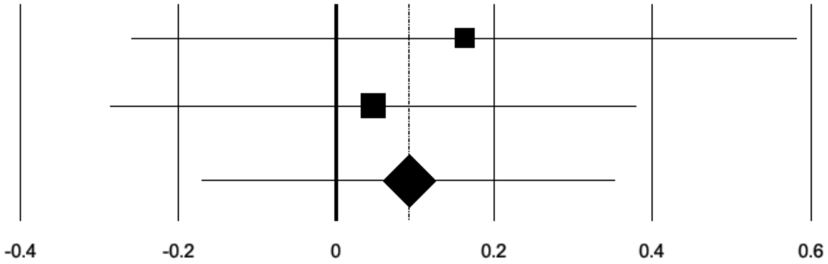

Heterogeneity: Cochran's Q = 0.17, df = 2 (p = 0.682), Tau^2 = 0, I^2 = 0

2C.10-4 Sensitivity Analyses

We performed sensitivity analyses, varying the $Corr_{pre\text{-}post}$ for both global cognitive function outcome meta-analyses and functional outcomes. As $Corr_{pre\text{-}post}$ was neither reported nor inferable from previous studies for any outcome category. The effect size magnitude remained largely comparable across all $Corr_{pre\text{-}post}$ variations performed. Forest plots for these analyses, along with their respective heterogeneity statistics, are presented in *eFigures 57-59*. Characteristics of the studies included in the sensitivity analyses are detailed in the *Characteristics of Studies* table.

eFigure 57: *Sensitivity analysis showing variation of nimodipine efficacy on global cognitive efficiency primary outcomes (SCAG, GBS intellectual) when pre-post correlation coefficient is varied between 0* ***(panel a)*** *and 0.8* ***(panel b)****. Effect size is reported as Cohen's d; a fixed effects model was used for estimation.*

a

	ES	95% CI	W	SE	Sig.	N	N1	N2
Pantoni 2000 MID	0.02	-0.29 / 0.33	34.16%	0.16	0.897	164	82	82
Pantoni 2000 SVD	0.11	-0.31 / 0.53	18.09%	0.21	0.600	87	43	44
Pantoni 2005	0.06	-0.20 / 0.31	47.75%	0.13	0.672	230	121	109
Overall (fixed-effect model)	0.05	-0.12 / 0.23	100.00%	0.09	0.554	481	246	235

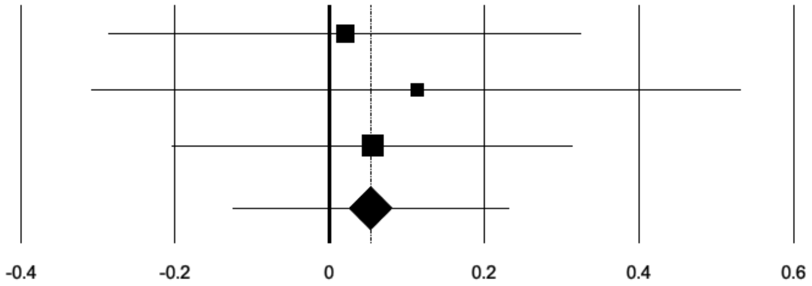

Heterogeneity: Cochran's Q = 0.12, df = 2 (p = 0.941), Tau^2 = 0, I^2 = 0

b

	ES	95% CI	W	SE	Sig.	N	N1	N2
Pantoni 2000 MID	0.02	-0.29 / 0.32	34.15%	0.16	0.923	164	82	82
Pantoni 2000 SVD	0.09	-0.33 / 0.51	18.10%	0.21	0.666	87	43	44
Pantoni 2005	0.06	-0.20 / 0.31	47.75%	0.13	0.672	230	121	109
Overall (fixed-effect model)	0.05	-0.13 / 0.23	100.00%	0.09	0.594	481	246	235

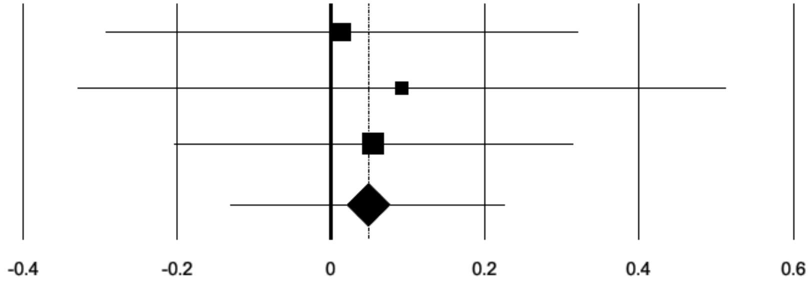

Heterogeneity: Cochran's Q = 0.08, df = 2 (p = 0.962), Tau^2 = 0, I^2 = 0

*eFigure 58: Sensitivity analysis showing variation of nimodipine efficacy on Mini-Mental State when pre-post correlation coefficient is varied between 0 **(panel a)** and 0.8 **(panel b)**. Effect size is reported as Cohen's d; a fixed effects model was used for estimation.*

a

	ES	95% CI	W	SE	Sig.	N	N1	N2
Pantoni 2000 MID	0.47	-1.08 / 2.02	37.57%	0.79	0.553	164	82	82
Pantoni 2000 SVD	0.75	-1.20 / 2.70	23.80%	1.00	0.452	87	43	44
Pantoni 2005	0.30	-1.23 / 1.83	38.63%	0.78	0.701	230	121	109
Overall (fixed-effect model)	0.47	-0.48 / 1.42	100.00%	0.49	0.333	481	246	235

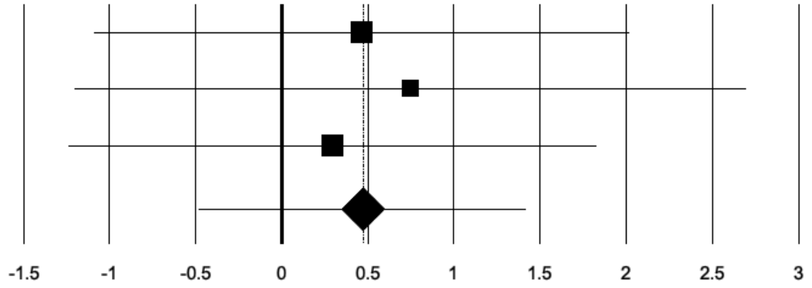

Heterogeneity: Cochran's Q = 0.13, df = 2 (p = 0.939), $Tau^2 = 0$, $I^2 = 0$

b

	ES	95% CI	W	SE	Sig.	N	N1	N2
Pantoni 2000 MID	0.47	-1.53 / 2.47	13.22%	1.02	0.646	164	82	82
Pantoni 2000 SVD	0.75	-1.71 / 3.21	8.75%	1.26	0.551	87	43	44
Pantoni 2005	0.30	-0.52 / 1.12	78.04%	0.42	0.476	230	121	109
Overall (fixed-effect model)	0.36	-0.37 / 1.09	100.00%	0.37	0.330	481	246	235

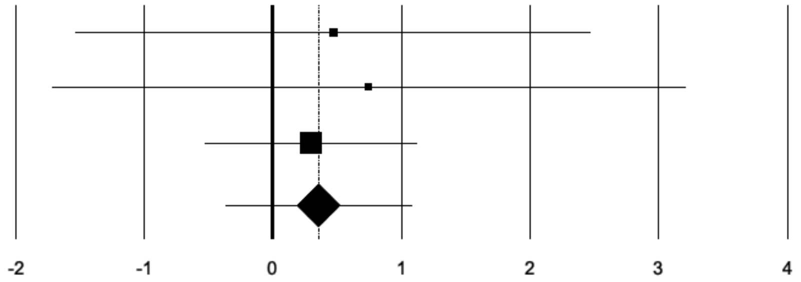

Heterogeneity: Cochran's Q = 0.13, df = 2 (p = 0.938), $Tau^2 = 0$, $I^2 = 0$

***eFigure 59**: Sensitivity analysis showing variation of nimodipine effect size on functional outcomes (NOSGER, IADL) when pre-post correlation coefficient is varied between 0 **(panel a)** and 0.8 **(panel b)**. Effect size is reported as Cohen's d; a fixed effects model was used for estimation.*

a

	ES	95% CI	W	SE	Sig.	N	N1	N2
Pantoni 2000 MID	0.05	-0.26 / 0.36	42.11%	0.16	0.746	164	82	82
Pantoni 2000 SVD	0.19	-0.23 / 0.62	22.24%	0.21	0.367	87	43	44
Pantoni 2005	0.04	-0.30 / 0.37	35.65%	0.17	0.832	149	94	55
Overall (fixed-effect model)	0.08	-0.12 / 0.28	100.00%	0.10	0.446	400	219	181

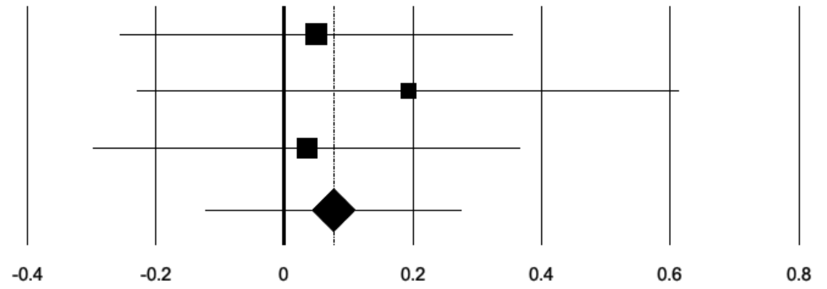

Heterogeneity: Cochran's Q = 0.38, df = 2 (p = 0.826), Tau^2 = 0, I^2 = 0

b

	ES	95% CI	W	SE	Sig.	N	N1	N2
Pantoni 2000 MID	0.04	-0.26 / 0.35	42.11%	0.16	0.788	164	82	82
Pantoni 2000 SVD	0.15	-0.27 / 0.57	22.28%	0.21	0.484	87	43	44
Pantoni 2005	0.08	-0.25 / 0.41	35.62%	0.17	0.643	149	94	55
Overall (fixed-effect model)	0.08	-0.12 / 0.28	100.00%	0.10	0.434	400	219	181

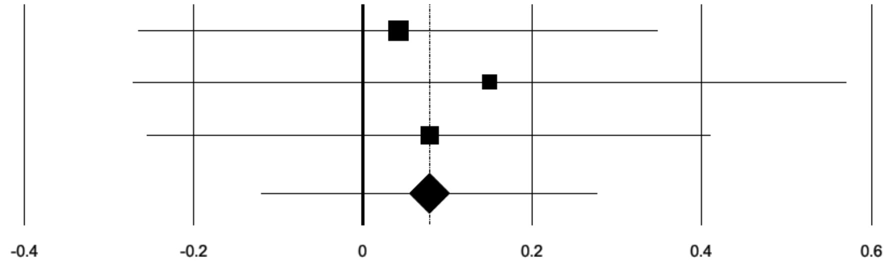

Heterogeneity: Cochran's Q = 0.17, df = 2 (p = 0.920), Tau^2 = 0, I^2 = 0

2C.11 Remote ischemic conditioning

2C.11-1 Description of studies and meta-analysis main results

We retrieved three randomised controlled trials (RCTs) evaluating remote ischaemic conditioning (RIC) monotherapy for VCI against sham treatment. All studies involved bilateral limb compression in four to five daily cycles of five minutes. Treatment duration varied significantly across studies, ranging from seven days to one year. The study with the shortest duration, [126], was included only in sensitivity analyses.

Of the two studies with similar durations ([112] and [113]), we were only able to perform a meta-analysis on the surrogate outcome of white matter volume change. Global cognitive function data (MoCA) were available only from study [112], while functional data (ADL) were available only from study [113]. Safety outcomes were reported solely by study [113], which reported no adverse events or incidents in either treatment arm. Data for outcomes from single studies were converted to effect size measures and reported with a note in the *Summary of Findings* table alongside the meta-analysed data.

Meta-analysis of RIC's effect on longitudinal change in white matter hyperintensity volume did not show a significant effect (negative direction favours treatment, Cohen’s d: -0.29, 95% CI [-0.78 to 0.19], p = 0.23, MD: -2.05 cm^3, 95% CI [-6.81 to 2.71], p = 0.39). No significant effects on global cognitive function or functional outcomes were reported by the single studies providing data on these outcome categories.

The results of the meta-analysis of instrumental outcomes and available data for other outcomes are presented in the *Summary of Findings table* and *eFigure 60*.

2C.11-2 Characteristic of studies considered for meta-analysis

Table Caption: Characteristics of studies assessing *remote ischemic conditioning* for Vascular Cognitive Impairment.

Setting: hospital and clinics
Intervention: *remote ischemic conditioning*

Studies included in meta-analysis:

VCI population (label)	Treatment arms	Treatment duration/follow-up	Outcomes	Efficacy	Safety	Quality score*	Study
Subcortical ischemic vascular MCI	*Bilateral upper limbs RIC twice daily (14)*<br><br>*vs*<br><br>*sham treatment (16)*	*12 months (NP)*	***Primary outcomes**:*<br>*Yes (WMHv)*<br><br>*Other outcomes:*<br>*Cognitive: yes*<br>*Functional: no*<br>*Patient-centred: no*<br>*Instrumental (CBC, cholesterol and lipids, TC-US parameters*	*Partially in favour of treatment*	*Safety outcomes not reported*	***Overall**: Good*<br><br>***QI**:*<br>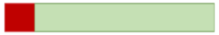	[112]
Subcortical ischemic vascular dementia	*Bilateral upper limbs RIC twice daily (18)*<br><br>*vs*<br><br>*sham treatment (21)*	*6 months (NP)*	***Primary outcomes**:*<br>*Yes (NPS tests)*<br><br>*Other outcomes:*<br>*Cognitive: no*<br>*Functional: no*<br>*Patient-centred: no*<br>*Instrumental: WMHv, DTI metrics, lab values (haematocrit, inflammatory serum markers)*	*Neutral*	*No adverse events were reported in both treatment arms*	***Overall**: Good*<br><br>***QI:***<br>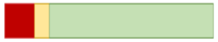	[113]

Studies included only in sensitivity analyses or excluded:

VCI population (label)	Treatment arms	Treatment duration/follow-up	Outcomes	Efficacy	Safety	Quality score*	Study
Post-stroke MCI (Acute/subacute stroke)	*Bilateral upper limbs RIC twice daily (24)*<br><br>*vs*<br><br>*sham treatment (24)*	*7 days (6 months)*	***Primary outcomes**:* *None reported*<br><br>*Other outcomes:*<br>*Cognitive: yes*<br>*Functional: no*<br>*Patient-centred: no*	*Neutral*	*Safety outcomes not reported*	**Overall**: Fair<br><br>**QI**: 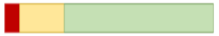	[126]

Abbreviations: AE, adverse events; CBC, complete blood count; DTI, diffusion tensor imaging; NP, not performed; RIC, Remote Ischemic conditioning; SAE, severe adverse events; WMHv, white matter hyperintensity volume.

Notes: *Overall quality, as rated according to the NIH Quality Assessment tools for controlled intervention studies, is reported here. QI (Quality Index) is a graphical, colour-coded representation of the number of items on the scale rated as high-risk (red), unclear risk (yellow) or low-risk (green) of bias.

2C.11-3 Summary of findings and figures for meta-analyses

Table Caption: Summary of findings for the main comparisons.

Setting: hospital and clinics
Intervention: *remote ischemic conditioning*
Comparator: *sham treatment*

Outcomes	N° of participants (n of studies)	VCI population label	Efficacy measure	Quality of evidence (GRADE)	Statistical heterogeneity	Studies
Global cognitive efficiency *MoCA*<br><br>***Treatment duration:*** *12 months*<br><br>***Follow-up after treatment:*** *none*	30 (1 RCT)[1]	Subcortical Ischemic Vascular MCI	**Cohen's d[1]:** 0.54 95% CI (-0.17 – 1.24)	⊕○○○ Very low[2,3,4]		[112]
Functional outcomes *ADL*<br><br>***Treatment duration:*** *6 months*<br><br>***Follow-up after treatment:*** *none*	39 (1 RCT)[2]	Subcortical Ischemic Vascular Dementia	**Cohen's d[2]:** 0.22 95%CI (0.45-0.90)	⊕○○○ Very low[2,3,5]		[113]
Patient-centred outcomes *Not reported*	*See note[3]*	*See note[3]*	*See note[3]*			

Instrumental outcomes White matter hyperintensity volume<br><br>***Treatment duration:*** *6-12 months*<br><br>***Follow-up after treatment:*** *none*	69 (2 RCTs)	Subcortical Ischemic VCI	**Cohen's d:**<br>-0.29<br>95%CI (-0.78-0.19)<br><br>**MD:**<br>-2.05 cm^3<br>95%CI (-6.81-2.71)	⊕○○○ Very low[2,3,4]	$I^2 = 0$	[112]<br>[113]
Safety outcomes AE and SAE	*See note*[5]	*See note*[5]	*See note*[5]			

Abbreviations: ADL, Activities of daily living; AE, adverse events; MD, mean difference; MoCA, Montreal Cognitive assessment; SAE, severe adverse events.

Notes

[1]Only study [112] included data on global cognitive efficiency outcomes: these data have been reported here alongside other meta-analysed data as Cohen's *d*.

GRADE Working Group grades of evidence:

High certainty: We are very confident that the true effect lies close to that of the estimate of the effect.

Moderate certainty: We are moderately confident in the effect estimate: the true effect is likely to be close to the estimate of the effect, but there is a possibility that it is substantially different.

Low certainty: Our confidence in the effect estimate is limited: the true effect may be substantially different from the estimate of the effect.

Very low certainty: We have very little confidence in the effect estimate: the true effect is likely to be substantially different from the estimate of effect.

[2]Only one trial reporting on the outcome (downgraded once).
[3]Two or more items at uncertain (or greater) risk of bias (downgraded once).
[4]Some imprecision (wide 95% confidence interval, downgraded once).
[5]Downgraded once due to imprecision: the 95% CI includes a result that would not be considered clinically important and a result that would likely be considered important.

eFigure 60: *Forest plot representing meta-analysis of remote ischemic conditioning efficacy data on white matter hyperintensity volume change. Effect size is reported as Cohen's d in panel **a** and as unstandardised mean difference (cm³) in panel **b**. A fixed effect model was used for estimation.*

a

	ES	95% CI	W	SE	Sig.	N	N1	N2
Liao 2019	-0.31	-0.96 / 0.34	55.24%	0.33	0.346	37	18	19
Wang 2017	-0.27	-0.99 / 0.45	44.76%	0.37	0.460	30	14	16
Overall (fixed-effect model)	-0.29	-0.78 / 0.19	100.00%	0.25	0.232	67	32	35

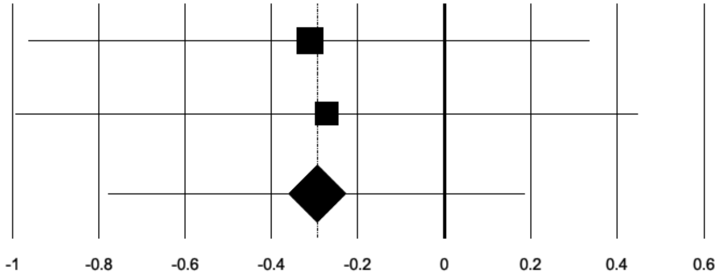

Heterogeneity: Cochran's Q = 0.01, df = 1 (p = 0.935), Tau² = 0, I² = 0

b

	ES	95% CI	W	SE	Sig.	N	N1	N2
Liao 2019	-11.22	-36.80 / 14.36	3.47%	13.05	0.390	37	18	19
Wang 2017	-1.72	-6.57 / 3.13	96.53%	2.47	0.487	30	14	16
Overall (fixed-effect model)	-2.05	-6.81 / 2.71	100.00%	2.43	0.399	67	32	35

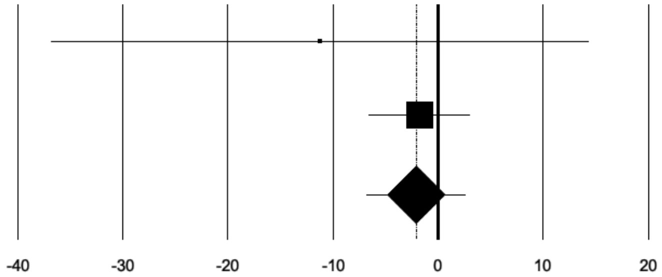

Heterogeneity: Cochran's Q = 0.151, df = 2 (p = 0.474), Tau² = 0, I² = 0

2C.11-4 Sensitivity Analyses

We performed a sensitivity analysis including the study with the shortest treatment duration, [126]. This study administered the intervention for seven days; outcomes were evaluated 180 days post-treatment.

Meta-analysis of global cognitive function outcomes (MoCA) showed a moderate effect size in favour of treatment (Cohen's d 0.75, 95% CI [0.29–1.20], p = 0.001; unstandardised mean difference +1.75 points, 95% CI [1.29–2.20]). The forest plot for this analysis, its associated heterogeneity statistics, and a table summarising the unstandardised mean difference results for the same outcome are presented in *eFigure 61* below. Characteristics of studies included in sensitivity analyses are detailed in the *Characteristics of Studies* table.

***eFigure 61**: Sensitivity analysis showing remote ischemic conditioning effect on global cognitive efficiency primary outcomes (MoCA) including a study with significant variation of intervention duration in the analysis. Effect size is reported as Cohen's d (**panel a**) and as unstandardised mean difference (**panel b**); a fixed effect model was used for estimations.*

a

	ES	95% CI	W	SE	Sig.	N	N1	N2
Li 2020	0.90	0.31 / 1.49	58.31%	0.30	0.003	48	24	24
Wang 2017	0.54	-0.17 / 1.24	41.69%	0.36	0.135	30	14	16
Overall (fixed-effect model)	0.75	0.29 / 1.20	100.00%	0.23	0.001	78	38	40

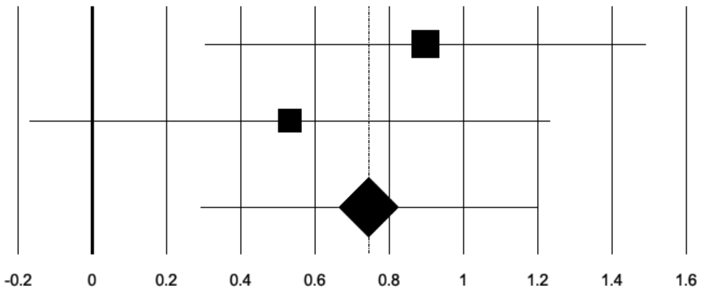

Heterogeneity: Cochran's Q = 0.60, df = 1 (p = 0.438), Tau^2 = 0, I^2 = 0

b

Study	***Unstandardised MD***	***95% Confidence Interval***	***Study Weight***
Li 2020	*1.91*	*1.59 – 2.23*	*15.38%*
Wang 2017	*0.863*	*0.10 – 1.63*	*84.62%*
Overall (fixed-effect model)	*1.748*	*1.29 – 2.20*	*100%*

2C.12 Cognitive Training

2C.12-1 Description of studies and meta-analysis main results

We retrieved eight studies evaluating cognitive training in the treatment of VCI. Regarding outcome categories, four studies reported global cognitive function measures (MoCA and MMSE), and one study reported functional measures (ADL, IADL, DAD) and patient-centred scales (SF-36, EuroQol, Attention Questionnaire). Despite the study [156] included global cognitive efficiency, functional and patient-centred scales, no meta-analysable data were available.

Only the remaining four studies including global cognitive function outcomes were considered for meta-analysis.

Treatment duration was similar for two studies (seven to eight weeks, approximately 18 hours of individual cognitive training), while one study evaluated the training effect over 20 weeks (approximately 40 hours of individual cognitive training) and another study over 4 weeks (approximately 20 hours of training).

The study quality was rated as good for three studies and fair for the remaining one. Pre-post correlation coefficients were set at 0.5 for three studies lacking sufficient data for direct estimation [135, 140, 173], while the coefficient was set at 0.55 in one study using MoCA [135].

The final meta-analysis showed a medium-to-large effect of cognitive training on global cognitive function metrics (Cohen's d 0.71, 95% CI 0.10–1.32, $p = 0.022$). The results of the meta-analysis are presented in the *Summary of Findings* table and its corresponding forest plot.

2C.12-2 Characteristic of studies

Table Caption: Characteristics of studies assessing *Cognitive Training* for Vascular Cognitive Impairment.

Setting: hospital and clinics
Intervention: *Cognitive Training*

Studies included in meta-analysis:

VCI population (label)	Treatment arms	Treatment duration (follow-up)	Outcomes	Efficacy	Quality score	Study
Subcortical vascular MCI	*(intervention group n=21, control group n=22)*	*20 weeks [total: »40 hours]*<br><br>*(follow-up: 6 months)*	***<u>Primary outcomes</u>***<br>***Functional: ADL, IADL, DAD***<br>***Patient-centred: SF-36, EuroQol, Attention Questionnaire***<br><br>*<u>Secondary outcomes</u>*<br>*Cognitive: YES*<br>*Functional: NO*<br>*Patient-centred: NO*<br>*Instrumental: YES (MRI)*	*Neutral*	***Overall**: Good*<br><br>***QI**:*<br>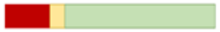	[135]
Subcortical vascular cognitive impairment no dementia	*(intervention group n=30, active control group n=30)*	*7 weeks [total: »17.5 hours]*<br><br>*(follow-up: 6 months)*	***<u>Primary outcomes</u>***<br>***Cognitive: MoCA, TMT B-A***<br><br>*<u>Secondary outcomes</u>*<br>*Cognitive: YES*<br>*Functional: YES*<br>*Patient-centred: NO*<br>*Instrumental: YES (MRI)*	*Partially in favour of treatment*	***Overall**: Good*<br><br>***QI**:*<br>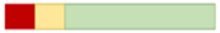	[134]
Post-stroke cognitive impairment	*(experimental group n=20, control group n=15)*	*8 weeks [total: »18 hours] acu*	***<u>Primary outcomes</u>***<br>***none***	*Partially in favour of treatment*	***Overall**: Good*	[139]

VCI population (label)	Treatment arms	Treatment duration (follow-up)	Outcomes	Efficacy	Quality score	Study
		(follow-up: NP)	*<u>Other outcomes</u>*<br>*Cognitive: YES*<br>*Functional: NO*<br>*Patient-centred: NO*		***QI***:<br>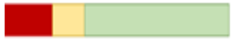	
Post-stroke cognitive impairment	*(experimental group n=25, active control group n=25)*	*4 weeks*<br>*[total: ~20 hours]*<br><br>*(follow-up: NP)*	***<u>Primary outcomes</u>***<br>***none***<br><br>*<u>Other outcomes</u>*<br>*Cognitive: YES*<br>*Functional: NO*<br>*Patient-centred: NO*	*Partially in favour of treatment*	***Overall***: *Fair*<br><br>***QI***:<br>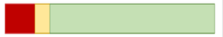	[168]

Studies excluded:

VCI population (label)	Treatment arms	Treatment duration (follow-up)	Outcomes	Efficacy	Quality score	Study
Vascular cognitive impairment no dementia	*(intervention group n=36, control group n=37)*	*12 weeks*<br>*[total: »60 hours]*<br><br>*(follow-up: NP)*	***<u>Primary outcomes</u>***<br>***none***<br><br>*<u>Other outcomes</u>*<br>*Cognitive: YES*<br>*Functional: NO*<br>*Patient-centred: NO*	*In favour of treatment*	***Overall***: *Fair*<br><br>***QI***:<br>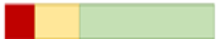	[138]
Post-stroke cognitive impairment	*(physical exercise group n=56, cognitive training group n=57, combined physical exercise and cognitive training group n=55, control group n=57)*	*12 weeks*<br>*[total: »36 hours]*<br><br>*(follow-up: 6 months)*	***<u>Primary outcomes</u>***<br>***none***<br><br>*<u>Other outcomes</u>*<br>*Cognitive: YES*<br>*Functional: NO*<br>*Patient-centred: NO*	*In favour of treatment*	***Overall***: *Good*<br><br>***QI***:<br>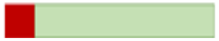	[142]

Post-stroke cognitive impairment	*(conventional cognitive rehabilitation n=20, enriched cognitive rehabilitation n=20)*	*8 weeks*<br>[*total: ~96 hours*]<br><br>*(follow-up: NP)*	***<u>Primary outcomes</u>***<br>***none***<br><br>*<u>Other outcomes</u>*<br>*Cognitive: YES*<br>*Functional: NO*<br>*Patient-centred: NO*	*In favour of treatment*	***Overall**: Fair*<br><br>***QI**:*<br>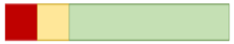	[163]
Post-stroke cognitive impairment	*(Hospital rehabilitation setting: intervention group n=57, control group n=60; community rehabilitation setting: intervention group n=60, control group n=61; home rehabilitation setting: intervention group n=57, control group n=55; nursing home rehabilitation setting: intervention group n=52, control group n=50*	*12 weeks*<br>[*total: ~30 hours*]<br><br>*(follow-up: 4 months)*	***<u>Primary outcomes</u>***<br>***Cognitive: MoCA***<br><br>*<u>Secondary outcomes</u>*<br>*Cognitive: YES*<br>*Functional: YES*<br>*Patient-centred: YES*	*Partially in favour of treatment*	***Overall**: Good*<br><br>***QI**:*<br>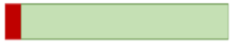	[156]

Abbreviations: ADL, Activities of Daily Living; DAD, Disability Assessment For Dementia; IADL: Instrumental Activities of Daily Living; MoCA, Montreal Cognitive Assessment; MRI, Magnetic Resonance Imaging; SF-36, Short Form Health Survey 36; TMT, Trail Making Test.

Notes: *Overall quality as rated according to the NIH Quality Assessment tools for controlled intervention studies is reported here. QI (Quality Index) is a graphical, colour-coded representation of the number of items on the scale rated respectively as at high-risk (red), unclear risk (yellow) or low-risk (green) of bias.

2C.12-3 Summary of findings and figures for meta-analysis

Table Caption: Summary of findings for the main comparisons. *Cognitive Training* for Vascular Cognitive Impairment.

Cognitive Training for Vascular Cognitive impairment

Setting: hospital and clinics
Intervention: *Cognitive Training*
Comparator: *standard care or active control group*

Outcomes	N° of participants (studies)	VCI population (label)	Effect size	Quality of evidence (GRADE)	Statistical heterogeneity	Studies
Global cognitive efficiency *(MoCA and MMSE)*<br><br>***Treatment duration:*** *4-20 weeks*<br><br>***Follow-up after treatment:*** *6 months (1)* [135]	188 (4 RCTs)	Subcortical vascular MCI (1) [135]<br>Subcortical vascular cognitive impairment no dementia (1) [134]<br>Post-stroke cognitive impairment (2) [139][168]	**Cohen's d:**<br>0.71 - 95% CI (0.10 – 1.32)	⊕○○○Very low[2,3,4,5,6]	I^2= 75.34	[134]<br>[135]<br>[139]<br>[168]
Functional outcomes (ADL, IADL, DAD)	43 (1 RCT)[1]	Subcortical vascular MCI	**ADL - Cohen's d[1]:**<br>0.35 - 95% CI (-0.25 – 0.95)<br><br>**IADL - Cohen's d[1]:**<br>0.16 - 95% CI (-0.44 – 0.76)<br><br>**DAD - Cohen's d[1]:**<br>0.09 - 95% CI (-0.51 – 0.69)	⊕○○○ Very low[4,7,8]	*See note[1]*	[135]
Patient-centred outcomes	43 (1 RCT)[1]	Subcortical vascular MCI	**SF-36 Mental Component - Cohen's d[1]:**<br>0.18 - 95% CI (-0.43 – 0.80)	⊕○○○ Very low[4,7,8]	*See note[1]*	[135]

(SF-36, EuroQol, Attention Questionnaire)			**SF-36 Physical Component - Cohen's d[1]:**<br>0.12 - 95% CI (-0.50 – 0.73)<br><br>**EuroQol (visual scale) - Cohen's d[1]:**<br>0.43 - 95% CI (-0.18 – 1.03)<br><br>**Attention Questionnaire- Cohen's d[1]:**<br>0.35 - 95% CI (-0.25 – 0.96)	

Abbreviations: ADL, Activities of Daily Living; DAD, Disability Assessment For Dementia; IADL: Instrumental Activities of Daily Living; MMSE, Mini Mental Stae Examination; MoCA, Montreal Cognitive Assessment; SF-36, Short Form Health Survey 36.

Notes:

[1]Only study [135] included data on functional and patient-centred outcomes: these data have been reported here alongside other meta-analysed data as Cohen's *d*.

GRADE Working Group grades of evidence:

High certainty: We are very confident that the true effect lies close to that of the estimate of the effect.

Moderate certainty: We are moderately confident in the effect estimate: the true effect is likely to be close to the estimate of the effect, but there is a possibility that it is substantially different.

Low certainty: Our confidence in the effect estimate is limited: the true effect may be substantially different from the estimate of the effect.

Very low certainty: We have very little confidence in the effect estimate: the true effect is likely to be substantially different from the estimate of effect.

[2]Low generalisability due to inclusion of different VCI populations (downgraded once for indirectness)
[3]Some inconsistency in point estimates (downgraded once).
[4]Two or more items at uncertain (or greater) risk of bias (downgraded once).
[5]Some imprecision (wide 95% confidence interval, downgraded once).
[6]Different outcome measures employed (downgraded once).
[7]Only one trial reporting on the outcome (downgraded once).
[8]Imprecision (wide 95% confidence interval, downgraded twice)

***eFigure 62**: Forest plot representing meta-analysis of Cognitive Training effect in VCI on global cognitive efficiency outcomes (MoCA and MMSE): random effect model, Cohen's d.*

	ES	95% CI	W	SE	Sig.	N	N1	N2
De Luca 2018	0.77	0.08 / 1.46	23.29%	0.35	0.029	35	20	15
Pantoni 2017	0.00	-0.60 / 0.60	25.24%	0.31	0.993	43	21	22
Soni 2025	1.53	0.90 / 2.16	24.57%	0.32	0.000	50	25	25
Tang 2019	0.58	0.07 / 1.10	26.89%	0.26	0.027	60	30	30
Overall (random-effects model)	0.71	0.10 / 1.32	100.00%	0.31	0.022	188	96	92

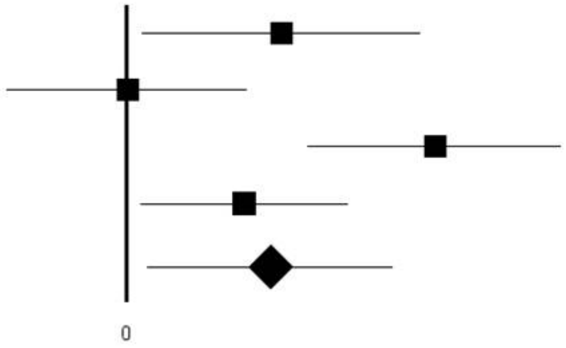

Heterogeneity: Cochran's Q = 12.17, df = 3 (p = 0.007), Tau^2 = 0.29, I^2 = 75.34

2C.12-4 Sensitivity Analyses

Sensitivity analyses were performed by varying pre-post correlation coefficients in three studies lacking sufficient data for direct estimation [135, 140, 173]. The effect size remained largely comparable across the variations. Forest plots for these analyses, along with their respective heterogeneity statistics, are presented in *eFigure 63*. Characteristics of the studies included in the sensitivity analyses are detailed in the *Characteristics of Studies* table.

*eFigure 63: Forest plot representing sensitivity-analyses of Cognitive Training effect in VCI on global cognitive efficiency outcomes (MoCA and MMSE), variating pre-post correlation coefficients between 0.8 (panel **a**) and 0 (panel **b**). Random effect model, Cohen's d.*

a

	ES	95% CI	W	SE	Sig.	N	N1	N2
De Luca 2018	1.21	0.48 / 1.93	23.06%	0.37	0.001	35	20	15
Pantoni 2017	0.00	-0.59 / 0.60	25.37%	0.31	0.990	43	21	22
Soni 2025	1.53	0.90 / 2.16	24.78%	0.32	0.000	50	25	25
Tang 2019	0.58	0.07 / 1.10	26.79%	0.26	0.027	60	30	30
Overall (random-effects model)	0.82	0.16 / 1.47	100.00%	0.34	0.015	188	96	92

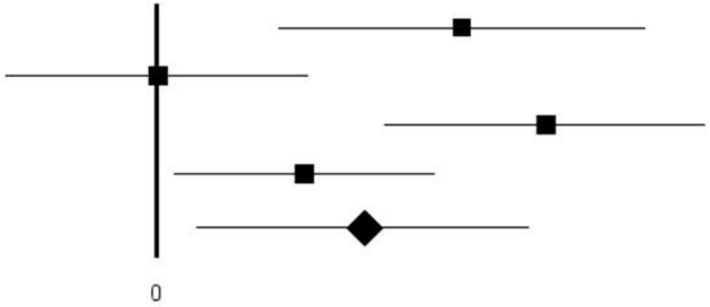

Heterogeneity: Cochran's Q = 13.82, df = 3 (p = 0.003), $Tau^2 = 0.35$, $I^2 = 78.29$

b

	ES	95% CI	W	SE	Sig.	N	N1	N2
De Luca 2018	0.55	-0.14 / 1.23	23.47%	0.35	0.116	35	20	15
Pantoni 2017	0.00	-0.60 / 0.60	25.18%	0.31	0.995	43	21	22
Soni 2025	1.53	0.90 / 2.16	24.51%	0.32	0.000	50	25	25
Tang 2019	0.58	0.07 / 1.10	26.83%	0.26	0.027	60	30	30
Overall (random-effects model)	0.66	0.05 / 1.27	100.00%	0.31	0.033	188	96	92

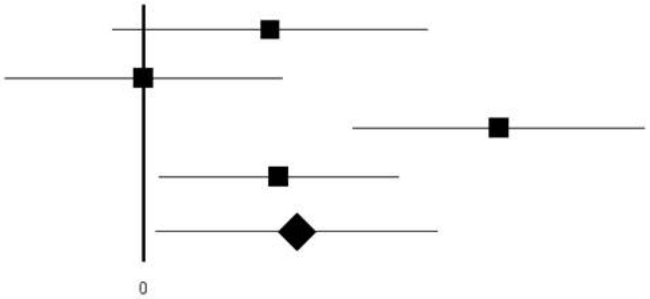

Heterogeneity: Cochran's Q = 12.21, df = 3 (p = 0.007), $Tau^2 = 0.29$, $I^2 = 75.43$.

2C.13 Repetitive Transcranial Magnetic Stimulation

2C.13-1 Description of studies and meta-analysis main results

We identified nine randomised controlled trials (RCTs) investigating repetitive transcranial magnetic stimulation (rTMS) for the treatment of vascular cognitive impairment (VCI). Of these, only three compared rTMS with an inactive control: two targeted the left dorsolateral prefrontal cortex (DLPFC), while one targeted the contralesional DLPFC. Unfortunately, the latter study [127] did not provide sufficient data to be included in the meta-analysis of primary efficacy outcomes and was retained solely for the evaluation of safety outcomes.

The two studies included in the meta-analysis applied high-frequency stimulation (≥ 5 Hz) to the left DLPFC at 80% of the resting motor threshold, administered daily five times per week over a four-week period (study [148] included also a second arm where stimulation over left DLPFC was combined with stimulation over ipsilesional primary motor area; to enhance comparability this arm was not included in meta-analysis).

We conducted a meta-analysis of global cognitive function using Montreal Cognitive Assessment (MoCA) scores of studies [122] and [148]. No adverse events were reported in either treatment arm of the studies that provided safety data [122, 127]. Data for functional outcome from study [122] were converted to effect size measures and are presented in the *Summary of Findings* table alongside the meta-analysed data.

The meta-analysis of rTMS effects on longitudinal changes in MoCA scores did not demonstrate a statistically significant effect (Cohen's d: 0.43, 95% CI [-0.38 to 1.23], p = 0.30; mean difference: 1.23, 95% CI [-1.16 to 3.62], p = 0.31). No significant effects on functional outcomes were reported by the single study that provided data in this category.

Results from the meta-analysis of global cognitive efficiency, along with available data for other outcomes, are summarised in the *Summary of Findings table* and *eFigure 64*.

2C.13-2 Characteristic of studies considered for meta-analysis

Table Caption: Characteristics of studies assessing *rTMS* for Vascular Cognitive Impairment.

Setting: hospital and clinics
Intervention: *rTMS (stimulation of the left dorsolateral prefrontal cortex, left DLPFC)*

Studies included in meta-analysis:

VCI population (label)	Treatment arms	Treatment duration/follow-up	Outcomes	Efficacy	Safety	Quality score*	Study
Post stroke cognitive impairment	*rTMS over left DFLPC - 5Hz, 80% resting motor threshold, 1200 total pulses – daily sessions 5 times/week (12)*<br><br>*vs*<br><br>*Sham stimulation (12)*	*4 weeks (NP)*	***<u>Primary outcomes</u>**: None reported*<br><br>*<u>Other outcomes</u>: Cognitive: yes Functional: no Patient-centred: no*	*In favour of treatment*	*No AE or SAE reported in either treatment arms*	**Overall**: Good<br><br>**QI**:<br>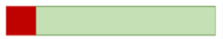	[122]
Post stroke cognitive impairment	*rTMS over left DFLPC - 10 Hz, 80% resting motor threshold, 2000 total pulses – daily sessions 5 times/week (15)*<br><br>*vs*<br><br>*Sham stimulation (18)*<br><br>*[vs rTMS over left DFLPC + ipsilesional M1- 10 Hz, 80% resting motor threshold, 2000 total*	*4 weeks (NP)*	***<u>Primary outcomes</u>**: Yes (MoCA)*<br><br>*<u>Other outcomes</u>: Cognitive: yes Functional: yes Patient-centred: no*	*In favour of treatment*	*Safety outcomes not reported*	**Overall**: Fair<br><br>**QI:**<br>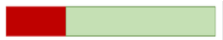	[148]

	pulses – daily sessions 5 times/week (15)]						

Studies included only in sensitivity analyses or excluded:

VCI population (label)	Treatment arms	Treatment duration/follow-up	Outcomes	Efficacy	Safety	Quality score*	Study
Post stroke cognitive impairment	*rTMS, controlesional DLPFC, frequency 1 Hz at 80% of resting motor threshold, 600 total pulses, 5 times/week*<br><br>*vs*<br><br>*Sham stimulation*	*8 weeks (NP)*	***Primary outcomes**: None reported*<br><br>*Other outcomes: Cognitive: yes<br>Functional: no<br>Patient-centred: no*	*In favour of treatment*	*No AE or SAE reported in either treatment arms*	**Overall**: Fair<br><br>**QI**:	[127]
Subcortical vascular cognitive impairment	*rTMS, left DLFPC + Donepezil 10 mg (58)*<br><br>*Vs*<br><br>*Donepezil 10 mg (57)*	*4-6 weeks (4 weeks)*<br><br>*[total: 28-42 sessions rTMS]*	*Primary outcomes<br>Cognitive: MMSE, MoCA<br>Functional: no<br>Patient-centred outcomes: no*<br><br>*No secondary outcomes*	*In favour of the combined therapy*	*No statistically significant difference in the incidence of adverse events in the two groups.*	***Overall**: Fair*<br><br>***QI:***	[150]
Post-stroke cognitive impairment*	*tDCS, left DLPFC<br>+ Sertraline 50 mg<br>+ Acupuncture<br>+ Cognitive training<br>+ Motor training<br>(15)*<br><br>*vs*	*4 weeks (NP)*<br><br>*[total: ~ 6.6 hours tDCS, 20 sessions acupuncture, ~ 20 sessions cognitive training, ~ 20*	*No primary outcomes*<br><br>*Outcomes<br>Cognitive: yes<br>Functional: no<br>Patient-centred outcomes: no*	*In favour of the combined therapy*	*No available data on safety*	***Overall**: Fair*<br><br>***QI***	[154]

	Sham tDCS, left DLPFC<br>+ Sertraline 50 mg<br>+ Acupuncture<br>+ Cognitive training<br>+ Motor training<br>(15)	sessions motor training]	Instrumental. fMRI, evoked potentials				
Post-stroke MCI*	*rTMS, DLPFC<br>+ Cognitive training<br>+ Motor training (16)<br><br>vs<br><br>Sham rTMS, DLPFC + Cognitive training<br>+ Motor training (15)*	*4 weeks (4 weeks)<br><br>[total: ~ 6.7 hours rTMS, ~ 10 hours cognitive training, ~ 13 hours motor training]*	*<u>No primary outcomes</u><br><br><u>Outcomes</u><br>Cognitive: yes<br>Functional: no<br>Patient-centred outcomes: no<br>Instrumental: Blood tests*	*Partially in favour of the treatment*	*No adverse effects reported*	***Overall**: Fair<br><br>**QI:**<br>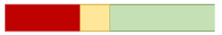*	[165]
Post-stroke cognitive impairment	*rTMS (prefrontal lobe)<br>+ Acupuncture (Xingnao Kaiqiao method) (90)<br><br>vs<br><br>rTMS (prefrontal lobe) (102)*	*4 weeks (NP)<br><br>[total: 20 sessions acupuncture, 20 sessions rTMS]*	*<u>No primary outcomes</u><br><br><u>Outcomes</u><br>Cognitive: yes<br>Functional: no<br>Patient-centred outcomes: no<br>Instrumental: Evoked potentials, Blood tests*	*In favour of the combined treatment*	*Only one AE (mild) reported in control group*	***Overall**: Poor<br><br>**QI:**<br>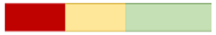*	[169]
Post-stroke MCI	*rTMS, left DLPFC + Computerized Cognitive training (16)<br><br>vs<br><br>Sham rTMS, left DLPFC + Computerized Cognitive training (18)*	*4 weeks (NP)<br><br>[total: ~ 6.6 hours rTMS, ~ 10 hours cognitive training]*	*<u>Primary outcomes</u><br>Cognitive: MoCA<br>Functional: no<br>Patient-centred outcomes: no<br><br><u>Secondary outcomes</u><br>Cognitive: yes<br>Functional: no<br>Patient-centred outcomes: no<br>Instrumental: fMRI*	*In favour of the combined treatment*	*No available data on safety*	***Overall**: Fair<br><br>**QI:**<br>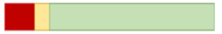*	[109]
Post-stroke cognitive impairment	*rTMS, left DLPFC + Cognitive training (15)<br><br>vs*	*3 weeks (NP)<br><br>[total: 15 rTMS sessions, ~ 7.5*	*<u>No primary outcomes</u><br><br><u>Outcomes</u><br>Cognitive: yes<br>Functional: no*	*In favour of the combined treatment*	*Only mild AE reported, not different between intervention arms*	***Overall**: Good<br><br>**QI**<br>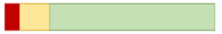*	[114]

	Sham rTMS, left DPLFC + Cognitive training (15)	*hours cognitive training]*	*Patient-centred outcomes: no Instrumental: fMRI*		

Abbreviations: AE, adverse events; DLPFC, dorsolateral prefrontal cortex; MCI, mild cognitive impairment; NP, not performed; rTMS, repetitive transcranial magnetic stimulation; SAE, severe adverse events; tDCS, transcranial Direct Current Stimulation.

Notes: * Overall quality as rated according to the NIH Quality Assessment tools for controlled intervention studies is reported here. QI (Quality Index) is a graphical, colour-coded representation of the number of items on the scale rated respectively as at high-risk (red), unclear risk (yellow) or low-risk (green) of bias.

2C.13-3 Summary of findings and figures for meta-analyses

Table Caption: Summary of findings for the main comparisons.

Setting: hospital and clinics
Intervention: *rTMS (stimulation of the left dorsolateral prefrontal cortex, left DLPFC)*
Comparator: *sham stimulation*

Outcomes	N° of participants (n of studies)	VCI population label	Efficacy measure	Quality of evidence (GRADE)	Statistical heterogeneity	Studies
Global cognitive efficiency *MoCA*<br><br>***Treatment duration:*** *4 weeks*<br><br>***Follow-up after treatment:*** *none*	*57 (2 RCTs)*	*Post-stroke cognitive impairment*	**Cohen's d**:<br>0.43<br>95% CI (-0.38 – 1.23)<br><br>**Mean difference**:<br>+ 1.23<br>95% CI (-1.16, 3.62)	⊕○○○ Very Low[3,4,5]	I^2=55.40	[122]<br>[148]
Functional outcomes *mBI*	*33 (1 RCT)*	*Post-stroke cognitive impairment*	**Cohen's d**[2]:<br>0.21<br>95% CI (-0.47 – 0.90)	⊕○○○ Very Low[3,5,6]		[148]
Patient-centred outcomes *Not reported*	*See note[1]*	*See note[1]*	*See note[1]*			
Safety outcomes *AE and SAE*		*Post-stroke cognitive impairment*	*Neither AE nor SAE were reported in any participant*			[122]<br>[127]

Abbreviations: AE, adverse events; 95%CI, 95% Confidence Interval; mBI, modified Barthel Index; MoCA, Montreal Cognitive Assessment; SAE, severe adverse events.

[1] No studies among the one included in meta-analysis reported functional-related or patient-centred outcomes.
[2] As data on functional outcomes were reported only in [148] the effect size reported reflects only the data reported in this single study (no-meta-analysis has been performed for this outcome).

GRADE Working Group grades of evidence:
High certainty: We are very confident that the true effect lies close to that of the estimate of the effect.

Moderate certainty: We are moderately confident in the effect estimate: the true effect is likely to be close to the estimate of the effect, but there is a possibility that it is substantially different.

Low certainty: Our confidence in the effect estimate is limited: the true effect may be substantially different from the estimate of the effect.

Very low certainty: We have very little confidence in the effect estimate: the true effect is likely to be substantially different from the estimate of effect.

[3]Some inconsistency in point estimates (downgraded once).

[4]Downgraded once due to imprecision: the 95% CI includes a result that would not be considered clinically important and a result that would be considered important.

[5]Two or more items at uncertain (or greater) risk of bias (downgraded once).

[6]Only one trial reporting on the outcome (downgraded once).

eFigure 64: *Forest plot representing meta-analysis of rTMS efficacy data on global cognitive efficiency scores (MoCA). Effect size is reported as Cohen's d in panel* ***a*** *and as unstandardised mean difference (MoCA points) in panel* ***b****. A random effect model was used for estimation.*

a

	ES	95% CI	W	SE	Sig.	N	N1	N2
Hu 2023	0.88	0.04 / 1.71	45.58%	0.43	0.040	24	12	12
Xu 2024	0.05	-0.64 / 0.74	54.42%	0.35	0.886	33	15	18
Overall (random-effects model)	0.43	-0.38 / 1.23	100.00%	0.41	0.300	57	27	30

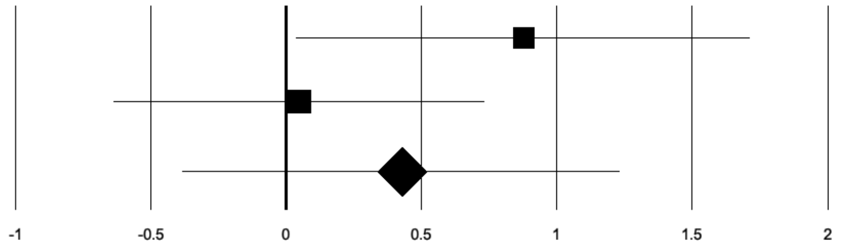

Heterogeneity: Cochran's Q = 2.24, df = 1 (p = 0.134), Tau² = 0.19, I² = 55.40

b

	ES	95% CI	W	SE	Sig.	N	N1	N2
Hu 2023	2.58	0.23 / 4.93	44.82%	1.20	0.032	24	12	12
Xu 2024	0.13	-1.65 / 1.91	55.18%	0.91	0.886	33	15	18
Overall (random-effects model)	1.23	-1.16 / 3.62	100.00%	1.22	0.313	57	27	30

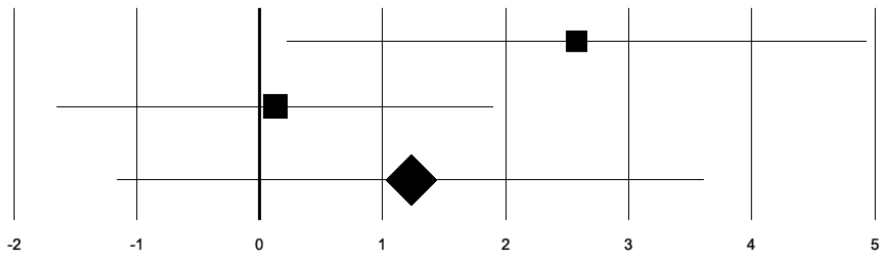

Heterogeneity: Cochran's Q = 2.65, df = 1 (p = 0.103), Tau² = 1.87, I² = 62.30

2C.14 Transcranial Direct Current Stimulation

2C.14-1 Description of studies and meta-analysis main results

We identified seven randomised controlled trials (RCTs) investigating transcranial direct current stimulation (tDCS) for the treatment of vascular cognitive impairment (VCI). Of these, only three compared tDCS with an inactive control, all targeting the left dorsolateral prefrontal cortex (DLPFC).

One study [125] reported data solely on neuropsychological attention scores, without providing information on other outcome domains or safety, and was therefore excluded from the meta-analysis.

The two studies included in the meta-analysis applied direct current stimulation (2 mA) to the left DLPFC for 20–30 minutes. Treatment duration varied: study [159] administered daily stimulation for four consecutive days, while study [124] delivered stimulation five times per week over two weeks.

Both studies assessed global cognitive efficiency, with outcomes measured at the end of treatment. Additionally, study [159] evaluated cognitive outcomes 10 days post-treatment. Neither study reported data on functional or patient-centred outcomes. Only study [124] provided safety data, stating that no adverse events occurred in either group; study [159] did not report on safety.

We conducted a meta-analysis of global cognitive function. The analysis of tDCS effects on longitudinal changes in global cognitive efficiency did not reveal a statistically significant effect (Cohen's d: 1.20, 95% CI [-0.70 to 3.11], p = 0.30). Results from the meta-analysis of global cognitive efficiency are summarised in the *Summary of Findings table* and *eFigure 65*.

2C.14-2 Characteristic of studies considered for meta-analysis

Table Caption: Characteristics of studies assessing *transcranial Direct Current Stimulation (tDCS)* for Vascular Cognitive Impairment.

Setting: hospital and clinics
Intervention: *tDCS (left dorsolateral prefrontal cortex, DLPFC)*

Studies included in meta-analysis:

VCI population (label)	Treatment arms	Treatment duration/follow-up	Outcomes	Efficacy	Safety	Quality score*	Study
Post-stroke dementia	*tDCS, left DLPFC (38) stimulation intensity 2 mA for 30 minutes, daily*<br><br>*Sham tDCS, left DLPFC (82)*	*4 days (10 days)*	***Primary outcomes**: Yes (MoCA)*<br><br>*Other outcomes: Cognitive: yes Functional: no Patient-centred: no*	*In favour of treatment*	*Two participants reported to have discontinued due to transient AEs (mild); no other safety outcomes reported.*	***Overall**: Good<br><br>**QI**:<br>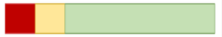	[159]
Vascular dementia	*tDCS, left DLPFC stimulation intensity 2 mA for 20 minutes, daily 5 times/week (13)*<br><br>*vs*<br><br>*Sham tDCS, left DLPFC (8)*	*2 weeks (NP)*<br><br>[*total: ~ 80 minutes*]	*Primary outcomes Cognitive: ADAS-Cog Functional: no Patient-centred outcomes: no*<br><br>*Secondary outcomes Cognitive: yes Functional: yes Patient-centred outcomes: no*	*In favour of the treatment*	*No adverse effect reported*	***Overall**: Fair*<br><br>***QI**:*<br>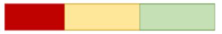	[124]

Studies included only in sensitivity analyses or excluded:

VCI population (label)	Treatment arms	Treatment duration/follow-up	Outcomes	Efficacy	Safety	Quality score*	Study
Post-stroke cognitive impairment	*Crossover-design*<br><br>*tDCS, left DLPFC (10)*<br><br>*vs*<br><br>*Sham tDCS, left DLPFC (10)*	*1 day (NP)*<br><br>*[total: ~20 minutes]*	*<u>Primary outcomes</u>*<br>*Cognitive: NPS attentive functions*<br>*Functional: no*<br>*Patient-centred outcomes: no*<br><br>*<u>No other outcomes</u>*	*Neutral*	*No data on safety available*	***Overall**: Fair*<br><br>***QI**:*<br>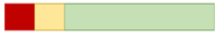	[125]
Post-stroke cognitive impairment*	*tDCS, left DLPFC*<br>*+ Sertraline 50 mg*<br>*+ Acupuncture*<br>*+ Cognitive training*<br>*+ Motor training*<br>*(15)*<br><br>*vs*<br><br>*Sham tDCS, left DLPFC*<br>*+ Sertraline 50 mg*<br>*+ Acupuncture*<br>*+ Cognitive training*<br>*+ Motor training*<br>*(15)*	*4 weeks (NP)*<br><br>*[total: ~6.6 hours tDCS, 20 sessions acupuncture, ~20 sessions cognitive training, ~20 sessions motor training]*	*<u>No primary outcomes</u>*<br><br>*<u>Outcomes</u>*<br>*Cognitive: yes*<br>*Functional: no*<br>*Patient-centred outcomes: no*<br>*Instrumental. fMRI, evoked potentials*	*In favour of the combined therapy*	*No available data on safety*	***Overall**: Fair*<br><br>***QI***<br>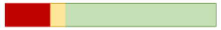	[154]
Post-stroke cognitive impairment	*tDCS, DLFPFC (30)*<br><br>*vs*<br><br>*Motor training*<br>*+ Cognitive training*<br>*(30)*	*4 weeks (NP)*<br><br>*[total: ~6.7 hours tDCS, ~6.7 hours motor training, ~ 6.7 hours cognitive training]*	*<u>No primary outcomes</u>*<br><br>*<u>Outcomes</u>*<br>*Cognitive: yes*<br>*Functional: no*<br>*Patient-centred outcomes: no*	*In favour of the combined therapy*	*2 participants in the tDCS group experienced mild adverse reactions*	***Overall**: Fair*<br><br>***QI**:*<br>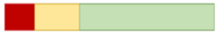	[123]

	vs<br><br>*tDCS*<br>*+ Motor training*<br>*+ Cognitive training*<br>*(tDCS simultaneously with cognitive rehabilitation) (30)*						
Post-stroke cognitive impairment	*Home-based tDCS, left DLFPC + Computerized cognitive training (12)*<br><br>*vs*<br><br>*Sham tDCS + Computerized cognitive training (14)*	*4 weeks (NP)*<br><br>*[total: ~ 10 hours tDCS, ~ 10 hours computerized cognitive training]*	*<u>Primary outcomes</u>*<br>*Cognitive: MoCA, Dementia rating scale - attentive functions*<br>*Functional: no*<br>*Patient-centred outcomes: no*<br><br>*<u>Secondary outcomes</u>*<br>*Cognitive: yes*<br>*Functional: no*<br>*Patient-centred outcomes: no*	*Partially in favour of the combined treatment*	*No serious adverse effects reported*	***Overall**: Good*<br><br>***QI***<br>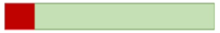	[132]
Post-stroke cognitive impairment	*tDCS, left DLPFC + Computerized cognitive training (simultaneous) (18)*<br><br>*vs*<br><br>*Computerized cognitive training (18)*<br><br>*vs*<br><br>*tDCS, left DLPFC (18)*<br><br>*vs*<br><br>*Cognitive training (18)*	*3 weeks (NP)*<br><br>*[total: ~ 5 hours tDCS, ~ 5 hours computerized cognitive training; ~ 5 hours cognitive training]*	*<u>No primary outcomes</u>*<br><br>*<u>Outcomes</u>*<br>*Cognitive: yes*<br>*Functional: yes*<br>*Patient-centred outcomes: no*<br>*Instrumental. TC-US*	*In favour of the combined therapy*	*One subject experienced skin redness after the first tDCS treatment.*	***Overall**: Good*<br><br>***QI***<br>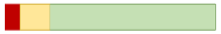	[158]

Abbreviations: AE, adverse events; DLPFC, dorsolateral prefrontal cortex; NP, not performed; SAE, severe adverse events.

Notes: * Overall quality as rated according to the NIH Quality Assessment tools for controlled intervention studies is reported here. QI (Quality Index) is a graphical, colour-coded representation of the number of items on the scale rated respectively as at high-risk (red), unclear risk (yellow) or low-risk (green) of bias.

2C.14-3 Summary of findings and figures for meta-analyses

Table Caption: Summary of findings for the main comparisons.

Setting: hospital and clinics
Intervention: *tDCS (left dorsolateral prefrontal cortex, DLPFC)*
Comparator: *sham stimulation*

Outcomes	N° of participants (n of studies)	VCI population label	Efficacy measure	Quality of evidence (GRADE)	Statistical heterogeneity	Studies
Global cognitive efficiency *ADAS-Cog, MoCA*<br><br>***Treatment duration**: 2 weeks*<br><br>***Follow-up after treatment**: none*	*97 (2 RCTs)*	*Vascular Dementia*<br>*Post-stroke dementia*	**Cohen's d:**<br>1.20<br>95% CI (-0.70 – 3.11)	⊕○○○ Very Low[2,3,4,5]	I^2= 92.43%	[159]<br>[124]
Functional outcomes Not reported	*See note[1]*	*See note[1]*	*See note[1]*			
Patient-centred outcomes Not reported	*See note[1]*	*See note[1]*	*See note[1]*			
Safety outcomes Insufficient data			*No adverse events reported by only one study*			[124]

Abbreviations: ADAS-Cog, Alzheimer's Disease Assessment Scale cognitive subscale; AE, adverse events; 95%CI, 95% Confidence Interval; MoCA, Montreal Cognitive Assessment; SAE, severe adverse events.

[1] No studies among the one included in meta-analysis reported functional-related or patient-centred outcomes.

GRADE Working Group grades of evidence:

High certainty: We are very confident that the true effect lies close to that of the estimate of the effect.

Moderate certainty: We are moderately confident in the effect estimate: the true effect is likely to be close to the estimate of the effect, but there is a possibility that it is substantially different.

Low certainty: Our confidence in the effect estimate is limited: the true effect may be substantially different from the estimate of the effect.

Very low certainty: We have very little confidence in the effect estimate: the true effect is likely to be substantially different from the estimate of effect.

[2]Inconsistency in point estimates (downgraded once).

[3]Downgraded twice due to imprecision (wide 95% confidence interval)

[4]Two or more items at uncertain (or greater) risk of bias (downgraded once).

[5]Different outcome measures employed (downgraded once).

eFigure 65: *Forest plot representing meta-analysis of tDCS efficacy data on global cognitive efficiency. Effect size is reported as Cohen's d; a random effects model was used for estimation.*

	ES	95% CI	W	SE	Sig.	N	N1	N2
Ai 2024	2.14	1.58 / 2.71	51.59%	0.29	0.000	76	38	38
Andre 2016	0.20	-0.68 / 1.08	48.41%	0.45	0.657	21	13	8
Overall (random-effects model)	1.20	-0.70 / 3.11	100.00%	0.97	0.216	97	51	46

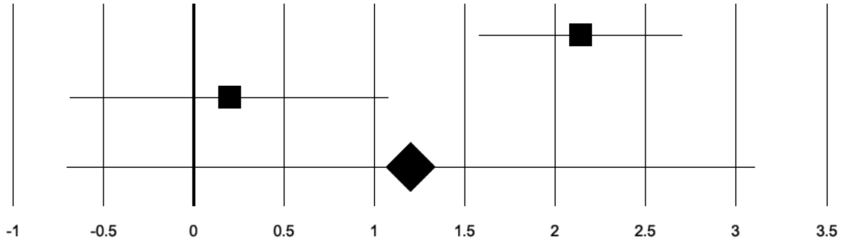

Heterogeneity: Cochran's Q = 13.21, df = 1 (p < 0.001), $Tau^2 = 1.74$*,* $I^2 = 92.43$

2C.14-4 Sensitivity Analyses

We performed sensitivity analyses varying $Corr_{pre\text{-}post}$ for global cognitive efficiency outcomes, as $Corr_{pre\text{-}post}$ was not reported nor inferable from previous studies for this class of outcomes.

Effect size was overall superimposable for $Corr_{pre\text{-}post}$ variation (effect size variation, *Cohen's d*, - 0.03 ie, ± 3% in proportion with main analysis). Forest plots for these analyses as well as their relative heterogeneity statistics are reported in *eFigure 66*.

*eFigure 66: Sensitivity analysis showing tDCS effect on global cognitive efficiency primary outcomes, variating pre-post correlation coefficients between 0 and 0.8 (panel **a** and panel **b** respectively). Effect sizes are reported as Cohen's d; random effects models were used for estimation.*

a

	ES	95% CI	W	SE	Sig.	N	N1	N2
Ai 2024	2.14	1.58 / 2.71	51.49%	0.29	0.000	76	38	38
Andre 2016	0.14	-0.74 / 1.02	48.51%	0.45	0.753	21	13	8
Overall (random-effects model)	1.17	-0.79 / 3.13	100.00%	1.00	0.241	97	51	46

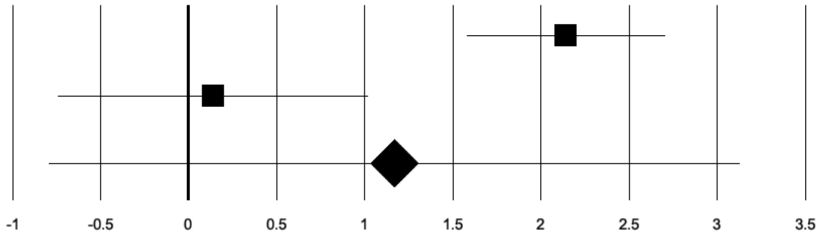

Heterogeneity: Cochran's Q = 14-05, df = 1 (p < 0.001), Tau^2 = 1.86, I^2 = 92.88

b

	ES	95% CI	W	SE	Sig.	N	N1	N2
Ai 2024	2.14	1.58 / 2.71	51.82%	0.29	0.000	76	38	38
Andre 2016	0.32	-0.57 / 1.20	48.18%	0.45	0.484	21	13	8
Overall (random-effects model)	1.26	-0.53 / 3.05	100.00%	0.91	0.167	97	51	46

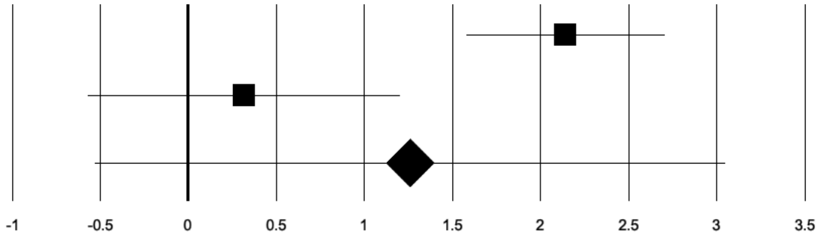

Heterogeneity: Cochran's Q = 11.62, df = 1 (p < 0.001), Tau^2 = 1.53, I^2 = 91.40

2C.15 Physical Exercise

2C.15-1 Description of studies and meta-analysis main results

We identified five randomised controlled trials (RCTs) investigating physical exercise as a treatment for vascular cognitive impairment (VCI). Of these, only three evaluated physical exercise as a monotherapy compared with an inactive control (usual care), while the remaining two either combined it with other intervention strategies or compared it with other active interventions.

One study [149], although reporting data on global cognitive efficiency and functional outcomes, did not provide sufficient information for data pooling and was therefore excluded from the final meta-analysis.

The two studies included in the meta-analysis involved thrice-weekly physical exercise sessions lasting 40–60 minutes over a period of 24 weeks/six months. Both studies assessed global cognitive efficiency, with outcomes measured at the end of treatment and after variable follow-up periods (four weeks for study [133], six months for study [137]). Additionally, study [137] evaluated functional outcomes both post-treatment and post-follow-up. Effect size data from this study are presented in the *Summary of Findings table* alongside the meta-analysed data. Neither study reported data on functional or patient-centred outcomes.

We therefore conducted a meta-analysis of global cognitive function. The analysis of physical exercise effects on longitudinal changes in global cognitive efficiency revealed a statistically significant effect of moderate magnitude (Cohen's d= 0.60, 95% CI [0.23–0.97], p = 0.001).

Results from the meta-analysis of global cognitive efficiency are summarised in the *Summary of Findings table* and *eFigure 67*.

2C.15-2 Characteristic of studies considered for meta-analysis

Table Caption: Characteristics of studies assessing *physical exercixe* for Vascular Cognitive Impairment.

Setting: hospital/clinics and community centres
Intervention: *physical exercise*

Studies included in meta-analysis:

VCI population (label)	Treatment arms	Treatment duration/follow-up	Outcomes	Efficacy	Safety	Quality score*	Study
Subcortical vascular MCI	*Aerobic Exercise (35)*<br><br>*vs*<br><br>*Usual care (35)*	*6 months*<br>[*total: ~48 hours*]<br><br>*(follow-up: 6 months)*	***Primary outcomes***<br>***Cognitive: ADAS-Cog, EXIT-25***<br>***Functional: ADCS-ADL***<br><br>*Secondary outcomes*<br>*Cognitive: yes*<br>*Functional: no*<br>*Patient-centred: no*<br>*Instrumental: yes (vital signs)*	*In favour of treatment*		***Overall**: Good*<br><br>***QI**:*<br>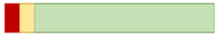	[137]
Post-stroke cognitive impairment	*Baduanjin exercise (22)*<br><br>*vs*<br><br>*Usual care (19)*	*24 weeks*<br>[*total: ~48 hours*]<br><br>*(follow-up: 1 month)*	***Primary outcomes***<br>***Cognitive: MoCA***<br><br>*Secondary outcomes*<br>*Cognitive: yes*<br>*Functional: yes*<br>*Patient-centred: no*	*In favour of treatment*		***Overall**: Good*<br><br>***QI**:*<br>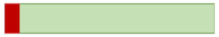	[133]

Studies included only in sensitivity analyses or excluded:

VCI population (label)	Treatment arms	Treatment duration/follow-up	Outcomes	Efficacy	Safety	Quality score*	Study
Vascular MCI	*Physical activity (53)*<br><br>*vs*<br><br>*usual care (51)*	*24 weeks*<br>*[total: ~ 72 hours]*<br><br>*(follow-up: NP)*	***<u>Primary outcomes</u>***<br>***Cognitive: cognitive decline to dementia***<br><br>*<u>Secondary outcomes</u>*<br>*Cognitive: yes*<br>*Functional: yes*<br>*Patient-centred: yes*	*Neutral*		***Overall**: Good*<br><br>***QI**:*<br>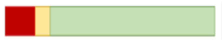	[149]
Post-stroke MCI	*Aerobic exercise group + Occupational therapy + motor rehabilitation + Acupuncture (10)*<br><br>*Vs*<br><br>*Occupational therapy + motor rehabilitation + Acupuncture (10)*	*2 weeks*<br>*[total: ~ 8 hours]*<br><br>*(follow-up: NP)*	***<u>Primary outcomes</u>***<br>***none***<br><br>*<u>Other outcomes</u>*<br>*Cognitive: yes*<br>*Functional: no*<br>*Patient-centred: no*	*In favour of treatment*	*No safety data reported*	***Overall**: Poor*<br><br>***QI**:*<br>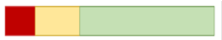	[155]
Post-stroke cognitive impairment	*Physical exercise group (56)*<br><br>*vs*<br><br>*Cognitive training group (57)*<br><br>*vs*<br><br>*Combined physical exercise and cognitive training group (55)*<br><br>*vs*	*12 weeks*<br>*[total: ~ 36 hours]*<br><br>*(follow-up: 6 months)*	***<u>Primary outcomes</u>***<br>***none***<br><br>*<u>Other outcomes</u>*<br>*Cognitive: yes*<br>*Functional: no*<br>*Patient-centred: no*	*In favour of treatment*		***Overall**: Good*<br><br>***QI**:*<br>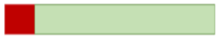	[142]

	Control group (57)				

Abbreviations: ADAS-Cog, Alzheimer's Disease Assessment Scale – cognitive subscale; ADCS-ADL, Alzheimer's Disease Cooperative Study-ADL; AE, adverse events; EXIT-25, executive interview; MoCA, Montreal Cognitive Assessment; NP, not performed; SAE, severe adverse events.

Notes: * Overall quality as rated according to the NIH Quality Assessment tools for controlled intervention studies is reported here. QI (Quality Index) is a graphical, colour-coded representation of the number of items on the scale rated respectively as at high-risk (red), unclear risk (yellow) or low-risk (green) of bias.

2C.15-3 Summary of findings and figures for meta-analyses

Table Caption: Summary of findings for the main comparisons.

Setting: hospital/clinics and community centres
Intervention: *physical exercise*
Comparator: *usual care*

Outcomes	N° of participants (n of studies)	VCI population label	Efficacy measure	Quality of evidence (GRADE)	Statistical heterogeneity	Studies
Global cognitive efficiency *ADAS-Cog, MoCA*<br><br>***Treatment duration:*** *24 weeks – 6 months*<br><br>***Follow-up after treatment:*** *1 – 6 months*	*118 (2 RCTs)*	*Subcortical Vascular MCI*<br>*Post-stroke cognitive impairment*	***At the end of treatment:*** *Cohen's d*: 0.60 95% CI (0.23-0.97)<br><br>***At the end of follow-up:*** *Cohen's d*: 0.50 95% CI (-0.17-1.16)	⊕○○○ Very Low[3,4,5,6]<br><br>⊕○○○ Very Low[3,4,6,7]	I^2= 0<br><br>I^2= 67.59	[137]<br>[133]
Functional outcomes *ADCS-ADL*	*70 (1 RCTs)*	*Subcortical Vascular MCI*	***At the end of treatment:*** *Cohen's d*: 0.32 95% CI (-0.16-0.79)<br><br>***At the end of follow-up:*** *Cohen's d*: 0.32 95% CI (-0.15-0.79)	⊕⊕○○ Low[3,8,9]<br><br>⊕⊕○○ Low[3,8,9]	*See note[1]*	[137]

Patient-centred outcomes<br>*Not reported*	*See note*[2]	*See note*[2]	*See note*[2]	
Safety outcomes<br>*AE and SAE*			*AE/SAE: none reported/not applicable*	

Abbreviations: AE, adverse events; 95%CI, 95% Confidence Interval; SAE, severe adverse events.

[1] As data on functional outcomes were reported only in [137] the effect size reported reflects only the data reported in this single study (no-meta-analysis has been performed for this outcome).
[2] No studies among the one included in meta-analysis reported functional-related or patient-centred outcomes.

GRADE Working Group grades of evidence:

High certainty: We are very confident that the true effect lies close to that of the estimate of the effect.

Moderate certainty: We are moderately confident in the effect estimate: the true effect is likely to be close to the estimate of the effect, but there is a possibility that it is substantially different.

Low certainty: Our confidence in the effect estimate is limited: the true effect may be substantially different from the estimate of the effect.

Very low certainty: We have very little confidence in the effect estimate: the true effect is likely to be substantially different from the estimate of effect.

[3]Some inconsistency in point estimates (downgraded once).

[4]Downgraded once due to imprecision: the 95% CI includes a result that would not be considered clinically important and a result that would be considered important.

[5]Low generalisability due to inclusion of different VCI populations (downgraded once for indirectness)

[6]Different outcome measures employed (downgraded once).

[7]Low generalisability due to inclusion of different VCI populations and due to different follow-up timespans (despite comparable treatment durations). Downgraded twice for indirectness.

[8]Only one trial reporting on the outcome (downgraded once).

[9]Some imprecision with wide estimate 95% CI (downgraded once)

*eFigure 67: Forest plot representing meta-analysis of physical exercise efficacy data on global cognitive efficiency outcomes. Effect size is reported as Cohen's d after the end of treatment (panel **a**) and after follow-up (panel **b**). Fixed or random effects models were used for estimation as appropriate.*

a

	ES	95% CI	W	SE	Sig.	N	N1	N2
Liu-Ambrose 2016	0.56	0.08 / 1.04	59.64%	0.24	0.021	70	35	35
Zheng 2020	0.65	0.07 / 1.23	40.36%	0.30	0.028	48	24	24
Overall (fixed-effect model)	0.60	0.23 / 0.97	100.00%	0.19	0.001	118	59	59

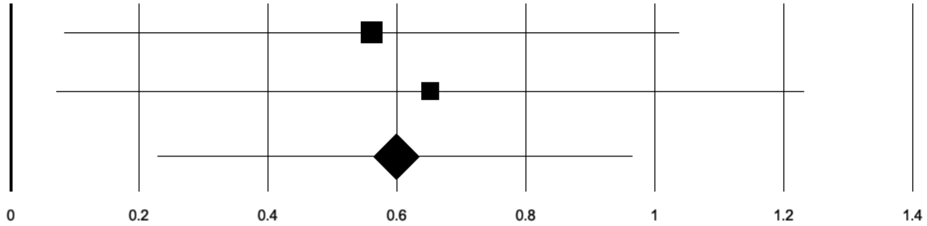

Heterogeneity: Cochran's Q = 0.06, df = 1 (p = 0.812), Tau² = 0, I² = 0

b

	ES	95% CI	W	SE	Sig.	N	N1	N2
Liu-Ambrose 2016	0.18	-0.29 / 0.65	53.67%	0.24	0.446	70	35	35
Zheng 2020	0.86	0.27 / 1.45	46.33%	0.30	0.004	48	24	24
Overall (random-effects model)	0.50	-0.17 / 1.16	100.00%	0.34	0.141	118	59	59

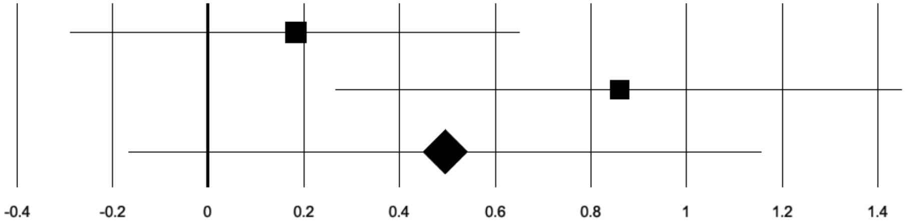

Heterogeneity: Cochran's Q = 3.09, df = 1 (p = 0.079), Tau² = 0.15, I² = 67.59

2C.15-4 Sensitivity Analyses

We performed sensitivity analyses varying $Corr_{pre\text{-}post}$ for global cognitive efficiency outcomes at both timepoints, as $Corr_{pre\text{-}post}$ was not reported nor inferable from previous studies for this class of outcomes.

Effect size differed slightly for $Corr_{pre\text{-}post}$ variation after the end of treatment (*Cohen's d* ranging from - 0.09 to +0.11, ie, approximately 15-20% relative to the main analysis), although statistical significance was consistently maintained.

Effect size varied more substantially with changes in $Corr_{pre\text{-}post}$ variation after the end of follow-up (*Cohen's d* ranging from - 0.13 to +0.22, ie, approximately ±25–40% relative to the main analysis), but statistical significance was never achieved.

Forest plots for these analyses, along with their corresponding heterogeneity statistics, are presented in *eFigure 68-69*.

__eFigure 68__: Sensitivity analysis showing physical exercise effect on global cognitive efficiency primary outcomes after the end of treatment, variating pre-post correlation coefficients between 0 and 0.8 (panel __a__ and panel __b__ respectively). Effect sizes are reported as Cohen's d; fixed effects models were used for estimation.

a

	ES	95% CI	W	SE	Sig.	N	N1	N2
Liu-Ambrose 2016	0.56	0.08 / 1.04	59.07%	0.24	0.021	70	35	35
Zheng 2020	0.48	-0.10 / 1.05	40.93%	0.29	0.104	48	24	24
Overall (fixed-effect model)	0.53	0.16 / 0.89	100.00%	0.19	0.005	118	59	59

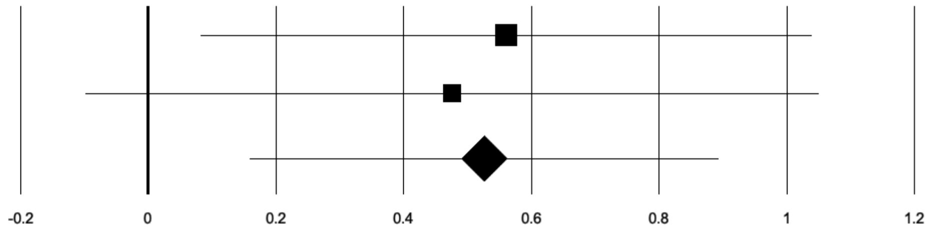

Heterogeneity: Cochran's Q = 0.05, df = 1 (p = 0.824), Tau^2 = 0, I^2 = 0

b

	ES	95% CI	W	SE	Sig.	N	N1	N2
Liu-Ambrose 2016	0.56	0.08 / 1.04	60.93%	0.24	0.021	70	35	35
Zheng 2020	0.95	0.35 / 1.54	39.07%	0.30	0.002	48	24	24
Overall (fixed-effect model)	0.71	0.34 / 1.08	100.00%	0.19	0.000	118	59	59

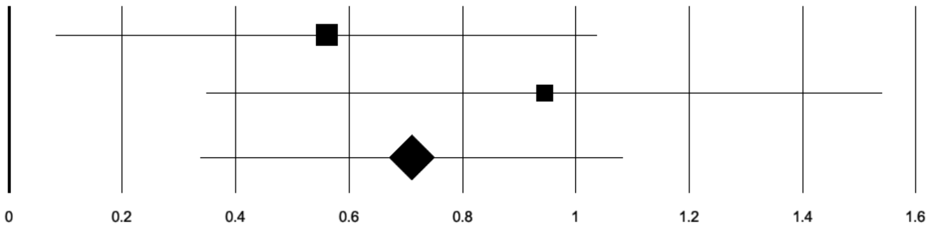

Heterogeneity: Cochran's Q = 0.97, df = 1 (p = 0.324), Tau^2 = 0, I^2 = 0

eFigure 69*: Sensitivity analysis showing physical exercise effect on global cognitive efficiency primary outcomes after the end of follow-up, variating pre-post correlation coefficients between 0 and 0.8 (panel **a** and panel **b** respectively). Effect sizes are reported as Cohen's d; random effects models were used for estimation.*

a

	ES	95% CI	W	SE	Sig.	N	N1	N2
Liu-Ambrose 2016	0.18	-0.29 / 0.65	57.90%	0.24	0.446	70	35	35
Zheng 2020	0.62	0.04 / 1.20	42.10%	0.30	0.037	48	24	24
Overall (random-effects model)	0.37	-0.06 / 0.79	100.00%	0.21	0.089	118	59	59

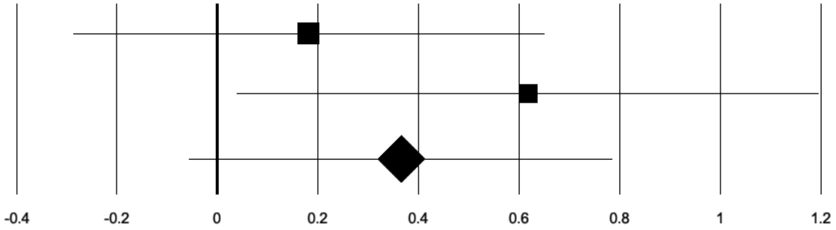

Heterogeneity: Cochran's Q = 1.31, df = 1 (p = 0.253), Tau^2 = 0.02, I^2 = 23.58

b

	ES	95% CI	W	SE	Sig.	N	N1	N2
Liu-Ambrose 2016	0.18	-0.29 / 0.65	51.75%	0.24	0.446	70	35	35
Zheng 2020	1.30	0.67 / 1.92	48.25%	0.32	0.000	48	24	24
Overall (random-effects model)	0.72	-0.37 / 1.81	100.00%	0.56	0.196	118	59	59

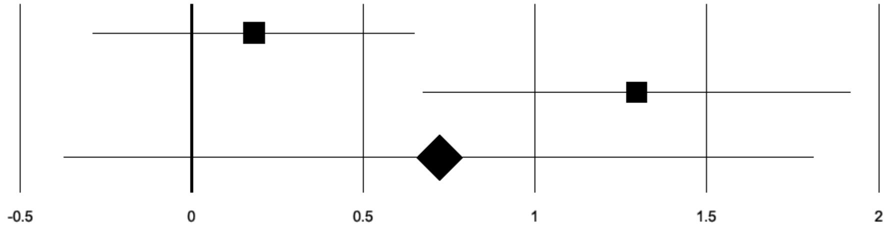

Heterogeneity: Cochran's Q = 7.85, df = 1 (p = 0.005), Tau^2 = 0.54, I^2 = 87.26

2C.16 All-treatment meta-analysis on safety outcomes

***eFigure 70** – Forest plot representing meta-analysis of safety outcomes (general adverse events) for all interventions considered for meta-analysis. Effects size is reports as rate ratio.*

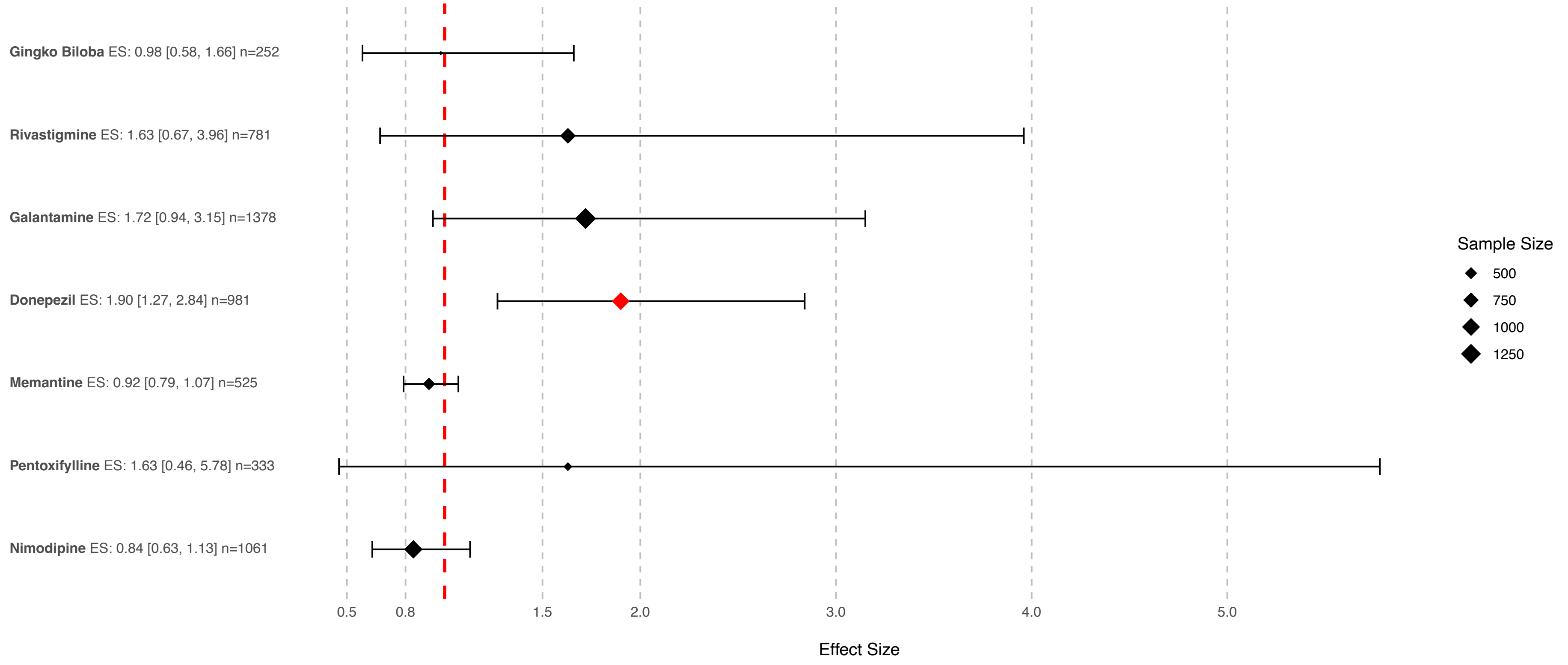

3. SUPPLEMENTARY TABLES

3.1 All treatments (not included in meta-analyses) in alphabetical order

3.1.1 *eTable 2* – Qualitative summary of study characteristics and efficacy of pharmacological interventions

Intervention	VCI population	Treatment arms	Treatment duration (follow-up)[$]	Outcomes	Efficacy	Safety profile	Quality score*	Study
Acetylsalicylic acid	*Multi-infarct dementia*	*ASA 325 mg (37)*<br><br>*vs*<br><br>*Best medical treatment (33)*	*3 years (NP)*	<u>*No primary outcomes*</u><br><br><u>*Outcomes*</u><br>*Cognitive: yes*<br>*Functional: no*<br>*Patient-centred outcomes: no*<br>*Instrumental: Regional cerebral blood flow*	*In favour of the treatment*	*No available data on safety*	***Overall**: Fair*<br><br>***QI**:*<br>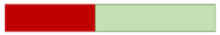	[55]
Actovegin	*Post-stroke MCI*	*Actovegin 2000 mg ev daily for ≤20 infusions followed by 1200 mg os daily (248)*<br><br>*vs*<br><br>*Placebo (255)*	*6 months (6 months)*	<u>*Primary outcomes*</u><br>*Cognitive: ADAS-Cog+*<br>*Functional: no*<br>*Patient-centred outcomes: no*<br><br><u>*Secondary outcomes*</u><br>*Cognitive: yes*<br>*Functional: yes*<br>*Patient-centred outcomes: yes*	*In favour of the treatment*	*No available data on safety*	***Overall**: Good*<br><br>***QI**:*<br>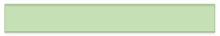	[76]
Buflomedil	*Vascular dementia*	*Treatment (T): Buflomedil 600 mg; Placebo (P); No-treatment (NT)*<br><br>*Arm 1 = T + T + T*<br>*Arm 2 = T + T + N*<br>*Arm 3 = P + T + T*<br>*Arm 4 = P + T + NT*	*90 days + 90 days + 90 days (NP)*	<u>*No primary outcomes*</u><br><br><u>*Outcomes*</u><br>*Cognitive: yes*<br>*Functional: yes*<br>*Patient-centred outcomes: no*	*In favour of the treatment*	*No available data on safety*	***Overall**: Good*<br><br>***QI**:*<br>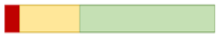	[70]
Bromocriptine	*Vascular dementia*	*Crossover design: Bromocriptine 30 mg (7)*<br><br>*vs*<br><br>*Placebo (7)*	*Crossover: 4 months + 4 months (NP)*	<u>*No primary outcomes*</u><br><br><u>*Outcomes*</u><br>*Cognitive: yes*<br>*Functional: no*	*Neutral*	*No available data on safety*	***Overall**: Fair*<br><br>***QI**:*<br>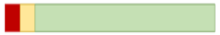	[24]

				Patient-centred outcomes: no				
Brovincamine/Vincamine	*Multi-infarct dementia*	*Crossover design: Brovincamine 80 mg g (10)*<br><br>*vs*<br><br>*Vincamine 80 mg (10)*<br><br>*vs*<br><br>*Placebo (10)*	*Crossover:*<br>*2 weeks +*<br>*2 weeks +*<br>*2 weeks (NP)*	*<u>No primary outcomes</u>*<br><br>*<u>Outcomes</u>*<br>*Cognitive: yes*<br>*Functional: no*<br>*Patient-centred outcomes: no*<br>*Instrumental: Regional cerebral blood flow*	*Partially in favour of both treatments*	*No available data on safety*	***Overall**: Fair*<br><br>***QI**:*<br>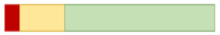	[74]
Butylphtalyde	*Vascular dementia*	*Butylphtalyde 0.6 g (62)*<br><br>*vs*<br><br>*Donepezil 5 mg (62)*	*3 months (NP)*	*<u>No primary outcomes</u>*<br><br>*<u>Outcomes</u>*<br>*Cognitive: yes*<br>*Functional: yes*<br>*Patient-centred outcomes: no*<br>*Instrumental: Blood tests, TC-US*	*In favour of the treatment*	*Incidence of adverse reactions in the intervention group was 14.52%, vs 6.45% in the control group. The difference was not statistically significant.*	***Overall**: Fair*<br><br>***QI**:*<br>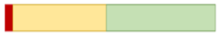	[13]
	Subcortical vascular MCI	*Butylphtalyde 600 mg (140)*<br><br>*vs*<br><br>*Placebo (140)*	*24 weeks (NP)*	*<u>Primary outcomes</u>*<br>*Cognitive: ADAS-Cog*<br>*Functional: CIBIC-plus*<br>*Patient-centred outcomes: no*<br><br>*<u>Secondary outcomes</u>*<br>*Cognitive: yes*<br>*Functional: no*<br>*Patient-centred outcomes: no*	*In favour of the treatment*	*No available data on safety*	***Overall**: Good*<br><br>***QI**:*<br>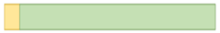	[39]
	Post-stroke Cognitive Impairment	*Butylphtalyde 600 mg (43)*<br><br>*vs*<br><br>*Placebo (39)*	*8 weeks (NP)*	*<u>No primary outcomes</u>*<br><br>*<u>Outcomes</u>*<br>*Cognitive: yes*<br>*Functional: yes*<br>*Patient-centred outcomes: no*<br>*Instrumental: MR metrics*	*In favour of the treatment*	*No available data on safety*	***Overall**: Poor*<br><br>***QI**:*<br>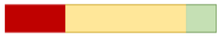	[173]
Butylphtalyde + Dengzhan Shengmai	*Vascular dementia*	*Butylphtalyde 600 mg (41)*<br><br>*vs*	*3 months (NP)*	*<u>No primary outcomes</u>*<br><br>*<u>Outcomes</u>*<br>*Cognitive: yes*<br>*Functional: yes*	*In favour of the combined treatment*	*There was no statistically significant difference in the incidence of*	***Overall**: Poor*<br><br>***QI**:*<br>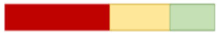	[96]

		Dengzhan Shengmai 1.08 g (41)<br><br>*vs*<br><br>*Butylphtalyde 600 mg + Dengzhan Shengmai 1.08 g (41)*		*Patient-centred outcomes: no*<br>*Instrumental: Blood tests*		*adverse reactions among the three groups (p>0.05).*		
Butylphtalyde + Piracetam	*Vascular dementia*	*Butylphtalyde 0.6 g + Piracetam 2.4 g (86)*<br><br>*vs*<br><br>*Piracetam 2.4 g (86)*	*12 weeks (NP)*	*No primary outcomes*<br><br>*Outcomes*<br>*Cognitive: yes*<br>*Functional: yes*<br>*Patient-centred outcomes: no*<br>*Instrumental: Blood tests*	*In favour of the combined treatment*	*No available data on safety*	***Overall**: Poor*<br><br>***QI**:*	[73]
Butylphtalyde + Piracetam + Idebenone	*Vascular dementia*	*Butylphtalyde 0.6 g + Piracetam 8 g + Idebenone 90 mg (44)*<br><br>*vs*<br><br>*Piracetam 8 g + Idebenone 90 mg (44)*	*12 weeks (NP)*	*No primary outcomes*<br><br>*Outcomes*<br>*Cognitive: yes*<br>*Functional: yes*<br>*Patient-centred outcomes: no*<br>*Instrumental: Blood test*	*Outcomes values were wrongly not reported within the publication (blood tests values reported instead of cognitive and functional outcomes)*	*No difference in AEs*	***Overall**: Poor*<br><br>***QI**:*	[6]
Buyang Huanwu Decoction + Olanzapine	*Post-stroke dementia*	*Buyang Huanwu Decoction 3 doses + Olanzapine 2.5 – 10 mg (45)*<br><br>*Vs*<br><br>*Olanzapine 2.5 – 10 mg (45)*	*6 weeks (NP)*	*No primary outcomes*<br><br>*Outcomes*<br>*Cognitive: yes*<br>*Functional: yes*<br>*Patient-centred outcomes: yes*<br>*Instrumental: Blood test*	*In favour of the combined treatment*	*The incidence of ARs was comparable in subjects who received BHD + olanzapine and those given olanzapine only (p > 0.05).*	***Overall**: Poor*<br><br>***QI**:*	[171]

Candesartan	*Vascular MCI*	*Candesartan 8 mg (goal BP <140/90 mmHg), subsequent dose titration: 8 - 16 - 32 mg (55)*<br><br>*vs*<br><br>*Lisinopril 10 mg (goal BP <140/90 mmHg) subsequent dose titration: 10 - 20- 40 mg (47)*	*12 months (NP)*	*<u>No primary outcomes</u>*<br><br>*<u>Outcomes</u>*<br>*Cognitive: yes*<br>*Functional: yes*<br>*Patient-centred outcomes: no*<br>*Instrumental: Cerebrovascular reactivity to CO_2 (MR metric)*	*Partially in favour of the treatment*	*No available data on safety*	***Overall**: Good*<br><br>***QI**:*<br>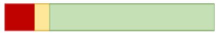	[87]
Choline alphoscerate	*Multi-infarct dementia*	*Choline alphoscerate 1000 mg (60)*<br><br>*vs*<br><br>*Citicoline 1000 mg (60)*	*90 days (NP)*	*<u>No primary outcomes</u>*<br><br>*<u>Outcomes</u>*<br>*Cognitive: yes*<br>*Functional: no*<br>*Patient-centred outcomes: no*	*Partially in favour of the treatment*	*No available data on safety*	***Overall**: Poor*<br><br>***QI**:*<br>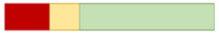	[68]
	Multi-infarct dementia	*Choline alphoscerate 1000 mg (59)*<br><br>*vs*<br><br>*Citicoline 1000 mg (58)*	*90 days (NP)*	*<u>No primary outcomes</u>*<br><br>*<u>Outcomes</u>*<br>*Cognitive: yes*<br>*Functional: no*<br>*Patient-centred outcomes: no*	*Partially in favour of the treatment*	*No available data on safety*	***Overall**: Fair*<br><br>***QI**:*<br>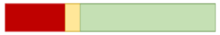	[61]
	Multi-infarct dementia	*Choline alphoscerate 1000 mg (57)*<br><br>*vs*<br><br>*Citicoline 1000 mg (56)*	*90 days (follow-up: 90 days)*	*<u>No primary outcomes</u>*<br><br>*<u>Outcomes</u>*<br>*Cognitive: yes*<br>*Functional: yes*<br>*Patient-centred outcomes: no*	*In favour of the treatment*	*No available data on safety*	***Overall**: Fair*<br><br>***QI**:*<br>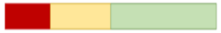	[45]
Choline alphoscerate + Nimodipine	*Subcortical Vascular dementia*	*Choline alphoscerate 1200 mg + Nimodipine 90 mg (24)*<br><br>*vs*<br><br>*Placebo + Nimodipine 90 mg (24)*	*12 months (NP)*	*<u>Primary outcomes</u>*<br>*Cognitive: MoCA*<br>*Functional: no*<br>*Patient-centred outcomes: no*<br><br>*<u>Secondary outcomes</u>*<br>*Cognitive: yes*<br>*Functional: yes*<br>*Patient-centred outcomes: yes*	*Neutral*	*8 patients referred a total of 14 symptoms compatible with an adverse reaction, of which 13 fell within those known for the drugs; none was classified as serious.*	***Overall**: Fair*<br><br>***QI**:*<br>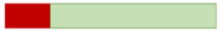	[3]
Choto-san	*Vascular dementia*	*Choto-san 7.5 g (69)*<br><br>*vs*<br><br>*Placebo (70)*	*12 weeks (NP)*	*<u>No primary outcomes</u>*<br><br>*<u>Outcomes</u>*<br>*Cognitive: yes*<br>*Functional: yes*	*Partially in favour of the treatment*	*No available data on safety*	***Overall**: Fair*<br><br>***QI**:*<br>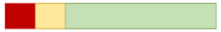	[41]

				Patient-centred outcomes: no				
**Citicoline**	*Vascular MCI*	*Citicoline 1000 mg (265)*<br><br>*vs*<br><br>*Best Medical Treatment (84)*	*9 months (NP)*	<u>*No primary outcomes*</u><br><br><u>*Outcomes*</u><br>*Cognitive: yes*<br>*Functional: yes*<br>*Patient-centred outcomes: no*	*Partially in favour of the treatment*	*No significant AEs. 5.6% occasional excitability or restlessness.*<br>*4.5 % digestive intolerance*<br>*3.6% self-limiting headaches*	***Overall**: Poor*<br><br>***QI**:*<br>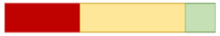	[95]
	Vascular Dementia	*Citicoline 1000 mg (15)*<br><br>*vs*<br><br>*Placebo (15)*	*12 months (NP)*	<u>*No primary outcomes*</u><br><br><u>*Outcomes*</u><br>*Cognitive: yes*<br>*Functional: no*<br>*Patient-centred outcomes: no*<br>*Instrumental: brain MRI*	*Neutral*	*No available data on safety*	***Overall**: Fair*<br><br>***QI**:*<br>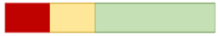	[72]
**Citicoline + Piracetam**	*Multi-infarct dementia*	*Citicoline 3 g OR Piracetam 6 g (NR)*<br><br>*vs*<br><br>*Citicoline 3 g + Piracetam 6 g (NR)*<br><br>*Total n. of participants*: 16	*30 days (NP)*	<u>*No primary outcomes*</u><br><br><u>*Outcomes*</u><br>*Cognitive: yes*<br>*Functional: no*<br>*Patient-centred outcomes: no*<br>*Instrumental: Blood tests, Sympathetic activity*	*Neutral*	*No available data on safety*	***Overall**: Fair*<br><br>***QI**:*<br>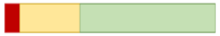	[71]
**Cytidine**	*Multi-infarct dementia*	*Cytidine 750 mg (NR)*<br><br>*vs*<br><br>*Placebo (NR)*<br><br>*Total n. of participants: 20*	*60 days (NP)*	<u>*No primary outcomes*</u><br><br><u>*Outcomes*</u><br>*Cognitive: no*<br>*Functional: no*<br>*Patient-centred outcomes: no*<br>*Instrumental: Evoked potential, EEG*	*In favour of the treatment*	*No available data on safety*	***Overall**: Fair*<br><br>***QI**:*<br>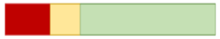	[60]
**Co-dergocrine mesylate**	*Multi-infarct dementia*	*Co-dergocrine mesylate (IV) 3 mg (17)*<br><br>*vs*<br><br>*Placebo (19)*	*1 week (follow-up: 3 weeks)*	<u>*No primary outcomes*</u><br><br><u>*Outcomes*</u><br>*Cognitive: yes*<br>*Functional: no*<br>*Patient-centred outcomes: no*	*Partially in favour of the treatment*	*AEs during IV infusions nausea (6), gastric discomfort (2) tremor, nasal congestion, flushing, hypotension and hypertension (1 each).*	***Overall**: Fair*<br><br>***QI**:*<br>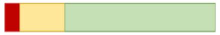	[84]

Denbufylline	*Multi-infarct dementia*	*Denbufylline 200 mg (NR)*<br><br>*vs*<br><br>*Placebo (NR)*<br><br>*Total n.of participants: 34*	*12 weeks (NP)*	*No primary outcomes*<br><br>*Outcomes*<br>*Cognitive: yes*<br>*Functional: no*<br>*Patient-centred outcomes: no*<br>*Instrumental: EEG*	*In favour of the treatment*	*No available data on safety*	***Overall**: Fair*<br><br>***QI**:*<br>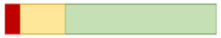	[5]
Dengzhan Shengmai	*Vascular MCI*	*Dengzhan Shengmai 6 capsules (45)*<br><br>*vs*<br><br>*Placebo (37)*	*6 months (NP)*	*Primary outcomes*<br>*Cognitive: ADAS-Cog*<br>*Functional: no*<br>*Patient-centred outcomes: no*<br><br>*Secondary outcomes*<br>*Cognitive: yes*<br>*Functional: no*<br>*Patient-centred outcomes: no*	*In favour of the treatment*	*No dropouts due to AEs*	***Overall**: Good*<br><br>***QI**:*<br>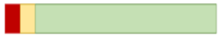	[27]
Danshen + Sanqi	*Vascular Dementia*	*Danshen + Sanqi 9 tablets (24)*<br><br>*vs*<br><br>*Placebo (23)*	*12 weeks (NP)*	*Primary outcomes*<br>*Cognitive: ADAS-Cog*<br>*Functional: no*<br>*Patient-centred outcomes: no*<br><br>*Secondary outcomes*<br>*Cognitive: yes*<br>*Functional: yes*<br>*Patient-centred outcomes: no*	*Partially in favour of the treatment*	*No available data on safety*	***Overall**: Good*<br><br>***QI**:*<br>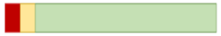	[12]
Fluoxetine	*Vascular dementia*	*Fluoxetine 20 mg (25)*<br><br>*vs*<br><br>*Placebo (25)*	*12 weeks (NP)*	*Primary outcomes*<br>*Cognitive: MMSE, CDT*<br>*Functional: no*<br>*Patient-centred outcomes: no*<br><br>*Secondary outcomes*<br>*Cognitive: yes*<br>*Functional: no*<br>*Patient-centred outcomes: no*<br>*Instrumental: BDNF*	*In favour of the treatment*	*No available data on safety*	***Overall**: Fair*<br><br>***QI**:*<br>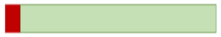	[28]
	Vascular MCI	*Fluoxetine 20 mg (25)*<br><br>*vs*	*12 weeks (NP)*	*Primary outcomes*<br>*Cognitive: ADAS-Cog, CDT.*	*In favour of the treatment*	*No available data on safety*	***Overall**: Good*<br><br>***QI**:*	[98]

		Best Medical Treatment (25)		*Functional: no*<br>*Patient-centred outcomes: no*<br><br>*<u>Secondary outcomes</u>*<br>*Cognitive: yes*<br>*Functional: no*<br>*Patient-centred outcomes: no*<br>*Instrumental: Blood tests*			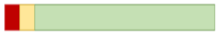	
Folic acid + Vitamin B12<br><br>****with hyperhomocysteinemia***	*Vascular MCI**	*Folic acid 5 mg + Vitamin B12 1500 mcg (60)*<br><br>*vs*<br><br>*Best Medical Treatment (60)*	*24 weeks (NP)*	*<u>No primary outcomes</u>*<br><br>*<u>Secondary outcomes</u>*<br>*Cognitive: yes*<br>*Functional: no*<br>*Patient-centred outcomes: no*<br>*Instrumental: Blood tests*	*In favour of the combined treatment*	*No available data on safety*	***Overall**: Fair*<br><br>***QI**:*<br>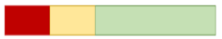	[38]
Galantamine + Cognitive training	*Post-stroke MCI*	*Galantamine 16 mg + Cognitive training (10)*<br><br>*vs*<br><br>*Placebo + Cognitive training (12)*	*12 weeks (8 weeks)*<br><br>*[total: 27 hours cognitive training]*	*<u>Primary outcomes</u>*<br>*Cognitive: no*<br>*Functional: Dementia diagnosis (ICD)*<br>*Patient-centred outcomes: no*<br><br>*<u>Secondary outcomes</u>*<br>*Cognitive: yes*<br>*Functional: yes*<br>*Patient-centred outcomes: no*	*Neutral*	*Tachycardia (1 pt, with interruption of galantamine); traumatic fall (1 pt, during wash-out); vagal syncope (1 pt, during wash-out).*	***Overall**: Good*<br><br>***QI**:*<br>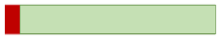	[151]
Guilingji	*Vascular MCI*	*Guilingji capsules 0.6 g + Placebo (37)*<br><br>*vs*<br><br>*Gingko extract 57.6 mg + Placebo (45)*	*24 weeks (NP)*	*<u>Primary outcomes</u>*<br>*Cognitive: MoCA*<br>*Functional: no*<br>*Patient-centred outcomes: no*<br><br>*<u>Secondary outcomes</u>*<br>*Cognitive: yes*<br>*Functional: yes*<br>*Patient-centred outcomes: no*<br>*Instrumental: Blood tests*	*In favour of the treatment*	*No reported AEs*	***Overall**: Fair*<br><br>***QI**:*<br>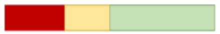	[89]

Huperzine A	*Vascular dementia*	*Huperzine A 0.2 mg (39)*<br><br>*vs*<br><br>*Vitamin C 100 mg (39)*	*12 weeks (NP)*	*<u>No primary outcomes</u>*<br><br>*<u>Outcomes</u>*<br>*Cognitive: yes*<br>*Functional: yes*<br>*Patient-centred outcomes: no*	*In favour of the treatment*	*Mild nausea (1 pt)*	***Overall**: Good*<br><br>***QI**:*<br>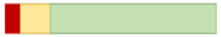	[10]
Idebenone	*Multi-infarct dementia*	*Idebenone 90 mg (56)*<br><br>*vs*<br><br>*Placebo (52)*	*120 days (NP)*	*<u>No primary outcomes</u>*<br><br>*<u>Outcomes</u>*<br>*Cognitive: yes*<br>*Functional: no*<br>*Patient-centred outcomes: no*	*In favour of the treatment*	*No available data on safety*	***Overall**: Fair*<br><br>***QI**:*<br>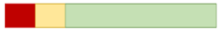	[25]
	Multi-infarct dementia	*Idebenone 90 mg (47)*<br><br>*vs*<br><br>*Placebo (50)*	*90 days (follow-up: 30 days)*	*<u>No primary outcomes</u>*<br><br>*<u>Outcomes</u>*<br>*Cognitive: yes*<br>*Functional: yes*<br>*Patient-centred outcomes: no*	*Partially in favour the treatment*	*No available data on safety*	***Overall**: Fair*<br><br>***QI**:*<br>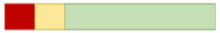	[80]
Jin Nao Ning	*Multi-infarct dementia*	*Jin Nao Ning 0.51 g (25)*<br><br>*vs*<br><br>*Duxil (Almitrine + Raubasine) 120 mg (15)*	*9 weeks (NP)*	*<u>Primary outcomes</u>*<br>*Cognitive: NPS Memory*<br>*Functional: no*<br>*Patient-centred outcomes: no*<br><br>*<u>No secondary outcomes</u>*	*Partially in favour of the treatment*	*No available data on safety*	***Overall**: Poor*<br><br>***QI**:*<br>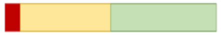	[15]
Methylphenidate	*Vascular dementia*	*Methylphenidate 10 mg (30)*<br><br>*vs*<br><br>*Galantamine 16 mg (30)*<br><br>*vs*<br><br>*Placebo (30)*	*Single drug administration (follow-up: 3.5 hours)*	*<u>No primary outcomes</u>*<br><br>*<u>Outcomes</u>*<br>*Cognitive: yes*<br>*Functional: yes*<br>*Patient-centred outcomes: no*<br>*Instrumental: EEG*	*Partially in favour of Methylphenidate*	*No available data on safety*	***Overall**: Good*<br><br>***QI**:*<br>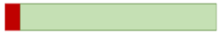	[35]
MLC601	*Vascular dementia*	*MLC601 1.2 g (41)*<br><br>*vs*<br><br>*Placebo (40)*	*24 months (NP)*	*<u>Primary outcomes</u>*<br>*Cognitive: MMSE, ADAS-Cog*<br>*Functional: no*	*In favour of the treatment*	*24.39% pts in MLC601 group transient gastrointestinal AE – 2 new strokes (1*	***Overall**: Fair*<br><br>***QI**:*<br>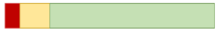	[20]

				Patient-centred outcomes: no<br><br>*<u>No secondary outcomes</u>*		*MLC601 and 1 placebo)*		
	Vascular dementia	*MLC601 15 g (232)*<br><br>*vs*<br><br>*Donepezil 5 mg (233)*<br><br>*vs*<br><br>*Placebo (55)*	*24 weeks (NP)*	*<u>Primary outcomes</u>*<br>*Cognitive: VaDAS*<br>*Functional: CIBIC-plus*<br>*Patient-centred outcomes: no*<br><br>*<u>Secondary outcomes</u>*<br>*Cognitive: yes*<br>*Functional: yes*<br>*Patient-centred outcomes: no*	*Neutral*	*Proportion of pts with AEs: 1.72% MLC601 group, 1.29% donepezil group, 1.82% placebo groups*	***Overall**: Good*<br><br>***QI**:*	[86]
MLC901	*Post-stroke MCI*	*MLC901 1.2 g (57)*<br><br>*vs*<br><br>*Placebo (46)*	*24 weeks (NP)*	*<u>Primary outcomes</u>:*<br>*Cognitive: NPS executive functions, language*<br>*Functional: no*<br>*Patient-centred outcomes: no*<br><br>*<u>Secondary outcomes:</u>*<br>*Cognitive: yes*<br>*Functional: yes*<br>*Patient-centred outcomes: no*	*Neutral*	*No significant difference in AEs and SAE*	***Overall**: Good*<br><br>***QI**:*	[94]
Modified Suanzaoren Decotion (M-SZRD)	*Post-stroke Cognitive impairment*	*M-SZRD twice daily (38)*<br><br>*vs*<br><br>*Zolpidem 5 mg (increase to 10 mg in < 65 years if not effective) (36)*	*4 weeks (NP)*	*<u>Primary outcomes</u>:*<br>*Cognitive: MoCA, PSQI*<br>*Functional: no*<br>*Patient-centred outcomes: no*<br><br>*<u>Secondary outcomes:</u>*<br>*Cognitive: yes*<br>*Functional: yes*<br>*Patient-centred outcomes: no*<br>*Instrumental: Plasma ACTH*	*Partially in favour of the treatment*	*No reports of serious adverse events*	***Overall**: Fair*<br><br>***QI**:*	[91]
Naftidrofuryl	*Vascular dementia*	*Naftidrofuryl 400 mg (113)*<br><br>*vs*	*6 months (NP)*	*<u>Primary outcomes</u>:*<br>*Cognitive: ADAS-Cog, SCAG*<br>*Functional: no*	*In favour of the treatment*	*No available data on safety*	***Overall**: Good*<br><br>***QI**:*	[53]

		Naftidrofuryl 600 mg (108)<br><br>*vs*<br><br>*Placebo (118)*		*Patient-centred outcomes: no*<br><br>*<u>Secondary outcomes:</u>*<br>*Cognitive: yes*<br>*Functional: yes*<br>*Patient-centred outcomes: no*				
	Multi-infarct dementia	*Naftidrofuryl 600 mg (13)*<br><br>*vs*<br><br>*Placebo (14*	*8 weeks (NP)*	*<u>Primary outcomes</u>:*<br>*Cognitive: Erzigkeit's Short Syndrome Test, NPS visuospatial, attention, memory, Depressiveness*<br>*Functional: no*<br>*Patient-centred outcomes: no*<br><br>*<u>Secondary outcomes:</u>*<br>*Cognitive: no*<br>*Functional: yes*<br>*Patient-centred outcomes: no*<br>*Instrumental: EEG*	*Partially in favour of the treatment*	*No available data on safety*	***Overall**: Good*<br><br>***QI**:*	[77]
**Naoxin'an**	*Vascular Cognitive Impairment*	*Naoxin'an 3 capsules (45)*<br><br>*vs*<br><br>*Ginkgo Biloba 3 capsules (35)*	*24 weeks (NP)*	*<u>Primary outcomes</u>:*<br>*Cognitive: MMSE, ADAS-Cog*<br>*Functional: no*<br>*Patient-centred outcomes: no*<br><br>*<u>Secondary outcomes:</u>*<br>*Cognitive: yes*<br>*Functional: no*<br>*Patient-centred outcomes: no*<br>*Instrumental: MRI and fMRI*	*In favour of the treatment*	*No available data on safety*	***Overall**: Fair*<br><br>***QI**:*	[88]
**Nicergoline**	*Multi-infarct dementia*	*Nicergoline 60 mg (28)*<br><br>*vs*<br><br>*Placebo (28)*	*8 weeks (NP)*	*<u>No primary outcomes</u>*<br><br>*<u>Secondary outcomes</u>*<br>*Cognitive: yes*<br>*Functional: no*<br>*Patient-centred outcomes: no*	*In favour of the treatment*	*Nicergoline: insomnia (2 pts), moderate rigor (1 pt).*<br>*Placebo: mild headache (1 pt), sweating (1 pt) and*	***Overall**: Good*<br><br>***QI**:*	[4]

				Instrumental: EEG, Evoked potentials		depressed mood (1 pt).		
	Multi-infarct dementia	Nicergoline 60 mg (70)<br><br>vs<br><br>Placebo (69)	6 months (NP)	Primary outcomes<br>Cognitive: SCAG, MMSE<br>Functional: no<br>Patient-centred outcomes: no<br><br>Secondary outcomes<br>Cognitive: yes<br>Functional: yes<br>Patient-centred outcomes: no	In favour of the treatment	No available data on safety	**Overall**: Good<br><br>**QI**:<br>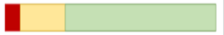	[56]
**Olanzapine**	Vascular dementia	Olanzapine 2.5 mg (titrated to 5 mg when required) (47)<br><br>vs<br><br>Bromazepam 4.5 mg (47)	6 months (NP)	No primary outcomes<br><br>Outcomes<br>Cognitive: yes<br>Functional: yes<br>Patient-centred outcomes: no	In favour of the treatment	No available data on safety	**Overall**: Poor<br><br>**QI**:<br>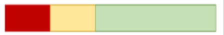	[50]
	Vascular dementia	Olanzapine 2.5 - 7.5 mg (173)<br><br>vs<br><br>Promazine 4% up to 10 drops x 3/day (60 pts) OR haloperidol 0.2% up to 10 drops x 3/day die (113)	12 months (NP)	No primary outcomes<br><br>Outcomes<br>Cognitive: yes<br>Functional: yes<br>Patient-centred outcomes: no	In favour of the treatment	Mild and transient somnolence, postural instability, and postural hypotension	**Overall**: Fair<br><br>**QI**:<br>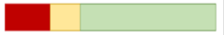	[51]
**Piracetam**	Multi-infarct dementia	Piracetam 4800 mg (65)<br><br>vs<br><br>Placebo (65)	12 weeks (NP)	No primary outcomes<br><br>Outcomes<br>Cognitive: yes<br>Functional: yes<br>Patient-centred outcomes: no	In favour of the treatment	No available data on safety	**Overall**: Good<br><br>**QI**:<br>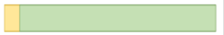	[57]
**Pyrintol (Vitamin B6)**	Multi-infarct dementia	Pyrintol 600 mg (27)<br><br>vs<br><br>Placebo (29)	12 weeks (NP)	No primary outcomes<br><br>Outcomes<br>Cognitive: yes<br>Functional: no<br>Patient-centred outcomes: no	In favour of the treatment	No available data on safety	**Overall**: Good<br><br>**QI**:<br>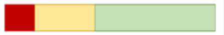	[62]

Posatirelin	*Vascular dementia*	*Posatirelin 10 mg/mL [no clear absolute dosage reported] i.m (54)*<br><br>*vs*<br><br>*Placebo (56)*	*12 weeks (follow-up: 4 weeks)*	*<u>No primary outcomes</u>*<br><br>*<u>Outcomes</u>*<br>*Cognitive: yes*<br>*Functional: no*<br>*Patient-centred outcomes: no*	*In favour of the treatment*	*5.8% treated pts: asthenia, general malaise and tremor, tachycardia, flushing, nausea and gastric pain.*	***Overall**: Good*<br><br>***QI**:*<br>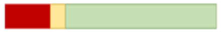	[9]
Pushen	*Vascular MCI*	*Pushen 5.4 mg (30)*<br><br>*vs*<br><br>*Ginkgo Biloba 57.6 mg (32)*	*12 weeks (NP)*	*<u>No primary outcomes</u>*<br><br>*<u>Outcomes</u>*<br>*Cognitive: yes*<br>*Functional: no*<br>*Patient-centred outcomes: no*<br>*Instrumental: Blood tests*	*Neutral*	*No available data on safety*	***Overall**: Fair*<br><br>***QI**:*<br>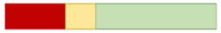	[34]
SaiLuo Tong	*Vascular dementia*	*SLT1: SaiLuo Tong 360 mg*<br>*SLT2: SaiLuo Tong 240 mg*<br>*P: placebo*<br><br>*SLT1 for 52 weeks (109)*<br><br>*vs*<br><br>*SLT2 for 52 weeks (108)*<br><br>*vs*<br><br>*P for 26 weeks + SLT1 for 26 weeks (55)*<br><br>*vs*<br><br>*P for 26 weeks + SLT2 for 26 weeks (53)*	*52 weeks (NP)*	*<u>Primary outcomes</u>:*<br>*Cognitive: VaDAS-cog, ADCS-CGIC*<br>*Functional: no*<br>*Patient-centred outcomes: no*<br><br>*<u>Secondary outcomes:</u>*<br>*Cognitive: yes*<br>*Functional: yes*<br>*Patient-centred outcomes: no*	*In favour of the treatment*	*No available data on safety*	***Overall**: Good*<br><br>***QI**:*<br>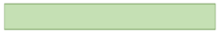	[40]
Shenlong Jiannao Tang<br>***+ Acupunture***<br>***+ Cognitive Training***	*Vascular dementia*	*Shenlong Jiannao Tang 600 ml + Cognitive training (32)*<br><br>*vs*<br><br>*Shenlong Jiannao Tang 600 ml + Acupuncture (33)*<br><br>*vs*	*3 months (NP)*<br><br>*[total: 24-36 hours cognitive training, 24-36 sessions acupuncture]*	*<u>No primary outcomes</u>*<br><br>*<u>Outcomes</u>*<br>*Cognitive: yes*<br>*Functional: no*<br>*Patient-centred outcomes: no*	*Partially in favour of the combined treatment*	*No available data on safety*	***Overall**: Poor*<br><br>***QI**:*<br>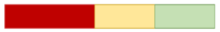	[105]

		Shenlong Jiannao Tang 600 ml + Cognitive training + Acupuncture (37)<br><br>*vs*<br><br>*Piracetam 4.9 g (32)*						
Shenmayizhi Formula + Gingko Biloba	*Vascular dementia*	*Shenmayizhi Formula 9.6 g + Gingko Biloba 3 capsules (85)*<br><br>*vs*<br><br>*Placebo + Ginkgo Biloba 3 capsules (87)*	*12 weeks (NP)*	*Primary outcomes:*<br>*Cognitive: MMSE*<br>*Functional: CM-SS*<br>*Patient-centred outcomes: no*<br>*Instrumental: ET-1, NO, vWF, NSE, BDNF.*<br><br>*No secondary outcomes:*	*In favour of the combined treatment*	*No available data on safety*	***Overall:** Good*<br><br>***QI:***<br>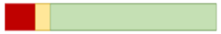	[1]
Shibing Xingnao granules + Acupuncture	*Post-stroke dementia*	*Shibing Xingnao granules 7.5 g + Acupuncture (39)*<br><br>*vs*<br><br>*Acupuncture (39)*	*4 weeks (NP)*<br><br>*[total: 24 sessions acupuncture]*	*No primary outcomes*<br><br>*Outcomes*<br>*Cognitive: yes*<br>*Functional: no*<br>*Patient-centred outcomes: no*	*In favour of the combined treatment*	*No available data on safety*	***Overall:** Poor*<br><br>***QI:***<br>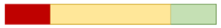	[166]
Sulfomucopolysaccharides	*Multi-infarct dementia*	*Sulfomucopolysaccharides 600 units (15)*<br><br>*vs*<br><br>*Placebo (15)*	*8 weeks (NP)*	*No primary outcomes*<br><br>*Outcomes*<br>*Cognitive: yes*<br>*Functional: no*<br>*Patient-centred outcomes: no*	*In favour of the treatment*	*No available data on safety*	***Overall:** Fair*<br><br>***QI:***<br>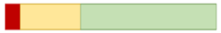	[7]
	Multi-infarct dementia	*Sulfomucopolysaccharides 500 units (15)*<br><br>*vs*<br><br>*Citicoline 1000 mg (15)*	*28 days (NP)*	*No primary outcomes*<br><br>*Outcomes*<br>*Cognitive: yes*<br>*Functional: no*<br>*Patient-centred outcomes: no*	*Partially in favour of the treatment*	*No available data on safety*	***Overall:** Fair*<br><br>***QI:***<br>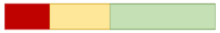	[69]
Sulodexide	*Vascular dementia*	*Sulodexide 100 mg (49)*<br><br>*vs*<br><br>*Pentoxifylline 1200 mg (44)*	*6 months (NP)*	*Primary outcomes:*<br>*Cognitive: no*<br>*Functional: no*<br>*Patient-centred outcomes: no*<br>*Instrumental: Fibrinogen*<br><br>*Secondary outcomes:*	*Partially in favour of the treatment*	*3 dropouts in both groups for AEs.*	***Overall:** Fair*<br><br>***QI:***<br>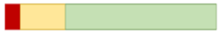	[8]

				Cognitive: yes<br>*Functional: no*<br>*Patient-centred outcomes: no*<br>*Instrumental: Blood test*				
Tandospirone citrate + Escitalopram<br><br>****Vascular depression MCI***	*Vascular MCI**	*Tandospirone 30 mg + Escitalopram 10 mg (55)*<br><br>*vs*<br><br>*Escitalopram 10 mg (55)*	*8 weeks (NP)*	*Primary outcomes:*<br>*Cognitive: NPS attention, executive functions, language, memory, CDT*<br>*Functional: no*<br>*Patient-centred outcomes: no*<br><br>*Secondary outcomes:*<br>*Cognitive: yes*<br>*Functional: no*<br>*Patient-centred outcomes: no*	*Partially in favour of the combined treatment*	*Tandospirone group: nausea (5 pts), dizziness (4 pts), somnolence (1 pt), constipation (1pt), overall AE incidence of 20.0%. Control group: nausea (4 pts), dizziness (3 pts), somnolence (1 pt), constipation (1 pt), an overall AE incidence of 17.0%. No significant difference in the overall incidence of AEs was found between the two groups (p = 0.708)*	***Overall:*** *Fair*<br><br>***QI:***<br>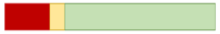	[90]
Trimetazidine + Oxiracetam	*Vascular dementia*	*Trimetazidine 60 mg + Oxiracetam 2.4 g (41)*<br><br>*vs*<br><br>*Placebo + Oxiracetam 2.4 g (41)*	*90 days (NP)*	*No primary outcomes*<br><br>*Outcomes*<br>*Cognitive: yes*<br>*Functional: yes*<br>*Patient-centred outcomes: no*	*In favour of the combined treatment*	*No significant difference in AEs*	***Overall:*** *Poor*<br><br>***QI:***<br>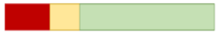	[2]
Vincamine	*Multi-infarct dementia*	*Vincamine 60 mg (NR)*<br><br>*vs*<br><br>*Placebo (NR)*<br><br>*Total n. of participants: 77*	*12 weeks (NP)*	*No primary outcomes*<br><br>*Outcomes*<br>*Cognitive: yes*<br>*Functional: no*<br>*Patient-centred outcomes: no*	*In favour of the treatment*	*No available data on safety*	***Overall:*** *Fair*<br><br>***QI:***<br>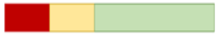	[63]
Xantinolnicotinate	*Multi-infarct dementia*	*Xantinolnicotinate 3 g (NR)*<br><br>*vs*	*12 weeks (NP)*	*Primary outcomes*<br>*Cognitive: ADCS-CGIC*<br>*Functional: no*	*In favour of the treatment*	*No available data on safety*	***Overall:*** *Good*<br><br>***QI:***<br>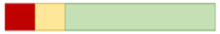	[37]

		Placebo (NR)		*Patient-centred outcomes: no*<br><br>*<u>Secondary outcomes</u>*<br>*Cognitive: yes*<br>*Functional: yes*<br>*Patient-centred outcomes: no*				
Xialong	*Post-stroke dementia*	*Xialong 8.1 g (34)*<br><br>*vs*<br><br>*Hydergine 3 mg (34)*	*3 months (treatment duration not clearly reported) (NP)*	*<u>No primary outcomes</u>*<br><br>*<u>Outcomes</u>*<br>*Cognitive: yes*<br>*Functional: yes*<br>*Patient-centred outcomes: no*	*In favour of the treatment*	*No reported AEs*	***Overall**: Poor*<br><br>***QI**:*	[99]
Yin-Xing-Tong-Zhi	*Vascular MCI*	*Yin-Xing-Tong-Zhi (dosage NR) (34)*<br><br>*vs*<br><br>*Placebo (34)*	*24 weeks (NP)*	*<u>Primary outcomes</u>:*<br>*Cognitive: ADAS-COG, MMSE,*<br>*Functional: CIBIC-plus*<br>*Patient-centred outcomes: no*<br>*Instrumental: no*<br><br>*<u>Secondary outcomes:</u>*<br>*Cognitive: no*<br>*Functional: no*<br>*Patient-centred outcomes: no*<br>*Instrumental: Blood tests*	*In favour of the treatment*	*No available data on safety*	***Overall**: Fair*<br><br>***QI**:*	[19]

Notes:
$ Follow-up time refers to the period of observation between the end of treatment and up to the end of the study (i.e. not including the timespan of treatment administration).
* Overall quality as rated according to the NIH Quality Assessment tools for controlled intervention studies is reported here. QI (Quality Index) is a graphical, colour-coded representation of the number of items on the scale rated respectively as at high-risk (red), unclear risk (yellow) or low-risk (green) of bias.

Abbreviations:
Abbreviations: ADAS-Cog, Alzheimer's Disease Assessment Scale – cognitive subscale; ADCS-CGIC, Alzheimer's Disease Cooperative Study-Clinical Global Impression of Change; BDNF, brain derived neurotrophic factor; CDT, Clock Drawing Test; CIBIC-plus, Clinician's Interview-Based Impression of Change Plus caregiver input; CM-SS, Chinese Medicine Symptom Scale; EEG, electroencephalogram; ET-1, endothelin 1; MMSE, Mini-Mental State Examination; MoCA,

Montreal Cognitive Assessment; MR, Magnetic Resonance Imaging of the brain; NO, nitric oxide; NSE, neuron specific enolase; SCAG, Sandoz Clinical Assessment Geriatric scale; TC-US, transcranial ultrasound; vWF, von-Willebrand factor; VaDAS, Vascular dementia Assessment Scale.

NP, not performed; NR, not reported.

3.1.2 *eTable 3* - Qualitative summary of study characteristics and efficacy of rehabilitative interventions

Intervention	VCI population	Treatment arms	Treatment duration (follow-up)[$]	Outcomes	Efficacy	Quality score[*]	Study
Cognitive stimulation	*Vascular*	*Reminiscence group (17)*<br><br>*vs*<br><br>*Social contact group (11)*<br><br>*vs*<br><br>*Control group (inactive treatment, 17)*	*3 months*<br>*[total: ~ 12 hours]*<br><br>*(follow-up: NP)*	***Primary outcomes***<br>***none***<br><br>*Other outcomes*<br>*Cognitive: yes*<br>*Functional: yes*<br>*Patient-centred: no*	*Neutral*	***Overall**: Fair*<br><br>***QI**:*	[143]
	Post-stroke	*Cognitive stimulation therapy group (10)*<br><br>*vs*<br><br>*Conventional rehabilitation group (10)*	*8 weeks*<br>*[total: ~ 53 hours]*<br><br>*(follow-up: NP)*	***Primary outcomes***<br>***Cognitive: MMSE***<br><br>*Secondary outcomes*<br>*Cognitive: no*<br>*Functional: yes*<br>*Patient-centred: yes*	*Neutral*	***Overall**: Good*<br><br>***QI**:*	[152]
Virtual reality	*Post-stroke*	*Experimental group (18)*<br><br>*vs*<br><br>*Control group (17)*	*6 weeks*<br>*[total: ~ 15 hours]*<br><br>*(follow-up:3 months)*	***Primary outcomes***<br>***Cognitive: attention, memory, executive function, and spatial awareness composite scores***<br><br>*Secondary outcomes*<br>*Cognitive: yes*<br>*Functional: yes*<br>*Patient-centred: no*	*Partially in favour of treatment*	***Overall**: Good*<br><br>***QI**:*	[136]
	Post-stroke	*Intervention group (15)*<br><br>*vs*<br><br>*Control group (15)*	*6 weeks*<br>*[total: ~ 9 hours]*<br><br>*(follow-up: NP)*	***Primary outcomes***<br>***none***<br><br>*Other outcomes*<br>*Cognitive: yes*<br>*Functional: yes*<br>*Patient-centred: no*	*Partially in favour of treatment*	***Overall**: Fair*<br><br>***QI**:*	[140]
Physical activity + Occupational Therapy	*Post-stroke*	*Aerobic exercise group + Occupational therapy*	*2 weeks*<br>*[total: ~ 8 hours]*	***Primary outcomes***<br>***none***	*In favour of treatment*	***Overall**: Poor*<br><br>***QI**:*	[155]

**+ Acupuncture**		*+ motor rehabilitation + Acupuncture (10)*<br><br>*Vs*<br><br>*Occupational therapy + motor rehabilitation + Acupuncture (10)*	*(follow-up: NP)*	*<u>Other outcomes</u>*<br>*Cognitive: yes*<br>*Functional: no*<br>*Patient-centred: no*		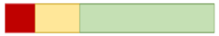	
**Motor rehabilitation**	*Post-stroke*	*Human–robotic interactive gait training (23)*<br><br>*vs*<br><br>*conventional physiotherapy (25)*	*6 weeks*<br>*[total: ~ 18 hours]*<br><br>*(follow-up: NP)*	***<u>Primary outcomes</u>***<br>***none***<br><br>*<u>Other outcomes</u>*<br>*Cognitive: yes*<br>*Functional: yes*<br>*Patient-centred: no*	*Partially in favour of treatment*	***Overall**: Poor*<br><br>***QI**:*<br>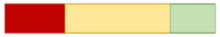	[164]
**Combined interventions**	*Vascular*	*Cognitive training group (10)*<br><br>*vs*<br><br>*Motor imagery + action observation group (10)*<br><br>*vs*<br><br>*Combined therapy group (10)*	*8 weeks*<br>*[total: ~ 26 hours]*<br><br>*(follow-up: 1 month)*	***<u>Primary outcomes</u>***<br>***Cognitive: MoCA***<br><br>*<u>Secondary outcomes</u>*<br>*Cognitive: yes*<br>*Functional: no*<br>*Patient-centred: no*<br>*Instrumental: yes (ERP)*	*In favour of treatment*	***Overall**: Fair*<br><br>***QI**:*<br>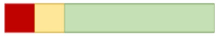	[141]
	Post-stroke	*Cognitive and motor training group (17)*<br><br>*vs*<br><br>*Cognitive training group (16)*	*4 weeks*<br>*[total: ~ 13 hours]*<br><br>*(follow-up: NP)*	***<u>Primary outcomes</u>***<br>***Cognitive: MoCA, MMSE***<br><br>*<u>Secondary outcomes</u>*<br>*Cognitive: no*<br>*Functional: no*<br>*Patient-centred: no*<br>*Instrumental: yes (ERP, fNIRS)*	*In favour of treatment*	***Overall**: Fair*<br><br>***QI**:*<br>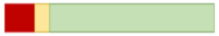	[144]
	Post-stroke	*Enriched rehabilitation group (20)*<br><br>*vs*<br><br>*Control group (20)*	*8 weeks*<br>*[total: ~ 96 hours]*<br><br>*(follow-up: NP)*	***<u>Primary outcomes</u>***<br>***none***<br><br>*<u>Other outcomes</u>*<br>*Cognitive: yes*<br>*Functional: no*<br>*Patient-centred: no*<br>*Instrumental: yes (laboratory)*	*In favour of treatment*	***Overall**: Poor*<br><br>***QI**:*<br>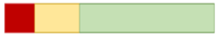	[145]

Personalised Music Playlist listening	*Post-stroke cognitive impairment*	*Personalised Music Playlist listening (18)*<br><br>*vs*<br><br>*White noise Playlist listening (18)*	*3 months*<br>[*total: ~ 90 hours*]<br><br>*(follow-up: NP)*	*<u>Primary outcomes</u>*<br>*Cognitive: MoCA*<br>*Functional: no*<br>*Patient-centred: no*<br><br>*<u>Secondary outcomes</u>*<br>*Cognitive: yes*<br>*Functional: yes*<br>*Patient-centred: yes*	*In favour of treatment*	***Overall**: Good*<br><br>***QI**:*<br>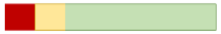	[157]

Notes:

[$] Follow-up time refers to the period of observation between the end of treatment and up to the end of the study (i.e. not including the timespan of treatment administration). Other relevant treatment specifiers (e.g., number of sessions, session duration) have been reported in square brackets.
***** Overall quality as rated according to the NIH Quality Assessment tools for controlled intervention studies is reported here. QI (Quality Index) is a graphical, colour-coded representation of the number of items on the scale rated respectively as at high-risk (red), unclear risk (yellow) or low-risk (green) of bias.

Abbreviations: *ADAS-Cog, Alzheimer's Disease Assessment Scale – cognitive subscale; ERP, event-related potential; fNIRS, functional near-infrared Spectroscopy; MMSE, Mini-Mental State Examination; MoCA, Montreal Cognitive Assessment.*

NP, not performed; NR, not reported.

3.1.3 *eTable 4* - Qualitative summary of study characteristics and efficacy of non-rehabilitative non-pharmacological interventions

Intervention	VCI population (label)	Treatment arms	Treatment duration (follow-up)[$]	Outcomes	Efficacy	Safety	Quality score[*]	Study
Acupuncture	*Vascular dementia*	*5 different types of acupuncture:*<br><br>*Conventional treatment) (10)*<br><br>*vs*<br><br>*Conventional treatment + treatment on DU20 (10)*<br><br>*vs*<br><br>*Conventional treatment + treatment on DU26 (10)*<br><br>*vs*<br><br>*Conventional treatment + treatment on HT7 (10)*<br><br>*vs*<br><br>*Conventional treatment + treatment on DU20, DU26 and Shenmen (10)*	*4 weeks (NP)*<br><br>[*total: 20 sessions*]	*<u>No primary outcomes</u>*<br><br>*<u>Outcomes</u>*<br>*Cognitive: no*<br>*Functional: yes*<br>*Patient-centred outcomes: no*<br>*Instrumental: FDG-PET*	*Different acupoints had different effects*	*No available data on safety*	***Overall**: Poor*<br><br>***QI**:*<br>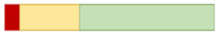	[116]
	Post-stroke dementia	*5 different types of acupuncture:*<br><br>*Conventional treatment) (10)*<br><br>*vs*	*4 weeks (NP)*<br><br>[*total: 20 sessions*]	*<u>No primary outcomes</u>*<br><br>*<u>Outcomes</u>*<br>*Cognitive: no*<br>*Functional: yes*<br>*Patient-centred outcomes: no*	*In favour of the treatment on Baihui, Shuigou and Shenmen*	*No available data on safety*	***Overall**: Poor*<br><br>***QI**:*<br>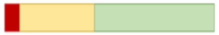	[115]

		Treatment on Baihui (DU20) (10)<br><br>*vs*<br><br>*Treatment on Shuigou (DU26) (10)*<br><br>*vs*<br><br>*Treatment on Shenmen (HT7) (10)*<br><br>*vs*<br><br>*Treatment on Baihui (DU20), Shuigou (DU26) and Shenmen (HT7) (10)*						
	Vascular dementia	*Acupuncture (22)*<br><br>*vs*<br><br>*Best medical treatment (22)*	*6 weeks (4 weeks)*<br><br>*[total: ~ 21 sessions]*	*<u>No primary outcomes</u>*<br><br>*<u>Outcomes</u>*<br>*Cognitive: no*<br>*Functional: yes*<br>*Patient-centred outcomes: yes*	*Partially in favour of the treatment*	*No available data on safety*	***Overall**: Good*<br><br>***QI***	[107]
	Vascular dementia	*Traditional acupuncture + "yi qi tiao xue, fu ben pei yuan' acupuncture (30)*<br><br>*vs*<br><br>*Traditional acupuncture + Best medical treatment (30)*	*6 weeks (NP)*<br><br>*[total: ~ 42 sessions]*	*<u>No primary outcomes</u>*<br><br>*<u>Outcomes</u>*<br>*Cognitive: no*<br>*Functional: yes*<br>*Patient-centred outcomes: no*	*In favour of the combined treatment*	*No available data on safety*	***Overall**: Good*<br><br>***QI***	[108]
	Vascular MCI	*Acupuncture (108)*<br><br>*vs*<br><br>*Citicoline 300 mg (105)*	*3 months (follow-up: 3 months)*<br><br>*[total: 24 sessions]*	*<u>Primary outcomes</u>*<br>*Cognitive: ADAS-CoG*<br>*Functional: no*<br>*Patient-centred outcomes: no*<br><br>*<u>Secondary outcomes</u>*	*Partially in favour of the treatment*	*The frequency of serious adverse events was very low in the two groups). The most commonly acupuncture-related side effects were*	***Overall**: Good*<br><br>***QI***	[110]

				Cognitive: yes<br>*Functional: yes*<br>*Patient-centred outcomes: no*		*bruising/hematoma and needle-related pain.*		
Acupuncture + Computerised Cognitive training	*Post-stroke cognitive impairment*	*Acupuncture + Computer cognitive training (simultaneously) (200)*<br><br>*vs*<br><br>*Acupuncture + Computer cognitive training (separately) (200)*<br><br>*vs*<br><br>*Acupuncture (197)*	*8 weeks (follow-up: 2 months)*<br><br>*[total: ~ 24 hours acupuncture, ~ 24 computer cognitive training]*	*Primary outcomes*<br>*Cognitive: MoCA, MMSE*<br>*Functional: no*<br>*Patient-centred outcomes: no*<br><br>*Secondary outcomes*<br>*Cognitive: yes*<br>*Functional: yes*<br>*Patient-centred outcomes: no*	*In favour of the combined simultaneous treatment*	*No available data on safety*	***Overall**: Good*<br><br>***QI***<br>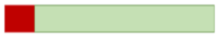	[130]
Acupuncture + Cognitive training	*Post-stroke cognitive impairment*	*Acupuncture "four seas theory" + Cognitive training (35)*<br><br>*vs*<br><br>*Cognitive training (35)*	*8 weeks (NP)*<br><br>*[total: 24 acupuncture sessions, NR cognitive training]*	*No primary outcomes*<br><br>*Outcomes*<br>*Cognitive: yes*<br>*Functional: yes*<br>*Patient-centred outcomes: no*<br>*Instrumental: Evoked potentials*	*In favour of the treatment*	*No available data on safety*	***Overall**: Fair*<br><br>***QI**:*<br>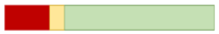	[120]
	Vascular dementia	*Acupuncture + Cognitive training (22)*<br><br>*vs*<br><br>*Cognitive training (22)*	*6 weeks (NP)*<br><br>*[total: ~ 21 acupuncture sessions, NR cognitive training]*	*No primary outcomes*<br><br>*Outcomes*<br>*Cognitive: no*<br>*Functional: yes*<br>*Patient-centred outcomes: no*	*Neutral*	*No available data on safety*	***Overall**: Poor*<br><br>***QI**:*<br>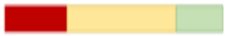	[106]
Acupuncture + Cognitive training + Motor training	Post-stroke MCI	*Acupuncture + Cognitive training + Motor training (35)*<br><br>*vs*<br><br>*Sham acupuncture + Cognitive training*	*12 weeks (NP)*<br><br>*[total: 48 acupuncture sessions, ~ 36 hours cognitive training, ~ 72 hours motor training]*	*No primary outcomes*<br><br>*Outcomes*<br>*Cognitive: yes*<br>*Functional: yes*<br>*Patient-centred outcomes: no*	*In favour of the combined treatment*	*No available data on safety*	***Overall**: Good*<br><br>***QI***<br>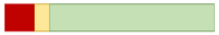	[128]

		+ Motor training (35)		*Instrumental: Blood works*				
Acupuncture + Donepezil	*Vascular dementia*	*Acupuncture + Donepezil 5 mg (84)*<br><br>*vs*<br><br>*Donepezil 5 mg (84)*	*56 days (NP)*<br><br>*[total: 56 sessions]*	*<u>No primary outcomes</u>*<br><br>*<u>Outcomes</u>*<br>*Cognitive: yes*<br>*Functional: no*<br>*Patient-centred outcomes: no*<br>*Instrumental: Evoked potentials*	*In favour of the treatment*	*No available data on safety*	***Overall**: Poor*<br><br>***QI**:*	[103]
Acupuncture + Intermittent Theta-Burst Stimulation (iTBS)	*Post stroke MCI*	*Acupuncture + iTBS (24)*<br><br>*vs*<br><br>*Acupuncture (24)*<br><br>*vs*<br><br>*iTBS, left DLPFC (24)*	*4 weeks (NP)*<br><br>*[total: ~ 12 hours acupuncture, 24 sessions iTBS]*	*<u>Primary outcomes</u>*<br>*Cognitive: MoCA*<br>*Functional: no*<br>*Patient-centred outcomes: no*<br><br>*<u>Secondary outcomes</u>*<br>*Cognitive: yes*<br>*Functional: no*<br>*Patient-centred outcomes: no*<br>*Instrumental: fNIRS*	*Partially in favour of the combined treatment*	*No available data on safety*	***Overall**: Fair*<br><br>***QI**:*	[146]
Acupuncture + Nimodipine	*Post-stroke MCI*	*Acupuncture 30 minutes 6 days/week + Nimodipine 90 mg (40)*<br><br>*vs*<br><br>*Acupuncture 30 minutes 6 days/week*<br><br>*vs*<br><br>*Nimodipine 90 mg (40)*	*3 months (follow-up: 3 months)*<br><br>*[total: ~ 72 acupuncture sessions]*	*<u>Primary outcomes</u>*<br>*Cognitive: MoCA*<br>*Functional: no*<br>*Patient-centred outcomes: no*<br><br>*<u>No secondary outcomes</u>*	*In favour of the combined treatment*	*No adverse event was reported during the study.*	***Overall**: Good*<br><br>***QI***	[101]
Acupuncture + Oxiracetam	*Post-stroke MCI*	*Acupuncture 1 every 2 days + Oxiracetam 2400 mg (32)*<br><br>*vs*<br><br>*Oxiracetam 2400 mg (31)*	*1 months (NR)*<br><br>*[total: ~ 15 sessions]*	*<u>Primary outcomes</u>*<br>*Cognitive: MoCA*<br>*Functional: no*<br>*Patient-centred outcomes: no*<br><br>*<u>Secondary outcomes</u>*<br>*Cognitive: yes*	*In favour of the combined treatment*	*No available data on safety*	***Overall**: Fair*<br><br>***QI**:*	[104]

				Functional: no<br>*Patient-centred outcomes: no*<br>*Instrumental: Blood works*				
Acupuncture (Xingnao Kaiqiao method) + repetitive Transcranial Magnetic Stimulation (rTMS)	*Post-stroke CI*	*rTMS pre-frontal lobe + Acupuncture (Xingnao Kaiqiao method) (90)*<br><br>*vs*<br><br>*rTMS pre-frontal lobe (102)*	*4 weeks (NP)*<br><br>*[total: 20 sessions acupuncture, 20 sessions rTMS]*	*No primary outcomes*<br><br>*Outcomes*<br>*Cognitive: yes*<br>*Functional: no*<br>*Patient-centred outcomes: no*<br>*Instrumental: Evoked potentials, Blood tests*	*In favour of the combined treatment*	*Transient mild headache (1 pt, control group)*	***Overall**: Poor*<br><br>***QI**:*	[169]
Electroacupuncture	*Vascular MCI*	*Electroacupuncture (60)*<br><br>*vs*<br><br>*Sham acupuncture (60)*	*32 weeks (24 weeks)*<br><br>*[total: 24 sessions]*	*Primary outcomes*<br>*Cognitive: MoCA*<br>*Functional: no*<br>*Patient-centred outcomes: no*<br><br>*Secondary outcomes*<br>*Cognitive: yes*<br>*Functional: yes*<br>*Patient-centred outcomes: no*	*In favour of the treatment*	*Not significant difference in the proportion of participants with AEs. No severe AEs.*	***Overall**: Good*<br><br>***QI***	[119]
	Vascular MCI	*Electroacupuncture (70)*<br><br>*vs*<br><br>*Best medical treatment (70)*	*8 weeks (1 week)*<br><br>*[total: ~ 20 hours]*	*Primary outcomes*<br>*Cognitive: MoCA*<br>*Functional: no*<br>*Patient-centred outcomes: no*<br><br>*Secondary outcomes*<br>*Cognitive: yes*<br>*Functional: no*<br>*Patient-centred outcomes: no*<br>*Instrumental: fMRI*	*Partially in favour of the treatment*	*No available data on safety*	***Overall**: Fair*<br><br>***QI**:*	[153]
Electroacupuncture + Cognitive training	*Post-stroke cognitive impairment*	*Electroacupuncture + Cognitive training (17)*<br><br>*vs*<br><br>*Cognitive training (17)*	*12 weeks (NP)*<br><br>*[total: ~ 30 hours electroacupuncture, ~ 20 hours cognitive training]*	*Primary outcomes*<br>*Cognitive: MoCA, Digit Span Test, Auditory Verbal Learning Test,*	*In favour of the combined treatment.*	*No adverse reactions occurred during this study.*	***Overall**: Fair*<br><br>***QI**:*	[162]

				Aphasia Screening Scale<br>*Functional: no*<br>*Patient-centred outcomes: no*<br><br>*<u>Secondary outcomes</u>*<br>*Cognitive: no*<br>*Functional: no*<br>*Patient-centred outcomes: no*<br>*Instrumental: DTI measures*				
Electroacupuncture + Nimodipine	*Vascular dementia*	*Electroacupuncture + Nimodipine 60 mg (26)*<br><br>*Vs*<br><br>*Electroacupuncture (23)*<br><br>*vs*<br><br>*Nimodipine 60 mg (24)*	*6 weeks (NP)*<br><br>*[total: 30 sessions]*	*<u>No primary outcomes</u>*<br><br>*<u>Outcomes</u>*<br>*Cognitive: yes*<br>*Functional: no*<br>*Patient-centred outcomes: no*<br>*Instrumental: Evoked potentials*	*Neutral*	*No adverse reaction reported, no incidents during acupuncture treatment. Fever (1 pt).*	***Overall**: Poor*<br><br>***QI**:*<br>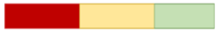	[100]
Heparin-induced extracorporeal LDL/fibrinogen precipitation (HELP) + Pentoxifylline	*Multi-infarct dementia*	*HELP + Pentoxifylline 1200 mg (141)*<br><br>*vs*<br><br>*Sham HELP + Pentoxifylline 1200 mg (75)*	*11 days (NP)*<br><br>*[total: 2 sessions]*	*<u>No primary outcomes</u>*<br><br>*<u>Outcomes</u>*<br>*Cognitive: yes*<br>*Functional: yes*<br>*Patient-centred outcomes: no*<br>*Instrumental: Blood works*	*In favour of the combined treatment*	*No available data on safety*	***Overall**: Fair*<br><br>***QI**:*<br>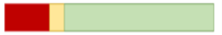	[102]
Hyperbaric Oxygen Therapy + Donepezil	*Vascular dementia*	*Hyperbaric Oxygen, 60 min session, 5 days/week + Donepezil 5 mg (79)*<br><br>*vs*<br><br>*Donepezil 5 mg (79)*	*12 weeks (NP)*<br><br>*[total: 60 sessions]*	*<u>No primary outcomes</u>*<br><br>*<u>Outcomes</u>*<br>*Cognitive: yes*<br>*Functional: no*<br>*Patient-centred outcomes: no*<br>*Instrumental: Blood works*	*In favour of the combined treatment*	*No available data on safety*	***Overall**: Fair*<br><br>***QI**:*<br>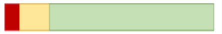	[111]

Intermittent Theta-Burst Stimulation (iTBS)<br>+ Computerized cognitive training	*Post-stroke cognitive impairment*	*iTBs, DLPC + Computerized cognitive training (19)<br><br>vs<br><br>Computerized cognitive training (18)*	*6 weeks (NP)<br><br>[total: 30 sessions iTBS, 30 sessions computerized cognitive training]*	*<u>No primary outcomes</u><br><br><u>Outcomes</u><br>Cognitive: yes<br>Functional: yes<br>Patient-centred outcomes: no<br>Instrumental: TC-US, fNIRS*	*In favour of the combined treatment*	*Temporary headaches (1 pt)*	***Overall**: Poor<br><br>**QI**:*<br>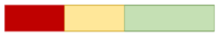	[131]
Intermittent Theta-Burst Stimulation (iTBS) +<br>Cognitive training	*Post-stroke cognitive impairment*	*iTBS, left DLPFC+ Cognitive training (25)<br><br>vs<br><br>Sham iTBS + Cognitive training (25)*	*4 weeks (NP)<br><br>[total: 20 sessions iTBS, ~ 10 hours cognitive training]*	*<u>No primary outcomes</u><br><br><u>Outcomes</u><br>Cognitive: yes<br>Functional: yes<br>Patient-centred outcomes: no*	*In favour of the combined treatment*	*No available data on safety*	***Overall**: Poor<br><br>**QI**:*<br>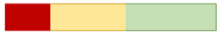	[170]
	Post-stroke cognitive impairment	*iTBS, left DLPFC+ Cognitive training (19)<br><br>Vs<br><br>Cognitive training (19)*	*6 weeks (NP)<br><br>[total: 30 sessions iTBS, ~ 15 hours cognitive training*	*<u>Primary outcomes</u><br>Cognitive: no<br>Functional: no<br>Patient-centred outcomes: no<br>Instrumental: fNIRS<br><br><u>Secondary outcomes</u><br>Cognitive: yes<br>Functional: yes<br>Patient-centred outcomes: no*	*In favour of the combined treatment*	*No available data on safety*	***Overall**: Good<br><br>**QI**:*<br>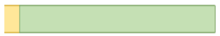	[160]
Intermittent Theta-Burst Stimulation (iTBS) +<br>Computerized Cognitive training	*Post-stroke cognitive impairment*	*Intermittent Theta-Burst Stimulation (iTBS) high-dose (3600 pulses/day) + Computerized Cognitive training (14)<br><br>Vs<br><br>Intermittent Theta-Burst Stimulation (iTBS) low-dose (1200 pulses/day) + Computerized Cognitive training (13)<br><br>Vs<br><br>Sham +*	*3 weeks (NP)<br><br>[total: 15 sessions iTBS, ~ 7.5 hours computerized cognitive training]*	*<u>Primary outcomes</u><br>Cognitive: MoCA<br>Functional: no<br>Patient-centred outcomes: no<br><br><u>Secondary outcomes</u><br>Cognitive: yes<br>Functional: no<br>Patient-centred outcomes: no*	*In favour of the first combined treatment*	*No serious adverse events reported in any group. Scalp pain and drowsiness were the most frequent mild adverse events.*	***Overall**: Good<br><br>**QI**:*<br>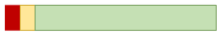	[167]

		Computerized Cognitive training (14)						
Intermittent Theta-Burst Stimulation (iTBS) OR Transcranial Direct Current Stimulation (tDCS) + Computerized cognitive training	*Post-stroke cognitive impairment*	*iTBS, DLPFC + Computerized cognitive training (21)*<br><br>*vs*<br><br>*tDCS, DLPFC + Computerized cognitive training (9)*<br><br>*vs*<br><br>*Computerized cognitive training (20)*	*6 weeks (NP)*<br><br>[*total: 30 sessions iTBS, 30 sessions tDCS, ~ 15 hours cognitive training*]	*Primary outcomes*<br>*Cognitive: LOTCA*<br>*Functional: no*<br>*Patient-centred outcomes: no*<br><br>*Secondary outcomes*<br>*Cognitive: no*<br>*Functional: yes*<br>*Patient-centred outcomes: no*<br>*Instrumental: fNIRS*	*Partially in favour, combined treatments equally effective*	*No obvious adverse reactions during the experiment*	***Overall**: Fair*<br><br>***QI**:*<br>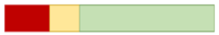	[121]
Light therapy	*Vascular dementia*	*Light therapy, 5000-3000 lux (7)*<br><br>*vs*<br><br>*Light therapy, 100 lux (5)*	*10 days (NP)*<br><br>[*total: ~ 20 hours*]	*Primary outcomes*<br>*Cognitive: MMSE*<br>*Functional: no*<br>*Patient-centred outcomes: no*<br><br>*No secondary outcomes*	*Neutral*	*No available data on safety*	***Overall**: Fair*<br><br>***QI**:*<br>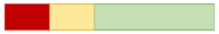	[118]
	Vascular dementia	*Bright therapy, 5000-8000 lux (12)*<br><br>*vs*<br><br>*Dim therapy, 300 lux (12)*	*2 weeks (NP)*<br><br>[*total: ~ 24 hours*]	*Primary outcomes*<br>*Cognitive: no*<br>*Functional: Absolute Nighttime Rest activity*<br>*Patient-centred outcomes: no*<br><br>*Secondary outcomes*<br>*Cognitive: no*<br>*Functional: yes*<br>*Patient-centred outcomes: no*	*In favour of the treatment*	*No available data on safety*	***Overall**: Poor*<br><br>***QI**:*<br>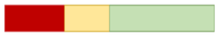	[117]
Repetitive transcranial magnetic stimulation (rTMS) + Computerized Cognitive training	*Post-stroke MCI*	*rTMS, left DLPFC + Computerized Cognitive training (16)*<br><br>*vs*<br><br>*Sham rTMS, left DLPFC +*	*4 weeks (NP)*<br><br>[*total: ~ 6.6 hours rTMS, ~ 10 hours cognitive training*]	*Primary outcomes*<br>*Cognitive: MoCA*<br>*Functional: no*<br>*Patient-centred outcomes: no*<br><br>*Secondary outcomes*<br>*Cognitive: yes*<br>*Functional: no*	*In favour of the combined treatment*	*No available data on safety*	***Overall**: Fair*<br><br>***QI**:*<br>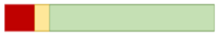	[109]

		Computerized Cognitive training (18)		*Patient-centred outcomes: no*<br>*Instrumental: fMRI*				
Repetitive transcranial magnetic stimulation (rTMS) + Cognitive training	*Post-stroke cognitive impairment*	*rTMS, left DLPFC + Cognitive training (15)*<br><br>*vs*<br><br>*Sham rTMS, left DPLFC*<br>*+ Cognitive training (15)*	*3 weeks (NP)*<br><br>*[total: 15 rTMS sessions, ~ 7.5 hours cognitive training]*	*No primary outcomes*<br><br>*Outcomes*<br>*Cognitive: yes*<br>*Functional: no*<br>*Patient-centred outcomes: no*<br>*Instrumental: fMRI*	*In favour of the combined treatment*	*rTMS: several transient dizziness, headache.*<br>*Control: dizziness (2 pts)*	***Overall**: Good*<br><br>***QI***<br>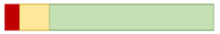	[114]
Repetitive transcranial magnetic stimulation (rTMS) + Cognitive training + Conventional motor training<br><br>****with low thyroid hormone levels***	*Post-stroke MCI**	*rTMS, DLPFC*<br>*+ Cognitive training*<br>*+ Motor training (16)*<br><br>*vs*<br><br>*Sham rTMS, DLPFC + Cognitive training*<br>*+ Motor training (15)*	*4 weeks (4 weeks)*<br><br>*[total: ~ 6.7 hours rTMS, ~ 10 hours cognitive training, ~ 13 hours motor training]*	*No primary outcomes*<br><br>*Outcomes*<br>*Cognitive: yes*<br>*Functional: no*<br>*Patient-centred outcomes: no*<br>*Instrumental: Blood tests*	*Partially in favour of the treatment*	*No adverse effects reported*	***Overall**: Fair*<br><br>***QI:***<br>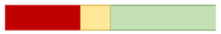	[170]
Repetitive transcranial magnetic stimulation (rTMS) + Donepezil	*Subcortical vascular cognitive impairment*	*rTMS, left DLFPC*<br>*+ Donepezil 10 mg (58)*<br><br>*Vs*<br>*Donepezil 10 mg (57)*	*4-6 weeks (4 weeks)*<br><br>*[total: 28-42 sessions rTMS]*	*Primary outcomes*<br>*Cognitive: MMSE, MoCA*<br>*Functional: no*<br>*Patient-centred outcomes: no*<br><br>*No secondary outcomes*	*In favour of the combined therapy*	*No statistically significant difference in the incidence of adverse events in the two groups.*	***Overall**: Fair*<br><br>***QI:***<br>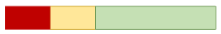	[150]
gooTranscranial direct current stimulation (tDCS) + Sertraline + Acupuncture + Cognitive training + Motor training	*Post-stroke cognitive impairment**	*tDCS, left DLPFC*<br>*+ Sertraline 50 mg*<br>*+ Acupuncture*<br>*+ Cognitive training*<br>*+ Motor training (15)*<br><br>*vs*<br><br>*Sham tDCS, left DLPFC*<br>*+ Sertraline 50 mg*<br>*+ Acupuncture*	*4 weeks (NP)*<br><br>*[total: ~ 6.6 hours tDCS, 20 sessions acupuncture, ~20 sessions cognitive training, ~20 sessions motor training]*	*No primary outcomes*<br><br>*Outcomes*<br>*Cognitive: yes*<br>*Functional: no*<br>*Patient-centred outcomes: no*<br>*Instrumental. fMRI, evoked potentials*	*In favour of the combined therapy*	*No available data on safety*	***Overall**: Fair*<br><br>***QI***<br>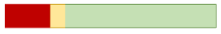	[154]

***** and depression**		+ Cognitive training<br>+ Motor training<br>(15)						
Transcranial direct current stimulation (tDCS) + Computerized cognitive training	*Post-stroke cognitive impairment*	*Home-based tDCS, left DLFPC + Computerized cognitive training (12)*<br><br>*vs*<br><br>*Sham tDCS + Computerized cognitive training (14)*	*4 weeks (NP)*<br><br>*[total: ~ 10 hours tDCS, ~ 10 hours computerized cognitive training]*	*<u>Primary outcomes</u>*<br>*Cognitive: MoCA, Dementia rating scale - attentive functions*<br>*Functional: no*<br>*Patient-centred outcomes: no*<br><br>*<u>Secondary outcomes</u>*<br>*Cognitive: yes*<br>*Functional: no*<br>*Patient-centred outcomes: no*	*Partially in favour of the combined treatment*	*No serious adverse effects reported*	***Overall**: Good*<br><br>***QI***<br>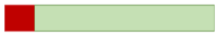	[132]
Transcranial direct current stimulation (tDCS) + Computerized cognitive training	*Post-stroke cognitive impairment*	*tDCS, left DLPFC + Computerized cognitive training (simultaneous) (18)*<br><br>*vs*<br><br>*Computerized cognitive training (18)*<br><br>*vs*<br><br>*tDCS, left DLPFC (18)*<br><br>*vs*<br><br>*Cognitive training (18)*	*3 weeks (NP)*<br><br>*[total: ~5 hours tDCS, ~5 hours computerized cognitive training; ~5 hours cognitive training]*	*<u>No primary outcomes</u>*<br><br>*<u>Outcomes</u>*<br>*Cognitive: yes*<br>*Functional: yes*<br>*Patient-centred outcomes: no*<br>*Instrumental. TC-US*	*In favour of the combined therapy*	*One subject experienced skin redness after the first tDCS treatment.*	***Overall**: Good*<br><br>***QI***<br>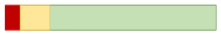	[158]
Transcranial direct current stimulation (tDCS) + Motor training + Cognitive training	*Post-stroke cognitive impairment*	*tDCS, DLFPFC (30)*<br><br>*vs*<br><br>*Motor training + Cognitive training (30)*<br><br>*vs*<br><br>*tDCS + Motor training*	*4 weeks (NP)*<br><br>*[total: ~6.7 hours tDCS, ~6.7 hours motor training, ~ 6.7 hours cognitive training]*	*<u>No primary outcomes</u>*<br><br>*<u>Outcomes</u>*<br>*Cognitive: yes*<br>*Functional: no*<br>*Patient-centred outcomes: no*	*In favour of the combined therapy*	*2 participants in the tDCS group experienced mild adverse reactions*	***Overall**: Fair*<br><br>***QI:***<br>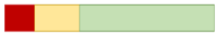	[123]

		+ Cognitive training (tDCS simultaneously with cognitive rehabilitation) (30)						
Transcranial ultrasound stimulation (TUS) + Cognitive training	*Post-stroke MCI*	*TUS + Cognitive training (30)*<br><br>*vs*<br><br>*Sham TUS + Cognitive training (30)*	*6 weeks (NP)*<br><br>*[total: ~10 hours TUS, cognitive training NR]*	*<u>No primary outcomes</u>*<br><br>*<u>Outcomes</u>*<br>*Cognitive: yes*<br>*Functional: yes*<br>*Patient-centred outcomes: no*<br>*Instrumental: Evoked potential, Blood works*	*In favour of the combined therapy*	*No available data on safety*	***Overall**: Poor*<br><br>***QI**:*<br>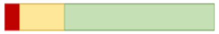	[129]

Notes:
$ Follow-up time refers to the period of observation between the end of treatment and up to the end of the study (i.e. not including the timespan of treatment administration). Other relevant treatment specifiers (e.g., number of sessions, session duration) have been reported in square brackets.
* Overall quality as rated according to the NIH Quality Assessment tools for controlled intervention studies is reported here. QI (Quality Index) is a graphical, colour-coded representation of the number of items on the scale rated respectively as at high-risk (red), unclear risk (yellow) or low-risk (green) of bias.

Abbreviations: *ADAS-Cog, Alzheimer's Disease Assessment Scale – cognitive subscale; CIBIC-plus, Clinician's Interview-Based Impression of Change Plus caregiver input; DTI, Diffusion Tensor Imaging; EEG, electroencephalogram; FDG-PET; fluorodeoxyglucose positron emission tomography; fNIRS, functional near-infrared spectroscopy; LOTCA, Lowenstein Occupational Therapy Cognitive Assessment; MMSE, Mini-Mental State Examination; MoCA, Montreal Cognitive Assessment; fMRI, functional magnetic resonance imaging of the brain; fNIRS, nearinfrared spectroscopy; NIHSS, National Institute of Health Stroke Scale; NPS, neuropsychological tests; TC-US, transcranial ultrasound.*
NP, not performed; NR, not reported.

3.1.4 *eTable 5* – Qualitative summary of study characteristics and efficacy of other interventions

Intervention	VCI population	Treatment arms	Treatment duration (follow-up)$	Outcomes$	Efficacy	Safety	Quality score*	Study
Stellate ganglion block	*Subcortical Vascular Cognitive Impairment*	*Stellate ganglion block 1 session/day (42)*<br><br>*vs*<br><br>*Best medical treatment (40)*	*20 days (NP)*<br><br>*[total: 20 sessions ganglion block]*	*<u>Primary outcomes</u>:*<br>*Other: Primary Aspiration Scale*<br>*Cognitive: no*<br>*Functional: no*<br>*Patient-centred outcomes: no*<br><br>*<u>Outcomes</u>*<br>*Cognitive: yes*<br>*Functional: yes*<br>*Patient-centred outcomes: no*	*In favour of the treatment*	*No severe adverse events were reported.*	***Overall**: Good*<br><br>***QI**:*<br>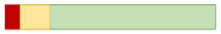	[147]

Notes:
$ Follow-up time refers to the period of observation between the end of treatment and up to the end of the study (i.e. not including the timespan of treatment administration). Other relevant treatment specifiers (e.g., number of sessions, session duration) have been reported in square brackets.
* Overall quality as rated according to the NIH Quality Assessment tools for controlled intervention studies is reported here. QI (Quality Index) is a graphical, colour-coded representation of the number of items on the scale rated respectively as at high-risk (red), unclear risk (yellow) or low-risk (green) of bias.

3.2 Interventions by VCI label

3.2.1 *eTable* 6: Interventions, not included in meta-analyses, evaluated in post-stroke cognitive impairment (including acute/subacute stroke and multi-infarct dementia)

Pharmacological interventions

Intervention	VCI population	Treatment arms	Treatment duration (follow-up)$^{\$}$	Outcomes	Efficacy	Safety profile	Quality score*	Study
Acetylsalicylic acid	*Multi-infarct dementia*	*ASA 325 mg (37)*<br><br>*vs*<br><br>*Best medical treatment (33)*	*3 years (NP)*	*No primary outcomes*<br><br>*Outcomes*<br>*Cognitive: yes*<br>*Functional: no*<br>*Patient-centred outcomes: no*<br>*Instrumental: Regional cerebral blood flow*	*In favour of the treatment*	*No available data on safety*	***Overall**: Fair*<br><br>***QI**:*<br>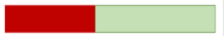	[55]
Actovegin	*Post-stroke MCI*	*Actovegin 2000 mg ev daily for ≤20 infusions followed by 1200 mg os daily (248)*<br><br>*vs*<br><br>*Placebo (255)*	*6 months (6 months)*	*Primary outcomes*<br>*Cognitive: ADAS-Cog+*<br>*Functional: no*<br>*Patient-centred outcomes: no*<br><br>*Secondary outcomes*<br>*Cognitive: yes*<br>*Functional: yes*<br>*Patient-centred outcomes: yes*	*In favour of the treatment*	*No available data on safety*	***Overall**: Good*<br><br>***QI**:*<br>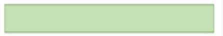	[76]
Brovincamine/Vincamine	*Multi-infarct dementia*	*Crossover design: Brovincamine 80 mg g (10)*<br><br>*vs*<br><br>*Vincamine 80 mg (10)*<br><br>*vs*<br><br>*Placebo (10)*	*Crossover: 2 weeks + 2 weeks + 2 weeks (NP)*	*No primary outcomes*<br><br>*Outcomes*<br>*Cognitive: yes*<br>*Functional: no*<br>*Patient-centred outcomes: no*<br>*Instrumental: Regional cerebral blood flow*	*Partially in favour of both treatments*	*No available data on safety*	***Overall**: Fair*<br><br>***QI**:*<br>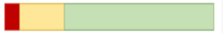	[74]
Butylphtalyde	*Post-stroke Cognitive Impairment*	*Butylphtalyde 600 mg (43)*<br><br>*vs*	*8 weeks (NP)*	*No primary outcomes*<br><br>*Outcomes*<br>*Cognitive: yes*	*In favour of the treatment*	*No available data on safety*	***Overall**: Poor*<br><br>***QI**:*	[173]

		Placebo (39)		*Functional: yes*<br>*Patient-centred outcomes: no*<br>*Instrumental: MRI*			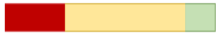	
Buyang Huanwu Decoction + Olanzapine	*Post-stroke dementia*	*Buyang Huanwu Decoction 3 doses + Olanzapine 2.5 – 10 mg (45)*<br><br>*Vs*<br><br>*Olanzapine 2.5 – 10 mg (45)*	*6 weeks (NP)*	*<u>No primary outcomes</u>*<br><br>*<u>Outcomes</u>*<br>*Cognitive: yes*<br>*Functional: yes*<br>*Patient-centred outcomes: yes*<br>*Instrumental: Blood test*	*In favour of the combined treatment*	*The incidence of ARs was comparable in subjects who received BHD + olanzapine and those given olanzapine only (p > 0.05).*	***Overall**: Poor*<br><br>***QI**:*<br>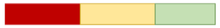	[171]
Choline alphoscerate	*Multi-infarct dementia*	*Choline alphoscerate 1000 mg (60)*<br><br>*vs*<br><br>*Citicoline 1000 mg (60)*	*90 days (NP)*	*<u>No primary outcomes</u>*<br><br>*<u>Outcomes</u>*<br>*Cognitive: yes*<br>*Functional: no*<br>*Patient-centred outcomes: no*	*Partially in favour of the treatment*	*No available data on safety*	***Overall**: Poor*<br><br>***QI**:*<br>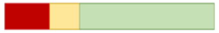	[68]
	Multi-infarct dementia	*Choline alphoscerate 1000 mg (59)*<br><br>*vs*<br><br>*Citicoline 1000 mg (58)*	*90 days (NP)*	*<u>No primary outcomes</u>*<br><br>*<u>Outcomes</u>*<br>*Cognitive: yes*<br>*Functional: no*<br>*Patient-centred outcomes: no*	*Partially in favour of the treatment*	*No available data on safety*	***Overall**: Fair*<br><br>***QI**:*<br>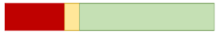	[61]
	Multi-infarct dementia	*Choline alphoscerate 1000 mg (57)*<br><br>*vs*<br><br>*Citicoline 1000 mg (56)*	*90 days (follow-up: 90 days)*	*<u>No primary outcomes</u>*<br><br>*<u>Outcomes</u>*<br>*Cognitive: yes*<br>*Functional: yes*<br>*Patient-centred outcomes: no*	*In favour of the treatment*	*No available data on safety*	***Overall**: Fair*<br><br>***QI**:*<br>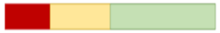	[45]

Citicoline + Piracetam	*Multi-infarct dementia*	*Citicoline 3 g OR Piracetam 6 g (NR)*<br><br>*vs*<br><br>*Citicoline 3 g + Piracetam 6 g (NR)*<br><br>*Total n. of participants:* *16*	*30 days (NP)*	*<u>No primary outcomes</u>*<br><br>*<u>Outcomes</u>*<br>*Cognitive: yes*<br>*Functional: no*<br>*Patient-centred outcomes: no*<br>*Instrumental: Blood tests, Sympathetic activity*	*Neutral*	*No available data on safety*	***Overall**: Fair*<br><br>***QI**:*<br>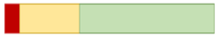	[71]
Cytidine	*Multi-infarct dementia*	*Cytidine 750 mg (NR)*<br><br>*vs*<br><br>*Placebo (NR)*<br><br>*Total n. of participants: 20*	*60 days (NP)*	*<u>No primary outcomes</u>*<br><br>*<u>Outcomes</u>*<br>*Cognitive: no*<br>*Functional: no*<br>*Patient-centred outcomes: no*<br>*Instrumental: Evoked potential, EEG*	*In favour of the treatment*	*No available data on safety*	***Overall**: Fair*<br><br>***QI**:*<br>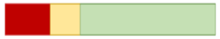	[60]
Co-dergocrine mesylate	*Multi-infarct dementia*	*Co-dergocrine mesylate (IV) 3 mg (17)*<br><br>*vs*<br><br>*Placebo (19)*	*1 week (follow-up: 3 weeks)*	*<u>No primary outcomes</u>*<br><br>*<u>Outcomes</u>*<br>*Cognitive: yes*<br>*Functional: no*<br>*Patient-centred outcomes: no*	*Partially in favour of the treatment*	*AEs during IV infusions nausea (6), gastric discomfort (2) tremor, nasal congestion, flushing, hypotension and hypertension (1 each).*	***Overall**: Fair*<br><br>***QI**:*<br>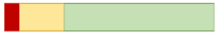	[84]
Denbufylline	*Multi-infarct dementia*	*Denbufylline 200 mg (NR)*<br><br>*vs*<br><br>*Placebo (NR)*<br><br>*Total n.of participants: 34*	*12 weeks (NP)*	*<u>No primary outcomes</u>*<br><br>*<u>Outcomes</u>*<br>*Cognitive: yes*<br>*Functional: no*<br>*Patient-centred outcomes: no*<br>*Instrumental: EEG*	*In favour of the treatment*	*No available data on safety*	***Overall**: Fair*<br><br>***QI**:*<br>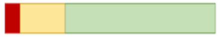	[5]
Galantamine + Cognitive training	*Post-stroke MCI*	*Galantamine 16 mg + Cognitive training (10)*<br><br>*vs*<br><br>*Placebo + Cognitive training (12)*	*12 weeks (8 weeks)*<br><br>*[total: 27 hours cognitive training]*	*<u>Primary outcomes</u>*<br>*Cognitive: no*<br>*Functional: Dementia diagnosis (ICD)*<br>*Patient-centred outcomes: no*<br><br>*<u>Secondary outcomes</u>*<br>*Cognitive: yes*<br>*Functional: yes*<br>*Patient-centred outcomes: no*	*Neutral*	*Tachycardia (1 pt, with interruption of galantamine); traumatic fall (1 pt, during wash-out); vagal syncope (1 pt, during wash-out).*	***Overall**: Good*<br><br>***QI**:*<br>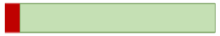	[151]

Idebenone	*Multi-infarct dementia*	*Idebenone 90 mg (56)*<br><br>*vs*<br><br>*Placebo (52)*	*120 days (NP)*	*No primary outcomes*<br><br>*Outcomes*<br>*Cognitive: yes*<br>*Functional: no*<br>*Patient-centred outcomes: no*	*In favour of the treatment*	*No available data on safety*	***Overall**: Fair*<br><br>***QI**:*<br>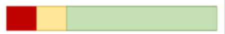	[25]
	Multi-infarct dementia	*Idebenone 90 mg (47)*<br><br>*vs*<br><br>*Placebo (50)*	*90 days (follow-up: 30 days)*	*No primary outcomes*<br><br>*Outcomes*<br>*Cognitive: yes*<br>*Functional: yes*<br>*Patient-centred outcomes: no*	*Partially in favour the treatment*	*No available data on safety*	***Overall**: Fair*<br><br>***QI**:*<br>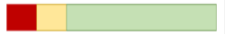	[80]
Jin Nao Ning	*Multi-infarct dementia*	*Jin Nao Ning 0.51 g (25)*<br><br>*vs*<br><br>*Duxil (Almitrine + Raubasine) 120 mg (15)*	*9 weeks (NP)*	*Primary outcomes*<br>*Cognitive: NPS Memory*<br>*Functional: no*<br>*Patient-centred outcomes: no*<br><br>*No secondary outcomes*	*Partially in favour of the treatment*	*No available data on safety*	***Overall**: Poor*<br><br>***QI**:*<br>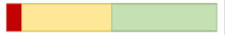	[15]
Metformine + Donepezil	*Vascular MCI*	*Metformin 500 mg + Donepezil 10 mg (48)*<br><br>*Vs*<br><br>*Acarbose 50 mg + Donepezil 10 mg (46)*	*52 weeks (NP)*	*No primary outcomes*<br><br>*Cognitive: yes*<br>*Functional: no*<br>*Patient-centred outcomes: no*<br>*Instrumental: HbA1c, carotid US*	*In favour of treatment*	*No available data on safety*	***Overall**: Good*<br><br>***QI**:*<br>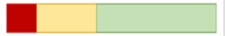	[161]
MLC901	*Post-stroke MCI*	*MLC901 1.2 g (57)*<br><br>*vs*<br><br>*Placebo (46)*	*24 weeks (NP)*	*Primary outcomes:*<br>*Cognitive: NPS executive functions, language*<br>*Functional: no*<br>*Patient-centred outcomes: no*<br><br>*Secondary outcomes:*<br>*Cognitive: yes*<br>*Functional: yes*<br>*Patient-centred outcomes: no*	*Neutral*	*No significant difference in AEs and SAE*	***Overall**: Good*<br><br>***QI**:*<br>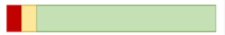	[94]

Modified Suanzaoren Decotion (M-SZRD)	*Post-stroke Cognitive impairment*	*M-SZRD twice daily (38)*<br><br>*vs*<br><br>*Zolpidem 5 mg (increase to 10 mg in < 65 years if not effective) (36)*	*4 weeks (NP)*	*Primary outcomes:*<br>*Cognitive: MoCA, PSQI*<br>*Functional: no*<br>*Patient-centred outcomes: no*<br><br>*Secondary outcomes:*<br>*Cognitive: yes*<br>*Functional: yes*<br>*Patient-centred outcomes: no*<br>*Instrumental: Plasma ACTH*	*Partially in favour of the treatment*	*No reports of serious adverse events*	***Overall**: Fair*<br><br>***QI**:*<br>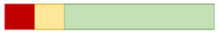	[91]
Naftidrofuryl	*Multi-infarct dementia*	*Naftidrofuryl 600 mg (13)*<br><br>*vs*<br><br>*Placebo (14*	*8 weeks (NP)*	*Primary outcomes:*<br>*Cognitive: Erzigkeit's Short Syndrome Test, NPS visuospatial, attention, memory, Depressiveness*<br>*Functional: no*<br>*Patient-centred outcomes: no*<br><br>*Secondary outcomes:*<br>*Cognitive: no*<br>*Functional: yes*<br>*Patient-centred outcomes: no*<br>*Instrumental: EEG*	*Partially in favour of the treatment*	*No available data on safety*	***Overall**: Good*<br><br>***QI**:*<br>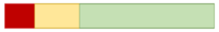	[77]
Nicergoline	*Multi-infarct dementia*	*Nicergoline 60 mg (28)*<br><br>*vs*<br><br>*Placebo (28)*	*8 weeks (NP)*	*No primary outcomes*<br><br>*Secondary outcomes*<br>*Cognitive: yes*<br>*Functional: no*<br>*Patient-centred outcomes: no*<br>*Instrumental: EEG, Evoked potentials*	*In favour of the treatment*	*Nicergoline: insomnia (2 pts), moderate rigor (1 pt). Placebo: mild headache (1 pt), sweating (1 pt) and depressed mood (1 pt).*	***Overall**: Good*<br><br>***QI**:*<br>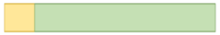	[4]
	Multi-infarct dementia	*Nicergoline 60 mg (70)*<br><br>*vs*<br><br>*Placebo (69)*	*6 months (NP)*	*Primary outcomes*<br>*Cognitive: SCAG, MMSE*<br>*Functional: no*<br>*Patient-centred outcomes: no*<br><br>*Secondary outcomes*<br>*Cognitive: yes*	*In favour of the treatment*	*No available data on safety*	***Overall**: Good*<br><br>***QI**:*<br>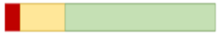	[56]

				Functional: yes<br>*Patient-centred outcomes: no*				
Piracetam	*Multi-infarct dementia*	*Piracetam 4800 mg (65)*<br><br>*vs*<br><br>*Placebo (65)*	*12 weeks (NP)*	*No primary outcomes*<br><br>*Outcomes*<br>*Cognitive: yes*<br>*Functional: yes*<br>*Patient-centred outcomes: no*	*In favour of the treatment*	*No available data on safety*	***Overall**: Good*<br><br>***QI**:*<br>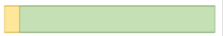	[57]
Pyrintol (Vitamin B6)	*Multi-infarct dementia*	*Pyrintol 600 mg (27)*<br><br>*vs*<br><br>*Placebo (29)*	*12 weeks (NP)*	*No primary outcomes*<br><br>*Outcomes*<br>*Cognitive: yes*<br>*Functional: no*<br>*Patient-centred outcomes: no*	*In favour of the treatment*	*No available data on safety*	***Overall**: Good*<br><br>***QI**:*<br>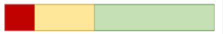	[62]
Shibing Xingnao granules + Acupuncture	*Post-stroke dementia*	*Shibing Xingnao granules 7.5 g + Acupuncture (39)*<br><br>*vs*<br><br>*Acupuncture (39)*	*4 weeks (NP)*<br><br>*[total: 24 sessions acupuncture]*	*No primary outcomes*<br><br>*Outcomes*<br>*Cognitive: yes*<br>*Functional: no*<br>*Patient-centred outcomes: no*	*In favour of the combined treatment*	*No available data on safety*	***Overall**: Poor*<br><br>***QI**:*<br>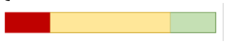	[166]
Sulfomucopolysacchar ides	*Multi-infarct dementia*	*Sulfomucopolysaccharid es 600 units (15)*<br><br>*vs*<br><br>*Placebo (15)*	*8 weeks (NP)*	*No primary outcomes*<br><br>*Outcomes*<br>*Cognitive: yes*<br>*Functional: no*<br>*Patient-centred outcomes: no*	*In favour of the treatment*	*No available data on safety*	***Overall**: Fair*<br><br>***QI**:*<br>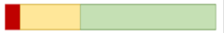	[7]
	Multi-infarct dementia	*Sulfomucopolysaccharid es 500 units (15)*<br><br>*vs*<br><br>*Citicoline 1000 mg (15)*	*28 days (NP)*	*No primary outcomes*<br><br>*Outcomes*<br>*Cognitive: yes*<br>*Functional: no*<br>*Patient-centred outcomes: no*	*Partially in favour of the treatment*	*No available data on safety*	***Overall**: Fair*<br><br>***QI**:*<br>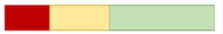	[69]

Vincamine	*Multi-infarct dementia*	*Vincamine 60 mg (NR)*<br><br>*vs*<br><br>*Placebo (NR)*<br><br>*Total n. of participants: 77*	*12 weeks (NP)*	*No primary outcomes*<br><br>*Outcomes*<br>*Cognitive: yes*<br>*Functional: no*<br>*Patient-centred outcomes: no*	*In favour of the treatment*	*No available data on safety*	***Overall**: Fair*<br><br>***QI**:*	[63]
Xantinolnicotinate	*Multi-infarct dementia*	*Xantinolnicotinate 3 g (NR)*<br><br>*vs*<br><br>*Placebo (NR)*	*12 weeks (NP)*	*Primary outcomes*<br>*Cognitive: ADCS-CGIC*<br>*Functional: no*<br>*Patient-centred outcomes: no*<br><br>*Secondary outcomes*<br>*Cognitive: yes*<br>*Functional: yes*<br>*Patient-centred outcomes: no*	*In favour of the treatment*	*No available data on safety*	***Overall**: Good*<br><br>***QI**:*	[37]
Xialong	*Post-stroke dementia*	*Xialong 8.1 g (34)*<br><br>*vs*<br><br>*Hydergine 3 mg (34)*	*3 months (treatment duration not clearly reported) (NP)*	*No primary outcomes*<br><br>*Outcomes*<br>*Cognitive: yes*<br>*Functional: yes*<br>*Patient-centred outcomes: no*	*In favour of the treatment*	*No reported AEs*	***Overall**: Poor*<br><br>***QI**:*	[99]

Notes:
$ Follow-up time refers to the period of observation between the end of treatment and up to the end of the study (i.e. not including the timespan of treatment administration).
* Overall quality as rated according to the NIH Quality Assessment tools for controlled intervention studies is reported here. QI (Quality Index) is a graphical, colour-coded representation of the number of items on the scale rated respectively as at high-risk (red), unclear risk (yellow) or low-risk (green) of bias.

Abbreviations:
Abbreviations: ADAS-Cog, Alzheimer's Disease Assessment Scale – cognitive subscale; ADCS-CGIC, Alzheimer's Disease Cooperative Study-Clinical Global Impression of Change; EEG, electroencephalogram; MMSE, Mini-Mental State Examination; MoCA, Montreal Cognitive Assessment; MRI, Magnetic Resonance Imaging of the brain; PSQI, Pittsburg Sleep Quality Index; SCAG, Sandoz Clinical Assessment Geriatric scale.

NP, not performed; NR, not reported.

Non-pharmacological non-rehabilitative interventions (application of physical devices)

Intervention	VCI population	Treatment arms	Treatment duration (follow-up)$	Outcomes	Efficacy	Safety	Quality score*	Study
Acupuncture	*Post-stroke dementia*	*5 different types of acupuncture:*<br><br>*Conventional treatment) (10)*<br><br>*vs*<br><br>*Treatment on Baihui (DU20) (10)*<br><br>*vs*<br><br>*Treatment on Shuigou (DU26) (10)*<br><br>*vs*<br><br>*Treatment on Shenmen (HT7) (10)*<br><br>*vs*<br><br>*Treatment on Baihui (DU20), Shuigou (DU26) and Shenmen (HT7) (10)*	*4 weeks (NP)*<br><br>[*total: 20 sessions*]	*No primary outcomes*<br><br>*Outcomes*<br>*Cognitive: no*<br>*Functional: yes*<br>*Patient-centred outcomes: no*	*In favour of the treatment on Baihui, Shuigou and Shenmen*	*No available data on safety*	***Overall**: Poor*<br><br>***QI**:*<br>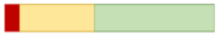	[115]
Acupunture + Computerised Cognitive training	*Post-stroke cognitive impairment*	*Acupuncture + Computer cognitive training (simultaneously) (200)*<br><br>*vs*<br><br>*Acupuncture + Computer cognitive*	*8 weeks (follow-up: 2 months)*<br><br>*[total: ~ 24 hours acupuncture, ~ 24 computer cognitive training]*	*Primary outcomes*<br>*Cognitive: MoCA, MMSE*<br>*Functional: no*<br>*Patient-centred outcomes: no*<br><br>*Secondary outcomes*<br>*Cognitive: yes*<br>*Functional: yes*	*In favour of the combined simultaneous treatment*	*No available data on safety*	***Overall**: Good*<br><br>***QI***<br>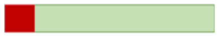	[130]

		training (separately) (200)<br><br>*vs*<br><br>*Acupuncture (197)*		*Patient-centred outcomes: no*				
Acupunture + Cognitive training	*Post-stroke cognitive impairment*	*Acupuncture "four seas theory" + Cognitive training (35)*<br><br>*vs*<br><br>*Cognitive training (35)*	*8 weeks (NP)*<br><br>*[total: 24 acupuncture sessions, NR cognitive training]*	*No primary outcomes*<br><br>*Outcomes*<br>*Cognitive: yes*<br>*Functional: yes*<br>*Patient-centred outcomes: no*<br>*Instrumental: Evoked potentials*	*In favour of the treatment*	*No available data on safety*	***Overall**: Fair*<br><br>***QI**:*<br>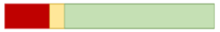	[120]
Acupunture + Cognitive training + Motor training	Post-stroke MCI	*Acupuncture + Cognitive training + Motor training (35)*<br><br>*vs*<br><br>*Sham acupuncture + Cognitive training + Motor training (35)*	*12 weeks (NP)*<br><br>*[total: 48 acupuncture sessions, ~ 36 hours cognitive training, ~ 72 hours motor training]*	*No primary outcomes*<br><br>*Outcomes*<br>*Cognitive: yes*<br>*Functional: yes*<br>*Patient-centred outcomes: no*<br>*Instrumental: Blood works*	*In favour of the combined treatment*	*No available data on safety*	***Overall**: Good*<br><br>***QI***<br>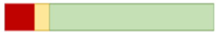	[128]
Acupuncture + Intermittent Theta-Burst Stimulation (iTBS)	*Post stroke MCI*	*Acupuncture + iTBS (24)*<br><br>*vs*<br><br>*Acupuncture (24)*<br><br>*vs*<br><br>*iTBS, left DLPFC (24)*	*4 weeks (NP)*<br><br>*[total: ~ 12 hours acupuncture, 24 sessions iTBS]*	*Primary outcomes*<br>*Cognitive: MoCA*<br>*Functional: no*<br>*Patient-centred outcomes: no*<br><br>*Secondary outcomes*<br>*Cognitive: yes*<br>*Functional: no*<br>*Patient-centred outcomes: no*<br>*Instrumental: fNIRS*	*Partially in favour of the combined treatment*	*No available data on safety*	***Overall**: Fair*<br><br>***QI**:*<br>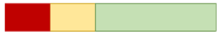	[146]
Acupuncture + Nimodipine	*Post-stroke MCI*	*Acupuncture 30 minutes 6 days/week + Nimodipine 90 mg (40)*<br><br>*vs*	*3 months (follow-up: 3 months)*<br><br>*[total: ~ 72 acupuncture sessions]*	*Primary outcomes*<br>*Cognitive: MoCA*<br>*Functional: no*<br>*Patient-centred outcomes: no*<br><br>*No secondary outcomes*	*In favour of the combined treatment*	*No adverse event was reported during the study.*	***Overall**: Good*<br><br>***QI***<br>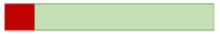	[101]

		Acupuncture 30 minutes 6 days/week<br><br>*vs*<br><br>*Nimodipine 90 mg (40)*						
Acupuncture + Oxiracetam	*Post-stroke MCI*	*Acupuncture 1 every 2 days + Oxiracetam 2400 mg (32)*<br><br>*vs*<br><br>*Oxiracetam 2400 mg (31)*	*1 months (NR)*<br><br>*[total: ~ 15 sessions]*	*<u>Primary outcomes</u>*<br>*Cognitive: MoCA*<br>*Functional: no*<br>*Patient-centred outcomes: no*<br><br>*<u>Secondary outcomes</u>*<br>*Cognitive: yes*<br>*Functional: no*<br>*Patient-centred outcomes: no*<br>*Instrumental: Blood works*	*In favour of the combined treatment*	*No available data on safety*	***Overall**: Fair*<br><br>***QI**:*	[104]
Acupuncture (Xingnao Kaiqiao method) + repetitive Transcranial Magnetic Stimulation (rTMS)	*Post-stroke CI*	*rTMS pre-frontal lobe + Acupuncture (Xingnao Kaiqiao method) (90)*<br><br>*vs*<br><br>*rTMS pre-frontal lobe (102)*	*4 weeks (NP)*<br><br>*[total: 20 sessions acupuncture, 20 sessions rTMS]*	*<u>No primary outcomes</u>*<br><br>*<u>Outcomes</u>*<br>*Cognitive: yes*<br>*Functional: no*<br>*Patient-centred outcomes: no*<br>*Instrumental: Evoked potentials, Blood tests*	*In favour of the combined treatment*	*Transient mild headache (1 pt, control group)*	***Overall**: Poor*<br><br>***QI**:*	[169]
Electroacupuncture + Cognitive training	*Post-stroke cognitive impairment*	*Electroacupuncture + Cognitive training (17)*<br><br>*vs*<br><br>*Cognitive training (17)*	*12 weeks (NP)*<br><br>*[total: ~ 30 hours electroacupuncture, ~ 20 hours cognitive training]*	*<u>Primary outcomes</u>*<br>*Cognitive: MoCA, Digit Span Test, Auditory Verbal Learning Test, Aphasia Screening Scale*<br>*Functional: no*<br>*Patient-centred outcomes: no*<br><br>*<u>Secondary outcomes</u>*<br>*Cognitive: no*<br>*Functional: no*<br>*Patient-centred outcomes: no*	*In favour of the combined treatment.*	*No adverse reactions occurred during this study.*	***Overall**: Fair*<br><br>***QI**:*	[162]

				Instrumental: DTI measures				
Heparin-induced extracorporeal LDL/fibrinogen precipitation (HELP) + Pentoxifylline	*Multi-infarct dementia*	*HELP + Pentoxifylline 1200 mg (141)*<br><br>*vs*<br><br>*Sham HELP + Pentoxifylline 1200 mg (75)*	*11 days (NP)*<br><br>*[total: 2 sessions]*	*<u>No primary outcomes</u>*<br><br>*<u>Outcomes</u>*<br>*Cognitive: yes*<br>*Functional: yes*<br>*Patient-centred outcomes: no*<br>*Instrumental: Blood works*	*In favour of the combined treatment*	*No available data on safety*	***Overall**: Fair*<br><br>***QI**:*<br>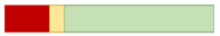	[102]
Intermittent Theta-Burst Stimulation (iTBS) + Computerized cognitive training	*Post-stroke cognitive impairment*	*iTBs, DLPC + Computerized cognitive training (19)*<br><br>*vs*<br><br>*Computerized cognitive training (18)*	*6 weeks (NP)*<br><br>*[total: 30 sessions iTBS, 30 sessions computerized cognitive training]*	*<u>No primary outcomes</u>*<br><br>*<u>Outcomes</u>*<br>*Cognitive: yes*<br>*Functional: yes*<br>*Patient-centred outcomes: no*<br>*Instrumental: TC-US, fNIRS*	*In favour of the combined treatment*	*Temporary headaches (1 pt)*	***Overall**: Poor*<br><br>***QI**:*<br>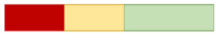	[131]
Intermittent Theta-Burst Stimulation (iTBS) + Cognitive training	*Post-stroke cognitive impairment*	*iTBS, left DLPFC+ Cognitive training (25)*<br><br>*vs*<br><br>*Sham iTBS + Cognitive training (25)*	*4 weeks (NP)*<br><br>*[total: 20 sessions iTBS, ~ 10 hours cognitive training]*	*<u>No primary outcomes</u>*<br><br>*<u>Outcomes</u>*<br>*Cognitive: yes*<br>*Functional: yes*<br>*Patient-centred outcomes: no*	*In favour of the combined treatment*	*No available data on safety*	***Overall**: Poor*<br><br>***QI**:*<br>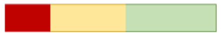	[170]
	Post-stroke cognitive impairment	*iTBS, left DLPFC+ Cognitive training (19)*<br><br>*Vs*<br><br>*Cognitive training (19)*	*6 weeks (NP)*<br><br>*[total: 30 sessions iTBS, ~ 15 hours cognitive training*	*<u>Primary outcomes</u>*<br>*Cognitive: no*<br>*Functional: no*<br>*Patient-centred outcomes: no*<br>*Instrumental: fNIRS*<br><br>*<u>Secondary outcomes</u>*<br>*Cognitive: yes*<br>*Functional: yes*<br>*Patient-centred outcomes: no*	*In favour of the combined treatment*	*No available data on safety*	***Overall**: Good*<br><br>***QI**:*<br>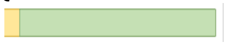	[160]
Intermittent Theta-Burst Stimulation (iTBS) + Computerized Cognitive training	*Post-stroke cognitive impairment*	*Intermittent Theta-Burst Stimulation (iTBS) high-dose (3600 pulses/day) + Computerized Cognitive training (14)*	*3 weeks (NP)*<br><br>*[total: 15 sessions iTBS, ~ 7.5 hours computerized cognitive training]*	*<u>Primary outcomes</u>*<br>*Cognitive: MoCA*<br>*Functional: no*<br>*Patient-centred outcomes: no*	*In favour of the first combined treatment*	*No serious adverse events reported in any group. Scalp pain and drowsiness*	***Overall**: Good*<br><br>***QI**:*<br>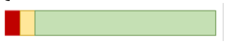	[167]

		Vs<br><br>Intermittent Theta-Burst Stimulation (iTBS) low-dose (1200 pulses/day) + Computerized Cognitive training (13)<br><br>Vs<br><br>Sham + Computerized Cognitive training (14)		Secondary outcomes<br>Cognitive: yes<br>Functional: no<br>Patient-centred outcomes: no		were the most frequent mild adverse events.		
Intermittent Theta-Burst Stimulation (iTBS) OR Transcranial Direct Current Stimulation (tDCS) + Computerized cognitive training	*Post-stroke cognitive impairment*	*iTBS, DLPFC + Computerized cognitive training (21)<br><br>vs<br><br>tDCS, DLPFC + Computerized cognitive training (9)<br><br>vs<br><br>Computerized cognitive training (20)*	*6 weeks (NP)<br><br>[total: 30 sessions iTBS, 30 sessions tDCS, ~ 15 hours cognitive training]*	*Primary outcomes<br>Cognitive: LOTCA<br>Functional: no<br>Patient-centred outcomes: no<br><br>Secondary outcomes<br>Cognitive: no<br>Functional: yes<br>Patient-centred outcomes: no<br>Instrumental: fNIRS*	*Partially in favour, combined treatments equally effective*	*No obvious adverse reactions during the experiment*	***Overall**: Fair<br><br>**QI:**<br>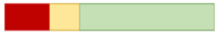*	[121]
Repetitive transcranial magnetic stimulation (rTMS) + Computerized Cognitive training	*Post-stroke MCI*	*rTMS, left DLPFC + Computerized Cognitive training (16)<br><br>vs<br><br>Sham rTMS, left DLPFC + Computerized Cognitive training (18)*	*4 weeks (NP)<br><br>[total: ~ 6.6 hours rTMS, ~ 10 hours cognitive training]*	*Primary outcomes<br>Cognitive: MoCA<br>Functional: no<br>Patient-centred outcomes: no<br><br>Secondary outcomes<br>Cognitive: yes<br>Functional: no<br>Patient-centred outcomes: no<br>Instrumental: fMRI*	*In favour of the combined treatment*	*No available data on safety*	***Overall**: Fair<br><br>**QI:**<br>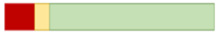*	[109]
Repetitive transcranial magnetic stimulation (rTMS) + Cognitive training	*Post-stroke cognitive impairment*	*rTMS, left DLPFC + Cognitive training (15)<br><br>vs*	*3 weeks (NP)<br><br>[total: 15 rTMS sessions, ~ 7.5 hours cognitive training]*	*No primary outcomes<br><br>Outcomes<br>Cognitive: yes<br>Functional: no*	*In favour of the combined treatment*	*rTMS: several transient dizziness, headache.*	***Overall**: Good<br><br>**QI**<br>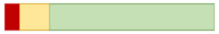*	[114]

		Sham rTMS, left DPLFC<br>*+ Cognitive training*<br>*(15)*		*Patient-centred outcomes: no*<br>*Instrumental: fMRI*		*Control: dizziness (2 pts)*		
Repetitive transcranial magnetic stimulation (rTMS)<br>***+ Cognitive training***<br>***+ Conventional motor training***<br><br>****with low thyroid hormone levels***	*Post-stroke MCI**	*rTMS, DLPFC*<br>*+ Cognitive training*<br>*+ Motor training (16)*<br><br>*vs*<br><br>*Sham rTMS, DLPFC + Cognitive training*<br>*+ Motor training (15)*	*4 weeks (4 weeks)*<br><br>*[total: ~ 6.7 hours rTMS, ~ 10 hours cognitive training, ~ 13 hours motor training]*	*<u>No primary outcomes</u>*<br><br>*<u>Outcomes</u>*<br>*Cognitive: yes*<br>*Functional: no*<br>*Patient-centred outcomes: no*<br>*Instrumental: Blood tests*	*Partially in favour of the treatment*	*No adverse effects reported*	***Overall**: Fair*<br><br>***QI:***<br>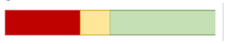	[165]
Transcranial direct current stimulation (tDCS)<br>***+ Sertraline***<br>***+ Acupuncture***<br>***+ Cognitive training***<br>***+ Motor training***<br><br>**** and depression***	*Post-stroke cognitive impairment**	*tDCS, left DLPFC*<br>*+ Sertraline 50 mg*<br>*+ Acupuncture*<br>*+ Cognitive training*<br>*+ Motor training*<br>*(15)*<br><br>*vs*<br><br>*Sham tDCS, left DLPFC*<br>*+ Sertraline 50 mg*<br>*+ Acupuncture*<br>*+ Cognitive training*<br>*+ Motor training*<br>*(15)*	*4 weeks (NP)*<br><br>*[total: ~ 6.6 hours tDCS, 20 sessions acupuncture, ~20 sessions cognitive training, ~20 sessions motor training]*	*<u>No primary outcomes</u>*<br><br>*<u>Outcomes</u>*<br>*Cognitive: yes*<br>*Functional: no*<br>*Patient-centred outcomes: no*<br>*Instrumental. fMRI, evoked potentials*	*In favour of the combined therapy*	*No available data on safety*	***Overall**: Fair*<br><br>***QI***<br>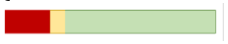	[154]
Transcranial direct current stimulation (tDCS) +<br>***Computerized cognitive training***	*Post-stroke cognitive impairment*	*Home-based tDCS, left DLFPC + Computerized cognitive training (12)*<br><br>*vs*<br><br>*Sham tDCS + Computerized cognitive training (14)*	*4 weeks (NP)*<br><br>*[total: ~ 10 hours tDCS, ~ 10 hours computerized cognitive training]*	*<u>Primary outcomes</u>*<br>*Cognitive: MoCA, Dementia rating scale - attentive functions*<br>*Functional: no*<br>*Patient-centred outcomes: no*<br><br>*<u>Secondary outcomes</u>*<br>*Cognitive: yes*<br>*Functional: no*<br>*Patient-centred outcomes: no*	*Partially in favour of the combined treatment*	*No serious adverse effects reported*	***Overall**: Good*<br><br>***QI***<br>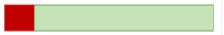	[132]

Transcranial direct current stimulation (tDCS) + Computerized cognitive training	*Post-stroke cognitive impairment*	*tDCS, left DLPFC + Computerized cognitive training (simultaneous) (18)*<br><br>*vs*<br><br>*Computerized cognitive training (18)*<br><br>*vs*<br><br>*tDCS, left DLPFC (18)*<br><br>*vs*<br><br>*Cognitive training (18)*	*3 weeks (NP)*<br><br>*[total: ~5 hours tDCS, ~5 hours computerized cognitive training; ~5 hours cognitive training]*	*<u>No primary outcomes</u>*<br><br>*<u>Outcomes</u>*<br>*Cognitive: yes*<br>*Functional: yes*<br>*Patient-centred outcomes: no*<br>*Instrumental. TC-US*	*In favour of the combined therapy*	*One subject experienced skin redness after the first tDCS treatment.*	***Overall**: Good*<br><br>***QI***<br>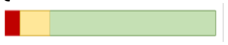	[158]
Transcranial direct current stimulation (tDCS) + Motor training + Cognitive training	*Post-stroke cognitive impairment*	*tDCS, DLFPFC (30)*<br><br>*vs*<br><br>*Motor training + Cognitive training (30)*<br><br>*vs*<br><br>*tDCS + Motor training + Cognitive training (tDCS simultaneously with cognitive rehabilitation) (30)*	*4 weeks (NP)*<br><br>*[total: ~6.7 hours tDCS, ~6.7 hours motor training, ~ 6.7 hours cognitive training]*	*<u>No primary outcomes</u>*<br><br>*<u>Outcomes</u>*<br>*Cognitive: yes*<br>*Functional: no*<br>*Patient-centred outcomes: no*	*In favour of the combined therapy*	*2 participants in the tDCS group experienced mild adverse reactions*	***Overall**: Fair*<br><br>***QI**:*<br>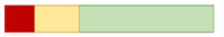	[123]
Transcranial ultrasound stimulation (TUS) + Cognitive training	*Post-stroke MCI*	*TUS + Cognitive training (30)*<br><br>*vs*<br><br>*Sham TUS + Cognitive training (30)*	*6 weeks (NP)*<br><br>*[total: ~10 hours TUS, cognitive training NR]*	*<u>No primary outcomes</u>*<br><br>*<u>Outcomes</u>*<br>*Cognitive: yes*<br>*Functional: yes*<br>*Patient-centred outcomes: no*<br>*Instrumental: Evoked potential, Blood works*	*In favour of the combined therapy*	*No available data on safety*	***Overall**: Poor*<br><br>***QI**:*<br>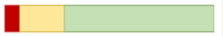	[129]

Notes:
$ Follow-up time refers to the period of observation between the end of treatment and up to the end of the study (i.e. not including the timespan of treatment administration). Other relevant treatment specifiers (e.g., number of sessions, session duration) have been reported in square brackets.
* Overall quality as rated according to the NIH Quality Assessment tools for controlled intervention studies is reported here. QI (Quality Index) is a graphical, colour-coded representation of the number of items on the scale rated respectively as at high-risk (red), unclear risk (yellow) or low-risk (green) of bias.

Abbreviations: *DTI, Diffusion Tensor Imaging; EEG, electroencephalogram; FDG-PET; fluorodeoxyglucose positron emission tomography; fMRI, functional magnetic resonance imaging of the brain; fNIRS, functional near-infrared spectroscopy; LOTCA, Lowenstein Occupational Therapy Cognitive Assessment; MMSE, Mini-Mental State Examination; MoCA, Montreal Cognitive Assessment; NIHSS, National Institute of Health Stroke Scale; NPS, neuropsychological tests; TC-US, transcranial ultrasound.*
NP, not performed; NR, not reported.

Rehabilitative interventions

Intervention	VCI population	Treatment arms	Treatment duration (follow-up)[$]	Outcomes	Efficacy	Quality score[*]	Study
Cognitive stimulation	*Vascular*	*Reminiscence group (17)*<br><br>*vs*<br><br>*Social contact group (11)*<br><br>*vs*<br><br>*Control group (inactive treatment, 17)*	*3 months*<br>*[total: ~ 12 hours]*<br><br>*(follow-up: NP)*	***Primary outcomes***<br>***none***<br><br>*Other outcomes*<br>*Cognitive: yes*<br>*Functional: yes*<br>*Patient-centred: no*	*Neutral*	***Overall**: Fair*<br><br>***QI**:*<br>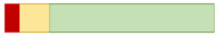	[143]
	Post-stroke	*Cognitive stimulation therapy group (10)*<br><br>*vs*<br><br>*Conventional rehabilitation group (10)*	*8 weeks*<br>*[total: ~ 53 hours]*<br><br>*(follow-up: NP)*	***Primary outcomes***<br>***Cognitive: MMSE***<br><br>*Secondary outcomes*<br>*Cognitive: no*<br>*Functional: yes*<br>*Patient-centred: yes*	*Neutral*	***Overall**: Good*<br><br>***QI**:*<br>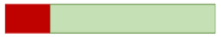	[152]
Virtual reality	*Post-stroke*	*Experimental group (18)*<br><br>*vs*<br><br>*Control group (17)*	*6 weeks*<br>*[total: ~ 15 hours]*<br><br>*(follow-up:3 months)*	***Primary outcomes***<br>***Cognitive: attention, memory, executive function, and spatial awareness composite scores***<br><br>*Secondary outcomes*<br>*Cognitive: yes*<br>*Functional: yes*<br>*Patient-centred: no*	*Partially in favour of treatment*	***Overall**: Good*<br><br>***QI**:*<br>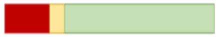	[136]
	Post-stroke	*Intervention group (15)*<br><br>*vs*<br><br>*Control group (15)*	*6 weeks*<br>*[total: ~ 9 hours]*<br><br>*(follow-up: NP)*	***Primary outcomes***<br>***none***<br><br>*Other outcomes*<br>*Cognitive: yes*<br>*Functional: yes*<br>*Patient-centred: no*	*Partially in favour of treatment*	***Overall**: Fair*<br><br>***QI**:*<br>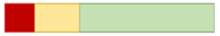	[140]
Physical activity + Occupational Therapy	*Post-stroke*	*Aerobic exercise group + Occupational therapy*	*2 weeks*<br>*[total: ~ 8 hours]*	***Primary outcomes***<br>***none***	*In favour of treatment*	***Overall**: Poor*<br><br>***QI**:*	[155]

**+ Acupuncture**		*+ motor rehabilitation + Acupuncture (10)*<br><br>*Vs*<br><br>*Occupational therapy + motor rehabilitation + Acupuncture (10)*	*(follow-up: NP)*	<u>*Other outcomes*</u><br>*Cognitive: yes*<br>*Functional: no*<br>*Patient-centred: no*		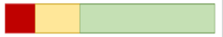	
**Motor rehabilitation**	*Post-stroke*	*Human–robotic interactive gait training (23)*<br><br>*vs*<br><br>*conventional physiotherapy (25)*	*6 weeks*<br>*[total: ~ 18 hours]*<br><br>*(follow-up: NP)*	<u>***Primary outcomes***</u><br>***none***<br><br><u>*Other outcomes*</u><br>*Cognitive: yes*<br>*Functional: yes*<br>*Patient-centred: no*	*Partially in favour of treatment*	***Overall:** Poor*<br><br>***QI:***<br>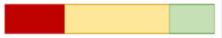	[164]
**Combined interventions**	*Vascular*	*Cognitive training group (10)*<br><br>*vs*<br><br>*Motor imagery + action observation group (10)*<br><br>*vs*<br><br>*Combined therapy group (10)*	*8 weeks*<br>*[total: ~ 26 hours]*<br><br>*(follow-up: 1 month)*	<u>***Primary outcomes***</u><br>***Cognitive: MoCA***<br><br><u>*Secondary outcomes*</u><br>*Cognitive: yes*<br>*Functional: no*<br>*Patient-centred: no*<br>*Instrumental: yes (ERP)*	*In favour of treatment*	***Overall:** Fair*<br><br>***QI:***<br>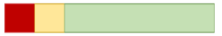	[141]
	Post-stroke	*Cognitive and motor training group (17)*<br><br>*vs*<br><br>*Cognitive training group (16)*	*4 weeks*<br>*[total: ~ 13 hours]*<br><br>*(follow-up: NP)*	<u>***Primary outcomes***</u><br>***Cognitive: MoCA, MMSE***<br><br><u>*Secondary outcomes*</u><br>*Cognitive: no*<br>*Functional: no*<br>*Patient-centred: no*<br>*Instrumental: yes (ERP, fNIRS)*	*In favour of treatment*	***Overall:** Fair*<br><br>***QI:***<br>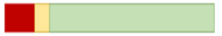	[144]
	Post-stroke	*Enriched rehabilitation group (20)*<br><br>*vs*<br><br>*Control group (20)*	*8 weeks*<br>*[total: ~ 96 hours]*<br><br>*(follow-up: NP)*	<u>***Primary outcomes***</u><br>***none***<br><br><u>*Other outcomes*</u><br>*Cognitive: yes*<br>*Functional: no*<br>*Patient-centred: no*<br>*Instrumental: yes (laboratory)*	*In favour of treatment*	***Overall:** Poor*<br><br>***QI:***<br>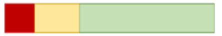	[145]

Personalised Music Playlist listening	*Post-stroke cognitive impairment*	*Personalised Music Playlist listening (18)*<br><br>*vs*<br><br>*White noise Playlist listening (18)*	*3 months*<br>[*total: ~ 90 hours*]<br><br>*(follow-up: NP)*	<u>*Primary outcomes*</u><br>*Cognitive: MoCA*<br>*Functional: no*<br>*Patient-centred: no*<br><br><u>*Secondary outcomes*</u><br>*Cognitive: yes*<br>*Functional: yes*<br>*Patient-centred: yes*	*In favour of treatment*	***Overall**: Good*<br><br>***QI**:* 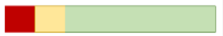	[157]

Notes:
$^{\$}$ Follow-up time refers to the period of observation between the end of treatment and up to the end of the study (i.e. not including the timespan of treatment administration). Other relevant treatment specifiers (e.g., number of sessions, session duration) have been reported in square brackets.
* Overall quality as rated according to the NIH Quality Assessment tools for controlled intervention studies is reported here. QI (Quality Index) is a graphical, colour-coded representation of the number of items on the scale rated respectively as at high-risk (red), unclear risk (yellow) or low-risk (green) of bias.

Abbreviations: *ERP, event-related potential; fNIRS, functional near-infrared Spectroscopy; MMSE, Mini-Mental State Examination; MoCA, Montreal Cognitive Assessment.*

NP, not performed; NR, not reported.

3.2.2 *eTable 7* - Interventions, not included in meta-analyses, evaluated in subcortical ischemic (SVD-related) cognitive impairment

Pharmacological interventions

Intervention	VCI population	Treatment arms	Treatment duration (follow-up)[$]	Outcomes	Efficacy	Safety profile	Quality score*	Study
Butylphtalyde	*Subcortical vascular MCI*	*Butylphtalyde 600 mg (140)*<br><br>*vs*<br><br>*Placebo (140)*	*24 weeks (NP)*	*Primary outcomes*<br>*Cognitive: ADAS-Cog*<br>*Functional: CIBIC-plus*<br>*Patient-centred outcomes: no*<br><br>*Secondary outcomes*<br>*Cognitive: yes*<br>*Functional: no*<br>*Patient-centred outcomes: no*	*In favour of the treatment*	*No available data on safety*	***Overall**: Good*<br><br>***QI**:*	[39]
Choline alphoscerate + Nimodipine	*Subcortical Vascular dementia*	*Choline alphoscerate 1200 mg + Nimodipine 90 mg (24)*<br><br>*vs*<br><br>*Placebo + Nimodipine 90 mg (24)*	*12 months (NP)*	*Primary outcomes*<br>*Cognitive: MoCA*<br>*Functional: no*<br>*Patient-centred outcomes: no*<br><br>*Secondary outcomes*<br>*Cognitive: yes*<br>*Functional: yes*<br>*Patient-centred outcomes: yes*	*Neutral*	*8 patients referred a total of 14 symptoms compatible with an adverse reaction, of which 13 fell within those known for the drugs; none was classified as serious.*	***Overall**: Fair*<br><br>***QI**:*	[3]

Notes:
[$] Follow-up time refers to the period of observation between the end of treatment and up to the end of the study (i.e. not including the timespan of treatment administration).
* Overall quality as rated according to the NIH Quality Assessment tools for controlled intervention studies is reported here. QI (Quality Index) is a graphical, colour-coded representation of the number of items on the scale rated respectively as at high-risk (red), unclear risk (yellow) or low-risk (green) of bias.

Abbreviations:
Abbreviations: ADAS-Cog, Alzheimer's Disease Assessment Scale – cognitive subscale; CIBIC-plus, Clinician's Interview-Based Impression of Change Plus caregiver input; MoCA, Montreal Cognitive Assessment.

NP, not performed; NR, not reported.

Non-pharmacological, non-rehabilitative interventions (application of physical devices)

Intervention	VCI population	Treatment arms	Treatment duration (follow-up)$	Outcomes	Efficacy	Safety	Quality score*	Study
Repetitive transcranial magnetic stimulation (rTMS) + Donepezil	*Subcortical vascular cognitive impairment*	*rTMS, left DLFPC + Donepezil 10 mg (58)*<br><br>*Vs*<br>*Donepezil 10 mg (57)*	*4-6 weeks (4 weeks)*<br><br>*[total: 28-42 sessions rTMS]*	*Primary outcomes*<br>*Cognitive: MMSE, MoCA*<br>*Functional: no*<br>*Patient-centred outcomes: no*<br><br>*No secondary outcomes*	*In favour of the combined therapy*	*No statistically significant difference in the incidence of adverse events in the two groups.*	***Overall**: Fair*<br><br>***QI:***<br>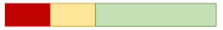	[150]

***Notes**:*
$ Follow-up time refers to the period of observation between the end of treatment and up to the end of the study (i.e. not including the timespan of treatment administration). Other relevant treatment specifiers (e.g., number of sessions, session duration) have been reported in square brackets.
** Overall quality as rated according to the NIH Quality Assessment tools for controlled intervention studies is reported here. QI (Quality Index) is a graphical, colour-coded representation of the number of items on the scale rated respectively as at high-risk (red), unclear risk (yellow) or low-risk (green) of bias.*

***Abbreviations:** MMSE, Mini-Mental State Examination; MoCA, Montreal Cognitive Assessment.*
NP, not performed; NR, not reported.

Other interventions

Intervention	VCI population	Treatment arms	Treatment duration (follow-up)$	Outcomes$	Efficacy	Safety	Quality score*	Study
Stellate ganglion block	*Subcortical Vascular Cognitive Impairment*	*Stellate ganglion block*<br>*1 session/day (42)*<br><br>*vs*	*20 days (NP)*<br><br>*[total: 20 sessions ganglion block]*	*Primary outcomes:*<br>*Other: Primary Aspiration Scale*<br>*Cognitive: no*<br>*Functional: no*<br>*Patient-centred outcomes: no*	*In favour of the treatment*	*No severe adverse events were reported.*	***Overall**: Good*<br><br>***QI:***<br>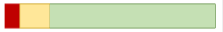	[147]

	Best medical treatment (40)	*<u>Outcomes</u>*<br>*Cognitive: yes*<br>*Functional: yes*<br>*Patient-centred outcomes: no*		

Notes:

$ Follow-up time refers to the period of observation between the end of treatment and up to the end of the study (i.e. not including the timespan of treatment administration). Other relevant treatment specifiers (e.g., number of sessions, session duration) have been reported in square brackets.

***** Overall quality as rated according to the NIH Quality Assessment tools for controlled intervention studies is reported here. QI (Quality Index) is a graphical, colour-coded representation of the number of items on the scale rated respectively as at high-risk (red), unclear risk (yellow) or low-risk (green) of bias.

4. SUPPLEMENTARY FILES

Supplementary File 1: List of Cochrane Reviews retrieved and screened by systematic search

Supplementary File 2: List of excluded full texts

Supplementary File 3: List of all included studies

List of all included with detailed bibliographic data (including the unique number identifier, which is consistently referenced throughout other tables), and overview of patients included (population and study size), interventions investigated, comparator employed, and outcomes evaluated.

Supplementary File 4: Quality assessment according to NIH-QAT for controlled studies

Consensus quality assessment according to National Institute of Health Quality Assessment Tool of Controlled Intervention Studies for each of the included studies, broken down and colour-coded for each item.

Supplementary File 5: List of studies not included in semi-quantitative estimation

5. SUPPLEMENTARY BIBLIOGRAPHY

1. Page MJ, McKenzie JE, Bossuyt PM, et al. The PRISMA 2020 statement: an updated guideline for reporting systematic reviews. *BMJ*. Mar 29 2021;372:n71. doi:10.1136/bmj.n71
2. Higgins JPT, Thomas J, Chandler J, et al. Cochrane Handbook for Systematic Reviews of Interventions version 6.5 (updated August 2024). . *Cochrane*. 2024;
3. Sawilowsky SS. New Effect Size Rules of Thumb. *Journal of Modern Applied Statistical Methods*. 2009;8:26.
4. Schünemann H, Brożek J, Guyatt G, Oxman A. *GRADE Handbook*. 2013.
